# Implementation of *in silico* methods to predict common epitopes for vaccine development against Chikungunya and Mayaro viruses

**DOI:** 10.1016/j.heliyon.2021.e06396

**Published:** 2021-03-08

**Authors:** Hammadul Hoque, Rahatul Islam, Srijon Ghosh, Md. Mashiur Rahaman, Nurnabi Azad Jewel, Md. Abunasar Miah

**Affiliations:** a Department of Genetic Engineering and Biotechnology, School of Life Sciences, Shahjalal University of Science and Technology, Sylhet, Bangladesh; b Department of Biotechnology and Genetic Engineering, Noakhali Science and Technology University, Noakhali, Bangladesh

**Keywords:** Chikungunya virus, Mayaro virus, Epitopes, Vaccine

## Abstract

Being a Positive sense RNA virus the recent reemergence of Chikungunya and Mayaro virus has taken the concern of the leading scientific communities of the world. Though the outbreak of Mayaro virus is limited to Neotropical region only, Chikungunya is already identified in over 60 countries around the world. Besides, the lack of a strong protective treatment, misdiagnosis issue and co-circulation of both the viruses calls for a new strategy which could potentially prevent these infections from spreading. In this study, we therefore, identified the peptide based vaccine candidates e.g. epitopes for B cell and T cell from Chikungunya virus which also showed to be homologous to the Mayaro virus through immuno-informatics and computational approaches. Final epitopes identified from the most antigenic structural polyprotein of both the viruses were 5 for CD8+ T cell Epitopes (^209^KPGDSGRPI^217^, ^219^TGTMGHFIL^227^, ^239^ALSVVTWNK^247^, ^98^KPGRRERMC^106^ and ^100^GRRERMCMK^108^), 2 epitopes for CD4+ T cell (^105^MCMKIENDCIFEVKH^119^ and ^502^DRTLLSQQSGNVKIT^516^) and a single epitope for B cell (^504^GGRFTIPTGAGKPGDSGRPI^518^). Analysis of our predicted epitopes for population coverage showed prominent population coverage (92.43%) around the world. Finally, molecular docking simulation of the foreseen T cell epitopes with respondent HLA alleles secured good HLA-epitope interaction. This study was directed towards the discovery of potential antigenic epitopes which can open up a new skyline to design novel vaccines for combating both of the diseases at the same time.

## 1 Introduction

After the first identification in Tanzania and Trinidad during the mid-1950s, the recent outbreak of Chikungunya (CHIKV) and Mayaro virus (MAYV) has again taken the concern of the health authorities around the world [1]. These viruses are mainly circulated by two of the species of mosquito- *Aedes aegypti* and *Aedes albopictus* (commonly known as Yellow Fever Mosquito and Asian Tiger Mosquito respectively), while recent study shows that, MAYV can be transmitted by Anopheles mosquito as well [2, 3, 4]. Both of the viruses shares the same genus (Alphavirus) and same family (Togaviridae) with direct hereditary and immunogenic connection and cause an acute febrile illness akin to each other. Even sometimes, MAYV is often misdiagnosed with CHIKV as both of the viruses causes similar symptoms including fever, headache, myalgia, rash, arthralgia and, mostly, arthritis. While Chikungunya is rapidly spreading around the world mostly in the tropical region, the former virus (MAYV) is known to be circulated in the Neotropical (South America, Peru Brazil) region only [5, 6]. However, Chikungunya has become one of the major burdens after its reemergence in Europe and America since 2000 with an estimated 3 million confirmed cases reported each year [7]. Previously the disease was typical in the developing countries mostly in Asia and Africa but a recent outbreak of the disease in America with 1,118,763 suspected cases in 2013–14 confirmed that the virus is now circulating around the whole world [8]. On the other hand, the MAYV infection was regarded as an acute, self-limited dengue like illness, however, recent outbreaks in 2010 in Venezuela and 2014–16 in Brazil and has led the scientific community to mark it as a global threat [9, 10]. Furthermore, a recent case in Haiti reported co-infection of the virus with dengue makes it much more concerning issue for the leading scientific community [11].

CHIKV genome can be characterized as an (+)-sense ssRNA genome of 11.6 kb in length that encodes 5 structural proteins (capsid protein, E3, E2, E1 and 6K) and 4 non-structural polyproteins (NSP1, NSP2, NSP3 and NSP4) [12]. MAYV also possess the similar structure characterized by an (+)-sense ssRNA genome of approximately 11.4 kb in length which is slightly shorter than CHIKV and encoding the same structural and non-structural polyproteins as the CHIKV or other alphaviruses [13] encode. Furthermore, CHIKV genotypes are extensively disseminated throughout the biosphere with three discrete lineages e.g. Asian, East/West/South and Central African genotypes whereas MAYV lineages are self-limited and circulated in two lineages (D and L) in the state of South America only [6, 14].

At present there are no approved definite vaccines at hand for CHIKV and MAYV, while scientist are trying hard to develop a specific treatment for these viruses. Only a live attenuated vaccine for CHIKV is now currently available in phase-2 trial but 85% of test showed resistance after one year [15]. Therefore, there is a crying need for a new vaccine candidate which should be highly immunogenic against both of these viruses. However, a recent research on a novel CHIKV recombinant vaccine (CHIKV/IRES) which stimulated immunity against another immunogenically related species named o'nyong-nyong virus (ONNV) suggested that vaccine developed for related antigenic strains of CHIKV can induce cross-neutralizing antibodies for both of the viruses [16]. That's why this study was designed to find out the prominent epitopes for CHIKV which are also conserved and homologous to MAYV and thus could be hypothesized to produce cross-neutralizing antibodies for both the viruses in vivo. Furthermore, in this study, we have also checked the possibility of cross reaction of our epitopes with other arboviruses (Zika Virus, Dengue Virus). We anticipated that, our predicted epitopes could aid as a prospective pathogen specific vaccine candidate with wide-ranging therapeutic applications against CHIKV/MAYV associated diseases.

## 2 Methodology

### 2.1 Retrieval of protein sequences from data bank

The structural polyprotein of both of the viruses (Chikungunya and Mayaro) were targeted for epitope prediction/selection as the viral structural proteins are involved in cell entry and subsequent fusion of the virus inside host cells.

Hence, the Swiss-Prot reviewed structural polyprotein sequences of both the viruses (Chikungunya and Mayaro) were regained from UniProt Knowledge Base (UniProtKB) database (https://www.uniprot.org/) and then saved in FASTA format for further antigenic properties analysis**.**

### 2.2 Selection of antigenic proteins

A toxin or other foreign components that stimulates the body's immune response, particularly in antibody production are known as antigens. To assess the best possible antigen, all the retrieved structural polyproteins of both of the viruses were subjected to computational antigenicity investigation in widely applied server known as VaxiJen2.0 (http://ddg-pharmfac.net/vaxijen/VaxiJen/VaxiJen.html) which is able to calculate antigenic proteins more than 80% accurately [17]. The cut off value for antigenic protein prediction was set at 0.4 which is default. Thus, non-antigenic polyprotein sequences were omitted in this study and antigenic proteins were selected and listed conforming to their orders. The VaxiJen results for the top antigenic polyproteins of both viruses were then selected for further analysis i.e., physiochemical parameters, prediction of B cell and T cell epitopes, conservancy of the epitopes as well as population coverage analysis of the epitopes, epitope modelling, protein-peptide docking and some other standards parameters (Allergenecity, Toxicity) were measured as prominent epitopes of a vaccine candidate.

### 2.3 Physicochemical characterization of the selected proteins

To identify the functional physiochemical parameters of the selected antigenic polyprotein, a widely used tool of Expasy server (http://expasy.org/cgi-bin/protpraram) named ProtParam was employed [18]. ProtParam can measure different parameters, such as helices, coils, molecular weight (MW), composition of amino acids, stability, theoretical p, hydrophilic property that are vital in evaluating the antigenicity of the protein. After characterizing the physicochemical parameters, the highest antigenic structural polyprotein of Chikungunya virus was selected as referral protein for further prediction of B cell and T cell epitopes.

### 2.4 Prediction of CD8+ T-cell Epitope

NetCTL 1.2 (http://www.cbs.dtu.dk/services/NetCTL/) was used to identify CD8+ or cytotoxic T cell (CTL) epitopes from the selected structural polyprotein of Chikungunya virus [19] depending on the different supertypes of major histocompatibility complex class I (MHC class I) viz., A1, A2, A3, A24, A26B7, B8, B27, B39, B44, B58, and B62 as well as peptide binding. The default threshold value of 0.75 was employed, so that we can be assured that the findings could be more conclusive for generating most potential epitopes. The best epitopes for vaccine candidates were also selected by combining score of transporter antigenic peptides (TAP) transport efficiency and proteasomal cleavage prediction from the NetCTL 1.2 server.

### 2.5 Prediction of CD4+ T-cell epitopes

The 15 mer CD4+ or helper T cell epitopes (HTL) of the structural polyprotein of Chikungunya virus were identified using IEDB webserver (http://tools.iedb.org/mhcii/) which is well known prediction tool for MHC II Binding. A total of 7 reference set of human HLA alleles (HLA-DRB1∗03:01, HLA-DRB1∗07:01, HLA-DRB1∗15:01, HLA-DRB3∗01:01, HLA-DRB3∗02:02, HLA-DRB4∗01:01 and HLA-DRB5∗01:01) from the same server were selected for CD4+ T cell epitope prediction [20]. To assess IC50 (half maximal inhibitory concentration) value of the peptide binds to MHC- II alleles, stabilized matrix method (SMM) [21] was utilized. The standard peptide affinity measurements were followed, viz., IC50 values <50nM considered as higher affinity, the IC50 value < 500nM considered as intermediate affinity and IC50 < 5000nM indicated lower affinity of the epitopes. However, we shortlisted the epitopes by assigning a cut off value of IC50 < 250nM.

### 2.6 Prediction of B cell epitopes

Linear B cell lymphocyte (BCL) epitopes of the selected Chikungunya structural polyprotein were identified from the BCPRED webserver (http://ailab.ist.psu.edu/bcpred/) [22]. This server has three alternative methods to predict B cell epitopes e.g. (i) AAP method (ii) BCPred (iii) FBCPred. To determine B cell epitopes, BCPred method was followed in this study due to its higher performance (AUC 0.758) comparison to other two methods [23] and the cut-off score >0.8 was preferred to get peptides resemble to maximum epitope-like properties.

### 2.7 Conservancy analysis across reference variants

#### 2.7.1 Conservancy analysis across reference variants

The epitopes for vaccine candidate against Chikungunya virus that were preliminary identified by different computational studies only designed on the basis of CHIKV structural polyprotein for the selection of B cell and T cell epitopes. To show potency as vaccine candidate for MAYV, all of the predicted epitopes from CHIKV should be match as a conserved and homologous sequence in the structural polyprotein of MAYV as well. That's why all the predicted epitopes from CHIKV structural polyprotein were also screened through structural polyprotein of MAYV to recognize the conserved epitopes in both of the viruses.

Furthermore after conservancy analysis, the desired conserved epitopes of both of the viruses were then again ran in the conservancy analysis tool against Dengue Virus (Type-1,2,3 and 4) and Zika Virus's reference polyprotein to check if any of the epitope is conserved and similar in those polyprotein or not. If our predicted epitopes are matched in these polyproteins, there may be a cross reaction possibility can be occurred.

#### 2.7.2 Conservancy analysis across all variants

After conservancy analysis in the reference variants, we also screened the conserved and homologous epitopes of both of the viruses (CHIKV and MAYV) in the all representative sequence of all the variants. For this, first of all we have identified all the representative sequences of both of the viruses' structural polyprotein from NCBI database. A total of 100 and 64 sequences were found and retrieved as a representative sequence of CHIKV AND MAYV respectively.

However, to get epitope conservancy patterns of the shortlisted and promising epitopes, online based tool viz., conservancy analysis at the IEDB (Immune Epitope Database) was used (http://tools.iedb.org/conservancy/) for the mayaro virus structural polyprotein [24]. The threshold value for the sequence identity was assigned at 100% and every epitopes that meet the threshold were filtered for subsequent inquiry.

### 2.8 Antigenicity, allergenicity, toxicity and immunogenicity assessment

As the epitope will be directly applied to human as vaccine candidates, thus the choice of non-allergenic, non-toxic and highly antigenic epitope identification was one of our prime aims.

Thus, the VaxiJen (http://www.ddg-pharmfac.net/vaxijen/), AllergenFP (https://ddg-pharmfac.net/AllergenFP/), and ToxinPred (http://crdd.osdd.net/raghava/toxinpred/) webservers were applied consecutively to find out the most appropriate antigenic epitopes, the allergenic and toxic activity of the short listed B cell and T-cell epitopes respectively [17, 25, 26]. Some epitopes were ignored due to their unfulfillment of the threshold level at the VaxiJen server. On the basis of the assessment scores in each of the webservers mentioned previously, only harmless (nontoxic and non-allergenic) epitopes were filtered. Immunogenicity was also predicted for helper T cell epitopes, as during a viral infection, it stimulates the release of diverse cytokines like the interferon alpha (IFN-α), interferon beta (IFN-β), interferon-gamma (IFN-γ), IL-4, IL-10. These cytokines later activate other immune cells e.g. macrophages, natural killer cells, cytotoxic T-cells upon activation [27]. Thus, the IFNepitope (http://crdd.osdd.net/raghava/ifnepitope/) server was used to predict IFN-γ induction capability of the proposed epitopes [28]. In addition, the interleukin-4 and interleukin-10 stimulating property of the helper T leucocytes epitopes were also predicted using two well-known webservers “IL4pred” [29] and “IL10pred” [30].

### 2.9 Determination of MHC-I and MHC-II alleles

Though NetCTL 1.2 predicted the possible CD8+ T cell epitopes from any protein that has antigenic property, it can't predict the corresponding allele to these epitopes. That's why the Major Histocompatibility Complex (MHC)/Human Leukocyte Antigens (HLA) interacting with selected individual CTL epitope from the structural polyprotein of CHIKV were selected by using http://tools.iedb.org/mhci/which is a MHC-I predicting interface at the IEDB server [31]. The IEDB recommended approach (default) can calculate the binding affinity of the epitopes to all 27 HLA reference set of MHC I alleles included in the server [32]. The output of the MHC-I binding prediction tool provide a percentile rank as an affinity marker for each of the epitope-allele complexes. Lower percentile rank exhibits good binding prediction so that we fixed the base value of percentile rank at 10 to assess epitope binding to all the major histocompatibility complex class-I alleles. Further, we also predict the alleles that can interact with individual HTL epitopes from the IEDB recommended 7 reference set of human HLA allele (HLA-DRB1∗03:01, HLA-DRB1∗07:01, HLA-DRB1∗15:01, HLA-DRB3∗01:01, HLA-DRB3∗02:02, HLA-DRB4∗01:01 and HLA-DRB5∗01:01). However we set the cut-off value of percentile rank at 20.0 according to IEDB recommendation so that these alleles could be used for broad epitope prediction [20].

### 2.10 Population coverage analysis

The population coverage of a vaccine candidate indicates its wideness of fortification against the respective disease among various ethnics in the world for effective vaccination. But in reality it is quite difficult to make a broad spectrum vaccine that would cover all countries and races due to the high degree of polymorphism of major histocompatibility complex molecules (approximate 6000). Thus, the binding ability of epitopes with numerous HLA could raise population coverage as well as effective for further shortlisting of the epitopes.

Therefore, the population coverage tool (http://tools.iedb.org/population/) provided by the IEDB server [33] has used to discover the population coverage of our epitopes in a distinct population/area based on HLA genotypic frequencies. Each population covers different sets of HLA molecules so that population coverage analysis is required. However, among three different algorithmic calculation methods namely (1) HLA class I, (2) HLA class II and (3) combined HLA class I and class II, that are available in the server, we chose the first one (HLA class-I) for our final suggested CTL epitopes (CD8+ T cell epitopes) population coverage analysis. We couldn't analyze the HLA class-II population coverage analysis of our HTL epitopes (CD4+ T cell epitopes) as the corresponding “HLA-DR” alleles against CD4+ T cell epitopes found in our study weren't updated yet in the IEDB population coverage analysis tool.

### 2.11 3D structures prediction of T-cell epitopes and chosen HLA molecule

PEP-FOLD3 (https://bioserv.rpbs.univ-paris-diderot.fr/services/PEP-FOLD/) was exploited to build the precise three dimensional structure in PDB format of the selected CD8+ and CD4+ epitopes [34]. PEP-FOLD3 which is a peptide structure prediction server can predict 3D structures from amino acid sequences by de novo method and it can also guess the 3D structures of small linear peptides by following SA (structural Alphabet) based mode of the peptides coupling with a greedy algorithm and a coarse-grained force field.

The interaction of the obtained T cell epitopes with MHC-I allele of HLA-B∗07:02, HLA-B∗51:01, HLA-B∗53:01, HLA-B∗35:01, HLA-B∗08:01 and HLA-B∗44:02 were observed in all cases with minimum IC50 value. But, we chose one of the MHC-I allele (HLA-B∗07:02) from the above list for docking analysis as the availability of the crystal structure of the protein in Protein Data Bank (PDB ID: 6AT5).

Further, the MHC- II alleles of HLA-DRB1∗15:01, HLA-DRB1∗07:01, HLA-DRB5∗01:01, HLA-DRB3∗02:02 were also observed to interact with the final CD4+ T cell epitopes. However, we chose the HLA-DRB1∗15:01 allele for docking analysis for the same reason describe above (PDB ID: 1BX2). However, a refinement of the whole protein structure is required prior to docking for improving the docking analysis beyond the precision level. So, we followed a standardized Protocol for molecular docking used in previous studies e.g. elimination of previously attached peptide, protonation, energy reduction followed by elimination of H_2_O molecules from the retrieved crystal structure [35, 36]. Energy minimization and refinement were performed by using a webserver based tool named Modrefiner [37]. After refinement, we used the PROCHECK webserver (https://servicesn.mbi.ucla.edu/PROCHECK/) for Structural validation of the refined HLA proteins [38].

### 2.12 Molecular docking analysis

Though the interaction of the final CD8+ and CD4+ T cell epitopes with the corresponding HLA (MHC-I & MHC-II) can be predicted by IEDB webserver, the HLA-Epitope interaction should be examined by analyzing *in silco* molecular docking simulation. That's why, a structure-based docking analysis was conducted between the 3D structure of CTL or HTL epitope and respective HLA allele (MHC- I and MHC- II) on PyRx interface [39], a collective platform by combining AutoDOCKVina, AutoDOCK4.2, Mayavi and Open Babel which was used to calculate the universal binding energy of the protein-peptide complex. We used a 13 amino acid long tumor associated peptide NY-ESO-1 (“APRGPHGGAASGL”) as a control epitope. The readily available crystal structure of the NY-ESO-1 peptide- HLA-B∗07:02 complex (PDB ID: 6AT5) led us to choose these epitope as a control for MHC- I binding in our study. As well as, in case of MHC- II complex, the control epitope used was 15 amino acid long myelin basic protein (“ENPVVHFFKNIVTPR”) for HLA-DRB1∗15:01 recognition (PDB ID: 2Q6W).

For docking, PDB files of both T cell epitopes (CTL, HTL and controls) and corresponding alleles (MHC- I and MHC- II) were uploaded to the PyRx AutoDockvina wizard. After successful completion, the docked complexes were then visualized in discovery studio visualizer.

## 3 Results

### 3.1 Target protein selection

The current study was designed on the basis of the available proteomic and genomic data of CHIKV and Mayaro virus's polyproteins. A total of 4 Swiss-Prot reviewed CHIKV structural polyproteins were found in the Uniprot Database. Then the antigenicity values of all 4 reviewed proteins were computed by auto cross covariance (ACC) conversion at Vaxijen webserver. All subjected proteins exhibited different antigenic value ranging from 0.5163 in CHIKV structural polyproteins of strain S27-African prototype to 0.5319 in CHIKV Frameshifted structural polyprotein (strain S27-African prototype) (Table 1). As Frameshifted structural polyprotein of CHIKV (strain S27-African prototype) (UniprotKB ID: P0DOK1) had shown the most antigenicity, these polyprotein was selected as a referral protein from CHIKV for further studies. Further, in case of Mayaro virus only one Swiss-Prot reviewed structural polyprotein was found which is Structural polyprotein of Mayaro virus (strain Brazil) (UniprotKB ID: Q8QZ72). Thus these polyprotein was selected as a referral protein for cross protecting epitope selection against Mayaro virus where the antigenicity value (0.5270) of this protein was found quite similar to the CHIKV Framshifted Structural Polyprotein. Furthermore, pairwise sequence alignment of both of this polyprotein through Emboss Needle- Pairwise Sequence Alignment tool revealed that, the sequence similarities between these sequences are 47.9%. The dataset for reference structural polyprotein of CHIKV (UniprotKB ID: P0DOK1) and MAYV (UniprotKB ID: Q8QZ72) can be found from Supplementary File 1.

**Table 1:** Antigenicity values of all Swiss-Prot reviewed CHIKV polyproteins. Framshifted structural polyprotein (UniprotID: P0DOK1) was found as most antigenic.

Protein names	Uniprot ID	Organism	VaxiJen score	Remarks
Structural polyprotein	Q5XXP3	Chikungunya virus (strain 37997)	0.5278	Antigenic
Structural polyprotein	Q5WQY5	Chikungunya virus (strain Nagpur)	0.5241	Antigenic
Frameshifted structural polyprotein	P0DOK1	Chikungunya virus (strain S27-African prototype)	0. 5319	Antigenic
Structural polyprotein	Q8JUX5	Chikungunya virus (strain S27-African prototype)	0.5163	Antigenic

### 3.2 Physicochemical characterization of the target proteins

ProtParam tools of the Expassy server disclosed many crucial properties of the top ranked antigenic polyprotein of both of the viruses. For example, the instability indexes of both of the highest antigenic polyproteins were not found as satisfactory but both of the polyproteins showed long longevity *in vitro* (half-life 30 h) in mammalian reticulocytes. Further, ProtParam tools predicted other physicochemical characteristics of both of the polyproteins such as molecular weight, theoretical pI, aliphatic index and Grand average of hydropathicity (GRAVY) value which are shown in Table 2. Analysis of ProtParam tools also revealed that, though the amino acid composition or amino acid numbers of both of the polyproteins are not same, both of the polyproteins showed almost similar physicochemical characteristics naturally.

**Table 2:** Physicochemical Characterization of CHIKV and Mayaro virus's final selected structural polyprotein. Both of the proteins possess almost similar characteristics.

Criteria	Score (CHIKV)	Score (MAYV)
Number of amino acids	838	1263
Molecular weight	92619.66	137057.14
Total number of atom	12955	19141
Formula	C_4080_H_6463_N_1183_O_1175_S_54_	C6057H9528N1704O1777S75
Theoretical pI	9.25	8.96
Estimated half-life in mammalian reticulocytes, in vitro	30 h	30 h
Instability index	40.97	43.07
Aliphatic index	72.06	73.89
Grand average of hydropathicity (GRAVY)	-0.270	-0.270

### 3.3 T-cell Epitope prediction (CD8+ & CD4+)

For identification of T cell epitopes from both of the viruses, we used the CHIKV Frameshifted structural polyprotein as the referral protein. Then, in the conservancy analysis, we predicted the overlapped epitopes which found homologous to the Mayaro virus's structural polyprotein.

Initially NetCTL 1.2 webserver predicted 245 potential CD8+ T cell epitopes or CTL (9 mer) contrary to 12 major histocompatibility complex super-classes (Supplementary Table 1) from the Frameshifted structural polyprotein of CHIKV. The threshold for the prediction was set at default (0.60).

Further, IEDB webserver predicted 306 CD4+ T cell epitopes or HTL (15 mer) against 7 reference set of human HLA allele from CHIKV Frameshifted structural polyprotein (Supplementary Table 2).

### 3.4 B-cell Epitope prediction

At 0.8 cut-off value, BCPRED webserver predicted 22 B cell epitope from the Frameshifted structural polyprotein of CHIKV (Supplementary Table 3). However, the epitopes predicted by all of the webservers were filtered through several pipelines (e.g. Conservancy, antigenic property, allergenic property and toxicity inquiry) to sort out the most potential epitopes in subsequent steps.

### 3.5 Conservancy analysis

#### 3.5.1 Conservancy analysis across reference variants

IEDB provided conservancy analysis tool (http://tools.iedb.org/conservancy/) was applied to observe the pattern of each of the B cell epitopes and T cell epitopes in the protein sequence of the Mayaro virus's structural polyprotein to filter out the conserved common homologous epitopes of both of the viruses (CHIKV and MAYV). Epitopes which showed 100% conservancy were selected for further analysis. Thus, after conservancy analysis, a total of 9 (nine) CTL, 10 (ten) HTL and 2 (two) B cell epitopes were found showing 100% conservancy in the structural polyprotein of both CHIKV and MAYV (Table 3).

**Table 3:** Conservancy analysis of the CHIKV epitopes in the MAYV structural polyprotein.

CD8+ T cell Epitope (CTL)			
Epitope	Conservancy_Hit CHIKV	Conservancy_Hit MAYV	Combined Score
KVTGYACLV	100.00% (1/1)	100.00% (1/1)	0.869
ALSVVTWNK	100.00% (1/1)	100.00% (1/1)	1.2483
KYDLECAQI	100.00% (1/1)	100.00% (1/1)	0.9517
KPGDSGRPI	100.00% (1/1)	100.00% (1/1)	1.6136
KPGRRERMC	100.00% (1/1)	100.00% (1/1)	0.8161
RRERMCMKI	100.00% (1/1)	100.00% (1/1)	1.2916
GRRERMCMK	100.00% (1/1)	100.00% (1/1)	0.9834
TGTMGHFIL	100.00% (1/1)	100.00% (1/1)	0.9569
FEVKHEGKV	100.00% (1/1)	100.00% (1/1)	1.0928
CD4+ T cell Epitope (HTL)			
Epitope	Conservancy_Hit CHIKV	Conservancy_Hit MAYV	SMM IC50 Value
ALSVVTWNKDIVTKI	100.00% (1/1)	100.00% (1/1)	162
LSVVTWNKDIVTKIT	100.00% (1/1)	100.00% (1/1)	162
VVTWNKDIVTKITPE	100.00% (1/1)	100.00% (1/1)	165
SVVTWNKDIVTKITP	100.00% (1/1)	100.00% (1/1)	166
VTWNKDIVTKITPEG	100.00% (1/1)	100.00% (1/1)	170
MCMKIENDCIFEVKH	100.00% (1/1)	100.00% (1/1)	187
RTLLSQQSGNVKITV	100.00% (1/1)	100.00% (1/1)	205
DRTLLSQQSGNVKIT	100.00% (1/1)	100.00% (1/1)	209
PDRTLLSQQSGNVKI	100.00% (1/1)	100.00% (1/1)	211
TLLSQQSGNVKITVN	100.00% (1/1)	100.00% (1/1)	240
B cell Epitope (BCL)			
Epitope	Conservancy_Hit CHIKV	Conservancy_Hit MAYV	Score
GGRFTIPTGAGKPGDSGRPI	100.00% (1/1)	100.00% (1/1)	1
LVGDKVMKPAHVKGTIDNAD	100.00% (1/1)	100.00% (1/1)	0.752

After conservancy analysis the predicted 9 (nine) CTL, 10 (ten) HTL and 2 (two) B cell epitopes were then again ran in the IEDB conservancy analysis tool against Dengue Virus and Zika virus's reference polyprotein (NCBI accession no: NP_059433.1, NP_056776.2, YP_001621843.1, NP_073286.1 and YP_002790881.1) to see if these epitopes were similar to this sequences or not. If any of the epitope was similar, then, there may be a cross reaction could occur. However, none of these epitopes showed conservancy against Dengue virus and Zika virus's referral proteome (Supplementary Table 4). So, it can suggest that there is no possibility of this epitope to be cross reactive with other arboviruses. The dataset for Dengue Virus and Zika virus's referral polyprotein can be found from Supplementary File 1.

#### 3.5.2 Conservancy analysis across all variants

We also screened all the predicted conserved and homologous epitopes (9 CTL, 10 HTL and 2 B cell epitopes) of CHIKV and MAYV in the all representative sequence of all variants. Maximum epitopes in our study were found as 100% conserved in all the 100 variants of CHIKV. However, for MAYV, out of 64 representative sequences, maximum 49 variants showed 100% conservancy for all the epitopes (Supplementary Table 5). As, none of our epitopes showed cross reaction against Dengue and Zika viruses referral proteome, we have omitted the analysis this for these viruses.

### 3.6 Analysis of antigenicity, allergenicity, toxicity and immunogenicity

The predicted conserved epitopes from the previous steps needs to be analyzed for antigenicity, allergenicity, toxicity and immunogenicity properties prior to concede as potential vaccine candidates.

Thus, subsequent analysis by VaxiJen webserver, ToxinPred and AllergenFP, we filtered out the non-antigenic, allergenic and toxic epitopes. These result in final 5 (five) CTL (CD8+) epitopes, 3 (three) HTL (CD4+) epitopes and 1 (one) B cell epitopes (Table 4). Additionally, immunogenicity analysis of the CD4+ T cell epitopes by IFNepitope, IL-4 and IL-10 webserver sorted out final 2 HTL epitopes (^105^MCMKIENDCIFEVKH^119^ and ^502^DRTLLSQQSGNVKIT^516^) from the above list by eliminating the non-immunogenic epitope ^504^TLLSQQSGNVKITVN^518^ (Table 5).

**Table 4:** Antigenicity, allergenicity and toxicity analysis of the conserved epitopes of both of the viruses.

CD8+ T cell Epitope (CTL)					
Peptide sequence	Antigenicity	Score	Allergenicity	Toxicity	Position
KPGDSGRPI	A	1.1546	NA	NT	209–217
TGTMGHFIL	A	0.5249	NA	NT	219–227
ALSVVTWNK	A	0.6979	NA	NT	239–247
KPGRRERMC	A	1.9453	NA	NT	98–106
GRRERMCMK	A	1.9807	NA	NT	100–108
CD4+ T cell Epitope (HTL)					
Peptide sequence	Antigenicity	Score	Allergenicity	Toxicity	Position
MCMKIENDCIFEVKH	A	1.7276	NA	NT	105–119
DRTLLSQQSGNVKIT	A	0.5502	NA	NT	502–516
TLLSQQSGNVKITVN	A	0.6306	NA	NT	504–518
B cell Epitope (BCL)					
Peptide sequence	Antigenicity	Score	Allergenicity	Toxicity	Position
GGRFTIPTGAGKPGDSGRPI	Antigenic	0.4508	NA	NT	198–217

∗A: Antigenic; ∗NA: Non-Antigenic; ∗NT: Non-Toxic.

**Table 5:** Antigenicity, Allergenicity, Toxicity and Immunogenicity analysis of the CD4+ T helper cell epitopes.

Peptide sequence	Antigenicity	Score	Allergenicity	Toxicity	IFN-ɣ	IL-4	IL-10
MCMKIENDCIFEVKH	A	1.7276	NA	NT	NEG	Inducer	Inducer
DRTLLSQQSGNVKIT	A	0.5502	NA	NT	POS	Inducer	Inducer

∗A: Antigenic; ∗NA: Non-Antigenic; ∗NT: Non-Toxic.

### 3.7 MHC-I and MHC-II binding allele prediction

The MHC-I and MHC-II binding prediction tool from the IEDB webserver predicted the corresponding MHC-I and MHC-II allele of the final 5 CD8+ (CTL) epitopes and final two HTL (CD4+) epitopes and shown in Tables 6 and 7 respectively. These alleles were then used for population coverage analysis against each of the T cell epitopes.

**Table 6:** Five shortlisted CD8+ T-cell epitopes MHC I binding predictions. Threshold: percentile rank <10.

Epitope	MHC- I Allele	Percentile Rank <10
KPGDSGRPI	HLA-B∗07:02 HLA-B∗51:01 HLA-B∗53:01, HLA-B∗08:01 HLA-B∗35:01	0.07 1.6 3.1 7.6 4.2
TGTMGHFIL	HLA-B∗08:01 HLA-B∗51:01 HLA-B∗07:02 HLA-B∗53:01 HLA-A∗24:02 HLA-A∗23:01 HLA-B∗35:01	4.2 6.2 6.9 7.6 7.7 8.3 9.1
ALSVVTWNK	HLA-A∗03:01 HLA-A∗11:01 HLA-A∗30:01 HLA-A∗31:01 HLA-A∗68:01 HLA-A∗32:01 HLA-A∗33:01 HLA-A∗30:02	0.1 0.11 1.1 1.5 2 2.6 4.8 5.8
KPGRRERMC	HLA-B∗07:02 HLA-B∗08:01	2.3 5.9
GRRERMCMK	HLA-A∗30:01 HLA-A∗03:01 HLA-A∗31:01	2.1 8 8.3

**Table 7:** Two shortlisted CD4+ T-cell epitopes MHC II binding predictions. Threshold: percentile rank <20.

Epitope	MHC- II Allele	Percentile Rank
MCMKIENDCIFEVKH	HLA-DRB3∗01:01 HLA-DRB1∗03:01	2 5.7
DRTLLSQQSGNVKIT	HLA-DRB4∗01:01 HLA-DRB5∗01:01 HLA-DRB1∗07:01 HLA-DRB3∗02:02	15 16 19 20

### 3.8 Population coverage of the predicted epitopes

**Figure 1:**
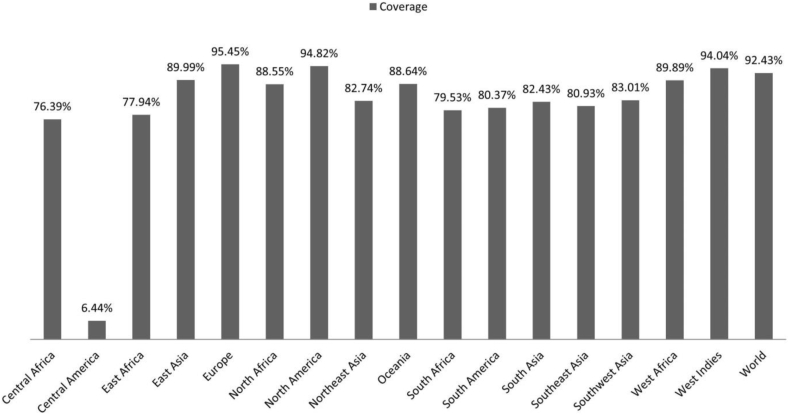
MHC I Combined population coverage of the CD8+ T cell epitopes. Highest population coverage: Europe; Lowest population coverage: Central America.

IEDB provided population coverage analysis tool was applied to predict the MHC I combined population coverage of the final five CD8+ T cell epitopes against a set of area around the world. However, the “HLA-DR” alleles in this server aren't updated for this analysis yet and that's why we skipped the MHC II population coverage analysis. The results showed the highest population coverage in the North America (94.82%) and lowest population coverage in the Central America (6.44%). Furthermore, Combination of the five epitopes can cover 92.43% of average world population predicted by the webserver (Figure 1).

### 3.9 Tertiary structure prediction, modification and validation

**Figure 2:**
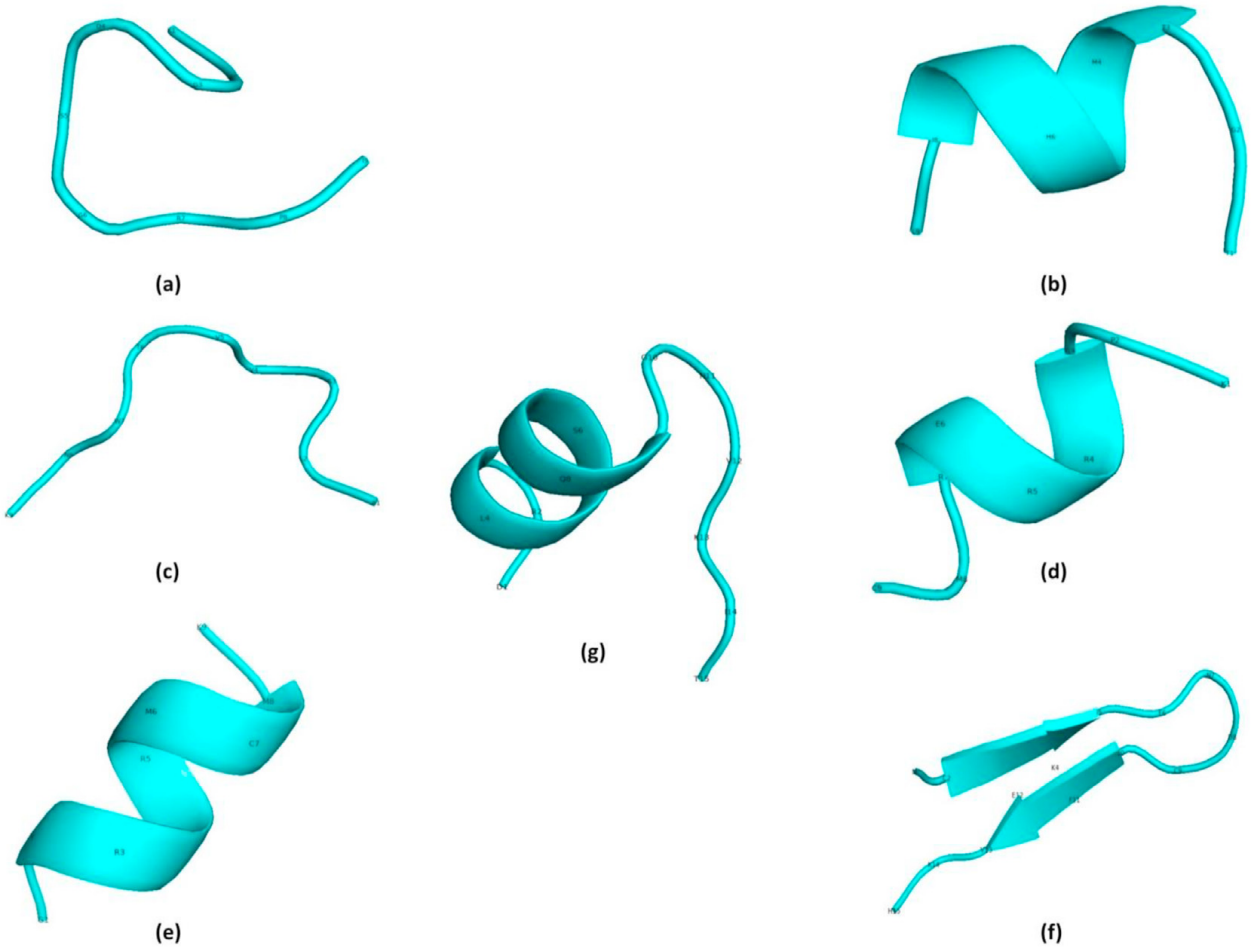
Tertiary structure representation of the final T cell epitopes (CD8+ and CD4+). a) Epitope ^209^KPGDSGRPI^217^; b) Epitope ^219^TGTMGHFIL^227^; c) Epitope ^239^ALSVVTWNK^247^; d) Epitope ^98^KPGRRERMC^106^; e) Epitope ^100^GRRERMCMK^108^; f) Epitope ^105^MCMKIENDCIFEVKH^119^ and g) Epitope ^504^TLLSQQSGNVKITVN^518^.

**Supplementary Figure 1:**
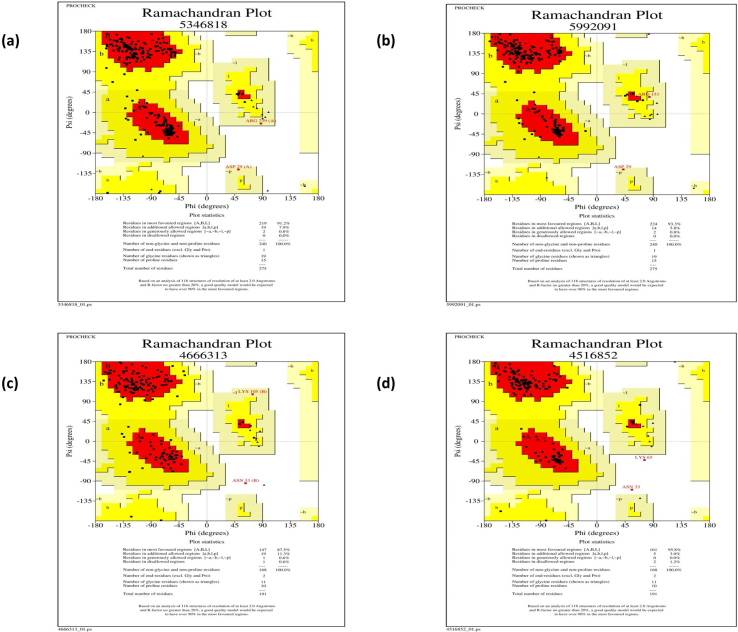
Ramachandran plot analysis of the HLA alleles before refinement and after refinement. a) HLA-B∗07:02 before refinement; b) HLA-B∗07:02 after refinement; c) HLA-DRB1∗15:01 before refinement and d) HLA-DRB1∗15:01 after refinement.

To predict the 3D structure of the final CTL and HTL epitopes, PEP-FOLD3 Peptide Structure Prediction server was used (Figure 2). Further, as mentioned in the method, we selected the HLA-B∗07:02 for binding interaction analysis with CTL epitopes and HLA-DRB1∗15:01 for binding interaction analysis with HTL epitopes. Then these alleles (HLA-B∗07:02 and HLA-DRB1∗15:01) were retrieved from the Protein Data Bank and refined by using Modrefiner prior to docking. To validate the refinement, Ramachandran plot analysis revealed that for HLA-B∗07:02, residues in the favored regions were increased by 2.1% after refinement. No residues were in the outlier region for HLA-B∗07:02 after refinement. Similarly for HLA-DRB1∗15:01, residues in the favored region were increased by 8.3% after refinement. We also notice 0.6% increments in the outlier region respectively for HLA-DRB1∗15:01 after refinement (Supplementary Figure 1).

### 3.10 Docking analysis of HLA-Epitope interaction

**Figure 3:**
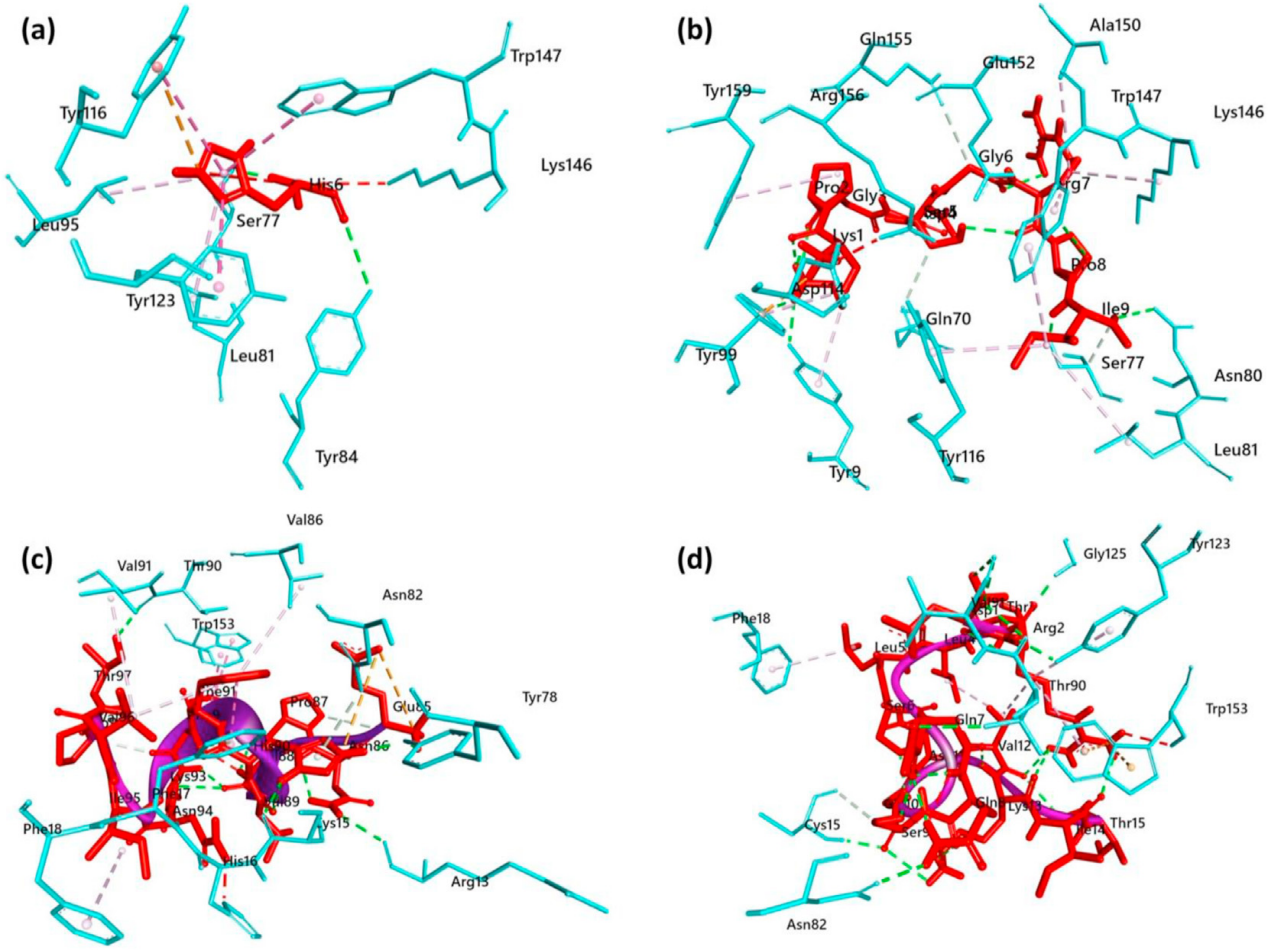
Molecular docking analysis of T cell epitopes with HLA alleles. a) Control Epitope docked with HLA-B∗07:02; b) Epitope ^209^KPGDSGRPI^217^ docked with HLA-B∗07:02; c) Control Epitope docked with HLA-DRB1∗15:01 and d) Epitope ^502^DRTLLSQQSGNVKIT^516^ docked with HLA-DRB1∗15:01. The interacting bonds are displayed as: Conventional hydrogen bonds/Carbon hydrogen bonds as green line, pi-pi stack/pi-alkyl as pink lines, Salt bridge/attractive charge/electrostatic as brown lines.

A structure based molecular docking was performed for analyzing the free binding energy and docking pose of each of the epitope-HLA molecule by utilizing AutoDock Vina in PyRx 0.8. For MHC-I-epitope interaction, as most of the epitope showed good binding affinity with HLA-B∗07:02 and ^209^KPGDSGRPI^217^ has the highest epitope score, we selected these epitope and HLA for MHC-I binding Molecular docking analysis. The control epitope used here is 13 amino acid long NY-ESO-1 peptide (“APRGPHGGAASGL”) to compare with our proposed CTL epitope. After epitope selection, The PDB files of each epitopes and refined 3D structure of the HLA were uploaded in the PyRx AutoDock Vina interface. To give the maximal space to the ligand for their liberal movement inside the search space which were fixed to 58.52Å in the X, 44.56Å in the Y and 71.36Å in the Z direction, so we made the grid box large enough during docking simulation. After docking simulation, the binding energy for epitope ^209^KPGDSGRPI^217^ to the binding groove of the HLA-B∗07:02 was found -6.8 kcal/mol. This binding energy represents good HLA-Epitope interaction in contrary experimental epitope which was -4.6 kcal/mol. Further, to examine for MHC-II binding interaction we used the epitope ^502^DRTLLSQQSGNVKIT^516^ as it showed maximum allele binding affinity (HLA-DRB1∗15:01, HLA-DRB1∗07:01, HLA-DRB5∗01:01, HLA-DRB3∗02:02). However, we finally subjected the HLA-DRB1∗15:01 for docking analysis as the readily available crystal structure of it's in the PDB database. The control epitope used here is 15 amino acid long myelin basic protein (“ENPVVHFFKNIVTPR”). The actual size of the grid box was slightly varied from the previous simulation and was fixed at 43.92Å, 76.37 Å and 55.25 Å in X, Y, and X axes correspondingly. After docking completion, the free binding energy of the epitope ^502^DRTLLSQQSGNVKIT^516^ and HLA-DRB1∗15:01 was found - 4.5 kcal/mol whereas the control epitope showed -5.6 kcal/mol with HLA-DRB1∗15:01. The interacting residues of the docked complexes of HLA-Epitope are shown in Figure 3 and Table 8.

**Table 8:** HLA-Epitope interaction (non-bond) analysis of T cell epitopes after docking.

Interacting Residue	Distance (Å)	Bond Types	Interacting Residue	Distance (Å)	Bond Types
**HLA-B∗07:02-Control Epitope**			**HLA-B∗07:02-** “**KPGDSGRPI”**		
TYR84	3.33967	H Bond	ASP114	2.98318	AC
SER77	1.84756	H Bond	ASN80	3.02273	H Bond
SER77	2.64115	H Bond	TRP147	3.20765	H Bond
TYR116	4.26229	Pi-Cation	TYR9	2.43215	H Bond
TYR123	5.69228	Pi-Pi Stacked	TYR99	1.97586	H Bond
TYR116	4.95875	Pi-Pi T-shaped	GLU152	2.04653	H Bond
TRP147	5.05543	Pi-Pi T-shaped	SER77	1.73811	H Bond
LEU81	5.28716	Pi-Alkyl	SER77	3.19062	CH Bond
LEU95	5.2538	Pi-Alkyl	GLN70	3.7926	CH Bond
			GLN155	3.58448	CH Bond
			TYR99	2.65845	Pi-Donor Hydrogen Bond
			LEU81	5.39445	Alkyl
			ALA150	4.61931	Alkyl
			LYS146	4.69667	Alkyl
			TYR9	4.8897	Pi-Alkyl
			TYR99	4.52285	Pi-Alkyl
			TYR116	5.09857	Pi-Alkyl
			TRP147	5.15463	Pi-Alkyl
			TRP147	4.84108	Pi-Alkyl
			TYR159	5.22647	Pi-Alkyl
HLA-DRB1∗15:01- Control Epitope			HLA-DRB1∗15:01-“DRTLLSQQSGNVKIT”		
HIS90	5.57759	AC	GLY125	2.55218	H Bond
TYR78	2.61271	H Bond	THR90	2.57846	H Bond
ARG13	2.59141	H Bond	VAL91	3.0038	H Bond
CYS15	2.36193	H Bond	VAL91	2.2295	H Bond
TYR78	1.96696	H Bond	TYR123	2.54355	H Bond
THR90	2.92635	H Bond	THR90	2.17327	H Bond
ASN82	3.88837	Pi-Donor Hydrogen Bond	ASN82	2.25545	H Bond
PHE17	5.18232	Pi-Pi Stacked	TRP153	4.73279	Alkyl
TRP153	4.57725	Pi-Pi Stacked	TRP153	4.25368	Alkyl
TRP153	3.69344	Pi-Pi Stacked	PHE18	5.1231	Pi-Alkyl
VAL91	5.37256	Alkyl	TYR123	5.40289	Pi-Alkyl
PHE18	4.78438	Pi-Alkyl			

∗AC: Attractive electrostatic Charge interaction; ∗H: Hydrogen bond; ∗CH: Carbon Hydrogen bond.

## 4 Discussion

From the last decades, more than 55 countries around the world have reported about the outbreak of reemerging CHIKV which caused human fatal chikungunya fever with muscle pain, joint pain, joint swelling, and rash [40]. Similar symptoms are also observed in the MAYV infection as both of the viruses share same genus (Alphaviruses) under the family Togaviridae. Thus, MAYV infection misdiagnosis is likely to occur because of cross-reactivity with other alphaviruses [41]. Recently, in Brazil the Mayaro virus leading infections were misinterpreted with Chikungunya viral infection from 2014 to 2016 [10]. From a recent study, it was established that, Chikungunya viral infection can also create defensive effects against Mayaro viral infection [42]. Therefore, a particular vaccine potent to work against both of the viruses could be a very viable option to minimize this issue.

However, several attempts based on designing of innovative subunit vaccine against CHIKV had failed, in the contrary, numerous epitope based vaccine candidates against particular microorganism are showing very promising in preclinical studies [43]. For example, epitope based vaccine candidates which are currently under the evaluation of Phase I and phase II clinical trials (29 and 13 respectively) against human immune deficiency virus (HIV), hepatitis B virus (HBV), hepatitis C virus (HCV) and cytomegalovirus [44]. No peptide based vaccine is available against CHIKV in clinical trial. In fact, no approved vaccines are available for CHIKV infection rather than a live attenuated vaccine which is in the phase II trial [15]. Furthermore, no vaccinology strategies followed till now for MAYV infection. Besides, the recent advancement in the *in silco* peptide vaccine designing with reverse vaccinology approach is getting popular day by day because of its low threats of re-activation as like as live attenuated vaccine. The first epitope based vaccine was designed against *N. meningitides* using bioinformatics and after that many of the vaccines are designed for different infections including Dengue virus [45], Nipah virus [46], Ebola virus [47], Herpes simplex virus [48], human norovirus [49] and so on.

Considering all the facts above, this study was conducted to identify the common epitopes between the structural polyproteins of CHIKV and MAYV which could assure a good protection against both of the viruses as vaccine candidates. The structural polyproteins were targeted in this study since both CHIKV/MAYV structural polyprotein plays a crucial role in viral attachment, penetration and subsequent fusion with host plasma membrane to form endosome within human cells. Theoretically, any vaccine substance must have two types of antigenic or immunogenic binding sites namely B cell and T cell epitopes to grow immunity in an individual [50]. B cell lymphoma (BCL) of a vaccine triggers B cells to produce antibodies (also their isotypes) and memory cells, as well as CTL can distinguish and destroy infected host cells and thus protect the adjacent healthy cells [51]. In this study, we have emphasized on identifying probable B cell and cytotoxic T lymphocyte epitopes from the structural proteins of both CHIKV and MAYV that can confer immunity of the host. Initially the most antigenic structural polyprotein of CHIKV was identified and used as a referral protein for epitope prediction. Then, we BLAST these initially predicted epitopes (Both B cell and T cell epitope) against MAYV structural polyprotein and sort out the 100% conserved epitopes. This result 100% conserved homologous epitopes for both of the viruses. Further, we studied the cross reaction possibilities of these epitopes against Dengue Virus (Type- 1, 2, 3 and 4) and Zika Virus's polyprotein. None of these epitopes found as cross reactive against these arboviruses. However, to confirm the safety and efficacy of the predicted peptide vaccine we vigorously screened these epitopes through various computational analysis e.g. immunogenicity, toxicity, allergenicity, and antigenicity. Thus we shortlisted the initially predicted epitopes and predicted 5 CTL (^209^KPGDSGRPI^217^, ^219^TGTMGHFIL^227^, ^239^ALSVVTWNK^247^, ^98^KPGRRERMC^106^ and ^100^GRRERMCMK^108^) out of 245, 2 HTL (^105^MCMKIENDCIFEVKH^119^ and ^502^DRTLLSQQSGNVKIT^516^) out of 306 and 1 BCL (^504^GGRFTIPTGAGKPGDSGRPI^518^) out of 22 as the most potential candidates. Furthermore, population coverage analysis of our 5 potential CTL epitopes showed outstanding average population coverage (92.43%) around the world where the standard world population coverage was at least 40% [33]. Lastly, docking analysis was conducted between the CTL and HTL with corresponding HLA alleles (class I and class II) to find out the relative binding affinity and protein-protein interaction between the ligands and receptors. Molecular docking simulation of our proposed HLA-Epitope assured good interaction compared to control. Thus by analyzing the binding potential it's again confirmed that, peptide found in our study is good immunogenic as compared to the naturally bound peptide used as control epitope in our study.

One limitation of our approach is that the length of the epitope itself. Generally, the length of an epitope isn't sufficient enough to induce immunization in vivo. Besides, though epitope based vaccine is safer than conventional vaccine (e.g. live attenuated vaccine); there is a possibility of enzymatic degradation of the epitopes in vivo. So, the peptides found in our study needs to be structurally or physically modified prior to use as a vaccine candidate. This could be done by using different strategies like prodrug approach, peptidomimetic method, analogue developments, hydrophobic ion coupling, adjuvant linking, and joining with fatty acids. By applying these approaches predicted peptides physiochemical stability can be enhanced. Of note, researchers have been working to eliminate these viruses by understanding theirs pathophysiology and transmission. We hope that, our predicted epitopes may be evaluated for further in vivo research and clinical assay to develop a potential vaccine which can eliminate these neglected tropical diseases beyond any doubt.

## 5 Conclusion

This study discovered viable epitope candidates for designing an epitope-based peptide vaccine against Chikungunya and Mayaro virus concomitantly. Furthermore, this study found the predicted prominent epitopes won't have any cross reacting activity with other arboviruses e.g. Dengue and Zika virus. However, though epitope based vaccine has several limitations regarding the size of the epitopes, we anticipated that, after further wet lab based experiments, our predicted epitopes may develop secure, reproducible and genetically stable vaccine that could induce an effective immune response in future.

## Declarations

### Author contribution statement

Hammadul Hoque: Conceived and designed the experiments.

Srijon Ghosh, Rahatul Islam: Performed the experiments; Wrote the paper.

Mashiur Rahaman, Nurnabi Azad Jewel: Analyzed and interpreted the data.

Abunasar Miah: Contributed reagents, materials, analysis tools or data.

### Funding statement

This research did not receive any specific grant from funding agencies in the public, commercial, or not-for-profit sectors.

### Data availability statement

Data included in article/supplementary material/referenced in article.

### Declaration of interests statement

The authors declare no conflict of interest.

### Additional information

No additional information is available for this paper.

## References

[1] (15 september 2020). “Chikungunya Fact sheet” WHO.

[2] Long K.C. (2011). Experimental transmission of Mayaro virus by Aedes aegypti.

[3] Smith G., Francy D. (1991). Laboratory studies of a Brazilian strain of Aedes albopictus as a potential vector of Mayaro and Oropouche viruses. J. Am. Mosquito Control Assoc..

[4] Brustolin M. (2018). Anopheles mosquitoes may drive invasion and transmission of Mayaro virus across geographically diverse regions.

[5] Halsey E.S. (2013). Mayaro virus infection, Amazon basin region, Peru, 2010–2013.

[6] Powers A.M. (2006). Genetic relationships among Mayaro and Una viruses suggest distinct patterns of transmission.

[7] Seppa N., Hirshfeld J.J.S.N. (2015). Chikungunya is on the move.

[8] (23 October 2015). Number of Cumulative Cases 2013–2014.

[9] Auguste A.J. (2015). Evolutionary and ecological characterization of Mayaro virus strains isolated during an outbreak, Venezuela, 2010.

[10] Esposito D.L.A., Fonseca B.A.L. (2017). Will Mayaro virus be responsible for the next outbreak of an arthropod-borne virus in Brazil?. Braz. J. Infect. Dis..

[11] Lednicky J. (2016). Mayaro virus in child with acute febrile illness, Haiti, 2015.

[12] Solignat M. (2009). Replication cycle of chikungunya: a re-emerging arbovirus.

[13] Lavergne A. (2006). Mayaro virus: complete nucleotide sequence and phylogenetic relationships with other alphaviruses.

[14] Powers A.M. (2000). Re-emergence of Chikungunya and O’nyong-nyong viruses: evidence for distinct geographical lineages and distant evolutionary relationships.

[15] Edelman R. (2000). Phase II safety and immunogenicity study of live chikungunya virus vaccine TSI-GSD-218.

[16] Partidos C.D. (2012). Cross-protective immunity against o ‘nyong-nyong virus afforded by a novel recombinant chikungunya vaccine.

[17] Doytchinova I.A., Flower D.R. (2007). VaxiJen: a server for prediction of protective antigens, tumour antigens and subunit vaccines. BMC Bioinf..

[18] Wilkins M.R. (1999). Protein identification and analysis tools in the ExPASy server. Methods Mol. Biol. (Clifton, N.J.).

[19] Larsen M.V. (2007). Large-scale validation of methods for cytotoxic T-lymphocyte epitope prediction. BMC Bioinf..

[20] Paul S. (2015). Development and validation of a broad scheme for prediction of HLA class II restricted. T Cell Epitopes.

[21] Nielsen M., Lundegaard C., Lund O. (2007). Prediction of MHC class II binding affinity using SMM-align, a novel stabilization matrix alignment method. BMC Bioinformatics.

[22] Chen J. (2007). Prediction of linear B-cell epitopes using amino acid pair antigenicity scale.

[23] Potocnakova L., Bhide M., Pulzova L.B. (2016). An Introduction to B-Cell Epitope Mapping and in Silico Epitope Prediction. J. Immunol. Res..

[24] Bui H.-H. (2007). Development of an epitope conservancy analysis tool to facilitate the design of epitope-based diagnostics and vaccines. BMC Bioinf..

[25] Dimitrov I. (2014). AllergenFP: allergenicity prediction by descriptor fingerprints.

[26] Gupta S. (2013). In silico approach for predicting toxicity of peptides and proteins. PloS One.

[27] Luckheeram R.V. (2012). CD4+ T Cells: Differentiation and Functions.

[28] Dhanda S.K., Vir P., Raghava G.P. (2013). Designing of interferon-gamma inducing MHC class-II binders. Biol. Direct.

[29] Dhanda S.K. (2013). Prediction of IL4 Inducing Peptides.

[30] Nagpal G. (2017). Computer-aided designing of immunosuppressive peptides based on IL-10 inducing potential.

[31] Trolle T. (2015). Automated benchmarking of peptide-MHC class I binding predictions. Bioinformatics.

[32] Reynisson B. (2020). NetMHCpan-4.1 and NetMHCIIpan-4.0: Improved Predictions of MHC Antigen Presentation by Concurrent Motif Deconvolution and Integration of MS MHC Eluted Ligand Data.

[33] Bui H.-H. (2006). Predicting population coverage of T-cell epitope-based diagnostics and vaccines. BMC Bioinf..

[34] Lamiable A. (2016). PEP-FOLD3: faster de novo structure prediction for linear peptides in solution and in complex. Nucleic Acids Res..

[35] ul Qamar M.T. (2014). Potential of plant alkaloids as dengue ns3 protease inhibitors: molecular docking and simulation approach.

[36] Ul Qamar M.T. (2014). Molecular docking based screening of plant flavonoids as dengue NS1 inhibitors.

[37] Xu D., Zhang Y. (2011). Improving the physical realism and structural accuracy of protein models by a two-step atomic-level energy minimization. Biophys. J..

[38] Laskowski R.A. (1993). PROCHECK: a program to check the stereochemical quality of protein structures.

[39] Dallakyan S., Olson A.J. (2015). Small-molecule library screening by docking with PyRx. Methods Mol. Biol..

[40] (6 April 2016). Chikungunya Virus Symptoms, Diagnosis, & Treatment.

[41] Smith J.L. (2018). Human antibody responses to emerging mayaro virus and cocirculating alphavirus infections examined by using structural proteins from nine new and old world lineages.

[42] Webb E.M. (2019). Effects of Chikungunya virus immunity on Mayaro virus disease and epidemic potential.

[43] Li W. (2014). Peptide vaccine: progress and challenges. Vaccines.

[44] Reginald K. (2018). Development of peptide vaccines in dengue. Curr. Pharmaceut. Des..

[45] Islam R., Parvez M.S., Anwar S., Hosen M.J. (2020). Delineating blueprint of an epitope-based peptide vaccine against the multiple serovars of dengue virus: a hierarchical reverse vaccinology approach. Inf. Med. Unlocked.

[46] Ravichandran L., Venkatesan A., Febin Prabhu Dass J. (2019). Epitope-based immunoinformatics approach on RNA-dependent RNA polymerase (RdRp) protein complex of Nipah virus (NiV). J. Cell. Biochem..

[47] Khan M. (2015). Epitope-based peptide vaccine design and target site depiction against Ebola viruses: an immunoinformatics study.

[48] Hasan M. (2020). Contriving a chimeric polyvalent vaccine to prevent infections caused by herpes simplex virus (type-1 and type-2): an exploratory immunoinformatic approach.

[49] Azim K.F. (2019). Immunoinformatics approaches for designing a novel multi epitope peptide vaccine against human norovirus (Norwalk virus).

[50] Tambunan U.S.F. (2016).

[51] Garcia K.C., Teyton L., Wilson I.A. (1999). Structural basis of T cell recognition. Annu. Rev. Immunol..

