# Supplementary material for: Implementation of in silico methods to predict common epitopes for vaccine development against Chikungunya and Mayaro viruses
Source: Heliyon. 2021 Mar 8;7(3):e06396. doi: 10.1016/j.heliyon.2021.e06396 (PMC7944042; doi:10.1016/j.heliyon.2021.e06396)
Supplement: Supplementary File 1 [file mmc1.docx]

**Supplementary File 1:** Dataset of the sequences used in the study

**Chikungunya Virus (Frameshifted Structural Polyprotein- 838 AA)**

>UniprotKB ID: **P0DOK1 (Ref_Seq)**

MEFIPTQTFYNRRYQPRPWTPRPTIQVIRPRPRPQRQAGQLAQLISAVNKLTMRAVPQQK

PRKNRKNKKQKQKQQAPQNNTNQKKQPPKKKPAQKKKKPGRRERMCMKIENDCIFEVKHE

GKVTGYACLVGDKVMKPAHVKGTIDNADLAKLAFKRSSKYDLECAQIPVHMKSDASKFTH

EKPEGYYNWHHGAVQYSGGRFTIPTGAGKPGDSGRPIFDNKGRVVAIVLGGANEGARTAL

SVVTWNKDIVTKITPEGAEEWSLAIPVMCLLANTTFPCSQPPCIPCCYEKEPEETLRMLE

DNVMRPGYYQLLQASLTCSPHRQRRSTKDNFNVYKATRPYLAHCPDCGEGHSCHSPVALE

RIRNEATDGTLKIQVSLQIGIGTDDSHDWTKLRYMDNHIPADAGRAGLFVRTSAPCTITG

TMGHFILARCPKGETLTVGFTDSRKISHSCTHPFHHDPPVIGREKFHSRPQHGKELPCST

YVQSNAATAEEIEVHMPPDTPDRTLLSQQSGNVKITVNSQTVRYKCNCGGSNEGLITTDK

VINNCKVDQCHAAVTNHKKWQYNSPLVPRNAELGDRKGKIHIPFPLANVTCMVPKARNPT

VTYGKNQVIMLLYPDHPTLLSYRSMGEEPNYQEEWVTHKKEVVLTVPTEGLEVTWGNNEP

YKYWPQLSANGTAHGHPHEIILYYYELYPTMTVVVVSVASFILLSMVGMAVGMCMCARRR

CITPYELTPGATVPFLLSLICCIRTAKAATYQEAAVYLWNEQQPLFWLQALIPLAALIVL

CNCLRLLPCCCKTLAFLSRNEHRCPHCERVRTRNSDPEHGGSTV

**Mayaro Virus (Structural Polyprotein- 1263 AA)**

>UniprotKB ID: **Q8QZ72 (Ref_Seq)**

MDFLPTQVFYGRRWRPRMPPRPWRPRMPTMQRPDQQARQMQQLIAAVSTLALRQNAAAPQ

RGKKKQPRRKKPKPQPEKPKKQEQKPKQKKAPKRKPGRRERMCMKIEHDCIFEVKHEGKV

TGYACLVGDKVMKPAHVPGVIDNADLARLSYKKSSKYDLECAQIPVAMKSDASKYTHEKP

EGHYNWHYGAVQYTGGRFTVPTGVGKPGDSGRPIFDNKGPVVAIVLGGANEGTRTALSVV

TWNKDMVTKITPEGTVEWAASTVTAMCLLTNISFPCFQPSCAPCCYEKGPEPTLRMLEEN

VNSEGYYDLLHAAVYCRNSSRSKRSTANHFNAYKLTRPYVAYCADCGMGHSCHSPAMIEN

IQADATDGTLKIQFASQIGLTKTDTHDHTKIRYAEGHDIAEAARSTLKVHSSSECTVTGT

MGHFILAKCPPGERISVSFVDSKNEHRTCRIAYHHEQRLIGRERFTVRPHHGIELPCTTY

QLTTAETSEEIDMHMPPDIPDRTILSQQSGNVKITVNGRTVRYSSSCGSQAVGTTTTDKT

INSCTVDKCQAYVTSHTKWQFNSPFVPRRMQAERKGKVHIPFPLINTTCRVPLAPEALVR

SGKREATLSLHPIHPTLLSYRTFGAERVFDEQWITAQTEVTIPVPVEGVEYQWGNHKPQR

FVVALTTEGKAHGWPHEIIEYYYGLHPTTTIVVVIRVSVVVLLSFAASVYMCVVARTKCL

TPYALTPGAVVPVTIGVLCCAPKAHAASFAEGMAYLWDNNQSMFWMELTGPLALLILATC

CARSLLSCCKGSFLVAMSIGSAVASAYEHTAIIPNQVGFPYKAHVAREGYSPLTLQMQVI

ETSLEPTLNLEYITCDYKTKVPSPYVKCCGTAECRTQDKPEYKCAVFTGVYPFMWGGAYC

FCDSENTQMSEAYVERADVCKHDHAAAYRAHTASLRAKIKVTYGTVNQTVEAYVNGDHAV

TIAGTKFIFGPVSTPWTPFDTKILVYKGELYNQDFPRYGAGQPGRFGDIQSRTLDSRDLY

ANTGLKLARPAAGNIHVPYTQTPSGFKTWQKDRDSPLNAKAPFGCIIQTNPVRAMNCAVG

NIPVSMDIADSAFTRLTDAPVISELTCTVSTCTHSSDFGGIAVLSYKVEKSGRCDIHSHS

NVAVLQEVSIETEGRSVIHFSTASASPSFVVSVCSSRATCTAKCEPPKDHVVTYPANHNG

VTLPDLSSTAMTWAQHLAGGVGLLIALAVLILVIVTCVTLRR

**Dengue Virus-Type 1 (Polyprotein- - 3449 AA)**

>NCBI accession no: **NP_059433.1 (Ref_Seq)**

MNNQRKKTGRPSFNMLKRARNRVSTVSQLAKRFSKGLLSGQGPMKLVMAFIAFLRFLAIP

PTAGILARWGSFKKNGAIKVLRGFKKEISNMLNIMNRRKRSVTMLLMLLPTALAFHLTTR

GGEPHMIVSKQERGKSLLFKTSAGVNMCTLIAMDLGELCEDTMTYKCPRITETEPDDVDC

WCNATETWVTYGTCSQTGEHRRDKRSVALAPHVGLGLETRTETWMSSEGAWKQIQKVETW

ALRHPGFTVIALFLAHAIGTSITQKGIIFILLMLVTPSMAMRCVGIGNRDFVEGLSGATW

VDVVLEHGSCVTTMAKDKPTLDIELLKTEVTNPAVLRKLCIEAKISNTTTDSRCPTQGEA

TLVEEQDTNFVCRRTFVDRGWGNGCGLFGKGSLITCAKFKCVTKLEGKIVQYENLKYSVI

VTVHTGDQHQVGNETTEHGTTATITPQAPTSEIQLTDYGALTLDCSPRTGLDFNEMVLLT

MEKKSWLVHKQWFLDLPLPWTSGASTSQETWNRQDLLVTFKTAHAKKQEVVVLGSQEGAM

HTALTGATEIQTSGTTTIFAGHLKCRLKMDKLTLKGMSYVMCTGSFKLEKEVAETQHGTV

LVQVKYEGTDAPCKIPFSSQDEKGVTQNGRLITANPIVTDKEKPVNIEAEPPFGESYIVV

GAGEKALKLSWFKKGSSIGKMFEATARGARRMAILGDTAWDFGSIGGVFTSVGKLIHQIF

GTAYGVLFSGVSWTMKIGIGILLTWLGLNSRSTSLSMTCIAVGMVTLYLGVMVQADSGCV

INWKGRELKCGSGIFVTNEVHTWTEQYKFQADSPKRLSAAIGKAWEEGVCGIRSATRLEN

IMWKQISNELNHILLENDMKFTVVVGDVSGILAQGKKMIRPQPMEHKYSWKSWGKAKIIG

ADVQNTTFIIDGPNTPECPDNQRAWNIWEVEDYGFGIFTTNIWLKLRDSYTQVCDHRLMS

AAIKDSKAVHADMGYWIESEKNETWKLARASFIEVKTCIWPKSHTLWSNGVLESEMIIPK

IYGGPISQHNYRPGYFTQTAGPWHLGKLELDFDLCEGTTVVVDEHCGNRGPSLRTTTVTG

KTIHEWCCRSCTLPPLRFKGEDGCWYGMEIRPVKEKEENLVKSMVSAGSGEVDSFSLGLL

CISIMIEEVMRSRWSRKMLMTGTLAVFLLLTMGQLTWNDLIRLCIMVGANASDKMGMGTT

YLALMATFRMRPMFAVGLLFRRLTSREVLLLTVGLSLVASVELPNSLEELGDGLAMGIMM

LKLLTDFQSHQLWATLLSLTFVKTTFSLHYAWKTMAMILSIVSLFPLCLSTTSQKTTWLP

VLLGSLGCKPLTMFLITENKIWGRKSWPLNEGIMAVGIVSILLSSLLKNDVPLAGPLIAG

GMLIACYVISGSSADLSLEKAAEVSWEEEAEHSGASHNILVEVQDDGTMKIKDEERDDTL

TILLKATLLAISGVYPMSIPATLFVWYFWQKKKQRSGVLWDTPSPPEVERAVLDDGIYRI

LQRGLLGRSQVGVGVFQEGVFHTMWHVTRGAVLMYQGKRLEPSWASVKKDLISYGGGWRF

QGSWNAGEEVQVIAVEPGKNPKNVQTAPGTFKTPEGEVGAIALDFKPGTSGSPIVNREGK

IVGLYGNGVVTTSGTYVSAIAQAKASQEGPLPEIEDEVFRKRNLTIMDLHPGSGKTRRYL

PAIVREAIRRNVRTLVLAPTRVVASEMAEALKGMPIRYQTTAVKSEHTGKEIVDLMCHAT

FTMRLLSPVRVPNYNMIIMDEAHFTDPASIAARGYISTRVGMGEAAAIFMTATPPGSVEA

FPQSNAVIQDEERDIPERSWNSGYDWITDFPGKTVWFVPSIKSGNDIANCLRKNGKRVVQ

LSRKTFDTEYQKTKNNDWDYVVTTDISEMGANFRADRVIDPRRCLKPVILKDGPERVILA

GPMPVTVASAAQRRGRIGRNQNKEGDQYIYMGQPLNNDEDHAHWTEAKMLLDNINTPEGI

IPALFEPEREKSAAIDGEYRLRGEARKTFVELMRRGDLPVWLSYKVASEGFQYSDRRWCF

DGERNNQVLEENMDVEIWTKEGERKKLRPRWLDARTYSDPLALREFKEFAAGRRSVSGDL

ILEIGKLPQHLTQRAQNALDNLVMLHNSEQGGKAYRHAMEELPDTIETLMLLALIAVLTG

GVTLFFLSGRGLGKTSIGLLCVIASSALLWMASVEPHWIAASIILEFFLMVLLIPEPDRQ

RTPQDNQLAYVVIGLLFMILTAAANEMGLLETTKKDLGIGHAAAENHHHAAMLDVDLHPA

SAWTLYAVATTIITPMMRHTIENTTANISLTAIANQAAILMGLDKGWPISKMDIGVPLLA

LGCYSQVNPLTLTAAVFMLVAHYAIIGPGLQAKATREAQKRTAAGIMKNPTVDGIVAIDL

DPVVYDAKFEKQLGQIMLLILCTSQILLMRTTWALCESITLATGPLTTLWEGSPGKFWNT

TIAVSMANIFRGSYLAGAGLAFSLMKSLGGGRRGTGAQGETLGEKWKRQLNQLSKSEFNT

YKRSGIIEVDRSEAKEGLKRGEPTKHAVSRGTAKLRWFVERNLVKPEGKVIDLGCGRGGW

SYYCAGLKKVTEVKGYTKGGPGHEEPIPMATYGWNLVKLYSGKDVFFTPPEKCDTLLCDI

GESSPNPTIEEGRTLRVLKMVEPWLRGNQFCIKILNPYMPSVVETLEQMQRKHGGMLVRN

PLSRNSTHEMYWVSCGTGNIVSAVNMTSRMLLNRFTMAHRKPTYERDVDLGAGTRHVAVE

PEVANLDIIGQRIENIKNGHKSTWHYDEDNPYKTWAYHGSYEVKPSGSASSMVNGVVRLL

TKPWDVIPMVTQIAMTDTTPFGQQRVFKEKVDTRTPKAKRGTAQIMEVTARWLWGFLSRN

KKPRICTREEFTRKVRSNAAIGAVFVDENQWNSAKEAVEDERFWDLVHRERELHKQGKCA

TCVYNMMGKREKKLGEFGKAKGSRAIWYMWLGARFLEFEALGFMNEDHWFSRENSLSGVE

GEGLHKLGYILRDISKIPGGNMYADDTAGWDTRITEDDLQNEAKITDIMEPEHALLATSI

FKLTYQNKVVRVQRPAKNGTVMDVISRRDQRGSGQVGTYGLNTFTNMEAQLIRQMESEGI

FSPSELETPNLAERVLDWLKKHGTERLKRMAISGDDCVVKPIDDRFATALTALNDMGKVR

KDIPQWEPSKGWNDWQQVPFCSHHFHQLIMKDGREIVVPCRNQDELVGRARVSQGAGWSL

RETACLGKSYAQMWQLMYFHRRDLRLAANAICSAVPVDWVPTSRTTWSIHAHHQWMTTED

MLSVWNRVWIEENPWMEDKTHVSSWEDVPYLGKREDRWCGSLIGLTARATWATNIQVAIN

QVRRLIGNENYLDFMTSMKRFKNESDPEGALW

**Dengue Virus-Type 2 (Polyprotein- 3448 AA)**

> NCBI accession no: **NP_056776.2** **(Ref_Seq)**

MNNQRKKAKNTPFNMLKRERNRVSTVQQLTKRFSLGMLQGRGPLKLFMALVAFLRFLTIP

PTAGILKRWGTIKKSKAINVLRGFRKEIGRMLNILNRRRRSAGMIIMLIPTVMAFHLTTR

NGEPHMIVSRQEKGKSLLFKTEDGVNMCTLMAMDLGELCEDTITYKCPLLRQNEPEDIDC

WCNSTSTWVTYGTCTTMGEHRREKRSVALVPHVGMGLETRTETWMSSEGAWKHVQRIETW

ILRHPGFTMMAAILAYTIGTTHFQRALIFILLTAVTPSMTMRCIGMSNRDFVEGVSGGSW

VDIVLEHGSCVTTMAKNKPTLDFELIKTEAKQPATLRKYCIEAKLTNTTTESRCPTQGEP

SLNEEQDKRFVCKHSMVDRGWGNGCGLFGKGGIVTCAMFRCKKNMEGKVVQPENLEYTIV

ITPHSGEEHAVGNDTGKHGKEIKITPQSSITEAELTGYGTVTMECSPRTGLDFNEMVLLQ

MENKAWLVHRQWFLDLPLPWLPGADTQGSNWIQKETLVTFKNPHAKKQDVVVLGSQEGAM

HTALTGATEIQMSSGNLLFTGHLKCRLRMDKLQLKGMSYSMCTGKFKVVKEIAETQHGTI

VIRVQYEGDGSPCKIPFEIMDLEKRHVLGRLITVNPIVTEKDSPVNIEAEPPFGDSYIII

GVEPGQLKLNWFKKGSSIGQMFETTMRGAKRMAILGDTAWDFGSLGGVFTSIGKALHQVF

GAIYGAAFSGVSWTMKILIGVIITWIGMNSRSTSLSVTLVLVGIVTLYLGVMVQADSGCV

VSWKNKELKCGSGIFITDNVHTWTEQYKFQPESPSKLASAIQKAHEEGICGIRSVTRLEN

LMWKQITPELNHILSENEVKLTIMTGDIKGIMQAGKRSLRPQPTELKYSWKTWGKAKMLS

TESHNQTFLIDGPETAECPNTNRAWNSLEVEDYGFGVFTTNIWLKLKEKQDVFCDSKLMS

AAIKDNRAVHADMGYWIESALNDTWKIEKASFIEVKNCHWPKSHTLWSNGVLESEMIIPK

NLAGPVSQHNYRPGYHTQITGPWHLGKLEMDFDFCDGTTVVVTEDCGNRGPSLRTTTASG

KLITEWCCRSCTLPPLRYRGEDGCWYGMEIRPLKEKEENLVNSLVTAGHGQVDNFSLGVL

GMALFLEEMLRTRVGTKHAILLVAVSFVTLITGNMSFRDLGRVMVMVGATMTDDIGMGVT

YLALLAAFKVRPTFAAGLLLRKLTSKELMMTTIGIVLLSQSTIPETILELTDALALGMMV

LKMVRNMEKYQLAVTIMAILCVPNAVILQNAWKVSCTILAVVSVSPLLLTSSQQKTDWIP

LALTIKGLNPTAIFLTTLSRTSKKRSWPLNEAIMAVGMVSILASSLLKNDIPMTGPLVAG

GLLTVCYVLTGRSADLELERAADVKWEDQAEISGSSPILSITISEDGSMSIKNEEEEQTL

TILIRTGLLVISGLFPVSIPITAAAWYLWEVKKQRAGVLWDVPSPPPMGKAELEDGAYRI

KQKGILGYSQIGAGVYKEGTFHTMWHVTRGAVLMHKGKRIEPSWADVKKDLISYGGGWKL

EGEWKEGEEVQVLALEPGKNPRAVQTKPGLFKTNAGTIGAVSLDFSPGTSGSPIIDKKGK

VVGLYGNGVVTRSGAYVSAIAQTEKSIEDNPEIEDDIFRKRRLTIMDLHPGAGKTKRYLP

AIVREAIKRGLRTLILAPTRVVAAEMEEALRGLPIRYQTPAIRAEHTGREIVDLMCHATF

TMRLLSPVRVPNYNLIIMDEAHFTDPASIAARGYISTRVEMGEAAGIFMTATPPGSRDPF

PQSNAPIIDEEREIPERSWNSGHEWVTDFKGKTVWFVPSIKAGNDIAACLRKNGKKVIQL

SRKTFDSEYVKTRTNDWDFVVTTDISEMGANFKAERVIDPRRCMKPVILTDGEERVILAG

PMPVTHSSAAQRRGRIGRNPKNENDQYIYMGEPLENDEDCAHWKEAKMLLDNINTPEGII

PSMFEPEREKVDAIDGEYRLRGEARKTFVDLMRRGDLPVWLAYRVAAEGINYADRRWCFD

GVKNNQILEENVEVEIWTKEGERKKLKPRWLDARIYSDPLALKEFKEFAAGRKSLTLNLI

TEMGRLPTFMTQKARDALDNLAVLHTAEAGGRAYNHALSELPETLETLLLLTLLATVTGG

IFLFLMSGRGIGKMTLGMCCIITASILLWYAQIQPHWIAASIILEFFLIVLLIPEPEKQR

TPQDNQLTYVVIAILTVVAATMANEMGFLEKTKKDLGLGSIATQQPESNILDIDLRPASA

WTLYAVATTFVTPMLRHSIENSSVNVSLTAIANQATVLMGLGKGWPLSKMDIGVPLLAIG

CYSQVNPITLTAALFLLVAHYAIIGPGLQAKATREAQKRAAAGIMKNPTVDGITVIDLDP

IPYDPKFEKQLGQVMLLVLCVTQVLMMRTTWALCEALTLATGPISTLWEGNPGRFWNTTI

AVSMANIFRGSYLAGAGLLFSIMKNTTNTRRGTGNIGETLGEKWKSRLNALGKSEFQIYK

KSGIQEVDRTLAKEGIKRGETDHHAVSRGSAKLRWFVERNMVTPEGKVVDLGCGRGGWSY

YCGGLKNVREVKGLTKGGPGHEEPIPMSTYGWNLVRLQSGVDVFFIPPEKCDTLLCDIGE

SSPNPTVEAGRTLRVLNLVENWLNNNTQFCIKVLNPYMPSVIEKMEALQRKYGGALVRNP

LSRNSTHEMYWVSNASGNIVSSVNMISRMLINRFTMRYKKATYEPDVDLGSGTRNIGIES

EIPNLDIIGKRIEKIKQEHETSWHYDQDHPYKTWAYHGSYETKQTGSASSMVNGVVRLLT

KPWDVVPMVTQMAMTDTTPFGQQRVFKEKVDTRTQEPKEGTKKLMKITAEWLWKELGKKK

TPRMCTREEFTRKVRSNAALGAIFTDENKWKSAREAVEDSRFWELVDKERNLHLEGKCET

CVYNMMGKREKKLGEFGKAKGSRAIWYMWLGARFLEFEALGFLNEDHWFSRENSLSGVEG

EGLHKLGYILRDVSKKEGGAMYADDTAGWDTRITLEDLKNEEMVTNHMEGEHKKLAEAIF

KLTYQNKVVRVQRPTPRGTVMDIISRRDQRGSGQVGTYGLNTFTNMEAQLIRQMEGEGVF

KSIQHLTITEEIAVQNWLARVGRERLSRMAISGDDCVVKPLDDRFASALTALNDMGKIRK

DIQQWEPSRGWNDWTQVPFCSHHFHELIMKDGRVLVVPCRNQDELIGRARISQGAGWSLR

ETACLGKSYAQMWSLMYFHRRDLRLAANAICSAVPSHWVPTSRTTWSIHAKHEWMTTEDM

LTVWNRVWIQENPWMEDKTPVESWEEIPYLGKREDQWCGSLIGLTSRATWAKNIQAAINQ

VRSLIGNEEYTDYMPSMKRFRREEEEAGVLW

**Dengue Virus-Type 3 (Polyprotein- 3447 AA)**

> NCBI accession no: **YP_001621843.1** **(Ref_Seq)**

MNNQRKKTGKPSINMLKRVRNRVSTGSQLAKRFSKGLLNGQGPMKLVMAFIAFLRFLAIP

PTAGVLARWGTFKKSGAIKVLKGFKKEISNMLSIINQRKKTSLCLMMILPAALAFHLTSR

DGEPRMIVGKNERGKSLLFKTASGINMCTLIAMDLGEMCDDTVTYKCPHITEVEPEDIDC

WCNLTSTWVTYGTCNQAGEHRRDKRSVALAPHVGMGLDTRTQTWMSAEGAWRQVEKVETW

ALRHPGFTILALFLAHYIGTSLTQKVVIFILLMLVTPSMTMRCVGVGNRDFVEGLSGATW

VDVVLEHGGCVTTMAKNKPTLDIELQKTEATQLATLRKLCIEGKITNITTDSRCPTQGEA

VLPEEQDQNYVCKHTYVDRGWGNGCGLFGKGSLVTCAKFQCLEPIEGKVVQYENLKYTVI

ITVHTGDQHQVGNETQGVTAEITPQASTTEAILPEYGTLGLECSPRTGLDFNEMILLTMK

NKAWMVHRQWFFDLPLPWASGATTETPTWNRKELLVTFKNAHAKKQEVVVLGSQEGAMHT

ALTGATEIQNSGGTSIFAGHLKCRLKMDKLELKGMSYAMCTNTFVLKKEVSETQHGTILI

KVEYKGEDAPCKIPFSTEDGQGKAHNGRLITANPVVTKKEEPVNIEAEPPFGESNIVIGI

GDNALKINWYKKGSSIGKMFEATERGARRMAILGDTAWDFGSVGGVLNSLGKMVHQIFGS

AYTALFSGVSWVMKIGIGVLLTWIGLNSKNTSMSFSCIAIGIITLYLGAVVQADMGCVIN

WKGKELKCGSGIFVTNEVHTWTEQYKFQADSPKRLATAIAGAWENGVCGIRSTTRMENLL

WKQIANELNYILWENNIKLTVVVGDTLGVLEQGKRTLTPQPMELKYSWKTWGKAKIVTAE

TQNSSFIIDGPNTPECPSASRAWNVWEVEDYGFGVFTTNIWLKLREVYTQLCDHRLMSAA

VKDERAVHADMGYWIESQKNGSWKLEKASLIEVKTCTWPKSHTLWTNGVLESDMIIPKSL

AGPISQHNYRPGYHTQTAGPWHLGKLELDFNYCEGTTVVITESCGTRGPSLRTTTVSGKL

IHEWCCRSCTLPPLRYMGEDGCWYGMEIRPISEKEENMVKSLVSAGSGKVDNFTMGVLCL

AILFEEVLRGKFGKKHMIAGVFFTFVLLLSGQITWRDMAHTLIMIGSNASDRMGMGVTYL

ALIATFKIQPFLALGFFLRKLTSRENLLLGVGLAMATTLQLPEDIEQMANGVALGLMALK

LITQFETYQLWTALVSLTCSNTIFTLTVAWRTATLILAGVSLLPVCQSSSMRKTDWLPMT

VAAMGVPPLPLFIFSLKDTLKRRSWPLNEGVMAVGLVSILASSLLRNDVPMAGPLVAGGL

LIACYVITGTSADLTVEKAPDVTWEEEAEQTGVSHNLMITVDDDGTMRIKDDETENILTV

LLKTALLIVSGIFPYSIPATLLVWHTWQKQTQRSGVLWDVPSPPETQKAELEEGVYRIKQ

QGIFGKTQVGVGVQKEGVFHTMWHVTRGAVLTHNGKRLEPNWASVKKDLISYGGGWRLSA

QWQKGEEVQVIAVEPGKNPKNFQTTPGTFQTTTGEIGAIALDFKPGTSGSPIINREGKVV

GLYGNGVVTKNGGYVSGIAQTNAEPDGPTPELEEEMFKKRNLTIMDLHPGSGKTRKYLPA

IVREAIKRRLRTLILAPTRVVAAEMEEALKGLPIRYQTTATKSEHTGREIVDLMCHATFT

MRLLSPVRVPNYNLIIMDEAHFTDPASIAARGYISTRVGMGEAAAIFMTATPPGTADAFP

QSNAPIQDEERDIPERSWNSGNEWITDFAGKTVWFVPSIKAGNDIANCLRKNGKKVIQLS

RKTFDTEYQKTKLNDWDFVVTTDISEMGANFKADRVIDPRRCLKPVILTDGPERVILAGP

MPVTAASAAQRRGRVGRNPQKENDQYIFTGQPLNNDEDHAHWTEAKMLLDNINTPEGIIP

ALFEPEREKSAAIDGEYRLKGESRKTFVELMRRGDLPVWLAHKVASEGIKYTDRKWCFDG

QRNNQILEENMDVEIWTKEGEKKKLRPRWLDARTYSDPLALKEFKDFAAGRKSIALDLVT

EIGRVPSHLAHRTRNALDNLVMLHTSEDGGRAYRHAVEELPETMETLLLLGLMILLTGGA

MLFLISGKGIGKTSIGLICVIASSGMLWMAEVPLQWIASAIVLEFFMMVLLIPEPEKQRT

PQDNQLAYVVIGILTLAATIAANEMGLLETTKRDLGMSKEPGVVSPTSYLDVDLHPASAW

TLYAVATTVITPMLRHTIENSTANVSLAAIANQAVVLMGLDKGWPISKMDLGVPLLALGC

YSQVNPLTLTAAVLLLITHYAIIGPGLQAKATREAQKRTAAGIMKNPTVDGIMTIDLDSV

IFDSKFEKQLGQVMLLVLCAVQLLLMRTSWALCEALTLATGPITTLWEGSPGKFWNTTIA

VSMANIFRGSYLAGAGLAFSIMKSVGTGKRGTGSQGETLGEKWKKKLNQLSRKEFDLYKK

SGITEVDRTEAKEGLKRGETTHHAVSRGSAKLQWFVERNMVVPEGRVIDLGCGRGGWSYY

CAGLKKVTEVRGYTKGGPGHEEPVPMSTYGWNIVKLMSGKDVFYLPPEKCDTLLCDIGES

SPSPTVEESRTIRVLKMVEPWLKNNQFCIKVLNPYMPTVIEHLERLQRKHGGMLVRNPLS

RNSTHEMYWISNGTGNIVSSVNMVSRLLLNRFTMTHRRPTIEKDVDLGAGTRHVNAEPET

PNMDVIGERIKRIKEEHNSTWHYDDENPYKTWAYHGSYEVKATGSASSMINGVVKLLTKP

WDVVPMVTQMAMTDTTPFGQQRVFKEKVDTRTPRPMPGTRKAMEITAEWLWRTLGRNKRP

RLCTREEFTKKVRTNAAMGAVFTEENQWDSAKAAVEDEEFWKLVDRERELHKLGKCGSCV

YNMMGKREKKLGEFGKAKGSRAIWYMWLGARYLEFEALGFLNEDHWFSRENSYSGVEGEG

LHKLGYILRDISKIPGGAMYADDTAGWDTRITEDDLHNEEKIIQQMDPEHRQLANAIFKL

TYQNKVVKVQRPTPTGTVMDIISRKDQRGSGQLGTYGLNTFTNMEAQLVRQMEGEGVLTK

ADLENPHLLEKKITQWLETKGVERLKRMAISGDDCVVKPIDDRFANALLALNDMGKVRKD

IPQWQPSKGWHDWQQVPFCSHHFHELIMKDGRKLVVPCRPQDELIGRARISQGAGWSLRE

TACLGKAYAQMWSLMYFHRRDLRLASNAICSAVPVHWVPTSRTTWSIHAHHQWMTTEDML

TVWNRVWIEENPWMEDKTPVTTWENVPYLGKREDQWCGSLIGLTSRATWAQNIPTAIQQV

RSLIGNEEFLDYMPSMKRFRKEEESEGAIW

**Dengue Virus-Type 4 (Polyprotein- 3444 AA)**

> NCBI accession no: **NP_073286.1** **(Ref_Seq)**

MNQRKKVVRPPFNMLKRERNRVSTPQGLVKRFSTGLFSGKGPLRMVLAFITFLRVLSIPP

TAGILKRWGQLKKNKAIKILIGFRKEIGRMLNILNGRKRSTITLLCLIPTVMAFSLSTRD

GEPLMIVAKHERGRPLLFKTTEGINKCTLIAMDLGEMCEDTVTYKCPLLVNTEPEDIDCW

CNLTSTWVMYGTCTQSGERRREKRSVALTPHSGMGLETRAETWMSSEGAWKHAQRVESWI

LRNPGFALLAGFMAYMIGQTGIQRTVFFVLMMLVAPSYGMRCVGVGNRDFVEGVSGGAWV

DLVLEHGGCVTTMAQGKPTLDFELTKTTAKEVALLRTYCIEASISNITTATRCPTQGEPY

LKEEQDQQYICRRDVVDRGWGNGCGLFGKGGVVTCAKFSCSGKITGNLVQIENLEYTVVV

TVHNGDTHAVGNDTSNHGVTAMITPRSPSVEVKLPDYGELTLDCEPRSGIDFNEMILMKM

KKKTWLVHKQWFLDLPLPWTAGADTSEVHWNYKERMVTFKVPHAKRQDVTVLGSQEGAMH

SALAGATEVDSGDGNHMFAGHLKCKVRMEKLRIKGMSYTMCSGKFSIDKEMAETQHGTTV

VKVKYEGAGAPCKVPIEIRDVNKEKVVGRIISSTPLAENTNSVTNIELEPPFGDSYIVIG

VGNSALTLHWFRKGSSIGKMFESTYRGAKRMAILGETAWDFGSVGGLFTSLGKAVHQVFG

SVYTTMFGGVSWMIRILIGFLVLWIGTNSRNTSMAMTCIAVGGITLFLGFTVQADMGCVA

SWSGKELKCGSGIFVVDNVHTWTEQYKFQPESPARLASAILNAHKDGVCGIRSTTRLENV

MWKQITNELNYVLWEGGHDLTVVAGDVKGVLTKGKRALTPPVSDLKYSWKTWGKAKIFTP

EARNSTFLIDGPDTSECPNERRAWNSLEVEDYGFGMFTTNIWMKFREGSSEVCDHRLMSA

AIKDQKAVHADMGYWIESSKNQTWQIEKASLIEVKTCLWPKTHTLWSNGVLESQMLIPKS

YAGPFSQHNYRQGYATQTVGPWHLGKLEIDFGECPGTTVTIQEDCDHRGPSLRTTTASGK

LVTQWCCRSCTMPPLRFLGEDGCWYGMEIRPLSEKEENMVKSQVTAGQGTSETFSMGLLC

LTLFVEECLRRRVTRKHMILVVVITLCAIILGGLTWMDLLRALIMLGDTMSGRIGGQIHL

AIMAVFKMSPGYVLGVFLRKLTSRETALMVIGMAMTTVLSIPHDLMELIDGISLGLILLK

IVTQFDNTQVGTLALSLTFIRSTMPLVMAWRTIMAVLFVVTLIPLCRTSCLQKQSHWVEI

TALILGAQALPVYLMTLMKGASRRSWPLNEGIMAVGLVSLLGSALLKNDVPLAGPMVAGG

LLLAAYVMSGSSADLSLEKAANVQWDEMADITGSSPIIEVKQDEDGSFSIRDVEETNMIT

LLVKLALITVSGLYPLAIPVTMTLWYMWQVKTQRSGALWDVPSPAATKKAALSEGVYRIM

QRGLFGKTQVGVGIHMEGVFHTMWHVTRGSVICHETGRLEPSWADVRNDMISYGGGWRLG

DKWDKEEDVQVLAIEPGKNPKHVQTKPGLFKTLTGEIGAVTLDFKPGTSGSPIINRKGKV

IGLYGNGVVTKSGDYVSAITQAERIGEPDYEVDEDIFRKKRLTIMDLHPGAGKTKRILPS

IVREALKRRLRTLILAPTRVVAAEMEEALRGLPIRYQTPAVKSEHTGREIVDLMCHATFT

TRLLSSTRVPNYNLIVMDEAHFTDPSSVAARGYISTRVEMGEAAAIFMTATPPGATDPFP

QSNSPIEDIEREIPERSWNTGFDWITDYQGKTVWFVPSIKAGNDIANCLRKSGKKVIQLS

RKTFDTEYPKTKLTDWDFVVTTDISEMGANFRAGRVIDPRRCLKPVILPDGPERVILAGP

IPVTPASAAQRRGRIGRNPAQEDDQYVFSGDPLKNDEDHAHWTEAKMLLDNIYTPEGIIP

TLFGPEREKTQAIDGEFRLRGEQRKTFVELMRRGDLPVWLSYKVASAGISYKDREWCFTG

ERNNQILEENMEVEIWTREGEKKKLRPRWLDARVYADPMALKDFKEFASGRKSITLDILT

EIASLPTYLSSRAKLALDNIVMLHTTERGGRAYQHALNELPESLETLMLVALLGAMTAGI

FLFFMQGKGIGKLSMGLITIAVASGLLWVAEIQPQWIAASIILEFFLMVLLIPEPEKQRT

PQDNQLIYVILTILTIIGLIAANEMGLIEKTKTDFGFYQVKTETTILDVDLRPASAWTLY

AVATTILTPMLRHTIENTSANLSLAAIANQAAVLMGLGKGWPLHRMDLGVPLLAMGCYSQ

VNPTTLTASLVMLLVHYAIIGPGLQAKATREAQKRTAAGIMKNPTVDGITVIDLEPISYD

PKFEKQLGQVMLLVLCAGQLLLMRTTWAFCEVLTLATGPILTLWEGNPGRFWNTTIAVST

ANIFRGSYLAGAGLAFSLIKNAQTPRRGTGTTGETLGEKWKRQLNSLDRKEFEEYKRSGI

LEVDRTEAKSALKDGSKIKHAVSRGSSKIRWIVERGMVKPKGKVVDLGCGRGGWSYYMAT

LKNVTEVKGYTKGGPGHEEPIPMATYGWNLVKLHSGVDVFYKPTEQVDTLLCDIGESSSN

PTIEEGRTLRVLKMVEPWLSSKPEFCIKVLNPYMPTVIEELEKLQRKHGGNLVRCPLSRN

STHEMYWVSGASGNIVSSVNTTSKMLLNRFTTRHRKPTYEKDVDLGAGTRSVSTETEKPD

MTIIGRRLQRLQEEHKETWHYDQENPYRTWAYHGSYEAPSTGSASSMVNGVVKLLTKPWD

VIPMVTQLAMTDTTPFGQQRVFKEKVDTRTPQPKPGTRMVMTTTANWLWALLGKKKNPRL

CTREEFISKVRSNAAIGAVFQEEQGWTSASEAVNDSRFWELVDKERALHQEGKCESCVYN

MMGKREKKLGEFGRAKGSRAIWYMWLGARFLEFEALGFLNEDHWFGRENSWSGVEGEGLH

RLGYILEEIDKKDGDLMYADDTAGWDTRITEDDLQNEELITEQMAPHHKILAKAIFKLTY

QNKVVKVLRPTPRGAVMDIISRKDQRGSGQVGTYGLNTFTNMEVQLIRQMEAEGVITQDD

MQNPKGLKERVEKWLKECGVDRLKRMAISGDDCVVKPLDERFGTSLLFLNDMGKVRKDIP

QWEPSKGWKNWQEVPFCSHHFHKIFMKDGRSLVVPCRNQDELIGRARISQGAGWSLRETA

CLGKAYAQMWSLMYFHRRDLRLASMAICSAVPTEWFPTSRTTWSIHAHHQWMTTEDMLKV

WNRVWIEDNPNMTDKTPVHSWEDIPYLGKREDLWCGSLIGLSSRATWAKNIHTAITQVRN

LIGKEEYVDYMPVMKRYSAPSESEGVL

**Zika Virus (Polyprotein- 3476 AA)**

> NCBI accession no: **YP_002790881.1** **(Ref_Seq)**

MKNPKEEIRRIRIVNMLKRGVARVNPLGGLKRLPAGLLLGHGPIRMVLAILAFLRFTAIK

PSLGLINRWGSVGKKEAMEIIKKFKKDLAAMLRIINARKERKRRGADTSIGIIGLLLTTA

MAAEITRRGSAYYMYLDRSDAGKAISFATTLGVNKCHVQIMDLGHMCDATMSYECPMLDE

GVEPDDVDCWCNTTSTWVVYGTCHHKKGEARRSRRAVTLPSHSTRKLQTRSQTWLESREY

TKHLIKVENWIFRNPGFALVAVAIAWLLGSSTSQKVIYLVMILLIAPAYSIRCIGVSNRD

FVEGMSGGTWVDVVLEHGGCVTVMAQDKPTVDIELVTTTVSNMAEVRSYCYEASISDMAS

DSRCPTQGEAYLDKQSDTQYVCKRTLVDRGWGNGCGLFGKGSLVTCAKFTCSKKMTGKSI

QPENLEYRIMLSVHGSQHSGMIGYETDEDRAKVEVTPNSPRAEATLGGFGSLGLDCEPRT

GLDFSDLYYLTMNNKHWLVHKEWFHDIPLPWHAGADTGTPHWNNKEALVEFKDAHAKRQT

VVVLGSQEGAVHTALAGALEAEMDGAKGRLFSGHLKCRLKMDKLRLKGVSYSLCTAAFTF

TKVPAETLHGTVTVEVQYAGTDGPCKIPVQMAVDMQTLTPVGRLITANPVITESTENSKM

MLELDPPFGDSYIVIGVGDKKITHHWHRSGSTIGKAFEATVRGAKRMAVLGDTAWDFGSV

GGVFNSLGKGIHQIFGAAFKSLFGGMSWFSQILIGTLLVWLGLNTKNGSISLTCLALGGV

MIFLSTAVSADVGCSVDFSKKETRCGTGVFIYNDVEAWRDRYKYHPDSPRRLAAAVKQAW

EEGICGISSVSRMENIMWKSVEGELNAILEENGVQLTVVVGSVKNPMWRGPQRLPVPVNE

LPHGWKAWGKSYFVRAAKTNNSFVVDGDTLKECPLEHRAWNSFLVEDHGFGVFHTSVWLK

VREDYSLECDPAVIGTAVKGREAAHSDLGYWIESEKNDTWRLKRAHLIEMKTCEWPKSHT

LWTDGVEESDLIIPKSLAGPLSHHNTREGYRTQVKGPWHSEELEIRFEECPGTKVYVEET

CGTRGPSLRSTTASGRVIEEWCCRECTMPPLSFRAKDGCWYGMEIRPRKEPESNLVRSMV

TAGSTDHMDHFSLGVLVILLMVQEGLKKRMTTKIIMSTSMAVLVVMILGGFSMSDLAKLV

ILMGATFAEMNTGGDVAHLALVAAFKVRPALLVSFIFRANWTPRESMLLALASCLLQTAI

SALEGDLMVLINGFALAWLAIRAMAVPRTDNIALPILAALTPLARGTLLVAWRAGLATCG

GIMLLSLKGKGSVKKNLPFVMALGLTAVRVVDPINVVGLLLLTRSGKRSWPPSEVLTAVG

LICALAGGFAKADIEMAGPMAAVGLLIVSYVVSGKSVDMYIERAGDITWEKDAEVTGNSP

RLDVALDESGDFSLVEEDGPPMREIILKVVLMAICGMNPIAIPFAAGAWYVYVKTGKRSG

ALWDVPAPKEVKKGETTDGVYRVMTRRLLGSTQVGVGVMQEGVFHTMWHVTKGAALRSGE

GRLDPYWGDVKQDLVSYCGPWKLDAAWDGLSEVQLLAVPPGERARNIQTLPGIFKTKDGD

IGAVALDYPAGTSGSPILDKCGRVIGLYGNGVVIKNGSYVSAITQGKREEETPVECFEPS

MLKKKQLTVLDLHPGAGKTRRVLPEIVREAIKKRLRTVILAPTRVVAAEMEEALRGLPVR

YMTTAVNVTHSGTEIVDLMCHATFTSRLLQPIRVPNYNLNIMDEAHFTDPSSIAARGYIS

TRVEMGEAAAIFMTATPPGTRDAFPDSNSPIMDTEVEVPERAWSSGFDWVTDHSGKTVWF

VPSVRNGNEIAACLTKAGKRVIQLSRKTFETEFQKTKNQEWDFVITTDISEMGANFKADR

VIDSRRCLKPVILDGERVILAGPMPVTHASAAQRRGRIGRNPNKPGDEYMYGGGCAETDE

GHAHWLEARMLLDNIYLQDGLIASLYRPEADKVAAIEGEFKLRTEQRKTFVELMKRGDLP

VWLAYQVASAGITYTDRRWCFDGTTNNTIMEDSVPAEVWTKYGEKRVLKPRWMDARVCSD

HAALKSFKEFAAGKRGAALGVMEALGTLPGHMTERFQEAIDNLAVLMRAETGSRPYKAAA

AQLPETLETIMLLGLLGTVSLGIFFVLMRNKGIGKMGFGMVTLGASAWLMWLSEIEPARI

ACVLIVVFLLLVVLIPEPEKQRSPQDNQMAIIIMVAVGLLGLITANELGWLERTKNDIAH

LMGRREEGATMGFSMDIDLRPASAWAIYAALTTLITPAVQHAVTTSYNNYSLMAMATQAG

VLFGMGKGMPFMHGDLGVPLLMMGCYSQLTPLTLIVAIILLVAHYMYLIPGLQAAAARAA

QKRTAAGIMKNPVVDGIVVTDIDTMTIDPQVEKKMGQVLLIAVAISSAVLLRTAWGWGEA

GALITAATSTLWEGSPNKYWNSSTATSLCNIFRGSYLAGASLIYTVTRNAGLVKRRGGGT

GETLGEKWKARLNQMSALEFYSYKKSGITEVCREEARRALKDGVATGGHAVSRGSAKIRW

LEERGYLQPYGKVVDLGCGRGGWSYYAATIRKVQEVRGYTKGGPGHEEPMLVQSYGWNIV

RLKSGVDVFHMAAEPCDTLLCDIGESSSSPEVEETRTLRVLSMVGDWLEKRPGAFCIKVL

CPYTSTMMETMERLQRRHGGGLVRVPLCRNSTHEMYWVSGAKSNIIKSVSTTSQLLLGRM

DGPRRPVKYEEDVNLGSGTRAVASCAEAPNMKIIGRRIERIRNEHAETWFLDENHPYRTW

AYHGSYEAPTQGSASSLVNGVVRLLSKPWDVVTGVTGIAMTDTTPYGQQRVFKEKVDTRV

PDPQEGTRQVMNIVSSWLWKELGKRKRPRVCTKEEFINKVRSNAALGAIFEEEKEWKTAV

EAVNDPRFWALVDREREHHLRGECHSCVYNMMGKREKKQGEFGKAKGSRAIWYMWLGARF

LEFEALGFLNEDHWMGRENSGGGVEGLGLQRLGYILEEMNRAPGGKMYADDTAGWDTRIS

KFDLENEALITNQMEEGHRTLALAVIKYTYQNKVVKVLRPAEGGKTVMDIISRQDQRGSG

QVVTYALNTFTNLVVQLIRNMEAEEVLEMQDLWLLRKPEKVTRWLQSNGWDRLKRMAVSG

DDCVVKPIDDRFAHALRFLNDMGKVRKDTQEWKPSTGWSNWEEVPFCSHHFNKLYLKDGR

SIVVPCRHQDELIGRARVSPGAGWSIRETACLAKSYAQMWQLLYFHRRDLRLMANAICSA

VPVDWVPTGRTTWSIHGKGEWMTTEDMLMVWNRVWIEENDHMEDKTPVTKWTDIPYLGKR

EDLWCGSLIGHRPRTTWAENIKDTVNMVRRIIGDEEKYMDYLSTQVRYLGEEGSTPGVL

**All the representative sequences of CHIKV from all variants/genotypes**

>ANH22457.1:1-824 truncated polyprotein [Chikungunya virus]MEFIPTQTFYNRRYQPRPWTPRPTIQVIRPRPRPQRQAGQLAQLISAVNKLTMRAVPQQKPRRNRKNKKQKQKQQAPQNNTNQKKQPPKKKPAQKKKKPGRRERMCMKIENDCIFEVKHEGKVTGYACLVGDKVMKPAHVKGTIDNADLAKLAFKRSSKYDLECAQIPVHMKSDASKFTHEKPEGYYNWHHGAVQYSGGRFTIPTGAGKPGDSGRPIFDNKGRVVAIVLGGANEGARTALSVVTWNKDIVTKITPEGAEEWSLAIPVMCLLANTTFPCSQPPCIPCCYEKEPEETLRMLEDNVMRPGYYQLLQASLTCSPHRQRRSTKDNFNVYKATRPYLAHCPDCGEGHSCHSPVALERIRNEATDGTLKIQVSLQIGIGTDDSHDWTKLRYMDNHIPADAGRAGLFVRTSAPCTITGTMGHFILARCPKGETLTVGFTDSRKISHSCTHPFHHDPPVIGREKFHSRPQHGKELPCSTYVQSNAATAEEIEVHMPPDTPDRTLLSQQSGNVKITVNGRTVRYKCNCGGSNEGLITTDKVINNCKVDQCHAAVTNHKKWQYNSPLVPRNAELGDRKGKIHIPFPLANVTCMVPKARNPTVTYGKNQVIMLLYPDHPTLLSYRSMGEEPNYQEEWVTHKKEVVLTVPTEGLEVTWGNNEPYKYWPQLSANGTAHGHPHEIILYYYELYPTMTVVVVSVASFILLSMVGMAVGMCMCARRRCITPYELTPGATVPFLLSLICCIRTAKAATYQEAAVYLWNEQQPLFWLQALIPLAALIVLCNCLRLLPCCCKTLAFLAVMSHRCPHCERVRTRNSDPEHGGSTV

>AYI50330.1:1-824 truncated structural polyprotein [Chikungunya virus]MEFIPTQTFYNRRYQPRPWTPRSTIQIIRPRPRPQRQAGQLAQLISAVNKLTMRAVPQQKPRRNRKNKKQKQKQQAPQNNTNQKKQPPKKKPAQKKKKPGRRERMCMKIENDCIFEVKHEGKVTGYACLVGDKVMKPAHVKGTIDNADLAKLAFKRSSKYDLECAQIPVHMKSDASKFTHEKPEGYYNWHHGAVQYSGGRFTIPTGAGKPGDSGRPIFDNKGRVVAIVLGGANEGARTALSVVTWNKDIVTKITPEGAEEWSLAIPVMCLLANTTFPCSQPPCTPCCYEKEPEETLRMLEDNVMRPGYYQLLQASLTCSPHRQRRSTKDNFNVYKATRPYLAHCPDCGEGHSCHSPVALERIRNEATDGTLKIQVSLQIGIKTDDSHDWTKLRYMDNHMPADAERAGLFVRTSAPCTITGTMGHFILARCPKGETLTVGFTDSRKISHSCTHPFHHDPPVIGREKFHSRPQHGKELPCSTYVQSTAATTEEIEVHMPPDTPDRTLMSQQSGNVKITVNGQTVRYKCNCGGSNEGLTTTDKVINNCKVDQCHAAVTNHKKWQYNSPLVPRNAELGDRKGKIHIPFPLANVTCRVPKARNPTVTYGKNQVIMLLYPDHPTLLSYRNMGEEPNYQEEWVMHKKEVVLTVPTEGLEVTWGNNEPYKYWPQLSTNGTAHGHPHEIILYYYELYPTMTVVVVSVATFILLSMVGMAVGMCMCARRRCITPYELTPGATVPFLLSLICCIRTAKAATYQEAAIYLWNEQQPLFWLQALIPLAALIVLCNCLRLLPCCCKTLAFLSRNERRCPHCERVRTRNSDPEHGGSTV

>AYI50324.1:1-824 truncated structural polyprotein [Chikungunya virus]MEFIPTQTFYNRRYQPRPWTPRPTIQVIRPRPRPQRQAGQLAQLISAVNKLTMRAVPQQKPRRNRKNKKQKQKQQAPQNNTNQKKQPPKKKPAQKKKKPGRRERMCMKIENDCIFEVKHEGKVTGYACLVGDKVMKPAHVKGTIDNADLAKLAFKRSSKYDLECAQIPVHMKSDASKFTHEKPEGYYNWHHGAVQYSGGRFTIPTGAGKPGDSGRPIFDNKGRVVAIVLGGANEGARTALSVVTWNKDIVTKITPEGAEEWSLAIPVMCLLANTTFPCSQPPCTPCCYEKEPEETLRMLEDNVMRPGYYQLLQASLTCSPHRQRRSTKDNFNVYKATRPYLAHCPDCGEGHSCHSPVALERIRNEATDGTLKIQVSLQIGIKTDDSHDWTKLRYMDNHMPADAERAGLFVRTSAPCTITGTMGHFILARCPKGETLTVGFTDSRKISHSCTHPFHHDPPVIGREKFHSRPQHGKELPCSTYVQSTAATTEEIEVHMPPDTPDRTLMSQQSGNVKITVNGQTVRYKCNCGGSNEGLTTTDKVINNCKVDQCHAAVTNHKKWQYNSPLVPRNAELGDRKGKIHIPFPLANVTCRVPKARNPTVTYGKNQVIMLLYPDHPTLLSYRNMGEEPHYQEEWVMHKKEVVLTVPTEGLEVTWGNNEPYKYWPQLSTNGTAHGHPHEIILYYYELYPTMTVVVVSVATFILLSMVGMAAGMCMCARRRCITPYELTPGATVPFLLSLICCIRTAKAATYQEAAIYLWNEQQPLFWLQALIPLAALIVLCNCLRLLPCCCKTLAFLSRNERRCPHCERVRTRNSDPEHGGSTV

>AQX78117.1:1-824 truncated polyprotein [Chikungunya virus]MEFIPTQTFYNRRYQPRPWTPRPTIQVIRPRPRPQRQAGQLAQLISAVNKLTMRAVPQQKPRRNRKNKKQKQKQQAPQNNTNQKKQPPKKKPAQKKKKPGRRERMCMKIENDCIFEVKHEGKVTGYACLVGDKVMKPAHVKGTIDNADLAKLAFKRSSKYDLECAQIPVHMKSDASKFTHEKPEGYYNWHHGAVQYSGGRFTIPTGAGKPGDSGRPIFDNKGRVVAIVLGGANEGARTALSVVTWNKDIVTKITPEGAEEWSLAIPVMCLLANTTFPCSQPPCTPCCYEKEPEETLRMLEDNVMRPGYYQLLQASLTCSPHRQRRSTKDNFNVYKATRPYLAHCPDCGEGHSCHSPVALERIRNEATDGTLKIQVSLQIGIKTDDSHDWTKLRYMDNHMPADAERAGLFVRTSAPCTITGTMGHFILARCPKGETLTVGFTDSRKISHSCTHPFHHDPPVIGREKFHSRPQHGKELPCSTYVQSTAATTEEIEVHMPPDTPDRTLMSQQSGNVKITVNGQTVRYKCNCGGSNEGLTTTDKVINNCKVDQCHAAVTNHKKWQYNSPLVPRNAELGDRKGKIHIPFPLANVTCRVPKARNPTVTYGKNQVIMLLYPDHPTLLSYRNMGEEPNYQEEWVMHKKEVVLTVPTEGLEVTWGNNEPYKYWPQLSTNGTAHGHPHEIILYYYELYPTMTVVVVSVATFILLSMVGMAAGMCMCARRRCITPYELTPGATVPFLLSLICCIRTAKAATYQEAAIYLWNEQQPLFWLQALIPLAALIVLCNCLRLLPCCCKTLAFLAVMSRRCPHCERVRTRNSDPEHGGSTV

>AQX78126.1:1-824 truncated polyprotein [Chikungunya virus]MEFIPTQTFYNRRYQPRPWTPRPTIQVIRPRPRPQRQAGQLAQLISAVNKLTMRAVPQQKPRRNRKNKKQKQKQQAPQNNTNQKKQPPKKKPAQKKKKPGRRERMCMKIENDCIFEVKHEGKVTGYACLVGDKVMKPAHVKGTIDNADLAKLAFKRSSKYDLECAQIPVHMKSDASKFTHEKPEGYYNWHHGAVQYSGGRFTIPTGAGKPGDSGRPIFDNKGRVVAIVLGGANEGARTALSVVTWNKDIVTKITPEGAEEWSLAIPVMCLLANTTFPCSQPPCTPCCYEKEPEETLRMLEDNVMRPGYYQLLQASLTCSPHRQRRSTKDNFNVYKATRPYLAHCPDCGEGHSCHSPVALERIRNEATDGTLKIQVSLQIGIKTDDSHDWTKLRYMDNHMPADAERAGLFVRTSAPCMITGTMGHFILARCPKGETLTVGFTDSRKISHSCTHPFHHDPPVIGREKFHSRPQHGRELPCSTYVQSTAATTEEIEVHMPPDTPDRTLMSQQSGNVKITVNGQTVRYKCNCGGSNEGLITTDKVINNCKIDQCHAAVTNHKKWQYNSPLVPRNAELGDRKGKIHIPFPLANVTCRVPKARNPTVTYGKNQVIMLLYPDHPTLLSYRNMGEEPNYQEEWVTHKKEVVLTVPTEGLEVTWGNNEPYKYWPQLSTNGTAHGHPHEIILYYYELYPTMTVVVVSVASFVLLSMVGVAVGMCMCARRRCITPYELTPGATVPFLLSLICCIRTAKAATYQEAAVYLWNEQQPLFWLQALIPLAALIVLCNCLRLLPCCCKTLAFLAVMSRRCPHCERVRTRNSDPEHGGSTV

>AQX78114.1:1-824 truncated polyprotein [Chikungunya virus]MEFIPTQTFYNRRYQPRPWTPRSTIQVIRPRPRPQRQAGQLAQLISAVNKLTMRAVPQQKPRRNRKNKKQKQKQQAPQNNTNQKKQPPKKKPAQKKKKPGRRERMCMKIENDCIFEVKHEGKVTGYACLVGDKVMKPAHVKGTIDNADLAKLAFKRSSKYDLECAQIPVHMKSDASKFTHEKPEGYYNWHHGAVQYSGGRFTIPTGAGKPGDSGRPIFDNKGRVVAIVLGGANEGARTALSVVTWNKDIVTKITPEGAEEWSLAIPVMCLLANTTFPCSQPPCTPCCYEKEPEETLRMLEDNVMRPGYYQLLQASLTCSPHRQRRSTKDNFNVYKATRPYLAHCPDCGEGHSCHSPVALERIRNEATDGTLKIQVSLQIGIKTDDSHDWTKLRYMDNHMPADAERAGLFVRTSAPCTITGTMGHFILARCPKGETLTVGFTDSRKISHSCTHPFHHDPPVIGREKFHSRPQHGKELPCSTYVQSTAATTEEIEVHMPPDTPDRTLMSQQSGNVKITVNGQTVRYKCNCGGSNEGLTTTDKVINNCKIDQCHAAVTNHKKWQYNSPLVPRNAELGDRKGKIHIPFPLANVTCRVPKARNPTVTYGKNQVIMLLYPDHPTLLSYRNMGEEPNYQEEWVMHKKEVVLTVPTEGLEVTWGNNEPYKYWPQLSTNGTAHGHPHEIILYYYELYPTMTVVVVSVATFILLSMVGMAAGMCMCARRRCITPYELTPGATVPFLLSLICCIRTAKAATYQEAAIYLWNEQQPLFWLQALIPLAALIVLCNCLRLLPCCCKTLAFLAVMSRRCPHCERVRTRNSDPEHGGSTV

>ANH22454.1:1-824 truncated polyprotein [Chikungunya virus]MEFIPTQTFYNRRYQPRPWTPRSTIQIIRPRPRPQRQAGQLAQLISAVNKLTMRAVPQQKPRRNRKNKKQKQKQQAPQNNTNQKKQPPKKKPAQKKKKPGRRERMCMKIENDCIFEVKHEGKVTGYACLVGDKVMKPAHVKGTIDNADLAKLAFKRSSKYDLECAQIPVHMKSDASKFTHEKPEGYYNWHHGAVQYSGGRFTIPTGAGKPGDSGRPIFDNKGRVVAIVLGGANEGARTALSVVTWNKDIVTKITPEGAEEWSLAIPVMCLLANTTFPCSQPPCTPCCYEKEPEETLRMLEDNVMRPGYYQLLQASLTCSPHRQRRSTKDNFNVYKATRPYLAHCPDCGEGHSCHSPVALERIRNEATDGTLKIQVSLQIGIKTDDSHDWTKLRYMDNHMPADAERAGLFVRTSAPCTITGTMGHFILARCPKGETLTVGFTDSRKISHSCTHPFHHDPPVIGREKFHSRPQHGKELPCSTYVQSTAATTEEIEVHMPPDTPDRTLMSQQSGNVKITVNGQTVRYKCNCGGSNEGLTTTDKVINNCKVDQCHAAVTNHKKWQYNSPLVPRNAELGDRKGKIHIPFPLANVTCRVPKARNPTVTYGKNQVIMLLYPDHPTLLSYRNMGEEPNYQEEWVMHKKEVVLTVPTEGLEVTWGNNEPYKYWPQLSTNGTAHGHPHEIILYYYELYPTMTVVVVSVATFILLSMVGMAAGMCMCARRRCITPYELTPGATVPFLLSLICCIRTAKAATYQEAAIYLWNEQQPLFWLQALIPLAALIVLCNCLRLLPCCCKTLAFLAVMSRRCPHCERVRTRNSDPEHGGSTV

>ANH22478.1:1-824 truncated polyprotein [Chikungunya virus]MEFIPTQTFYNRRYQPRPWTPRSTIQIIRPRPRPQRQAGQLAQLISAVNKLTMRAVPQQKPRRNRKNKKQKQKQQAPQNNTNQKKQPPKKKPAQKKKKPGRRERMCMKIENDCIFEVKHEGKVTGYACLVGDKVMKPAHVKGTIDNADLAKLAFKRSSKYDLECAQIPVHMKSDASKFTHEKPEGYYNWHHGAVQYSGGRFTIPTGAGKPGDSGRPIFDNKGRVVAIVLGGANEGARTALSVVTWNKDIVTKITPEGAEEWSLAIPVMCLLANTTFPCSQPPCTPCCYEKEPEETLRMLEDNVMRPGYYQLLQASLTCSPHRQRRSTKDNFNVYKATRPYLAHCPDCGEGHSCHSPVALERIRNEATDGTLKIQVSLQIGIKTDDSHDWTKLRYMDNHMPADAERAGLFVRTSAPCTITGTMGHFILARCPKGETLTVGFTDSRKISHSCTHPFHHDPPVIGREKFHSRPQHGKELPCSTYVQSTAATTEEIEVHMPPDTPDRTLMSQQSGNVKITVNGQTVRYKCNCGGSNEGLTTTDKVINNCKVDQCHAAVTNHKKWQYNSPLVPRNAELGDRQGKIHIPFPLANVTCRVPKARNPTVTYGKNQVIMLLYPDHPTLLSYRNMGEEPNYQEEWVMHKKEVVLTVPTEGLEVTWGNNEPYKYWPQLSTNGTAHGHPHEIILYYYELYPTMTVVVVSVATFILLSMVGMAAGMCMCARRRCITPYELTPGATVPFLLSLICCIRTAKAATYQEAAIYLWNEQQPLFWLQALIPLAALIVLCNCLRLLPCCCKTLAFLAVMSRRCPHCERVRTRNSDPEHGGSTV

>QBB72850.1:1-824 truncated polyprotein [Chikungunya virus]MEYIPTQTFYNRRYQPRPWTPRPTIQVIRPRPRPQRQAGQLAQLISAVNKLTMRAVPQQKPRRNRKNKKQKQKRQAPQNNTNQKKQPPKKKPAQKKKKPGRRERMCMKIENDCIFEVKYEGKVTGYACLVGDKVMKPAHVKGTIDNADLAKLAFKRSSKYDLECAQIPVHMKSDASKFTHEKPEGYYNWHHGAVQYSGGRFTIPTGAGKPGDSGRPIFDNKGRVVAIVLGGANEGARTALSVVTWNKDIVTKITPEGAEEWSLAIPVMCLLANTTFPCSQPPCTPCCYEREPEETLRMLEDNVMRPGYYQLLQASLTCSPHRQRRSTKDNFNVYKATRPYLAHCPDCGEGHSCHSPVALERIRNEATDGTLKIQVSLQIGIKTDDSHDWTKLRYMDNHTPADAERAGLFVRTSAPCTITGTMGHFILTRCPKGETLTVGFTDSRKISHSCTHPFHHDPPVIGREKFHSRPQHGKELPCSTYVQSTAATTEEIEVHMPPDTPDRTLMSQQSGNVKITVNGQTVRYKCNCGGSNEGLITTDKVINNCKVDQCHAAVTNHKKWQYNSPLVPRNAELGDRKGKIHIPFPLANVTCRVPKARNPTVTYGKNQVIMLLYPDHPTLLSYRNMGEEPNYQEEWVTHKKEVVLTVPTEGLEVTWGNNEPYKYWPQLSTNGTAHGHPHEIILYYYELYPTMTVVVVSVASFVLLSMVGVAVGMCMCARRRCITPYELTPGATVPFLLSLICCIRTAKAATYQEAAVYLWNEQQPLFWLQALIPLAALIVLCNCLRLLPCCCKTLAFLAVMSRRCPHCERVRTRNSDPEHGGSTV

>ANH22466.1:1-824 truncated polyprotein [Chikungunya virus]MEFIPTQTFYNRRYQPRPWTPRSTIQIIRPRPRPQRQAGQLAQLISAVNKLTMRAVPQQKPRRNRKNKKQKQKQQAPQNNTNQKKQPPKKKPAQKKKKPGRRERMCMKIENDCIFEVKHEGKVTGYACLVGDKVMKPAHVKGTIDNADLAKLAFKRSSKYDLECAQIPVHMKSDASKFTHEKPEGYYNWHHGAVQYSGGRFTIPTGAGKPGDSGRPIFDNKGRVVAIVLGGANEGARTALSVVTWNKDIVTKITPEGAEEWSLAIPVMCLLANTTFPCSQPPCTPCCYEKEPEETLRMLEDNVMRPGYYQLLQASLTCSPHRQRRSTKNNFNVYKATRPYLAHCPDCGEGHSCHSPVALERIRNEATDGTLKIQVSLQIGIKTDDSHDWTKLRYMDNHMPADAERAGLFVRTSAPCTITGTMGHFILARCPKGETLTVGFTDSRKISHSCTHPFHHDPPVIGREKFHSRPQHGKELPCSTYVQSTAATTEEIEVHMPPDTPDRTLMSQQSGNVKITVNGQTVRYKCNCGGSNEGLTTTDKVINNCKVDQCHAAVTNHKKWQYNSPLVPRNAELGDRKGKIHIPFPLANVTCRVPKARNPTVTYGKNQVIMLLYPDHPTLLSYRNMGEEPNYQEEWVMHKKEVVLTVPTEGLEVTWGNNEPYKYWPQLSTNGTAHGHPHEIILYYYELYPTMTVVVVSVATFILLSMVGMAAGMCMCARRRCITPYELTPGATVPFLLSLICCIRTAKAATYQEAAIYLWNEQQPLFWLQALIPLAALIVLCNCLRLLPCCCKTLAFLAVMSRRCPHCERVRTRNSDPEHGGSTV

>QBB72877.1:1-824 truncated polyprotein [Chikungunya virus]MEYIPTQTFYNRRYQPRPWTPRPTIQVIRPRPRPQRQAGQLAQLISAVNKLTMRAVPQQKPRRNRKNKKQKQKRQAPQNNTNQKKQPPKKKPAQKKKKPGRRERMCMKIENDCIFEVKYEGKVTGYACLVGDKVMKPAHVKGTIDNADLAKLAFKRSSKYDLECAQIPVHMKSDASKFTHEKPEGYYNWHHGAVQYSGGRFTIPTGAGKPGDSGRPIFDNKGRVVAIVLGGANEGARTALSVVTWNKDIVTKITPEGAEEWSLAIPVMCLLANTTFPCSQPPCTPCCYEREPEETLRMLEDNVMRPGYYQLLQASLTCSPHRQRRSTKDNFNVYKATRPYLAHCPDCGEGHSCHSPVALERIRNEATDGTLKIQVSLQIGIKTDDSHDWTKLRYMDNHTPADAERAGLFVRTSAPCTITGTMGHFILTRCPKGETLTVGFTDSRKISHSCTHPFHHDPPVIGREKFHSRPQHGKELPCSTYVQSTAATTEEIEVHMPPDTPDRTLMSQQSGNVKITVNGQTVRYKCNCGGSNEGLITTDKVINNCKVDQCHAAVTNHKKWQYNSPLVPRNAELGDRKGKIHIPFPLANVTCRVPKARNPTVTYGKNQVIMLLYPDHPTLLSYRNMGEEPNYQEEWVTHKKEVVLTVPTEGLEVTWGNNEPYKYWPQLSTNGTAHGHPHEIILYYYELYPTMTVVVVSVASFVLLSMVGVAVGMCMCARRRCITPYELTPGATVPFLLSLICCIRTAKAATYQEAAVYLWNEQQPLFWLQALIPLAALIVLCNCLRLLPCCCKTMAFLAVMSRRCPHCERVRTRNSDPEHGGSTV

>QBB72844.1:1-824 truncated polyprotein [Chikungunya virus]MEYIPTQTFYNRRYQPRPWTPRPTIQVIRPRPRPQRQAGQLAQLISAVNKLTMRAVPQQKPRRNRKNKKQKQKRQAPQNNTNQKKQPPKKKPAQKKKKPGRRERMCMKIENDCIFEVKYEGKVTGYACLVGDKVMKPAHVKGTIDNADLAKLAFKRSSKYDLECAQIPVHMKSDASKFTHEKPEGYYNWHHGAVQYSGGRFTIPTGAGKPGDSGRPIFDNKGRVVAIVLGGANEGARTALSVVTWNKDIVTKITPEGAEEWSLAIPVMCLLANTTFPCSQPPCTPCCYEREPEETLRMLEDNVMRPGYYQLLQASLTCSPHRQRRSTKDNFNVYKATRPYLAHCPDCGEGHSCHSPVALERIRNEATDGTLKIQVSLQIGIKTDDSHDWTKLRYMDNHTPADAERAGLFVRTSAPCTITGTMGHFILTRCPKGETLTVGFTDSRKISHSCTHPFHHDPPVIGREKFHSRPQHGKELPCSTYVQSTAATTEEIEVHMPPDTPDRTLMSQQSGNVKITVNGQTVRYKCNCGGSNEGLITTDKVINNCKVDQCHAAVTNHKKWQYNSPLVPRNAELGDRKGKIHIPFPLANVTCRVPKARNPTVTYGKNQVIMLLYPDHPTLLSYRNMGEEPNYQEEWVTHKKEVVLTVPTEGLEVTWGNNEPYKYWPQLSTNGTAHGHPHEIILYYYELYPTMTVVVVSVASFVLLSMVGVAVGMCMCARRRCITPYELTPGATVPFLLSLICCIRTAKAATYQEAAVYLWNEQQPLFWLQALIPLAALIVLCNCLRLLPCCCKTLAFLAVLSRRCPHCERVRTRNSDPEHGGSTV

>AQX78105.1:1-824 truncated polyprotein [Chikungunya virus]MEFIPTQTFYNRRYQPRPWTPRSTIQIIRPRPRPQRQAGQLAQLISAVNKLTMRAVPQQKPRRNRKNKKQKQKQQAPQNNTNQKKQPPKKKPAQKKKKPGRRERMCMKIENDCIFEVKHEGKVTGYACLVGDKVMKPAHVKGTIDNADLAKLAFKRSSKYDLECAQIPVHMKSDASKFTHEKPEGYYNWHHGAVQYSGGRFTIPTGAGKPGDSGRPIFDNKGRVVAIVLGGANEGARTALSVVTWNKDIVTKITPEGAEEWSLAIPVMCLLANTTFPCSQPPCTPCCYEKEPEETLRMLEDNVMRPGYYQLLQASLTCSPHRQRRSTKDNFNVYKATRPYLAHCPDCGEGHSCHSPVALERIRNEATDGTLKIQVSLQIGIKTDDSHDWTKLRYMDNHMPADAERAGLFVRTSALCTITGTMGHFILARCPKGETLTVGFTDSRKISHSCTHPFHHDPPVIGREKFHSRPQHGKELPCSTYVQSTAATTEEIEVHMPPDTPDRTLMSQQSGNVKITVNGQTVRYKCNCGGSNEGLTTTDKVINNCKVDQCHAAVTNHKKWQYNSPLVPRNAELGDRKGKIHIPFPLANVTCRVPKARNPTVTYGKNQVIMLLYPDHPTLLSYRNMGEEPNYQEEWVMHKKEVVLTVPTEGLEVTWGNNEPYKYWPQLSTNGTAHGHPHEIILYYYELYPTMTVVVVSVATFILLSMVGMAAGMCMCARRRCITPYELTPGATVPFLLSLICCIRTAKAATYQEAAIYLWNEQQPLFWLQALIPLAALIVLCNCLRLLPCCCKTLAFLAVMSRRCPHCERVRTRNSDPEHGGSTV

>NP_690589.2:1-798 structural polyprotein [Chikungunya virus]MEFIPTQTFYNRRYQPRPWTPRPTIQVIRPRPRPQRQAGQLAQLISAVNKLTMRAVPQQKPRKNRKNKKQKQKQQAPQNNTNQKKQPPKKKPAQKKKKPGRRERMCMKIENDCIFEVKHEGKVTGYACLVGDKVMKPAHVKGTIDNADLAKLAFKRSSKYDLECAQIPVHMKSDASKFTHEKPEGYYNWHHGAVQYSGGRFTIPTGAGKPGDSGRPIFDNKGRVVAIVLGGANEGARTALSVVTWNKDIVTKITPEGAEEWSLAIPVMCLLANTTFPCSQPPCIPCCYEKEPEETLRMLEDNVMRPGYYQLLQASLTCSPHRQRRSTKDNFNVYKATRPYLAHCPDCGEGHSCHSPVALERIRNEATDGTLKIQVSLQIGIGTDDSHDWTKLRYMDNHIPADAGRAGLFVRTSAPCTITGTMGHFILARCPKGETLTVGFTDSRKISHSCTHPFHHDPPVIGREKFHSRPQHGKELPCSTYVQSNAATAEEIEVHMPPDTPDRTLLSQQSGNVKITVNSQTVRYKCNCGGSNEGLITTDKVINNCKVDQCHAAVTNHKKWQYNSPLVPRNAELGDRKGKIHIPFPLANVTCMVPKARNPTVTYGKNQVIMLLYPDHPTLLSYRSMGEEPNYQEEWVTHKKEVVLTVPTEGLEVTWGNNEPYKYWPQLSANGTAHGHPHEIILYYYELYPTMTVVVVSVASFILLSMVGMAVGMCMCARRRCITPYELTPGATVPFLLSLICCIRTAKAATYQEAAVYLWNEQQPLFWLQALIPLAALIVLCNCLRLLPCCCKTLAFLA

>ANH22475.1:1-824 truncated polyprotein [Chikungunya virus]MEFIPTQTFYNRRYQPRPWTPRPTIQVIRPRPRPQRRAGQLAQLISAVNKLTMRAVPQQKPRRNRKNKKQKQKQQAPQNNTNQKKQPPKKKPAQKKKKPGRRERMCMKIENDCIFEVKHEGRVTGYACLVGDKVMKPAHVKGTIDNADLAKLAFKRSSKYDLECAQIPVHMKSDASKFTHEKPEGYYNWHHGAVQYSGGRFTIPTGAGKPGDSGRPIFDNKGRVVAIVLGGANEGARTALSVVTWNKDIVTKITPEGAEEWSLAIPVMCLLANTTFPCSQPPCTPCCYEKEPEETLRMLEDNVMRPGYYQLLQASLTCSPRRQRRSTKDNFNVYKATRPYLAHCPDCGEGHSCHSPVALERIRNEATDGTLKIQVSLQIGIKTDDSHDWTKLRYMDNHTPADAERAGLFVRTSAPCTITGTMGHFILARCPKGETLTVGFTDGRKISHSCTHPFHHDPPVIGREKFHSRPQHGRELPCSTYVQSTAATTEEIEVHMPPDTPDRTLMSQQSGNVKITVNGQTVRYKCNCGGSNEGLTTTDKVINNCKIDQCHAAVTNHKKWQYNSPLVPRNDELGDRKGKIHIPFPLANVTCRVPKARNPTVTYGKNQVIMLLYPDHPTLLSYRNMGEEPNYQEEWVTHKKEVVLTVPTEGLEVTWGNNEPYKYWPQLSTNGTAHGHPHEIILYYYELYPTMTVVVVSVASFILLSMVGMAVGMCMCARRRCITPYELTPGATVPFLLSLLCCIRTAKAATYQEAAVYLWNEQQPLFWLQALIPLAALIVLCNCLRLLPCCCKTLAFLAVMSRRCPHCERVRTRNSDPEHGGSTV

>QBB72856.1:1-824 truncated polyprotein [Chikungunya virus]MEYIPTQTFYNRRYQPRPWTPRPTIQVIRPRPRPQRQAGQLAQLISAVNKLTMRAVPQQKPRRNRKNKKQKQKRQAPQNNTNQKKQPPKKKPAQKKKKPGRRERMCMKIENDCIFEVKYEGKVTGYACLVGDKVMKPAHVKGTIDNADLAKLAFKRSSKYDLECAQIPVHMKSDASKFTHEKPEGYYNWHHGAVQYSGGRFTIPTGAGKPGDSGRPIFDNKGRVVAIVLGGANEGARTALSVVTWNKDIVTKITPEGAEEWSLAIPVMCLLANTTFPCSQPPCTPCCYEREPEETLRMLEDNVMRPGYYQLLQASLTCSPHRQRRSTKDNFNVYKATRPYLAHCPDCGEGHSCHSPVALERIRNEATDGTLKIQVSLQIGIKTDDSHDWTKLRYMDNHTPADAERAGLFVRTSAPCTITGTMGHFILTRCPKGETLTVGFTDSRKISHSCTHPFHHDPPVIGREKFHSRPQHGKELPCSTYVQSTAATTEEIEVHMPPDTPDHTLMSQQSGNVKITVNGQTVRYKCNCGGSNEGLITTDKVINNCKVDQCHAAVTNHKKWQYNSPLVPRNAELGDRKGKIHIPFPLANVTCRVPKARNPTVTYGKNQVIMLLYPDHPTLLSYRNMGEEPNYQEEWVTYKKEVVLTVPTEGLEVTWGNNEPYKYWPQLSTNGTAHGHPHEIILYYYELYPTMTVVVVSVASFVLLSMVGVAVGMCMCARRRCITPYELTPGATVPFLLSLICCIRTAKAATYQEAAVYLWNEQQPLFWLQALIPLAALIVLCNCLRLLPCCCKTLAFLAVMSRRCPHCERVRTRNSDPEHGGSTV

>AYI50336.1:1-824 truncated structural polyprotein [Chikungunya virus]MEFIPTQTFYNRRYQPRPWTPRPTIQVIRPRPRPQRKAGQLAQLISAVNKLTMRAVPQQKPRKNRKNKKQKQKQQAPRNNMNQKKQPPKKKPAQKKKKPGRRERMCMKIENDCIFEVKHEGKVTGYACLVGDKVMKPAHVKGTIDNADLAKLAFKRSSKYDLECAQIPVHMKSDASKFTHEKPEGYYNWHHGAVQYSGGRFTIPTGAGKPGDSGRPIFDNKGRVVAIVLGGANEGARTALSVVTWNKDIVTKITPEGAEEWSLAIPVMCLLANTTFPCSQPPCTPCCYEKEPEKTLRMLEDNVMSPGYYQLLQASLTCSPRRQRRSIKDNFNVYKATRPYLAHCPDCGEGHSCHSPVALERIRNEATDGTLKIQVSLQIGIKTDDSHDWTKLRYMDNHMPADAERAGLFVRTSAPCTITGTMGHFILARCPKGETLTVGFTDGRKISHSCTHPFHHDPPVIGREKFHSRPQHGRELPCSTYAQSTAATAEEIEVHMPPDTPDRTLMSQQSGNVKITVNSQTVRYKCNCGDSNEGLTTTDKVINNCKVDQCHAAVTNHKKWQYNSPLVPRNAELGDRKGKVHIPFPLANVTCRVPKARNPTVTYGKNQVIMLLYPDHPTLLSYRNMGEEPNYQEEWVTHKKEIRLTVPTEGLEVTWGNNEPYKYWPQLSTNGTAHGHPHEIILYYYELYPTMTVVVVSVASFVLLSMVGVAVGMCMCARRRCITPYELTPGATVPFLLSLICCIRTAKAATYQEAAVYLWNEQQPLFWLQALIPLAALIVLCNCLRLLPCCCKTLTFLSRNERRCPHCERVRTRNSDPEHGGSTV

>Q8JUX5.3:1-798 RecName: Full=Structural polyprotein; AltName: Full=p130; Contains: RecName: Full=Capsid protein; AltName: Full=Coat protein; Short=C; Contains: RecName: Full=Precursor of protein E3/E2; AltName: Full=p62; AltName: Full=pE2; Contains: RecName: Full=Assembly protein E3; Contains: RecName: Full=Spike glycoprotein E2; AltName: Full=E2 envelope glycoprotein; Contains: RecName: Full=6K protein; Contains: RecName: Full=Spike glycoprotein E1; AltName: Full=E1 envelope glycoprotein [Chikungunya virus strain S27-African prototype]MEFIPTQTFYNRRYQPRPWTPRPTIQVIRPRPRPQRQAGQLAQLISAVNKLTMRAVPQQKPRRNRKNKKQKQKQQAPQNNTNQKKQPPKKKPAQKKKKPGRRERMCMKIENDCIFEVKHEGKVTGYACLVGDKVMKPAHVKGTIDNADLAKLAFKRSSKYDLECAQIPVHMKSDASKFTHEKPEGYYNWHHGAVQYSGGRFTIPTGAGKPGDSGRPIFDNKGRVVAIVLGGANEGARTALSVVTWNKDIVTKITPEGAEEWSLAIPVMCLLANTTFPCSQPPCIPCCYEKEPEETLRMLEDNVMRPGYYQLLQASLTCSPHRQRRSTKDNFNVYKATRPYLAHCPDCGEGHSCHSPVALERIRNEATDGTLKIQVSLQIGIGTDDSHDWTKLRYMDNHIPADAGRAGLFVRTSAPCTITGTMGHFILARCPKGETLTVGFTDSRKISHSCTHPFHHDPPVIGREKFHSRPQHGKELPCSTYVQSNAATAEEIEVHMPPDTPDRTLLSQQSGNVKITVNGRTVRYKCNCGGSNEGLITTDKVINNCKVDQCHAAVTNHKKWQYNSPLVPRNAELGDRKGKIHIPFPLANVTCMVPKARNPTVTYGKNQVIMLLYPDHPTLLSYRSMGEEPNYQEEWVTHKKEVVLTVPTEGLEVTWGNNEPYKYWPQLSANGTAHGHPHEIILYYYELYPTMTVVVVSVASFILLSMVGMAVGMCMCARRRCITPYELTPGATVPFLLSLICCIRTAKAATYQEAAVYLWNEQQPLFWLQALIPLAALIVLCNCLRLLPCCCKTLAFLA

>ATW74975.1:1-798 structural polyprotein [Chikungunya virus]MEFIPTQTFYNRRYQPRPWTPRPTIQVIRPRPRPQRQAGQLAQLISAVNKLTMRAVPQQKPRRNRKNKKQKQKQQAPQNNTNQKKQPPKKKPAQKKKKPGRRERMCMKIENDCIFEVKHEGKVTGYACLVGDKVMKPAHVKGTIDNADLAKLAFKRSSKYDLECAQIPVHMKSDASKFTHEKPEGYYNWHHGAVQYSGGRFTIPTGAGKPGDSGRPIFDNKGRVVAIVLGGANEGARTALSVVTWNKDIVTKITPEGAEEWSLAIPVMCLLANTTFPCSQPPCIPCCYEKEPEETLRMLEDNVMRPGYYQLLQASLTCSPHRQRRSTKDNFNVYKATRPYLAHCPDCGEGHSCHSPVALERIRNEATDGTLKIQVSLQIGIGTDDSHDWTKLRYMDNHIPADAGRAGLFVRTSAPCTITGTMGHFILARCPKGETLTVGFTDSRKISHSCTHPFHHDPPVIGREKFHSRPQHGKELPCSTYVQSNAATAEEIEVHMPPDTPDRTLLSQQSGNVKITVNGRTVRYKCNCGGSNEGIITTDKVINNCKVDQCHAAVTNHKKWQYNSPLVPRNAELGDRKGKIHIPFPLANVTCMVPKARNPTVTYGKNQVIMLLYPDHPTLLSYRSMGEEPNYQEEWVTHKKEVVLTVPTEGLEVTWGNNEPYKYWPQLSANGTAHGHPHEIILYYYELYPTMTVVVVSVASFILLSMVGMAVGMCMCARRRCITPYELTPGATVPFLLSLICCIRTAKAATYQEAAVYLWNEQQPLFWLQALIPLAALIVLCNCLRLLPCCCKTLAFLA

>ANH22451.1:1-824 truncated polyprotein [Chikungunya virus]MEFIPTQTFYNRRYQPRPWTPRPTIQVIRPRPRPQRKAGQLAQLISAVNKLTMRAVPQQKPRKNRKNKKQKQKQQAPRNNTNQKKQPPKKKPVQKKKKPGRRERMCMKIENDCIFEVKHEGKVTGYACLVGDKVMKPAHVKGTIDNADLAKLAFKRSSKYDLECAQIPVHMKSDASKFTHEKPEGYYNWHHGAVQYSGGRFTIPTGAGKPGDSGRPIFDNKGRVVAIVLGGANEGARTALSVVTWNKDIVTKITPEGAEEWSLAIPVMCLLANTTFPCSQPPCTPCCYEKEPEKTLRMLEDNVMSPGYYQLLQASLTCSPRRQRRSIKDNFNVYKATRPYLAHCPDCGEGHSCHSPVALERIRNEATDGTLKIQVSLQIGIKTDDSHDWTKLRYMDNHMPADAERAGLFVRTSAPCTITGTMGHFILARCPKGETLTVGFTDGRKISHSCTHPFHHDPPVIGREKFHSRPQHGRELPCSTYAQSTAATAEEIEVHMPPDTPDRTLMSQQSGNVKITVNSQTVRYKCNCGDSNEGLTTTDKVINNCKVDQCHAAVTNHKKWQYNSPLVPRNAELGDRKGKVHIPFPLANVTCRVPKARNPTVTYGKNQVIMLLYPDHPTLLSYRNMGEEPNYQEEWVTHKKEIRLTVPTEGLEVTWGNNEPYKYWPQLSTNGTAHGHPHEIILYYYELYPTMTVVVVSVASFVLLSMVGVAVGMCMCARRRCITPYELTPGATVPFLLSLICCIRTAKAATYQEAAVYLWNEQQPLFWLQALIPLAALIVLCNCLRLLPCCCKTLTFLAVLSHRCPHCERVRTRNSDPEHGGSTV

>AYI50333.1:1-824 truncated structural polyprotein [Chikungunya virus]MEFIPTQTFYNRRYQPRPWTPRPTIQVIRPRPRPQRKAGQLAQLISAVNKLTMRAVPQQKPRKNRKNKKQKQKQQAPRNNMNQKKQPPKKKPAQKKKKPGRRERMCMKIENDCIFEVKHEGKVTGYACLVGDKVMKPAHVKGTIDNADLAKLAFKRSSKYDLECAQIPVHMKSDASKFTHEKPEGYYNWHHGAVQYSGGRFTIPTGAGKPGDSGRPIFDNKGRVVAIVLGGANEGARTALSVVTWNKDIVTKITPEGAEEWSLAIPVMCLLANTTFPCSQPPCTPCCYEKEPEKTLRMLEDNVMSPGYYQLLQASLTCSPRRQRRSIKDNFNVYKAIRPYLAHCPDCGEGHSCHSPVALERIRNEATDGTLKIQVSLQIGIKTDDSHDWTKLRYMDNHMPADAERARLFVRTSAPCTITGTMGHFILARCPKGETLTVGFTDGRKISHSCTHPFHHDPPVIGREKFHSRPQHGRELPCSTYAQSTAATAEEIEVHMPPDTPDRTLMSQQSGNVKITVNSQTVRYKCNCGDSNEGLTTTDKVINNCKVDQCHAAVTNHKKWQYNSPLVPRNAELGDRKGKVHIPFPLANVTCRVPKARNPTVTYGKNQVIMLLYPDHPTLLSYRNMGEEPNYQEEWVTHKKEIRLTVPTEGLEVTWGNNEPYKYWPQLSTNGTAHGHPHEIILYYYELYPTMTVVVVSVASFVLLSMVGVAVGMCMCARRRCITPYELTPGATVPFLLSLICCIRTAKAATYQEAAVYLWNEQQPLFWLQALIPLAALIVLCNCLRLLPCFCKTLTFLSRNERRCPHCERVRTRNSDPEHGGSTV

>ADG95932.1:1-798 structural polyprotein [Chikungunya virus]MEFIPTQTFYNRRYQPRPWTPRPTIQVIRPRPRPQRQAGQLAQLISAVNKLTMRAVPQQKPRRNRKNKKQKQKQQAPQNNTNQKKQPPKKKPAQKKKKPGRRERMCMKIENDCIFEVKHEGKVTGYACLVGDKVMKPAHVKGTIDNADLAKLAFKRSSKYDLECAQIPVHMKSDASKFTHEKPEGYYNWHHGAVQYSGGRFTIPTGAGKPGDSGRPIFDNKGRVVAIVLGGANEGARTALSVVTWNKDIVTKITPEGAEEWSLAIPVMCLLANTTFPCSQPPCTPCCYEKEPEETLRMLEDNVMRPGYYQLLQASLTCSPHRQRRSTKDNFNVYKATRPYLAHCPDCGEGHSCHSPVALERIRNEATDGTLKIQVSLQIGIKTDGSHDWTKLRYMDNHMPADAERAGLFVRTSAPCTITGTMGHFILARCPKGETLTVGFTDSRKISHSCTHPFHHDPPVIGREKFHSRPQHGKELPCSTYVQSTAATAEEIEVHMPPDTPDRTLMSQQSGNVKITVNGQTVRYKCNCGGSNEGLITTDKVINNCRVDQCHAAVTNHKKWQYNSPLVPRNAELGDRKGKIHIPFPLANVTCRVPKARNPTVTYGKNQVIMLLYPDHPTLLSYRSMGEEPNYQEEWVTHKKEVVLTVPTEGLEVTWGNNEPYKYWPQLSANGTAHGHPHEIILYYYELYPTMTVVVVSVASFILLSMVGMAVGMCMCARRRCITPYELTPGATVPFLLSLICCIRTAKAATYQEAAVYLWNEQQPLFWLQALIPLAALIVLCNCLRLLPCCCKTLAFLA

>ASZ70604.1:1-798 structural polyprotein [Chikungunya virus]MEFIPTQTFYNRRYQPRPWTPRPTIQVIRPRPRPQRQAGQLAQLISAVNKLTMRAVPQQKPRRNRKNKKQKQKQQAPQNNTNQKKQPPKKKPAQKKKKPGRRERMCMKIENDCIFEVKHEGKVTGYACLVGDKVMKPAHVKGTIDNADLAKLAFKRSSKYDLECAQIPVHMKSDASKFTHEKPEGYYNWHHGAVQYSGGRFTIPTGAGKPGDSGRPIFDNKGRVVAIVLGGANEGARTALSVVTWNKDIVTKITPEGAEEWSLAIPVMCLLANTTFPCSQPPCTPCCYEKEPEETLRMLEDNVMRPGYYQLLQASLTCSPHRQRRSTKDNFNVYKATRPYLAHCPDCGEGHSCHSPVALERIRNEATDGTLKIQVSLQIGIKTDDSHDWTKLRYMDNHLPADAERAGLFVRTSAPCTITGTMGHFILARCPRGETLTVGFTDSRKISHSCTHPFHHDPPVIGREKFHSRPQHGKELPCSTYVQSTAATAEEIEVHMPPDTPDRTLMSQQSGNVKITVNGQTVRYKCNCGGSNEGLITTDKVINNCKVDQCHAAVTNHKKWQYNSPLVPRNAELGDRKGKIHIPFPLANVTCRVPKARNPTVTYGKNQVIMLLYPDHPTLLSYRNMGEEPNYQEEWVAHKKEVVLTVPTEGLEVTWGNNEPYKYWPQLSTNGTAHGHPHEIILYYYELYPTMTVVVVSVASFILLSMVGMAVGMCMCARRRCITPYELTPGATVPFLLSLICCIRTAKAATYQEAAVYLWNEQQPLFWLQALIPLAALIVLCNCLRLLPCCCKTLAFLA

>ADG95899.1:1-798 structural polyprotein [Chikungunya virus]MEFIPTQTFYNRRYQPRPWTPRPTIQVIRPRPRPQRQAGQLAQLISAVNKLTMRAVPQQKPRRNRKNKKQKQKQQAPQNNTNQKKQPPKKKPAQKKKKPGRRERMCMKIENDCIFEVKHEGKVTGYACLVGDKVMKPAHVKGTIDNADLAKLAFKRSSKYDLECAQIPVHMKSDASKFTHEKPEGYYNWHHGAVQYSGGRFTIPTGAGKPGDSGRPIFDNKGRVVAIVLGGANEGARTALSVVTWNKDIVTKITPEGAEEWSLAIPVMCLLANTTFPCSQPPCTPCCYEKEPEETLRMLEDNVMRPGYYQLLQASLTCSPHRQRRSTKDNFNVYKATRPYLAHCPDCGEGHSCHSPVALERIRNEATDGTLKIQVSLQIGIKTDDSHDWTKLRYMDNHMPADAERAGLFVRTSAPCTITGTMGHFILARCPKGETLTVGFTDGRKISHSCTHPFHHDPPVIGREKFHSRPQHGKELPCSTYVQSTAATTEEIEVHMPPDTPDRTLMSQQSGNVKITVNGQTVRYKCNCGGSNEGLITTDKVINNCKVDQCHAAVTNHKKWQYNSPLVPRNAELGDRKGKIHIPFPLANVTCRVPKARNPTVTYGKNQVIMLLYPDHPTLLSYRNMGEEPNYQEEWVTHKKEVVLTVPTEGLEVTWGNNEPYKYWPQLSTNGTAHGHPHEIILYYYELYPTMTVVVVSVASFVLLSMVGMAVGMCMCARRRCITPYELTPGATVPFLLSLICCIRTAKAATYQEAAVYLWNEQQPLFWLQALIPLAALIVLCNCLRLLPCCCKTLAFLA

>ADG95923.1:1-798 structural polyprotein [Chikungunya virus]MEFIPTQTFYNRRYQPRPWTPRPTIQVIRPRPRPQRQAGQLAQLISAVNKLTMRAVPQQKPRRNRKNKKQKQKQQAPQNNTNQKKQPPKKKPAQKKKKPGRRERMCMKIENDCIFEVKHEGKVTGYACLVGDKVMKPAHVKGTIDNADLAKLAFKRSSKYDLECAQIPVHMKSDASKFTHEKPEGYYNWHHGAVQYSGGRFTIPTGAGKPGDSGRPIFDNKGRVVAIVLGGANEGARTALSVVTWNKDIVTKITPEGAEEWSLAIPVMCLLANTTFPCSQPPCIPCCYEKEPEETLRMLEDNVMRPGYYQLLQASLTCSPHRQRRSTKDNFNVYKATRPYLAQCPDCGEGHSCHSPVALERIRNEATDGTLKIQVSLQLGIGTDDSHDWTKLRYMDNHIPADAGRAGLFVRTSAPCTITGTMGHFILARCPKGETLTVGFTDSRKISHSCTHPFHHDPPVIGREKFHSRPQHGKELPCSTYVQSTAATADEIEVHVPPDTPDRTLLSQQSSNVTITVNGRTVRYKCNCGGSNEGTITTDKVINNCKIDQCHAAVTNHKKWQYNSPLVPRNAELGDRKGKIHIPFLLANVTCMVPKARNPTVTYGKNQVIMLLYPDHPTLLSYRSMGEEPNYQEEWVTHKKEVVLTVPTEGLEVTWGNNEPYKYWPQLSANGTAHGHPHEIILYYYELYPTMTVVVVSVASFILLSMVGMAVGMCMCARRRCITPYELTPGATVPFLLSLTCCIRTAKAATYQEAAVYLWNEQQPLFWLQALIPLAALIVLCNCLRLLPCCCKTLAFLA

>ADG95897.1:1-798 structural polyprotein [Chikungunya virus]MEFIPTQTFYNRRYQPRPWTPRPTIQVIRPRPRPQRQAGQLAQLISAVNKLTMRAVPQQKPRRNRKNKKQKQKQQAPQNNTNQKKQPPKKKPAQKKKKPGRRERMCMKIENDCIFEVKHEGKVTGYACLVGDKVMKPAHVKGTIDNADLAKLAFKRSSKYDLECAQIPVHMKSDASKFTHEKPEGYYNWHHGAVQYSGGRFTIPTGAGKPGDSGRPIFDNKGRVVAIVLGGANEGARTALSVVTWNKDIVTKITPEGAEEWSLAIPVMCLLANTTFPCSQPPCTPCCYEKEPEETLRMLEDNVMRPGYYQLLQASLTCSPHRQRRSTKDNFNVYKATRPYLAHCPDCGEGHSCHSPVALERIRNEATDGTLKIQVSLQIGIKTDGSHDWTKLRYMDSHMPADAERAGLFVRTSAPCTITGTMGHFILARCPKGETLTVGFTDSRKISHSCTHPFHHDPPVIGREKFHSRPQHGKELPCSTYVQSTAVTAEEIEVHMPPDTPDRTLMSQQSGNVKITVNGQTVRYKCNCGGSNEGLITTDKVINNCKVDQCHAAVTNHKKWQYNSPLVPRNAELGDRKGKIHIPFPLANVTCRVPKARNPTVTYGKNQVIMLLYPDHPTLLSYRNMGEEPNYQEEWVTHKKEVVLTVPTEGLEVTWGNNEPYKYWPQLSANGTAHGHPHEIILYYYELYPTMTVVVVSVASFILLSMVGMAVGMCMCARRRCITPYELTPGATVPFLLSLICCIRTAKAATYQEAAVYLWNEQQPLFWLQALIPLAALIVLCNCLRLLPCCCKTLAFLA

>ABN04198.1:1-798 structural polyprotein [Chikungunya virus]MEFIPTQTFYNRRYQPRPWTPRPTIQVIRPRPRPQRQAGQLAQLISAVNKLTMRAVPQQKPRRNRKNKKQKQKQQAPQNNTNQKKQPPKKKPAQKKKKPGRRERMCMKIENDCIFEVKHEGKVTGYACLVGDKVMKPAHVKGTIDNADLAKLAFKRSSKYDLECAQIPVHMKSDASKFTHEKPEGYYNWHHGAVQYSGGRFTIPTGAGKPGDSGRPIFDNKGRVVAIVLGGANEGARTALSVVTWNKDIVTKITPEGAEEWSLAIPVMCLLANTTFPCSQPPCTPCCYEKEPEETLRMLEDNVMRPGYYQLLQASLTCSPHRQRRSTKDNFNVYKATRPYLAHCPDCGEGHSCHSPVALERIRNEATDGTLKIQVSLQIGIKTDDSHDWTKLRYMDNHMPADAERAGLFVRTSAPCTITGTMGHFILARCPKGETLTVGFTDSRKISHSCTHPFHHDPPVIGREKFHSRPQHGKELPCSTYVQSTAVTTEEIEVHMPPDTPDRTLMSQQSGNVKITVNGQTVRYKCNCGGSNEGLITTDKVINNCKVDQCHAAVTNHKKWQYNSPLVPRNAELGDRKGKIHIPFPLANVTCRVPKARNPTVTYGKNQVIMLLYPDHPTLLSYRNMGEEPNYQEEWVTHKKEVMLTVPTEGLEVTWGNNEPYKYWPQLSTNGTAHGHPHEIILYYYELYPTMTVVVVSVASFVLLSMVGMAVGMCMCARRRCITPYELTPGATVPFLLSLICCIRTAKAATYQEAAVYLWNEQQPLFWLQALIPLAALIVLCNCLRLLPCCCKTLAFLA

>ADG95956.1:1-798 structural polyprotein [Chikungunya virus]MEFIPTQTFYNRRYQPRPWTPRPTIQVIRPRPRPQRQAGQLAQLISAVNKLTMRAVPQQKPRRNRKNKKQKQKQQAPQNNTNQKKQPPKKKPAQKKKKPGRRERMCMKIENDCIFEVKHEGKVTGYACLVGDKVMKPAHVKGTIDNADLAKLAFKRSSKYDLECAQIPVHMKSDASKFTHEKPEGYYNWHHGAVQYSGGRFTIPTGAGKPGDSGRPIFDNKGRVVAIVLGGANEGARTALSVVTWNKDIVTKITPEGAEEWSLAIPVMCLLANTTFPCSQPPCTPCCYEKEPEETLRMLEDNVMRPGYYQLLQASLTCSPHRQRRSTKDNFNVYKATRPYLAHCPDCGEGHSCHSPVALERIRNEATDGTLKIQVSLQIGIKTDGSHDWTKLRYMDNHMPADAERAGLFVRTSAPCTITGTMGHFILARCPKGETLTVGFTDSRKISHSCTHPFHHDPPVIGREKFHSRPQHGKELPCSTYVQSTAATTEEIEVHMPPDTPDRTLMSQQSGNVKITVNGQTVRYKCNCGGSNEGLITTDKVINNCKVDQCHAAVTNHKKWQYNSPLVPRNAELGDRKGKIHIPFPLANVTCRVPKARNPTVTYGKNQVIMLLYPDHPTLLSYRNMGEEPNYQEEWVTHKKEVVLTVPTEGLEVTWGNNEPYKYWPQLSTNGTAHGHPHEIILYYYELYPTMTVVVVSVASFVLLSMVGVAVGMCMCARRRCITPYELTPGATVPFLLSLICCIRTAKAATYQEAAVYLWNEQQPLFWLQALIPLAALIVLCNCLRLLPCCCKTLAFLA

>ADG95902.1:1-798 structural polyprotein [Chikungunya virus]MEFIPTQTFYNRRYQPRPWTPRPTIQVIRPRPRPQRQAGQLAQLISAVNKLTMRAVPQQKPRRNRKNKKQKQKQQAPQNNTNQKKQPPKKKPAQKKKKPGRRERMCMKIENDCIFEVKHEGKVTGYACLVGDKVMKPAHVKGTIDNADLAKLAFKRSSKYDLECAQIPVHMKSDASKFTHEKPEGYYNWHHGAVQYSGGRFTIPTGAGKPGDSGRPIFDNKGRVVAIVLGGANEGARTALSVVTWNKDIVTKITPEGAEEWSLAIPVMCLLANTTFPCSQPPCTPCCYEKEPEETLRMLEDNVMRPGYYQLLQASLTCSPHRQRRSTKDNFNVYKATRPYLAHCPDCGEGHSCHSPVALERIRNEATDGTLKIQVSLQIGIKTDDSHDWTKLRYMDNHMPADAERAGLFVRTSAPCTITGTXGHFILARCPKGETLTVGFTDSRKISHSCTHPFHHDPPVIGREKFHSRPQHGKELPCSTYVQNTAATAEEIEVHMPPDTPDRTLMSQQSGNVKITVNGQTVRYKCNCGGSNEGLTTTDKVINNCKVDQCHAAVTNHKKWQYNSPLVPRNAELGDRKGKIHIPFPLANVTCRVPKARNPTVTYGKNQVIMLLYPDHPTLLSYRNMGEEPNYQEEWVTYKKEVVLTVPTEGLEVTWGNNEPYKYWPQLSANGTAHGHPHEIILYYYELYPTMTVVVVSVASFILLSMVGMAVGMCMCARRRCITPYELTPGATVPFLLSLICCFRTAKAATYQEAAVYLWNEQQPLFWLQALIPLAALIVLCNCLRLLPCCCKTLAFLA

>ADG95881.1:1-798 structural polyprotein [Chikungunya virus]MEFIPTQTFYNRRYQPRPWTPRPTIQVIRPRPRPQRQAGQLAQLISAVNKLTMRAVPQQKPRRNRKNKKQKQKQQAPQNNTNQKKQPPKKKPAQKKKKPGRRERMCMKIENDCIFEVKHEGKVTGYACLVGDKVMKPAHVKGTIDNADLAKLAFKRSSKYDLECAQIPVHMKSDASKFTHEKPEGYYNWHHGAVQYSGGRFTIPTGAGKPGDSGRPIFDNKGRVVAIVLGGANEGARTALSVVTWNKDIVTKITPEGAEEWSLAIPVMCLLANTTFPCSQPPCTPCCYEKEPEETLRMLEDNVMRPGYYQLLQASLTCSPHRQRRSTKDNFNVYKATRPYLAHCPDCGEGHSCHSPVALERIRNEATDGTLKIQVSLQIGIKTDDSHDWTKLRYMDNHMPADAERAGLFVRTSAPCTITGTMGHFILARCPKGETLTVGFTDSRKISHSCTHPFHHDPPVIGREKFHSRPQHGKELPCSTYVQSTAATTEEIEVHMPPDTPDRTLMSQQSGNVKITVNGQTVRYKCNCGGSNEGLITTDKVINNCKVDQCHAAVTNHKKWQYNSPLVPRNAELGDRKGKIHIPFPLANVTCRVPKARNPTVTYGKNQVIMLLYPDHPTLLSYRNMGEEPNYQEEWVTHKKEVVLTVPTEGLEVTWGNNEPYKYWPQLSTNGTAHGHPHEIILYYYELYPTKTVVVVSVASFVLLSMVGVAVGMCMCARRRCITPYELTPGATVPFLLSLICCIRTAKAATYQEAAVYLWNEQQPLFWLQALIPLAALIVLCNCLRLLPCCCKTLAFLA

>ADG95954.1:1-798 structural polyprotein [Chikungunya virus]MDFIPTQTFYNRRYQPRPWTPRPTIQVIRPRPRPQRQAGQLAQLISAVNKLTMRAVPQQKPRRNRKNKKQKQKQQAPQNNTNQKKQPPKKKPAQKKKKPGRRERMCMKIENDCIFEVKHEGKVTGYACLVGDKVMKPAHVKGTIDNADLAKLAFKRSSKYDLECAQIPVHMKSDASKFTHEKPEGYYNWHHGAVQYSGGRFTIPTGAGKPGDSGRPIFDNKGRVVAIVLGGANEGARTALSVVTWNKDIVTKITPEGAEEWSLAIPVMCLLANTTFPCSQPPCTPCCYEKEPEETLRMLEDNVMRPGYYQLLQASLTCSPHRQRRSTKDNFNVYKATRPYLAHCPDCGEGHSCHSPVALERIRNEATDGTLKIQVSLQIGIKTDDSHDWTKLRYMDNHMPADAERAGLFVRTSAPCTITGTMGHFILARCPKGETLTVGFTDGRKISHSCTHPFHHDPPVIGREKFHSRPQHGKELPCSTYVQSTAATTEEIEVHMPPDTPDRTLMSQQSGNVKITVNGQTVRYKCNCGGSNEGLITTDKVINNCKVDQCHAAVTNHKKWQYNSPLVPRNAELGDRKGKIHIPFPLANVTCRVPKARNPTVTYGKNQVIMLLYPDHPTLLSYRNMGEEPNYQEEWVTHKKEVVLTVPTEGLEVTWGNNEPYKYWPQLSTNGTAHGHPHEIILYYYELYPTMTVVVVSVASFVLLSMVGMAVGMCMCARRRCITPYELTPGATVPFLLSLICCIRTTKAATYQEAAVYLWNEQQPLFWLQALIPLAALIVLCNCLRLLPCCCKTLAFLA

>APP91301.1:1-798 structural polyprotein [Chikungunya virus]MEFIPTQTFYNRRYQPRPWTPRPTIQVIRPRPRPQRQAGQLAQLISAVNKLTMRAVPQQKPRRNRKNKKQKQKQQAPQNNTNQKKQPPKKKPAQKKKKPGRRERMCMKIENDCIFEVKHEGKVTGYACLVGDKVMKPAHVKGTIDNADLAKLAFKRSSKYDLECAQIPVHMKSDASKFTHEKPEGYYNWHHGAVQYSGGRFTIPTGAGKPGDSGRPIFDNKGRVVAIVLGGANEGARTALSVVTWNKDIVTKITPEGAEEWSLAIPVMCLLANTTFPCSQPPCTPCCYEKEPEETLRMLEDNVMRPGYYQLLQASLTCSPHRQRRSTKDNFNVYKATRPYLAHCPDCGEGHSCHSPVALERIRNEATDGTLKIQVSLQIGIKTDDSHDWTKLRYMDNHMPADAERAGLFVRTSAPCTITGTMGHFILARCPKGETLTVGFTDGRKISHSCTHPFHHDPPVIGREKFHSRPQHGKELPCSTYVQSTAATTEEIEVHMPPDTPDRTLMSQQSGNVKITVNGQTVRYKCNCGGSNEGLITTDKVINNCKVDQCHAAVTNHKKWQYNSPLVPRNAELGDRKGKIYIPFPLANVTCRVPKARNPTVTYGKNQVIMLLYPDHPTLLSYRNMGEEPNYQEEWVTHKKEVVLTVPTEGLEVTWGNNEPYKYWPQLSTNGTAHGHPHEIILYYYELYPTMTVVVVSVASFVLLSMVGMAVGMCMCARRRCITPYELTPGATVPFLLSLICCIRTTKAATYQEAAVYLWNEQQPLFWLQALIPLAALIVLCNCLRLLPCCCKTLAFLA

>AQX78127.1:1-798 structural polyprotein [Chikungunya virus]MEFIPTQTFYNRRYQPRPWTPRPTIQVIRPRPRPQRQAGQLAQLISAVNKLTMRAVPQQKPRRNRKNKKQKQKQQAPQNNTNQKKQPPKKKPAQKKKKPGRRERMCMKIENDCIFEVKHEGKVTGYACLVGDKVMKPAHVKGTIDNADLAKLAFKRSSKYDLECAQIPVHMKSDASKFTHEKPEGYYNWHHGAVQYSGGRFTIPTGAGKPGDSGRPIFDNKGRVVAIVLGGANEGARTALSVVTWNKDIVTKITPEGAEEWSLAIPVMCLLANTTFPCSQPPCTPCCYEKEPEETLRMLEDNVMRPGYYQLLQASLTCSPHRQRRSTKDNFNVYKATRPYLAHCPDCGEGHSCHSPVALERIRNEATDGTLKIQVSLQIGIKTDDSHDWTKLRYMDNHMPADAERAGLFVRTSAPCMITGTMGHFILARCPKGETLTVGFTDSRKISHSCTHPFHHDPPVIGREKFHSRPQHGRELPCSTYVQSTAATTEEIEVHMPPDTPDRTLMSQQSGNVKITVNGQTVRYKCNCGGSNEGLITTDKVINNCKIDQCHAAVTNHKKWQYNSPLVPRNAELGDRKGKIHIPFPLANVTCRVPKARNPTVTYGKNQVIMLLYPDHPTLLSYRNMGEEPNYQEEWVTHKKEVVLTVPTEGLEVTWGNNEPYKYWPQLSTNGTAHGHPHEIILYYYELYPTMTVVVVSVASFVLLSMVGVAVGMCMCARRRCITPYELTPGATVPFLLSLICCIRTAKAATYQEAAVYLWNEQQPLFWLQALIPLAALIVLCNCLRLLPCCCKTLAFLA

>AGX45492.1:1-798 structural polyprotein [Chikungunya virus]MEFIPTQTFYNRRYQPRPWTPRPTIQVIRPRPRPQRQAGQLAQLISAVNKLTMRAVPQQKPRRNRKNKKQKQKQQAPQNNTNQKKQPPKKKPAQKKKKPGRRERMCMKIENDCIFEVKHEGKVTGYACLVGDKVMKPAHVKGTIDNADLAKLAFKRSSKYDLECAQIPVHMKSDASKFTHEKPEGYYNWHHGAVQYSGGRFTIPTGAGKPGDSGRPIFDNKGRVVAIVLGGANEGARTALSVVTWNKDIVTKITPEGAEEWSLAIPVMCLLANTTFPCSQPPCTPCCYEKEPEETLRMLEDNVMRPGYYQLLQASLTCSPHRQRRSTKDNFNVYKATRPYLAHCPDCGEGHSCHSPVALERIRNEATDGTLKIQVSLQIGIKTDDSHDWTKLRYMDNHMPADAERAGLFVRTSAPCTITGTMGHFILARCPKGETLTVGFTDSRKISHSCTHPFHHDPPVIGREKFHSRPQHGKELPCSTYVQSTAATTEEIEVHMPPDTPDRTLMSQQSGNVKITVNGQTVRYKCNCGGSNEGLTTTDKVINNCKVDQCHAAVTNHKKWQYNSPLVPRNAELGDRKGKIHIPFPLANVTCRVPKARNPTVTYGKNQVIMLLYPDHPTLLSYRNMGEEPNYQEEWVMHKKEVVLTVPTEGLEVTWGNNEPYKYWPQLSTNGTAHGHPHEIILYYYELYPTMTVVVVSVATFILLSMVGMAAGMCMCARRRCITPYELTPGATVPFLLSLICCIRTAKAATYQEAAIYLWNEQQPLFWLQALIPLAALIVLCNCLRLLPCCCKTLAFLA

>BBC18142.1:1-798 structural polyprotein [Chikungunya virus]MEFIPTQTFYNRRYQPRPWTPRPTIQVIRPRPRPQRQAGQLAQLISAVNKLTMRAVPQQKPRRNRKNKKQKQKQQAPQNNTNQKKQPPKKKPAPKKKKPGRRERMCMKIENDCIFEVKHEGKVTGYACLVGDKVMKPAHVKGTIDNADLAKLAFKRSSKYDLECAQIPVHMKSDASKFTHEKPEGYYNWHHGAVQYSGGRFTIPTGAGKPGDSGRPIFDNKGRVVAIVLGGANEGARTALSVVTWNKDIVTKITPEGAEEWSLAIPVMCLLANTTFPCSQPPCTPCCYEKEPEETLRMLEDNVMRPGYYQLLQASLTCSPHRQRRSTKDNFNVYKATRPYLAHCPDCGEGHSCHSPVALERIRNEATDGTLKIQVSLQIGIKTDDSHDWTKLRYMDNHVPADAERAGLFVRTSAPCTITGTMGHFILARCPKGETLTVGFTDGRKISHSCTHPFHHDPPVIGREKFHSRPQHGKELPCSTYVQSTAATTEEIEVHMPPDTPDRTLMSQQSGNVKITVNGQTVRYKCNCGGSNEGLITTDKVINNCKVDQCHAAVTNHKKWQYNSPLVPRNAELGDRKGKIHIPFPLANVTCRVPKARNPTVTYGKNQVIMLLYPDHPTLLSYRNMGEEPNYQEEWVTHKKEVVLTVPTEGLEVTWGNNEPYKYWPQLSTNGTAHGHPHEIILYYYELYPTTTVVVVSVASFVLLSMVGMAVGMCMCARRRCITPYELTPGATVPFLLSLICCIRTAKAATYQEAAVYLWNEQQPLFWLQALIPLAALIVLCNCLRLLPCCCKTLAFLA

>ABD95938.1:1-798 structural polyprotein [Chikungunya virus]MEFIPTQTFYNRRYQPRPWTPRPTIQVIRPRPRPQRQAGQLAQLISAVNKLTMRAVPQQKPRRNRKNKKQKQKQQAPQNNTNQKKQPPKKKPAQKKKKPGRRERMCMKIENDCIFEVKHEGKVTGYACLVGDKVMKPAHVKGTIDNADLAKLAFKRSSKYDLECAQIPVHMKSDASKFTHEKPEGYYNWHHGAVQYSGGRFTIPTGAGKPGDSGRPIFDNKGRVVAIVLGGANEGARTALSVVTWNKDIVTKITPEGAEEWSLAIPVMCLLANTTFPCSQPPCTPCCYEKEPEETLRMLEDNVMRPGYYQLLQASLTCSPHRQRRSTKDNFNVYKATRPYLAHCPDCGEGHSCHSPVALERIRNEATDGTLKIQVSLQIGIKTDDSHDWTKLRYMDNHMPADAERAGLFVRTSAPCTITGTMGHFILARCPKGETLTVGFTDSRKISHSCTHPFHHDPPVIGREKFHSRPQHGKELPCSTYVQSTAATTEEIEVHMPPDTPDRTLMSQQSGNVKITVNGQTVRYKCNCGGSNEGLTTTDKVINNCKVDQCHAAVTNHKKWQYNSPLVPRNAELGDRKGKIHIPFPLANVTCRVPKARNPTVTYGKNQVIMLLYPDHPTLLSYRNMGEEPNYQEEWVMHKKEVVLTVPTEGLEVTWGNNEPYKYWPQLSTNGTAHGHPHEIILYYYELYPTMTVVVVSVATFILLSMVGMAAGMCMCARRRCITPYELTPGATVPFLLSLICCIRTAKAATYQEAAIYLWNEQQPLFWLQALIPLAALIVLCNCLRLLPCCCKTLAFLA

>APP91303.1:1-798 structural polyprotein [Chikungunya virus]MEFIPTQTFYNRRYQPRPWTPRPTIQVIRPRPRPQRQAGQLAQLISAVNKLTMRAVPQQKPRRNRKNKKQKQKQQAPQNNTNQKKQPPKKKPAPKKKKPGRRERMCMKIENDCIFEVKHEGKVTGYACLVGDKVMKPAHVKGTIDNADLAKLAFKRSSKYDLECAQIPVHMKSDASKFTHEKPEGYYNWHHGAVQYSGGRFTIPTGAGKPGDSGRPIFDNKGRVVAIVLGGANEGARTALSVVTWNKDIVTKITPEGAEEWSLAIPVMCLLANTTFPCSQPPCTPCCYEKEPEETLRMLEDNVMRPGYYQLLQASLTCSPHRQRRGTKDNFNVYKATRPYLAHCPDCGEGHSCHSPVALERIRNEATDGTLKIQVSLQIGIKTDDSHDWTKLRYMDNHMPADAERAGLFVRTSAPCTITGTMGHFILARCPKGETLTVGFTDGRKISHSCTHPFHHDPPVIGREKFHSRPQHGKELPCSTYVQSTAATTEEIEVHMPPDTPDRTLMSQQSGNVKITVNGQTVRYKCNCGGSNEGLITTDKVINNCKVDQCHAAVTNHKKWQYNSPLVPRNAELGDRKGKIHIPFPLANVTCRVPKARNPTVTYGKNQVIMLLYPDHPTLLSYRNMGEEPNYQEEWVTHKKEVVLTVPTEGLEVTWGNNEPYKYWPQLSTNGTAHGHPHEIILYYYELYPTMTVVVVSVASFVLLSMVGMAVGMCMCARRRCITPYELTPGATVPFLLSLICCIRTAKAATYQEAAVYLWNEQQPLFWLQALIPLAALIVLCNCLRLLPCCCKTLAFLA

>ADG95934.1:1-798 structural polyprotein [Chikungunya virus]MEFIPTQTFYNRRYQPRPWTPRPTIQVIRPRPRPQRQAGQLAQLISAVNKLTMRAVPQQKPRRNRKNKKQKQKQQAPQNNTNQKKQPPKKKPAQKKKKPGRRERMCMKIENDCIFEVKHEGKVTGYACLVGDKVMKPAHVKGTIDNADLAKLAFKRSSKYDLECAQIPVHMKSDASKFTHEKPEGYYNWHHGAVQYSGGRFTIPTGAGKPGDSGRPIFDNKGRVVAIVLGGANEGARTALSVVTWNKDIVTKITPEGAEEWSLAIPVMCLLANTTFPCSQPPCTPCCYEKEPEETLRMLEDNVMRPGYYQLLQASLTCSPHRQRRSTKDNFNVYKATRPYLAHCPDCGEGHSCHSPVALERIRNEATDGTLKIQVSLQIGIKTDGSHDWTKLRYMDNHMPADAERAGLFVRTSAPCTITGTMGHFILARCPKGETLTVGFTDSRKISHSCTHPFHHDPPVIGREKFHSRPQHGKELPCSTYVQSTAVTTEEIEVHMPPDTPDRTLMSQQSGNVKITVNGQTVRYKCNCGGSNEGLITTDKVINNCKVDQCHAAVTNHKKWQYNSPLVPRNAELGDRKGKIHIPFPLANVTCRVPKARNPTVTYGKNQVIMLLYPDHPTLLSYRNMGEEPNYQEEWVTHKKEVMLTVPTEGLEVTWGNNEPYKYWPQLSTNGTAHGHPHEIILYYYELYPTMTVVVVSVASFVLLSMVGMAVGMCMCARRRCITPYELTPGATVPFLLSLICCIRTAKAATYQEAAVYLWNEQQPLFWLQALIPLAALIVLCNCLRLLPCCCKTLAFLA

>ABN04188.1:1-798 structural polyprotein [Chikungunya virus]MEFIPTQTFYNRRYQPRPWTPRSTIQVIRPRPRPQRQAGQLAQLISAVNKLTMRAVPQQKPRRNRKNKKQKQKQQAPQNNTNQKKQPPKKKPAQKKKKPGRRERMCMKIENDCIFEVKHEGKVTGYACLVGDKVMKPAHVKGTIDNADLAKLAFKRSSKYDLECAQIPVHMKSDASKFTHEKPEGYYNWHHGAVQYSGGRFTIPTGAGKPGDSGRPIFDNKGRVVAIVLGGANEGARTALSVVTWNKDIVTKITPEGAEEWSLAIPVMCLLANTTFPCSQPPCTPCCYEKEPEETLRMLEDNVMRPGYYQLLQASLTCSPHRQRRSTKDNFNVYKATRPYLAHCPDCGEGHSCHSPVALERIRNEATDGTLKIQVSLQIGIKTDDSHDWTKLRYMDNHMPADAERAGLFVRTSAPCTITGTMGHFILARCPKGETLTVGFTDSRKISHSCTHPFHHDPPVIGREKFHSRPQHGKELPCSTYVQSTAATTEEIEVHMPPDTPDRTLMSQQSGNVKITVNGQTVRYKCNCGGSNEGLTTTDKVINNCKVDQCHAAVTNHKKWQYNSPLVPRNAELGDRKGKIHIPFPLANVTCRVPKARNPTVTYGKNQVIMLLYPDHPTLLSYRNMGEEPNYQEEWVMHKKEVVLTVPTEGLEVTWGNNEPYKYWPQLSTNGTAHGHPHEIILYYYELYPTMTVVVVSVATFILLSMVGMAAGMCMCARRRCITPYELTPGATVPFLLSLICCIRTAKAATYQEAAIYLWNEQQPLFWLQALIPLAALIVLCNCLRLLPCCCKTLAFLA

>ADG95921.1:1-798 structural polyprotein [Chikungunya virus]MEFIPTQTFYNRRYQPRPWTPRPTIQVIRPRPRPQRQAGQLAQLISAVNKLTMRAVPQQKPRRNRKNKKQKQKQQAPQNNTNQKKQPPKKKPAQKKKKPGRRERMCMKIENDCIFEVKHEGKVTGYACLVGDKVMKPAHVKGTIDNADLAKLAFKRSSKYDLECAQIPVHMKSDASKFTHEKPEGYYNWHHGAVQYSGGRFTIPTGAGKPGDSGRPIFDNKGRVVAIVLGGANEGARTALSVVTWNKDIVTKITPEGAEEWSLAIPVMCLLANTTFPCSQPPCTPCCYEKEPEETLRMLEDNVMRPGYYQLLQASLTCSPHRQRRSTKDNFNVYKATRPYLAHCPDCGEGHSCHSPVALERIRNEATDGTLKIQVSLQIGIKTDDSHDWTKLRYMDNHMPADAERAGLFVRTSAPCTITGTMGHFILARCPKGETLTVGFTDSRKISHSCTHPFHHDPPVIGREKFHSRPQHGKELPCSTXVQNTAATAEEIEVHMPPDTPDRTLMSQQSGNVKITVNGQTVRYKCNCGGSNEGLTTTDKVINNCKVDQCHAAVTNHKKWQYNSPLVPRNAELGDRKGKIHIPFPLANXTCRVPKARNPTVTYGKNQVIMLLYPDHPTLLSYRNMGEEPNYQEEWVTYKKEVVLTVPTEGLEVTWGNNEPYKYWPQLSANGTAHGHPHEIILYYYELYPTMTVVVVSVASFILLSMVGMAVGMCMCARRRCITPYELTPGATVPFLLSLICCFRTAKAATYQEAAVYLWNEQQPLFWLQALIPLAALIVLCNCLRLLPCCCKTLAFLA

>ACZ72971.1:1-798 structural polyprotein [Chikungunya virus]MEFIPTQTFYNRRYQPRPWTPRSTIQIIRPRPRPQRQAGQLAQLISAVNKLTMRAVPQQKPRRNRKNKKQKQKQQAPQNNTNQKKQPPKKKPAQKKKKPGRRERMCMKIENDCIFEVKHEGKVTGYACLVGDKVMKPAHVKGTIDNADLAKLAFKRSSKYDLECAQIPVHMKSDASKFTHEKPEGYYNWHHGAVQYSGGRFTIPTGAGKPGDSGRPIFDNKGRVVAIVLGGANEGARTALSVVTWNKDIVTKITPEGAEEWSLAIPVMCLLANTTFPCSQPPCTPCCYEKEPEETLRMLEDNVMRPGYYQLLQASLTCSPHRQRRSTKDNFNVYKATRPYLAHCPDCGEGHSCHSPVALERIRNEATDGTLKIQVSLQIGIKTDDSHDWTKLRYMDNHMPADAERAGLFVRTSAPCTITGTMGHFILARCPKGETLTVGFTDSRKISHSCTHPFHHDPPVIGREKFHSRPQHGKELPCSTYVQSTAATTEEIEVHMPPDTPDRTLMSQQSGNVKITVNGQTVRYKCNCGGSNEGLTTTDKVINNCKVDQCHAAVTNHKKWQYNSPLVPRNAELGDRKGKIHIPFPLANVTCRVPKARNPTVTYGKNQVIMLLYPDHPTLLSYRNMGEEPNYQEEWVMHKKEVVLTVPTEGLEVTWGNNEPYKYWPQLSTNGTAHGHPHEIILYYYELYPTMTVVVVSVATFILLSMVGMAVGMCMCARRRCITPYELTPGATVPFLLSLICCIRTAKAATYQEAAIYLWNEQQPLFWLQALIPLAALIVLCNCLRLLPCCCKTLAFLA

>ACA81773.1:1-798 structural polyprotein [Chikungunya virus]MEFIPTQTFYNRRYQPRPWTPRSTIQVIRPRPRPQRQAGQLAQLISAVNKLTMRAVPQQKPRRNRKNKKQKQKQQAPQNNTNQKKQPPKKKPAQKKKKPGRRERMCMKIENDCIFEVKHEGKVTGYACLVGDKVMKPAHVKGTIDNADLAKLAFKRSSKYDLECAQIPVHMKSDASKFTHEKPEGYYNWHHGAVQYSGGRFTIPTGAGKPGDSGRPIFDNKGRVVAIVLGGANEGARTALSVVTWNKDIVTKITPEGAEEWSLAIPVMCLLANTTFPCSQPPCTPCCYEKEPEETLRMLEDNVMRPGYYQLLQASLTCSPHRQRRSTKDNFNVYKATRPYLAHCPDCGEGHSCHSPVALERIRNEATDGTLKIQVSLQIGIKTDDSHDWTKLRYMDNHMPADAERAGLFVRTSAPCTITGTMGHFILARCPKGETLTVGFTDSRKISHSCTHPFHHDPPVIGREKFHSRPQHGKELPCSTYVQSTAATTEEIEVHMPPDTPDRTLMSQQSGNVKITVNGQTVRYKCNCGGSNEGLTTTDKVINNCKVDQCHAAVTNHKKWQYNSPLVPRNAELGDRKGKIHIPFPLANVTCRVPKARNPTVTYGKNQVIMLLYPDHPTLLSYRNMGEEPNYQEEWVMHKKEVVLTVPTEGLEVTWGNNEPYKYWPQLSTNGTAHGHPHEIILYYYELYPTMTVVVVSVATFILLSMVGMAAGMCMCARRRCITPYELTPGATVPFLLSLICCIRTAKAATYQEAAIYLWNEQQPLFWLQALIPLAALIVLCNCLRLLPCCCKTLAFLA

>CAJ90473.1:1-798 structural polyprotein [Chikungunya virus]MEFIPTQTFYNRRYQPRPWTPRPTIQVIRPRPRPQRQAGQLAQLISAVNKLTMRAVPQQKPRRNRKNKKQKQKQQAPQNNTNQKKQPPKKKPAQKKKKPGRRERMCMKIENDCIFEVKHEGKVTGYACLVGDKVMKPAHVKGTIDNADLAKLAFKRSSKYDLECAQIPVHMKSDASKFTHEKPEGYYNWHHGAVQYSGGRFTIPTGAGKPGDSGRPIFDNKGRVVAIVLGGANEGARTALSVVTWNKDIVTKITPEGAEEWSLAIPVMCLLANTTFPCSQPPCTPCCYEKEPEETLRMLEDNVMRPGYYQLLQASLTCSPHRQRRSTKDNFNVYKATRPYLAHCPDCGEGHSCHSPVALERIRNEATDGTLKIQVSLQIGIKTDDSHDWTKLRYMDNHMPADAERAGLFVRTSAPCTITGTMGHFILARCPKGETLTVGFTDSRKISHSCTHPFHHDPPVIGREKFHSRPRHGKELPCSTYVQSTAATTEEIEVHMPPDTPDRTLMSQQSGNVKITVNGQTVRYKCNCGGSNEGLTTTDKVINNCKVDQCHAAVTNHKKWQYNSPLVPRNAELGDRKGKIHIPFPLANVTCRVPKARNPTVTYGKNQVIMLLYPDHPTLLSYRNMGEEPNYQEEWVMHKKEVVLTVPTEGLEVTWGNNEPYKYWPQLSTNGTAHGHPHEIILYYYELYPTMTVVVVSVATFILLSMVGMAAGMCMCARRRCITPYELTPGATVPFLLSLICCIRTAKAATYQEAAIYLWNEQQPLFWLQALIPLAALIVLCNCLRLLPCCCKTLAFLA

>ADG95928.1:1-798 structural polyprotein [Chikungunya virus]MEFIPTQTFYNRRYQPRPWTPRPTIQVIRPRPRPQRQAGQLAQLISAVNKLTMRAVPQQKPRRNRKNKKQKQKQQAPQNNTNQKKQPPKKKPAQKKKKPGRRERMCMKIENDCIFEVKHEGKVTGYACLVGDKVMKPAHVKGTIDNADLAKLAFKRSSKYDLECAQIPVHMKSDASKFTHEKPEGYYNWHHGAVQYSGGRFTIPTGAGKPGDSGRPIFDNKGRVVAIVLGGANEGARTALSVVTWNKDIVTKITPEGAEEWSLAIPVMCLLANTTFPCSQPPCTPCCYEKEPEGTLRMLEDNVMRPGYYQLLQASLTCSPHRQRRSTKDNFNVYKATRPYLAHCPDCGEGHSCHSPVALERIRNEATDGTLKIQVSLQIGIKTDDSHDWTKLRYMDNHMPADAERAGLFVRTSAPCTITGTMGHFILARCPKGETLTVGFTDSRKISHSCTHPFHHDPPVIGREKFHSRPQHGKELPCSTYVQSTAATAEEIEVHMPPDTPDRTLMSQQSGNVKITVNGQTVRYKCNCGGSNEGLITTDKVINNCKVDQCHAAVTNHKKWQYNSPLVPRNAELGDRKGKIHIPFPLANVTCRVPKARNPTVTYGKNQVIMLLYPDHPTLLSYRNMGEKPNYQEEWVTHKKEVVLTVPTEGLEVTWGNNEPYKYWPQLSTNGTAHGHPHEIILYYYELYPTTTVVVVSVASFILLSMVGTAVGMCMCARRRCITPYELTPGATVPFLLSLICCIRTAKAATYQEAAVYLWNEQQPLFWLQALIPLAALIVLCNCLRLLPCCCKTLAFLA

>QHB74132.1:1-798 structural polyprotein [Chikungunya virus]MEFIPTQTFYNRRYQPRPWTPRSTIQIIRPRPRPQRQAGQLAQLISAVNKLTMRAVPQQKPRRNRKNKKQKQKQQAPQNNTNQKKQPPKKKPAQKKKKPGRRERMCMKIENDCIFEVKHEGKVTGYACLVGDKVMKPAHVKGTIDNADLAKLAFKRSSKYDLECAQIPVHMKSDASKFTHEKPEGYYNWHHGAVQYSGGRFTIPTGAGKPGDSGRPIFDNKGRVVAIVLGGANEGARTALSVVTWNKDIVTKITPEGAEEWSLAIPVMCLLANTTFPCSQPPCTPCCYEKEPEETLRMLEDNVMRPGYYQLLQASLTCSPHRQRRSTKDNFNVYKATRPYLAHCPDCGEGHSCHSPVALERIRNEATDGTLKIQVSLQIGIKTDDSHDWTKLRYMDNHMPADAERAGLFVRTSAPCTITGTMGHFILARCPKGETLTVGFTDSRKISHSCTHPFHHDPPVIGREKFHSRPQHGKELPCSTYVQSTAATAEEIEVHMPPDTPDRTLLSQQSGNVKITVNGQTVRYKCNCGGSNEGLTTTDKVINNCKVDQCHAAVTNHKKWQYNSPLVPRNAELGDRKGKIHIPFPLANATCRVPKARNPTVTYGKNQVIMLLYPDHPTLLSYRNMGEEPNYQEEWVMHKKEVVLTVPTEGLEVTWGNNEPYKYWPQLSTNGTAHGHPHEIILYYYELYPTMTVVVVSVATFILLSMVGMAAGMCMCARRRCITPYELTPGATVPFLLSLICCIRTAKAATYQEAAIYLWNEQQPLFWLQALIPLAALIVLCNCLRLLPCCCKTLAFLA

>ABN04192.1:1-798 structural polyprotein [Chikungunya virus]MEFIPTQTFYNRRYQPRPWTPRSTIQIIRPRPRPQRQAGQLAQLISAVNKLTMRAVPQQKPRRNRKNKKQKQKQQAPQNNTNQKKQPPKKKPAQKKKKPGRRERMCMKIENDCIFEVKHEGKVTGYACLVGDKVMKPAHVKGTIDNADLAKLAFKRSSKYDLECAQIPVHMKSDASKFTHEKPEGYYNWHHGAVQYSGGRFTIPTGAGKPGDSGRPIFDNKGRVVAIVLGGANEGARTALSVVTWNKDIVTKITPEGAEEWSLAIPVMCLLANTTFPCSQPPCTPCCYEKEPEETLRMLEDNVMRPGYYQLLQASLTCSPHRQRRSTKDNFNVYKATRPYLAHCPDCGEGHSCHSPVALERIRNEATDGTLKIQVSLQIGIKTDDSHDWTKLRYMDNHMPADAERAGLFVRTSAPCTITGTMGHFILARCPKGETLTVGFTDSRKISHSCTHPFHHDPPVIGREKFHSRPQHGKELPCSTYVQSTAATTEEIEVHMPPDTPDRTLMSQQSGNVKITVNGQTVRYKCNCGGSNEGLTTTDKVINNCKVDQCHAAVTNHKKWQYNSPLVPRNAELGDRKGKIHIPFPLANVTCRVPKARNPTVTYGKNQVIMLLYPDHPTLLSYRNMGEEPNYQEEWVMHKKEVVLTVPTEGLEVTWGNNEPYKYWPQLSTNGTAHGHPHEIILYYYELYPTMTVVVVSVATFILLSMVGMAAGMCMCARRRCITPYELTPGATVPFLLSLICCIRTAKAATYQEAAIYLWNEQQPLFWLQALIPLAALIVLCNCLRLLPCCCKTLAFLA

>BAH97933.1:1-798 structural polyprotein [Chikungunya virus]MEFIPTQTFYNRRYQPRPWTPRSTIQIIRPRPRPQRQAGQLAQLISAVNKLTMRAVPQQKPRRNRKNKKQKQKQQAPQNNTNQKKQPPKKKPAQKKKKPGRRERMCMKIENDCIFEVKHEGKVTGYACLVGDKVMKPAHVKGTIDNADLAKLAFKRSSKYDLECAQIPVHMKSDASKFTHEKPEGYYNWHHGAVQYSGGRFTIPTGAGKPGDSGRPIFDNKGRVVAIVLGGANEGARTALSVVTWNKDIVTKITPEGAEEWSLAIPVMCLLANTTFPCSQPPCTPCCYEKEPEETLRMLEDNVMRPGYYQLLQASLTCSPHRQRRSTKDNFNVYKATRPYLAHCPDCGEGHSCHSPVALERIRNEATDGTLKIQVSLQIGIKTDDSHDWTKLRYMDNHMPADAERAGLFVRTSAPCTITGTMGHFILARCPKGETLTVGFTDSRKISHSCTHPFHHDPPVIGREKFHSRPQHGKELPCSTYVQSTAATTEEIEVHMPPDTPDRTLMSQQSGNVKITVNGQTVRYKCNCGGSNEGLTTTDKVINNCKVDQCHAAVTNHKKWQYNSPLVPRNAELGDRKGKIHIPFPLANVTCRVPKARNPTVTYGKNQVIMLLYPDHPTLLSYRNMGEEPNYQEEWVMHKKEVVLTVPTEGLEVTWGNNEPYKYWPQLSTNGTAHGHPHEIILYYYELYPTMTVVVVSVATFILLSMVGMAAGMCMCARRRCITPYELTPGATVPFLLSLICCIRTAKAATYQEAAIYLWNEQQPLFWLQALIPLAALIVLCNCLRLLPCCCKTLAFLA

>ABX38965.1:1-798 structural polyprotein [Chikungunya virus]MEFIPTQTFYNRRYQPRPWTPRSTIQIIRPRPRPQRQAGQLAQLISAVNKLTMRAVPQQKPRRNRKNKKQKQKQQAPQNNTNQKKQPPKKKPAQKKKKPGRRERMCMKIENDCIFEVKHEGKVTGYACLVGDKVMKPAHVKGTIDNADLAKLAFKRSSKYDLECAQIPVHMKSDASKFTHEKPEGYYNWHHGAVQYSGGRFTIPTGAGKPGDSGRPIFDNKGRVVAIVLGGANEGARTALSVVTWNKDIVTKITPEGAEEWSLAIPVMCLLANTTFPCSQPPCTPCCYEKEPEETLRMLEDNVMRPGYYQLLQASLTCSPHRQRRSTKDNFNVYKATRPYLAHCPDCGEGHSCHSPVALERIRNEATDGTLKIQVSLQIGIKTDDSHDWTKLRYMDNHMPADAERAGLFVRTSAPCTITGTMGHFILARCPKGETLTVGFTDSRKISHSCTHPFHHDPPVIGREKFHSRPQHGKELPCSTYVQSTAATTEEIEVHMPPDTPDRTLMSQQSGNVKITVNGQTVRYKCNCGGSNEGLTTTDKVINNCKVDQCHAAVTNHKKWQYNSPLVPRNAELGDRKGKIHIPFPLANVTCRVPKARNPTVTYGKNQVIMLLYPDHPTLLSYRNMGEEPNYQEEWVMHKKEVVLTVPTEGLEVTWGNNEPYKYWPQLSTNGTAHGHPHEIILYYYELYPTMTVVVVSVATFILLSMVGMAAGMCMCARRRCITPYELTPGATVPFLLSLICCIRTAKAATYQEAAIYLWNEQQPLFWLQALIPLAALIVLCNCLRLLPCCCKTLAFLA

>AEE60791.1:1-798 structural polyprotein [Chikungunya virus]MEFIPTQTFYNRRYQPRPWTPRSTIQIIRPRPRPQRQAGQLAQLISAVNKLTMRAVPQQKPRRNRKNKKQKQKQQAPQNNTNQKKQPPKKKPAQKKKKPGRRERMCMKIENDCIFEVKHEGKVTGYACLVGDKVMKPAHVKGTIDNADLAKLAFKRSSKYDLECAQIPVHMKSDASKFTHEKPEGYYNWHHGAVQYSGGRFTIPTGAGKPGDSGRPIFDNKGRVVAIVLGGANEGARTALSVVTWNKDIVTKITPEGAEEWSLAIPVMCLLANTTFPCSQPPCTPCCYEKEPEETLRMLEDNVMRPGYYQLLQASLTCSPHRQRRSTKDNFNVYKATRPYLAHCPDCGEGHSCHSPVALERIRNEATDGTLKIQVSLQIGIKTDDSHDWTKLRYMDNHMPADAERAGLFVRTSAPCTITGTMGHFILARCPKGETLTVGFTDSRKISHSCTHPFHHDPPVIGREKFHSRPQHGKELPCSTYVQSTAATTEEIEVHMPPDTPDRTLMSQQSGNVKITVNGQTVRYKCNCGGSNEGLTTTDKVINNCKVDQCHAAVTNHKKWQYNSPLVPRNAELGDRKGKIHIPFPLANVTCRVPKARNPTVTYGKNQVIMLLYPDHPTLLSYRNMGEEPNYQEEWVMHKKEVVLTVPTEGLEVTWGNNEPYKYWPQLSTNGTAHGHPHEIILYYYELYPTMTVVVVSVATFILLSMVGMAAGMCMCARRRCITPYELTPGATVPFLLSLICCIRTAKAATYQEAAIYLWNEQQPLFWLQALIPLAALIVLCNCLRLLPCCCKTLAFLA

>ACZ98837.1:1-798 structural polyprotein [Chikungunya virus]MEFIPTQTFYNRRYQPRPWTPRSTIQVIRPRPRPQRQAGQLAQLISAVNKLTMRAVPQQKPRRNRKNKKQKQKQQAPQNNTNQKKQPPKKKPAQKKKKPGRRERMCMKIENDCIFEVKHEGKVTGYACLVGDKVMKPAHVKGTIDNADLAKLAFKRSSKYDLECAQIPVHMKSDASKFTHEKPEGYYNWHHGAVQYSGGRFTIPTGAGKPGDSGRPIFDNKGRVVAIVLGGANEGARTALSVVTWNKDIVTKITPEGAEEWSLAIPVMCLLANTTFPCSQPPCTPCCYEKEPEETLRMLEDNVMRPGYYQLLQASLTCSPHRQRRSTKDNFNVYKATRPYLAHCPDCGEGHSCHSPVALERIRNEATDGTLKIQVSLQIGIKTDDSHDWTKLRYMDNHMPADAERAGLFVRTSAPCTITGTMGHFILARCPKGETLTVGFTDSRKISHSCTHPFHHDPPVIGREKFHSRPQHGKELPCSTYVQSTAATTEEIEVHMPPDTPDRTLMSQQSGNVKITVNGQTVRYKCNCGGSNEGLTTTDKVINNCKVDQCHAAVTNHKKWQYNSPLVPRNAELGDRKGKIHIPFPLANVTCRVPKARNPTVTYGKNQVIMLLYPDHPTLLSYRNMGEEPNYQEEWVMHKKEVVLTVPTEGLEVTWGNNEPYKYWPQLSTNGTAHGHPHEIILYYYELYPTMTVVVVSVATFILLSMVGMAAGMCMCARRRCITPYELTPGATVPFLLSLICCIRTAKAATYQEAAIYLWNEQQPLFWLQALIPLAALIVLCNCLRLLPCCCKTLAFLA

>AYI50323.1:1-798 structural polyprotein [Chikungunya virus]MEFIPTQTFYNRRYQPRPWTPRPTIQVIRPRPRPQRQAGQLAQLISAVNKLTMRAVPQQKPRRNRKNKKQKQKQQAPQNNTNQKKQPPKKKPAQKKKKPGRRERMCMKIENDCIFEVKHEGKVTGYACLVGDKVMKPAHVKGTIDNADLAKLAFKRSSKYDLECAQIPVHMKSDASKFTHEKPEGYYNWHHGAVQYSGGRFTIPTGAGKPGDSGRPIFDNKGRVVAIVLGGANEGARTALSVVTWNKDIVTKITPEGAEEWSLAIPVMCLLANTTFPCSQPPCTPCCYEKEPEETLRMLEDNVMRPGYYQLLQASLTCSPHRQRRSTKDNFNVYKATRPYLAHCPDCGEGHSCHSPVALERIRNEATDGTLKIQVSLQIGIKTDDSHDWTKLRYMDNHMPADAERAGLFVRTSAPCTITGTMGHFILARCPKGETLTVGFTDSRKISHSCTHPFHHDPPVIGREKFHSRPQHGKELPCSTYVQSTAATTEEIEVHMPPDTPDRTLMSQQSGNVKITVNGQTVRYKCNCGGSNEGLTTTDKVINNCKVDQCHAAVTNHKKWQYNSPLVPRNAELGDRKGKIHIPFPLANVTCRVPKARNPTVTYGKNQVIMLLYPDHPTLLSYRNMGEEPHYQEEWVMHKKEVVLTVPTEGLEVTWGNNEPYKYWPQLSTNGTAHGHPHEIILYYYELYPTMTVVVVSVATFILLSMVGMAAGMCMCARRRCITPYELTPGATVPFLLSLICCIRTAKAATYQEAAIYLWNEQQPLFWLQALIPLAALIVLCNCLRLLPCCCKTLAFLA

>ABN04190.1:1-798 structural polyprotein [Chikungunya virus]MEFIPTQTFYNRRYQPRPWTPRSTIQIIRPRPRPQRQAGQLAQLISAVNKLTMRAVPQQKPRRNRKNKKQKQKQQAPQNNTNQKKQPPKKKPAQKKKKPGRRERMCMKIENDCIFEVKHEGKVTGYACLVGDKVMKPAHVKGTIDNADLAKLAFKRSSKYDLECAQIPVHMKSDASKFTHEKPEGYYNWHHGAVQYSGGRFTIPTGAGKPGDSGRPIFDNKGRVVAIVLGGANEGARTALSVVTWNKDIVTKITPEGAEEWSLAIPVMCLLANTTFPCSQPPCTPCCYEKEPEETLRMLEDNVMRPGYYQLLQASLTCSPHRQRRSTKDNFNVYKATRPYLAHCPDCGEGHSCHSPVALERIRNEATDGTLKIQVSLQIGIKTDDSHDWTKLRYMDNHMPADAERAGLFVRTSAPCTITGTMGHFILARCPKGETLTVGFTDSRKISHSCTHPFHHDPPVIGREKFHSRPQHGKELPCSTYVQSTAATTEEIEVHMPPDTPDRTLMSQQSGNVKITVNGQTVRYKCNCGGSNEGLTTTDKVINNCKVDQCHAAVTNHKKWQYNSPLVPRNAELGDRKGKIHIPFPLANVTCRVPKARNPTVTYGKNQVIMLLYPDHPTLLSYRNMGEEPNYQEEWVMHKKEVVLTVPTEGLEVTWGNNEPYKYWPQLSTNGTAHGHPHEIILYYYELYPTMTVVVVSVATFILLSMVGMAAGMCMCARRRCITPYELTPGATVPFLLSLICCIRTAKAATYQEAAIYLWNEQQPLFWLQALIPLAALIVLCNCLRLLPCCCKTLAFLA

>AJD20052.1:1-798 structural polyprotein [Chikungunya virus]MEFIPTQTFYNRRYQPRPWTPRPTIQVIRPRPRPQRQAGQLAQLISAVNKLTMRAVPQQKPRRNRKNNKQKQKQQAPQNNTNQKKQPPKKKPAQKKKKPGRRERMCMKIENDCIFEVKHEGKVTGYACLVGDKVMKPAHVKGTIDNADLAKLAFKRSSKYDLECAQIPVHMKSDASKFTHEKPEGYYNWHHGAVQYSGGRFTIPTGAGKPGDSGRPIFDNKGRVVAIVLGGANEGARTALSVVTWNKDIVTKITPEGAEEWSLAIPVMCLLANTTFPCSQPPCTPCCYEKEPEETLRMLEDNVMRPGYYQLLQASLTCSPHRQRRSTKDNFNVYKATRPYLAHCPDCGEGHSCHSPVALERIRNEATDGTLKIQVSLQIGIKTDDSHDWTKLRYMDNHMPADAERAGLFVRTSAPCTITGTMGHFILARCPKGETLTVGFTDSRKISHSCTHPFHHDPPVIGREKFHSRPQHGKELPCSTYVQSTAATTEEIEVHMPPDTPDRTLMSQQSGNVKITVNGQTVRYKCNCGGSNEGLTTTDKVINNCKVDQCHAAVTNHKKWQYNSPLVPRNAELGDRKGKIHIPFPLANVTCRVPKARNPTVTYGKNQVIMLLYPDHPTLLSYRNMGEEPNYQEEWVMHKKEVVLTVPTEGLEVTWGNNEPYKYWPQLSTNGTAHGHPHEIILYYYELYPTMTVVVVSVATFILLSMVGMAAGMCMCARRRCITPYELTPGATVPFLLSLICCIRTAKAATYQEAAIYLWNEQQPLFWLQALIPLAALIVLCNCLRLLPCCCKTLAFLA

>AKN79949.1:1-798 structural polyprotein, partial [Chikungunya virus]MEFIPTQTFYNRRYQPRPWTPRPTIQVIRPRPRPQRQAGQLAQLISAVNKLTMRAVPQQKPRRNRKNKKQKQKQQAPQNNTNQKKQPPKKKPAQKKKKPGRRERMCMKIENDCIFEVKHEGKVTGYACLVGDKVMKPAHVKGTIDNADLAKLAFKRSSKYDLECAQIPVHMKSDASKFTHEKPEGYYNWHHGAVQYSGGRFTIPTGAGKPGDSGRPIFDNKGRVVAIVLGGANEGARTALSVVTWNKDIVTKITPEGAEEWSLAIPVMCLLANTTFPCSQPPCTPCCYEKEPEETLRMLEDNVMRPGYYQLLQASLTCSPHRQRRSTKDNFNVYKATRPYLAHCPDCGEGHSCHSPVALERIRNEATDGTLKIQVSLQIGIKTDDSHDWTKLRYMDNHMPADAERAGLFVRTSAPCTITGTMGHFILARCPKGETLTVGFTDSRKISHSCTHPFHHDPPVIGREKFHSRPQHGKELPCSTYVQSTAATTEEIEVHMPPDTPDRTLMSQQSGNVKITVNGQTVRYKCNCGGSNEGLTTTDKVINNCKVDQCHAAVTNHKKWQYNSPLVPRNAELGDRKGKIHIPFPLANVTCRVPKARNPTVTYGKNQVIMLLYPDHPTLLSYRNMGEEPNYQEEWVMHKKEVVLTVPTEGLEVTWGNNEPYKYWPQLSTNGTAHGHPHEIILYYYELYPTMTVVVVSVATFILLSMVGMAAGMCMCARRRCITPYELTPGATVPFLLSLICCIRTAKAATYQEAAIYLWNEQQPLFWLQALIPLAALIVLCNCLRLLPCCCKTLAFLA

>QHB74128.1:1-798 structural polyprotein [Chikungunya virus]MEFIPTQTFYNRRYQPRPWTPRSTIQIIRPRPRPQRQAGQLAQLISAVNKLTMRAVPQQKPRRNRKNKKQKQKQQAPQSNTNQKKQPPKKKPAQKKKKPGRRERMCMKIENDCIFEVKHEGKVTGYACLVGDKVMKPAHVKGTIDNADLAKLAFKRSSKYDLECAQIPVHMKSDASKFTHEKPEGYYNWHHGAVQYSGGRFTIPTGAGKPGDSGRPIFDNKGRVVAIVLGGANEGARTALSVVTWNKDIVTKITPEGAEEWSLAIPVMCLLANTTFPCSQPPCTPCCYEKEPEETLRMLEDNVMRPGYYQLLQASLTCSPHRQRRSTKDNFNVYKATRPYLAHCPDCGEGHSCHSPVALERIRNEATDGTLKIQVSLQIGIKTDDSHDWTKLRYMDNHMPTDAERAGLFVRTSAPCTITGTMGHFILARCPKGETLTVGFTDSRKISHSCTHPFHHDPPVIGREKFHSRPQHGKELPCSTYVQSNAATAEEIEVHMPPDTPDRTLLSQQSGNVKITVNGQTVRYKCNCGGSNEGLTTTDKVINNCKVDQCHAAVTNHKKWQYNSPLVPRNAELGDRKGKIHIPFPLANATCRVPKARNPTVTYGKNQVIMLLYPDHPTLLSYRNMGEEPNYQEEWVMHKKEVVLTVPTEGLEVTWGNNEPYKYWPQLSTNGTAHGHPHEIILYYYELYPTMTVVVVSVATFILLSMVGMAAGMCMCARRRCITPYELTPGATVPFLLSLICCIRTAKAATYQEAAIYLWNEQQPLFWLQALIPLAALIVLCNCLRLLPCCCKTLAFLA

>ACY09941.1:1-798 structural polyprotein [Chikungunya virus]MEFIPTQTFYNRRYQPRPWTPRSTIQVIRPRPRPQRQAGQLAQLISAVNKLTMRAVPQQKPRRNRKNKKQKQKQQAPQNNTNQKKQPPKKKPAQKKKKPGRRERMCMKIENDCIFEVKHEGKVTGYACLVGDKVMKPAHVKGTIDNADLAKLAFKRSSKYDLECAQIPVHMKSDASKFTHEKPEGYYNWHHGAVQYSGGRFTIPTGAGKPGDSGRPIFDNKGRVVAIVLGGANEGARTALSVVTWNKDIVTKITPEGAEEWSLAIPVMCLLANTTFPCSQPPCTPCCYEKEPEETLRMLEDNVMRPGYYQLLQASLTCSPHRQRRSTKDNFNVYKATRPYLAHCPDCGEGHSCHSPVALERIRNEATDGTLKIQVSLQIGIKTDDSHDWTKLRYMDNHMPADAERAGLFVRTSAPCTITGTMGHFILARCPKGETLTVGFTDSRKISHSCTHPFHHDPPVIGREKFHSRPQHGKELPCSTYVQSTAATTEEIEVHMPPDTPDRTLMSQQSGNVKITVNGQTVRYKCNCGGSNEGLTTTDKVINNCKIDQCHAAVTNHKKWQYNSPLVPRNAELGDRKGKIHIPFPLANVTCRVPKARNPTVTYGKNQVIMLLYPDHPTLLSYRNMGEEPNYQEEWVMHKKEVVLTVPTEGLEVTWGNNEPYKYWPQLSTNGTAHGHPHEIILYYYELYPTMTVVVVSVATFILLSMVGMAAGMCMCARRRCITPYELTPGATVPFLLSLICCIRTAKAATYQEAAIYLWNEQQPLFWLQALIPLAALIVLCNCLRLLPCCCKTLAFLA

>ACY25942.1:1-798 structural polyprotein [Chikungunya virus]MEFIPTQTFYNRRYQPRPWTPRSTIQIIRPRPRPQRQAGQLAQLISAVNKLTMRAVPQQKPRRNRKNKKQKQKQQAPQNNTNQKKQPPKKKPAQKKKKPGRRERMCMKIENDCIFEVKHEGKVTGYACLVGDKVMKPAHVKGTIDNADLAKLAFKRSSKYDLECAQIPVHMKSDASKFTHEKPEGYYNWHHGAVQYSGGRFTIPTGAGKPGDSGRPIFDNKGRVVAIVLGGANEGARTALSVVTWNKDIVTKITPEGAEEWSLAIPVMCLLANTTFPCSQPPCTPCCYEKEPEETLRMLEDNVMRPGYYQLLQASLTCSPHRQRRSTKDNFNVYKATRPYLAHCPDCGEGHSCHSPVALERIRNEATDGTLKIQVSLQIGIKTDDSHDWTKLRYMDNHMPADAERAGLFVRTSAPCTITGTMGHFILARCPKGETLTVGFTDSRKISHSCTHPFHHDPPVIGREKFHSRPQHGKELPCSTYVQSTAATTEEIEVHMPPDTPDRTLMSQQSGNVKITVNGQTVRYKCNCGGSNEGLTTTDKVINNCKVDQCHAAVTNHKKWQYNSPLVPRNAELGDRQGKIHIPFPLANVTCRVPKARNPTVTYGKNQVIMLLYPDHPTLLSYRNMGEEPNYQEEWVMHKKEVVLTVPTEGLEVTWGNNEPYKYWPQLSTNGTAHGHPHEIILYYYELYPTMTVVVVSVATFILLSMVGMAAGMCMCARRRCITPYELTPGATVPFLLSLICCIRTAKAATYQEAAIYLWNEQQPLFWLQALIPLAALIVLCNCLRLLPCCCKTLAFLA

>AHB79113.1:1-798 structural polyprotein [Chikungunya virus]MEFIPTQTFYNRRYQPRPWTPRSTIQVIRPRPRPQRQAGQLAQLISAVNKLTMRAVPQQKPRRNRKNKKQKQKQQAPQNNTNRKKQPPKKKPAQKKKKPGRRERMCMKIENDCIFEVKHEGKVTGYACLVGDKVMKPAHVKGTIDNADLAKLAFKRSSKYDLECAQIPVHMKSDASKFTHEKPEGYYNWHHGAVQYSGGRFTIPTGAGKPGDSGRPIFDNKGRVVAIVLGGANEGARTALSVVTWNKDIVTKITPEGAEEWSLAIPVMCLLANTTFPCSQPPCTPCCYEKEPEETLRMLEDNVMRPGYYQLLQASLTCSPHRQRRSTKDNFNVYKATRPYLAHCPDCGEGHSCHSPVALERIRNEATDGTLKIQVSLQIGIKTDDSHDWTKLRYMDNHMPADAERAGLFVRTSAPCTITGTMGHFILARCPKGETLTVGFTDSRKISHSCTHPFHHDPPVIGREKFHSRPQHGKELPCSTYVQSTAATTEEIEVHMPPDTPDRTLMSQQSGNVKITVNGQTVRYKCNCGGSNEGLTTTDKVINNCKVDQCHAAVTNHKKWQYNSPLVPRNAELGDRKGKIHIPFPLANVTCRVPKARNPTVTYGKNQVIMLLYPDHPTLLSYRNMGEEPNYQEEWVMHKKEVVLTVPTEGLEVTWGNNEPYKYWPQLSTNGTAHGHPHEIILYYYELYPTMTVVVVSVATFILLSMVGMAAGMCMCARRRCITPYELTPGATVPFLLSLICCIRTAKAATYQEAAIYLWNEQQPLFWLQALIPLAALIVLCNCLRLLPCCCKTLAFLA

>AHB79111.1:1-798 structural polyprotein [Chikungunya virus]MEFIPTQTFYNRRYQPRPWTPRSTIQIIRPRPRPQRQAGQLAQLISAVNKLTMRAVPQQKPRRNRKNKKQKQKQQAPQNNTNQKKQPPKKKPAQKKKKPGRRERMCMKIENDCIFEVKHEGKVTGYACLVGDKVMKPAHVKGTIDNADLAKLAFKRSSKYDLECAQIPVHMKSDASKFTHEKPEGYYNWHHGAVQYSGGRFTIPTGAGKPGDSGRPIFDNKGRVVAIVLGGANEGARTALSVVTWNKDIVTKITPEGAEEWSLAIPVMCLLANTTFPCSQPPCTPCCYEKEPEETLRMLEDNVMRPGYYQLLQASLTCSPHRQRRSTKDNFNVYKATRPYLAHCPDCGEGHSCHSPVALERIRNEATDGTLKIQVSLQIGIKTDDSHDWTKLRYMDNHMPADAERAGLFVRTSAPCTITGTMGHFILARCPKGETLTVGFTDSRKISHSCTHPFHHDPPVIGREKFHSRPQHGKELPCSTYVQSTAATTEEIEVHMPPDTPDRTIMSQQSGNVKITVNGQTVRYKCNCGGSNEGLTTTDKVINNCKVDQCHAAVTNHKKWQYNSPLVPRNAELGDRKGKIHIPFPLANVTCRVPKARNPTVTYGKNQVIMLLYPDHPTLLSYRNMGEEPNYQEEWVMHKKEVVLTVPTEGLEVTWGNNEPYKYWPQLSTNGTAHGHPHEIILYYYELYPTMTVVVVSVATFILLSMVGMAAGMCMCARRRCITPYELTPGATVPFLLSLICCIRTAKAATYQEAAIYLWNEQQPLFWLQALIPLAALIVLCNCLRLLPCCCKTLAFLA

>ARU83751.1:1-798 structural polyprotein [Chikungunya virus]MEFIPTQTFYNRRYQPRPWTPRSTIQIIRPRPRPQRQAGQLAQLISAVNKLTMRAVPQQKPRRNRKNKKQKQKQQAPQNNTNQKKQPPKKKPAQKKKKPGRRERMCMKIENDCIFEVKHEGKVTGYACLVGDKVMKPAHVKGTIDNADLAKLAFKRSSKYDLECAQIPVHMKSDASKFTHEKPEGYYNWHHGAVQYSGGRFTIPTGAGKPGDSGRPIFDNKGRVVAIVLGGANEGARTALSVVTWNKDIVTKITPEGAEEWSLAIPVMCLLANTTFPCSQPPCTPCCYEKEPEETLRMLEDNVMRPGYYQLLQASLTCSPHRQRRSTKDNFNVYKATRPYLAHCPDCGEGHSCHSPVALERIRNEATDGTLKIQVSLQIGIKTDDSHDWTKLRYMDNHMPADAERAGLFVRTSAPCTITGTMGHFILARCPKGETLTVGFTDSRKISHSCTHPFHHDPPVIGREKFHSRPQHGKELPCSTYVQSTAATTEEIEVHMPPDTPDRTLMSQQSGNVKITVNGQTVRYKCNCGGSNEGLTTTDKVINNCKVDQCHAAVTNHKKWQYNSPLVPRNAELGDRKGKIHIPFPLANATCRVPKARNPTVTYGKNQVIMLLYPDHPTLLSYRNMGEEPNYQEEWVMHKKEVVLTVPTEGLEVTWGNNEPYKYWPQLSTNGTAHGHPHEIILYYYELYPTMTVVVVSVATFILLSMVGMAAGMCMCARRRCITPYELTPGATVPFLLSLICCIRTAKAATYQEAAIYLWNEQQPLFWLQALIPLAALIVLCNCLRLLPCCCKTLAFLA

>AHC05720.1:1-798 structural polyprotein [Chikungunya virus]MEFIPTQTFYNRRYQPRPWTPRSTIQIIRPRPRPQRQAGQLAQLISAVNKLTMRAVPQQKPRRNRKNKKQKQKQQAPQNNTNQKKQPPKKKPAQKKKKPGRRERMCMKIENDCIFEVKHEGKVTGYACLVGDKVMKPAHVKGTIDNADLAKLAFKRSSKYDLECAQIPVHMKSDASKFTHEKPEGYYNWHHGAVQYSGGRFTIPTGAGKPGDSGRPIFDNKGRVVAIVLGGANEGARTALSVVTWNKDIVTKITPEGAEEWSLAIPVMCLLANTTFPCSQPPCTPCCYEKEPEETLRMLEDNVMRPGYYQLLQASLTCSPHRQRRSTKDNFNVYKATRPYLAHCPDCGEGHSCHSPVALERIRNEATDGTLKIQVSLQIGIKTDDSHDWTKLRYMDNHMPADAERAGLFVRTSAPCTITGTMGHFILARCPKGETLTVGFTDSRKISHSCTHPFHHDPPVIGREKFHSRPQHGKELPCSTYVQSTAATTEEIEVHMPPDTPDRTLMSQQSGNVKITVNGQTVRYKCNCGGSNEGLTTTDKVINNCKVDQCHAAVTNHKKWQYNSPLVPRNAELGDRKGKIHIPFPLANATCRVPKARNPTVTYGKNQVIMLLYPDHPTLLSYRNMGEEPNYQEEWVMHKKEVVLTVPTEGLEVTWGNNEPYKYWPQLSTNGTAHGHPHEIILYYYELYPTMTVVVVSVATFILLSMVGMAAGMCMCARRRCITPYELTPGATVPFLLSLICCIRTAKAATYQEAAIYLWNEQQPLFWLQALIPLAALIVLCNCLRLLPCCCKTLAFLA

>ACV88657.1:1-798 structural polyprotein [Chikungunya virus]MEFIPTQTFYNRRYQPRPWTPRPTIQVIRPRPRPQRQAGQLAQLISAVNKLTMRAVPQQKPRRNRKNKKQKQKQQAPQNNTNQKKQPPKKKPAQKKKKPGRRERMCMKIENDCIFEVKHEGKVTGYACLVGDKVMKPAHVKGTIDNADLAKLAFKRSSKYDLECAQIPVHMKSDASKFTHEKLEGYYNWHHGAVQYSGGRFTIPTGAGKPGDSGRPIFDNKGRVVAIVLGGANEGARTALSVVTWNKDIVTKITPEGAEEWSLAIPVMCLLANTTFPCSQPPCTPCCYEKEPEETLRMLEDNVMRPGYYQLLQASLTCSPHRQRRSTKDNFNVYKATRPYLAHCPDCGEGHSCHSPVALERIRNEATDGTLKIQVSLQIGIKTDDSHDWTKLRYMDNHMPADAERAGLFVRTSAPCTITGTMGHFILARCPKGETLTVGFTDSRKISHSCTHPFHHDPPVIGREKFHSRPQHGKELPCSTYVQSTAATTEEIEVHMPPDTPDRTLMSQQSGNVKITVNGQTVRYKCNCGGSNEGLTTTDKVINNCKVDQCHAAVTNHKKWQYNSPLVPRNAELGDRKGKIHIPFPLANVTCRVPKARNPTVTYGKNQVIMLLYPDHPTLLSYRNMGEEPNYQEEWVMHKKEVVLTVPTEGLEVTWGNNEPYKYWPQLSTNGTAHGHPHEIILYYYELYPTMTVVVVSVATFILLSMVGMAAGMCMCARRRCITPYELTPGATVPFLLSLICCIRTAKAATYQEAAIYLWNEQQPLFWLQALIPLAALIVLCNCLRLLPCCCKTLAFLA

>ADC53730.1:1-798 structural polyprotein [Chikungunya virus]MEFIPTQTFYNRRYQPRPWTPRSTIQVIRPRPRPQRQAGQLAQLISAVNKLTMRAVPQQKPRRNRKNKKQKQKQQAPQNNTNQKKQPPKKKPAQKKKKPGRRERMCMKIENDCIFEVKHEGKVTGYACLVGDKVMKPAHVKGTIDNADLAKLAFKRSSKYDLECAQIPVHMKSDASKFTHEKPEGYYNWHHGAVQYSGGRFTIPTGAGKPGDSGRPIFDNKGRVVAIVLGGANEGARTALSVVTWNKDIVTKITPEGAEEWSLAIPVMCLLANTTFPCSQPPCTPCCYEKEPEETLRMLEDNVMRPGYYQLLQASLTCSPHRQRRSTKDNFNVYKATRPYLAHCPDCGEGHSCHSPVALERIRNEATDGTLKIQVSLQIGIKTDDSHDWTKLRYMDNHMPADAERAGLFVRTSAPCTITGTMGHFILARCPKGETLTVGFTDSRKISHSCTHPFHHDPPVIGREKFHSRPQHGKELPCSTYVQSTAATTEEIEVHMPPDTPDRTLMSQQSGNVKITVNGQTVRYKCNCGGSNEGLTTTDKVINNCKIDQCHAAVTNHKKWQYNSPLVPRNAELGDRKGKIHIPFPLANVTCRVPKARNPTVTYGKNQVIMLLYPDHPTLLSYRNMGEEPNYQEEWVMHKKEVVLTVPTEGLEVTWGNNEPYKYWPQLSTNGTAHGHPHEIILYYYELYPTMTVVVVSVATFILLSMVGMAAGMCMCARRRCITPYELTPGATVPFLLSLICCIRTAKAATYQEAAIYLWNEQQPLFWLQALIPLAALIVLCNCLRLLPCCCKTLAFLA

>ACY66845.1:1-798 structural polyprotein [Chikungunya virus]MEFIPTQTFYNRRYQPRPWTPRSTIQVIRPRPRPQRQAGQLAQLISAVNKLTMRAVPQQKPRRNRKNKKQKQKQQAPQNNTNQKKQPPKKKPAQKKKKPGRRERMCMKIENDCIFEVKHEGKVTGYACLVGDKVMKPAHVKGTIDNADLAKLAFKRSSKYDLECAQIPVHMKSDASKFTHEKPEGYYNWHHGAVQYSGGRFTIPTGAGKPGDSGRPIFDNKGRVVAIVLGGANEGARTALSVVTWNKDIVTKITPEGAEEWSLAIPVMCLLANTTFPCSQPPCTPCCYEKEPEETLRMLEDNVMRPGYYQLLQASLTCSPHRQRRSTKDNFNVYKATRPYLAHCPDCGEGHSCHSPVALERIRNEATDGTLKIQVSLQIGIKTDDSHDWTKLRYMDNHMPADAERAGLFVRTSAPCTITGTMGHFILARCPKGETLTVGFTDSRKISHSCTHPFHHDPPVIGREKFHSRPQHGKELPCSTYVQSTAATTEEIEVHMPPDTPDRTLMSQQSGNVKITVNGQTVRYKCNCGGSNEGLTTTDKVINNCKVDQCHAAVTNHKKWQYNSPLVPRNAELGDRKGKIHIPFPLANVTCRVPKARNPTVTYGKNQVIMLLYPDHPTLLSYRNMGEEPNYQEEWVMHKKEVVLAVPTEGLEVTWGNNEPYKYWPQLSTNGTAHGHPHEIILYYYELYPTMTVVVVSVATFILLSMVGMAAGMCMCARRRCITPYELTPGATVPFLLSLICCIRTAKAATYQEAAIYLWNEQQPLFWLQALIPLAALIVLCNCLRLLPCCCKTLAFLA

>ACM09921.1:1-798 structural polyprotein [Chikungunya virus]MEFIPTQTFYNRRYQPRPWTPRSTIQIIRPRPRPQRQAGQLAQLISAVNKLTMRAVPQQKPRRNRKNKKQKQKQQAPQNNTNQKKQPPKKKPAQKKKKPGRRERMCMKIENDCIFEVKHEGKVTGYACLVGDKVMKPAHVKGTIDNADLAKLAFKRSSKYDLECAQIPVHMKSDASKFTHEKPEGYYNWHHGAVQYSGGRFTIPTGAGKPGDSGRPIFDNKGRVVAIVLGGANEGARTALSVVTWNKDIVTKITPEGAEEWSLAIPVMCLLANTTFPCSQPPCTPCCYEKEPEETLRMLEDNVMRPGYYQLLQASLTCSPHRQRRSTKDNFNVYKATRPYLAHCPDCGEGHSCHSPVALERIRNEATDGTLKIQVSLQIGIKTDDSHDWTKLRYMDNHMPADAERAGLFVRTSAPCTITGTMGHFILARCPKGETLTVGFTDSRKISHSCTHPFHHDPPVIGREKFHSRPQHGKELPCSTYVQSTAATTEEIEVHMPPDTPDRTLMSQQSGNVKITVNGQTVRYKCNCGGSNEGLTTTDKVINNCKVDQCHAAVTNHKKWQYNSPLVPRNAELGDRQGKIHIPFPLANVTCRVPKARNPTVTYGKNQVIMLLYPDHPTLLSYRNMGEEPNYQEEWVMHKKEVVLTVPTEGLEVTWGNNEPYKYWPQLSTNGTAHGHPHEIILYYYELYPTMTVVVVSVATFILLSMVGMAAGMCMCARRRCITPYELTPGATVPFLLSLICCIRTAKAATYQEAAIYLWNEQQPLFWLQALIPLAALIVLCNCLRLLPCCCKTLAFLA

>ARD08054.1:1-798 structural polyprotein [Chikungunya virus]MEFIPTQTFYNRRYQPRPWTPRSTIQIIRPRPRPQRQAGQLAQLISAVNKLTMRAVPQQKPRRNRKNKKQKQKQQAPQNNTNQKKQPPKKKPAQKKKKPGRRERMCMKIENDCIFEVKHEGKVTGYACLVGDKVMKPAHVKGTIDNADLAKLAFKRSSKYDLECAQIPVHMKSDASKFTHEKPEGYYNWHHGAVQYSGGRFTIPTGAGKPGDSGRPIFDNKGRVVAIVLGGANEGARTALSVVTWNKDIVTKITPEGAEEWSLAIPVMCLLANTTFPCSQPPCTPCCYEKEPEETLRMLEDNVMRPGYYQLLQASLTCSPHRQRRSTKDNFNVYKATRPYLAHCPDCGEGHSCHSPVALERIRNEATDGTLKIQVSLQIGIKTDDSHDWTKLRYMDNHMPADAERAGLFVRTSAPCTITGTMGHFILARCPKGETLTVGFTDSRKISHSCTHPFHHDPPVIGREKFHSRPQHGKELPCSTYVQSTAATTEEIEVHMPPDTPDRTLMSQQSGNVKITVNGQTVRYKCNCGGSNEGLTTTDKVINNCKVDQCHAAVTNHKKWQYNSPLVPRNAELGDRKGKIHIPFPLANATCRVPKARNPTVTYGKNQVIMLLYPDHPTLLSYRNMGEEPNYQEEWVMHKKEVVLTVPTEGLEVTWGNNEPYKYWPQLSTNGTAHGHPHEIILYYYELYPTMTVVVVSVATFILLSMVGMAAGMCMCARRRCITPYELTPGATVPFLLSLICCIRTAKAATYQEAAIYLWNEQQPLFWLQALIPLAALIVLCNCLRLLPCCCKTLAFLA

>BAP74202.1:1-798 structural polyprotein [Chikungunya virus]MEFIPTQTFYNRRYQPRPWTPRSTIQIIRPRPRPQRQAGQLAQLISAVNKLTMRAVPQQKPRRNRKNKKQKQKQQAPQNNTNQKKQPPKKKPAQKKKKPGRRERMCMKIENDCIFEVKHEGKVTGYACLVGDKVMKPAHVKGTIDNADLAKLAFKRSSKYDLECAQIPVHMKSDASKFTHEKPEGYYNWHHGAVQYSGGRFTIPTGAGKPGDSGRPIFDNKGRVVAIVLGGANEGARTALSVVTWNKDIVTKITPEGAEEWSLAIPVMCLLANTTFPCSQPPCTPCCYEKEPEETLRMLEDNVMRPGYYQLLQASLTCSPHRQRRSTKDNFNVYKATRPYLAHCPDCGEGHSCHSPVALERIRNEATDGTLKIQVSLQIGIKTDDSHDWTKLRYMDNHMPADAERAGLFVRTSAPCTITGTMGHFILARCPKGETLTVGFTDSRKISHSCTHPFHHDPPVIGREKFHSRPQHGKELPCSTYVQSTAATTEEIEVHMPPDTPDRTLMSQQSGNVKITVNGQTVRYKCNCGGSNEGLTTTDKVINNCKVDQCHAAVTNHKKWQYNSPLVPRNAELGDRQGKIHIPFPLANVTCRVPKARNPTVTYGKNQVIMLLYPDHPTLLSYRNMGEEPNYQEEWVMHKKEVVLTVPTEGLEVTWGNNEPYKYWPQLSTNGTAHGHPHEIILYYYELYPTMTVVVVSVATFILLSMVGMAAGMCMCARRRCITPYELTPGATVPFLLSLICCIRTAKAATYQEAAIYLWNEQQPLFWLQALIPLAALIVLCNCLRLLPCCCKTLAFLA

>QAB14116.1:1-798 structural polyprotein [Chikungunya virus]MEFIPTQTFYNRRYQPRPWTPRSTIQIIRPRPRPQRQAGQLAQLISAVNKLTMRAVPQQKPRRNRKNKKQKQKQQAPQNNTNQKKQPPKKKPAQKKKKPGRRERMCMKIENDCIFEVKHEGKVTGYACLVGDKVMKPAHVKGTIDNADLAKLAFKRSSKYDLECAQIPVHMKSDASKFTHEKPEGYYNWHHGAVQYSGGRFTIPTGAGKPGDSGRPIFDNKGRVVAIVLGGANEGARTALSVVTWNKDIVTKITPEGAEEWSLAIPVMCLLANTTFPCSQPPCSPCCYEKEPEETLRMLEDNVMRPGYYQLLQASLTCSPHRQRRSTKDNFNVYKATRPYLAHCPDCGEGHSCHSPVALERIRNEATDGTLKIQVSLQIGIKTDDSHDWTKLRYMDNHMPADAERAGLFVRTSAPCTITGTMGHFILARCPKGETLTVGFTDSRKISHSCTHPFHHDPPVIGREKFHSRPQHGKELPCSTYVQSTAATTEEIEVHMPPDTPDRTLMSQQSGNVKITVNGQTVRYKCNCGGSNEGLTTTDKVINNCKVDQCHAAVTNHKKWQYNSPLVPRNAELGDRKGKIHIPFPLANATCRVPKARNPTVTYGKNQVIMLLYPDHPTLLSYRNMGEEPNYQEEWVMHKKEVVLTVPTEGLEVTWGNNEPYKYWPQLSTNGTAHGHPHEIILYYYELYPTMTVVVVSVATFILLSMVGMAAGMCMCARRRCITPYELTPGATVPFLLSLICCIRTAKAATYQEAAIYLWNEQQPLFWLQALIPLAALIVLCNCLRLLPCCCKTLAFLA

>ASY03806.1:1-798 structural polyprotein [Chikungunya virus]MEFIPTQTFYNRRYQPRPWTPRSTIQIIRPRPRPQRQAGQLAQLISAVNKLTMRAVPQQKPRRNRKNKKQKQKQQAPQNNTNQKKQPPKKKPAQKKKKPGRRERMCMKIENDCIFEVKHEGKVTGYACLVGDKVMKPAHVKGTIDNADLAKLAFKRSSKYDLECAQIPVHMKSDASKFTHEKPEGYYNWHHGAVQYSGGRFTIPTGAGKPGDSGRPIFDNKGRVVAIVLGGANEGARTALSVVTWNKDIVTKITPEGAEEWSLAIPVMCLLANTTFPCSQPPCTPCCYEKEPEETLRMLEDNVMRPGYYQLLQASLTCSPHRQRRSTKDNFNVYKATRPYLAHCPDCGEGHSCHSPVALERIRNEATDGTLKIQVSLQIGIKTDDSHDWTKLRYMDNHMPADAERAGLFVRTSAPCTITGTMGHFILARCPKGETLTVGFTDSRKISHSCTHPFHHDPPVIGREKFHSRPQHGKELPCSTYVQSTAATTEEIEVHMPPDTPDRTLMSQQSGNVKITVNGQTVRYKCNCGGSNEGLTTTDKVINNCKVDQCHAAVTNHKKWQYNSPLVPRNAELGDRKGKIHIPFPLANATCRVPKARNPTVTYGKNQVIMLLYPDHPTLLSYRNMGEEPNYQEEWVMHKKEVVLTVPTEGLEVTWGNNEPYKYWPQLSTNGTAHGHPHEIILYYYELYPTMTVVVVSVATFILLSMVGMAAGMCMCARRRCITPYELTPGATVPFLLSLICCIRTAKAATYQEAAIYLWNEQQPLFWLQALIPLAALIVLCNCLRLLPCCCKTLAFLA

>ANY26985.1:1-798 structural polyprotein [Chikungunya virus]MEFIPTQTFYNRRYQPRPWTPRSTIQIIRPRPRPQRQAGQLAQLISAVNKLTMRAVPQQKPRRNRKNKKQKQKQQAPQNNTNQKKQPPKKKPAQKKKKPGRRERMCMKIENDCIFEVKHEGKVTGYACLVGDKVMKPAHVKGTIDNADLAKLAFKRSSKYDLECAQIPVHMKSDASKFTHEKPEGYYNWHHGAVQYSGGRFTIPTGAGKPGDSGRPIFDNKGRVVAIVLGGANEGARTALSVVTWNKDIVTKITPEGAEEWSLAIPVMCLLANTTFPCSQPPCTPCCYEKEPEETLRMLEDNVMRPGYYQLLQASLTCSPHRQRRSTKDNFNVYKATRPYLAHCPDCGEGHSCHSPVALERIRNEATDGTLKIQVSLQIGIKTDDSHDWTKLRYMDNHMPADAERAGLFVRTSAPCTITGTMGHFILARCPKGETLTVGFTDSRKISHSCTHPFHHDPPVIGREKFHSRPQHGKELPCSTYVQSTAATTEEIEVHMPPDTPDRTLMSQQSGNVKITVNGQTVRYKCNCGGSNEGLTTTDKVINNCKVDQCHAAVTNHKKWQYNSPLVPRNAELGDRQGKIHIPFPLANVTCRVPKARNPTVTYGKNQVIMLLYPDHPTLLSYRNMGEEPNYQEEWVMHKKEVVLTVPTEGLEVTWGNNEPYKYWPQLSTNGTAHGHPHEIILYYYELYPTMTVVVVSVATFILLSMVGMAAGMCMCARRRCITPYELTPGATVPFLLSLICCIRTAKAATYQEAAIYLWNEQQPLFWLQALIPLAALIVLCNCLRLLPCCCKTLAFLA

>ARF20161.1:1-798 structural polyprotein [Chikungunya virus]MEFIPTQTFYNRRYQPRPWTPRSTIQIIRPRPRPQRQAGQLAQLISAVNKLTMRAVPQQKPRRNRKNKKQKQKQQAPQNNTNQKKQPPKKKPAQKKKKPGRRERMCMKIENDCIFEVKHEGKVTGYACLVGDKVMKPAHVKGTIDNADLAKLAFKRSSKYDLECAQIPVHMKSDASKFTHEKPEGYYNWHHGAVQYSGGRFTIPTGAGKPGDSGRPIFDNKGRVVAIVLGGANEGARTALSVVTWNKDIVTKITPEGAEEWSLAIPVMCLLANTTFPCSQPPCTPCCYEKEPEETLRMLEDNVMRPGYYQLLQASLTCSPHRQRRSTKDNFNVYKATRPYLAHCPDCGEGHSCHSPVALERIRNEATDGTLKIQVSLQIGIKTDDSHDWTKLRYMDNHMPADAERAGLFVRTSAPCTITGTMGHFILARCPKGETLTVGFTDSRKISHSCTHPFHHDPPVIGREKFHSRPQHGKELPCSTYVQSTAATTEEIEVHMPPDTPDRTLMSQQSGNVKITVNGQTVRYKCNCGGSNEGLTTTDKVINNCKVDQCHAAVTNHKKWQYNSPLVPRNAELGDRKGKIHIPFPLANATCRVPKARNPTVTYGKNQVIMLLYPDHPTLLSYRNMGEEPNYQEEWVMHKKEVVLTVPTEGLEVTWGNNEPYKYWPQLSTNGTAHGHPHEIILYYYELYPTMTVVVVSVATFILLSMVGMAAGMCMCARRRCITPYELTPGATVPFLLSLICCIRTAKAATYQEAAIYLWNEQQPLFWLQALIPLAALIVLCNCLRLLPCCCKTLAFLA

>QOW97266.1:1-798 structural polyprotein, partial [Chikungunya virus]MEYIPTQTFYNRRYQPRPWTPRPTIQVIRPRPRPQRQAGQLAQLISAVNKLTMRAVPQQKPRRNRKNKKQKQKRQAPQNNTNQKKQPPKKKPAQKKKKPGRRERMCMKIENDCIFEVKYEGKVTGYACLVGDKVMKPAHVKGTIDNADLAKLAFKRSSKYDLECAQIPVHMKSDASKFTHEKPEGYYNWHHGAVQYSGGRFTIPTGAGKPGDSGRPIFDNKGRVVAIVLGGANEGARTALSVVTWNKDIVTKITPEGAEEWSLAIPVMCLLANTTFPCSQPPCTPCCYEREPEETLRMLEDNVMRPGYYQLLQASLTCSPHRQRRSTKDNFNVYKATRPYLAHCPDCGEGHSCHSPVALERIRNEATDGTLKIQVSLQIGIKTDDSHDWTKLRYMDNHMPADAERAGLFVRTSAPCTITGTMGHFILTRCPKGETLTVGFTDSRKISHSCTHPFHHDPPVIGREKFHSRPQHGKELPCSTYVQSTAATTEEIEVHMPPDTPDRTLMSQQSGNVKITVNGQTVRYKCNCGGSNEGLITTDKVINNCKVDQCHAAVTNHKKWQYNSPLVPRNAELGDRKGKIHIPFPLANVTCRVPKARNPTVTYGKNQVIMLLYPDHPTLLSYRNMGEEPNYQEEWVTHKKEVVLTVPTEGLEVTWGNNEPYKYWPQLSTNGTAHGHPHEIILYYYELYPTMTVVVVSVASFVLLSMVGVAVGMCMCARRRCITPYELTPGATVPFLLSLICCIRTAKAATYQEAAVYLWNEQQPLFWLQALIPLAALIVLCNCLRLLPCCCKTLAFLA

>APA34057.1:1-798 structural polyprotein [Chikungunya virus]MEFIPTQTFYNRRYQPRPWTPRSTIQIIRPRPRPQRQAGQLAQLISAVNKLTMRAVPQQKPRRNRKNKKQKQKQQAPQNNTNQKKQPPKKKPAQKKKKPGRRERMCMKIENDCIFEVKHEGKVTGYACLVGDKVMKPAHVKGTIDNADLAKLAFKRSSKYDLECAQIPVHMKSDASKFTHEKPEGYYNWHHGAVQYSGGRFTIPTGAGKPGDSGRPIFDNKGRVVAIVLGGANEGARTALSVVTWNKDIVTKITPEGAEEWSLAIPVMCLLANTTFPCSQPPCTPCCYEKEPEETLRMLEDNVMRPGYYQLLQASLTCSPHRQRRSTKDNFNVYKATRPYLAHCPDCGEGHSCHSPVALERIRNEATDGTLKIQVSLQIGIKTDDSHDWTKLRYMDNHMPADAERAGLFVRTSAPCTITGTMGHFILARCPKGETLTVGFTDSRKISHSCTHPFHHDPPVIGREKFHSRPQHGKELPCSTYVQSTAATTEEIEVHMPPDTPDRTLMSQQSGNVKITVNGQTVRYKCNCGGSNEGLTTTDKVINNCKVDQCHAAVTNHKKWQYNSPLVPRNAELGDRKGKIHIPFPLANATCRVPKARNPTVTYGKNQVIMLLYPDHPTLLSYRNMGEEPNYQEEWVMHKKEVVLTVPTEGLEVTWGNNEPYKYWPQLSTNGTAHGHPHEIILYYYELYPTMTVVVVSVATFILLSMVGMAAGMCMCARRRCITPYELTPGATVPFLLSLICCIRTAKAATYQEAAIYLWNEQQPLFWLQALIPLAALIVLCNCLRLLPCCCKTLAFLA

>AFP33173.1:1-798 structural polyprotein [Chikungunya virus]MEFIPTQTFYNRRYQPRPWTPRSTIQIIRPRPRPQRQAGQLAQLISAVNKLTMRAVPQQKPRRNRKNKKQKQKQQAPQNDTNQKKQPPKKKPAQKKKKPGRRERMCMKIENDCIFEVKHEGKVTGYACLVGDKVMKPAHVKGTIDNADLAKLAFKRSSKYDLECAQIPVHMKSDASKFTHEKPEGYYNWHHGAVQYSGGRFTIPTGAGKPGDSGRPIFDNKGRVVAIVLGGANEGARTALSVVTWNKDIVTKITPEGAEEWSLAIPVMCLLANTTFPCSQPPCTPCCYEKEPEETLRMLEDNVMRPGYYQLLQASLTCSPHRQRRSTKDNFNVYKATRPYLAHCPDCGEGHSCHSPVALERIRNEATDGTLKIQVSLQIGIKTDDSHDWTKLRYMDNHMPADAERAGLFVRTSAPCTITGTMGHFILARCPKGETLTVGFTDSRKISHSCTHPFHHDPPVIGREKFHSRPQHGKELPCSTYVQSTAATTEEIEVHMPPDTPDRTLMSQQSGNVKITVNGQTVRYKCNCGGSNEGLTTTDKVINNCKVDQCHAAVTNHKKWQYNSPLVPRNAELGDRKGKIHIPFPLANVTCRVPKARNPTVTYGKNQVIMLLYPDHPTLLSYRNMGEEPNYQEEWVMHKKEVVLTVPTEGLEVTWGNNEPYKYWPQLSTNGTAHGHPHEIILYYYELYPTMTVVVVSVATFILLSMVGMAAGMCMCARRRCITPYELTPGATVPFLLSLICCIRTAKAATYQEAAIYLWNEQQPLFWLQALIPLAALIVLCNCLRLLPCCCKTLAFLA

>BBC18140.1:1-798 structural polyprotein [Chikungunya virus]MEFIPTQTFYNRRYQPRPWTPRSTIQIIRPRPRPQRQAGQLAQLISAVNKLTMRAVPQQKPRRNRKNKKQKQKQQAPQNNTNQKKQPPKKKPAQKKRKPGRRERMCMKIENDCIFEVKHEGKVTGYACLVGDKVMKPAHVKGTIDNADLAKLAFKRSSKYDLECAQIPVHMKSDASKFTHEKPEGYYNWHHGAVQYSGGRFTIPTGAGKPGDSGRPIFDNKGRVVAIVLGGANEGARTALSVVTWNKDIVTKITPEGAEEWSLAIPVMCLLANTTFPCSQPPCTPCCYEKEPEETLRMLEDNVMRPGYYQLLQASLTCSPHRQRRSTKDNFNVYKATRPYLAHCPDCGEGHSCHSPVALERIRNEATDGTLKIQVSLQIGIKTDDSHDWTKLRYMDNHMPADAERAGLFVRTSAPCTITGTMGHFILARCPKGETLTVGFTDSRKISHSCTHPFHHDPPVIGREKFHSRPQHGKELPCSTYVQSTAATTEEIEVHMPPDTPDRTLMSQQSGNVKITVNGQTVRYKCNCGGSNEGLTTTDKVINNCKVDQCHAAVTNHKKWQYNSPLVPRNAELGDRQGKIHIPFPLANVTCRVPKARNPTVTYGKNQVIMLLYPDHPTLLSYRNMGEEPNYQEEWVMHKKEVVLTVPTEGLEVTWGNNEPYKYWPQLSTNGTAHGHPHEIILYYYELYPTMTVVVVSVATFILLSMVGMAAGMCMCARRRCITPYELTPGATVPFLLSLICCIRTAKAATYQEAAIYLWNEQQPLFWLQALIPLAALIVLCNCLRLLPCCCKTLAFLA

>QEP54778.1:1-798 structural polyprotein [Chikungunya virus]MEFIPTQTFYNRRYQPRPWTPRSTIQIIRPRPRPQRQAGQLAQLISAVNKLTMRAVPQQKPRRNRKNKKQKQKQQAPQNNTNQKKQPPKKKPAQKKKKPGRRERMCMKIENDCIFEVKHEGKVTGYACLVGDKVMKPAHVKGTIDNADLAKLAFKRSSKYDLECAQIPVHMKSDASKFTHEKPEGYYNWHHGAVQYSGGRFTIPTGAGKPGDSGRPIFDNKGRVVAIVLGGANEGARTALSVVTWNKDIVTKITPEGAEEWSLAIPVMCLLANTTFPCSQPPCTPCCYEKEPEETLRMLEDNVMRPGYYQLLQASLTCSPHRQRRSTKDNFNVYKATRPYLAHCPDCGEGHSCHSPVALERIRNEATDGTLKIQVSLQIGIKTDDSHDWTKLRYMDNHMPADAERAGLFVRTSAPCTITGTMGHFILARCPKGETLTVGFTDSRKISHSCTHPFHHDPPVIGREKFHSRPQHGKELPCSTYVQSTAATTEEIEVHMPPDTPDRTLMSQQSGNVKITVNGQTVRYKCNCGGSNEGLTTTDKVINNCKVDQCHAAVTNHKKWQYNSPLVPRNAELGDRKGKIHIPFPLANATCRVPKARNPTVTYGKNQVIMLLYPDHPTLLSYRNMGEEPNYQEEWVMHKKEVVLTVPTEGLEVTWGNNEPYKYWPQLSTNGTAHGHPHEIILYYYELYPTMTVVVVSVATFILLSMVGMAAGMCMCARRRCITPYELTPGATVPFLLSLICCIRTAKAATYQEAAIYLWNEQQPLFWLQALIPLAALIVLCNCLRLLPCCCKTLAFLA

>ACM09909.1:1-798 structural polyprotein [Chikungunya virus]MEFIPTQTFYNRRYQPRPWTPRSTIQIIRPRPRPQRQAGQLAQLISAVNKLTMRAVPQQKPRRNRKNKKQKQKQQAPQNNTNQKKQPPKKKPAQKKKKPGRRERMCMKIENDCIFEVKHEGKVTGYACLVGDKVMKPAHVKGTIDNADLAKLAFKRSSKYDLECAQIPVHMKSDASKFTHEKPEGYYNWHHGAVQYSGGRFTIPTGAGKPGDSGRPIFDNKGRVVAIVLGGANEGARTALSVVTWNKDIVTKITPEGAEEWSLAIPVMCLLANTTFPCSQPPCTPCCYEKEPEETLRMLEDNVMRPGYYQLLQASLTCSPHRQRRSTKDNFNVYKATRPYLAHCPDCGEGHSCHSPVALERIRNEATDGTLKIQVSLQIGIKTDDSHDWTKLRYMDNHMPADAERAGLFVRTSAPCTITGTMGHFILARCPKGETLTVGFTDSRKISHSCTHPFHHDPPVIGREKFYSRPQHGKELPCSTYVQSTAATTEEIEVHMPPDTPDRTLMSQQSGNVKITVNGQTVRYKCNCGGSNEGLTTTDKVINNCKVDQCHAAVTNHKKWQYNSPLVPRNAELGDRKGKIHIPFPLANVTCRVPKARNPTVTYGKNQVIMLLYPDHPTLLSYRNMGEEPNYQEEWVMHKKEVVLTVPTEGLEVTWGNNEPYKYWPQLSTNGTAHGHPHEIILYYYELYPTMTVVVVSVATFILLSMVGMAAGMCMCARRRCITPYELTPGATVPFLLSLICCIRTAKAATYQEAAIYLWNEQQPLFWLQALIPLAALIVLCNCLRLLPCCCKTLAFLA

>ASM47567.1:1-798 structural polyprotein, partial [Chikungunya virus]MEYIPTQTFYNRRYQPRPWTPRPTIQVIRPRPRPQRQAGQLAQLISAVNKLTMRAVPQQKPRRNRKNKKQKQKRQAPQNNTNQKKQPPKKKPAQKKKKPGRRERMCMKIENDCIFEVKYEGKVTGYACLVGDKVMKPAHVKGTIDNADLAKLAFKRSSKYDLECAQIPVHMKSDASKFTHEKPEGYYNWHHGAVQYSGGRFTIPTGAGKPGDSGRPIFDNKGRVVAIVLGGANEGARTALSVVTWNKDIVTKITPEGAEEWSLAIPVMCLLANTTFPCSQPPCTPCCYEREPEETLRMLEDNVMRPGYYQLLQASLTCSPHRQRRSTKDNFNVYKATRPYLAHCPDCGEGHSCHSPVALERIRNEATDGTLKIQVSLQIGIKTDDSHDWTKLRYMDNHTPADAERAGLFVRTSAPCTITGTMGHFILTRCPKGETLTVGFTDSRKISHSCTHPFHHDPPVIGREKFHSRPQHGKELPCSTYVQSTAATTEEIEVHMPPDTPDRTLMSQQSGNVKITVNGQTVRYKCNCGGSNEGLITTDKVINNCKVDQCHAAVTNHKKWQYNSPLVPRNAELGDRKGKIHIPFPLANVTCRVPKARNPTVTYGKNQVIMLLYPDHPTLLSYRNMGEEPNYQEEWVTHKKEVVLTVPTEGLEVTWGNNEPYKYWPQLSTNGTAHGHPHEIILYYYELYPTMTVVVVSVASFVLLSMVGVAVGMCMCARRRCITPYELTPGATVPFLLSLICCIRTAKAATYQEAAVYLWNEQQPLFWLQALIPLAALIVLCNCLRLLPCCCKTLAFLA

>QCC38224.1:1-798 structural polyprotein [Chikungunya virus]MEFIPTQTFYNRRYQPRPWTPRSTIQIIRPRPRPQRQAGQLAQLISAVNKLTMRAVPQQKPRRNRKNKKQKQKQQAPQNNTNQKKQPPKKKPAQKKKKPGRRERMCMKIENDCIFEVKHEGKVTGYACLVGDKVMKPAHVKGTIDNADLAKLAFKRSSKYDLECAQIPVHMKSDASKFTHEKPEGYYNWHHGAVQYSGGRFTIPTGAGKPGDSGRPIFDNKGRVVAIVLGGANEGARTALSVVTWNKDIVTKITPEGAEEWSLAIPVMCLLANTTFPCSQPPCTPCCYEKEPEETLRMLEDNVMRPGYYQLLQASLTCSPHRQRRSTKDNFNVYKATRPYLAHCPDCGEGHSCHSPVALERVRNEATDGTLKIQVSLQIGIKTDDSHDWTKLRYMDNHMPADAERAGLFVRTSAPCTITGTMGHFILARCPKGETLTVGFTDSRKISHSCTHPFHHDPPVIGREKFHSRPQHGKELPCSTYVQSTAATTEEIEVHMPPDTPDRTLMSQQSGNVKITVNGQTVRYKCNCGGSNEGLTTTDKVINNCKVDQCHAAVTNHKKWQYNSPLVPRNAELGDRKGKIHIPFPLANATCRVPKARNPTVTYGKNQVIMLLYPDHPTLLSYRNMGEEPNYQEEWVMHKKEVVLTVPTEGLEVTWGNNEPYKYWPQLSTNGTAHGHPHEIILYYYELYPTMTVVVVSVATFILLSMVGMAAGMCMCARRRCITPYELTPGATVPFLLSLICCIRTAKAATYQEAAIYLWNEQQPLFWLQALIPLAALIVLCNCLRLLPCCCKTLAFLA

>ABN04196.1:1-798 structural polyprotein [Chikungunya virus]MEFIPTQTFYNRRYQPRPWTPRSTIQIIRPRPRPQRQAGQLAQLISAVNKLTMRAVPQQKPRRNRKNKKQKQKQQAPQNDTNQKKQPPKKKPAQKKKKPGRRERMCMKIENDCIFEVKHEGKVTGYACLVGDKVMKPAHVKGTIDNADLAKLAFKRSSKYDLECAQIPVHMKSDASKFTHEKPEGYYNWHHGAVQYSGGRFTIPTGAGKPGDSGRPIFDNKGRVVAIVLGGANEGARTALSVVTWNKDIVTKITPEGAEEWSLAIPVMCLLANTTFPCSQPPCTPCCYEKEPEETLRMLEDNVMRPGYYQLLQASLTCSPHRQRRSTKDNFNVYKATRPYLAHCPDCGEGHSCHSPVALERIRNEATDGTLKIQVSLQIGIKTDDSHDWTKLRYMDNHMPADAERAGLFVRTSAPCTITGTMGHFILARCPKGETLTVGFTDSRKISHSCTHPFHHDPPVIGREKFHSRPQHGKELPCSTYVQSTAATTEEIEVHMPPDTPDRTLMSQQSGNVKITVNGQTVRYKCNCGGSNEGLTTTDKVINNCKVDQCHAAVTNHKKWQYNSPLVPRNAELGDRKGKIHIPFPLANVTCRVPKARNPTVTYGKNQVIMLLYPDHPTLLSYRNMGEEPNYQEEWVMHKKEVVLTVPTEGLEVTWGNNEPYKYWPQLSTNGTAHGHPHEIILYYYELYPTMTVVVVSVATFILLSMVGMAAGMCMCARRRCITPYELTPGATVPFLLSLICCIRTAKAATYQEAAIYLWNEQQPLFWLQALIPLAALIVLCNCLRLLPCCCKTLAFLA

>ASM47569.1:1-798 structural polyprotein, partial [Chikungunya virus]MEYIPTQTFYNRRYQPRPWTPRPTIQVIRPRPRPQRQAGQLAQLISAVNKLTMRAVPQQKPRRNRKNKKQKQKRQAPQNNTNQKKQPPKKKPAQKKKKPGRRERMCMKIENDCIFEVKYEGKVTGYACLVGDKVMKPAHVKGTIDNADLAKLAFKRSSKYDLECAQIPVHMKSDASKFTHEKPEGYYNWHHGAVQYSGGRFTIPTGAGKPGDSGRPIFDNKGRVVAIVLGGANEGARTALSVVTWNKDIVTKITPEGAEEWSLAIPVMCLLANTTFPCSQPPCTPCCYEREPEETLRMLEDNVMRPGYYQLLQASLTCSPHRQRRSTKDNFNVYKATRPYLAHCPDCGEGHSCHSPVALERIRNEATDGTLKIQVSLQIGIKTDDSHDWTKLRYMDNHTPADAERAGLFVRTSAPCTITGTMGHFILTRCPKGETLTVGFTDSRKISHSCTHPFHHDPPVIGREKFHSRPQHGKELPCSTYVQSTAATTEEIEVHMPPDTPDRTLMSQQSGNVKITVNGQTVRYKCNCGGSNEGLITTDKVINNCKVDQCHAAVTNHKKWQYNSPLVPRNAELGDRKGKIHIPFPLANVTCRVPKARNPTVTYGKNQVIMLLYPDHPTLLSYRNMGEEPNYQEEWVTHKKEVVLTVPTEGLEVTWGNNEPYKYWPQLSTNGTAHGHPHEIILYYYELYPTMTVVVVSVASFVLLSMVGVAVGMCMCARRRCITPYELTPGATVPFLLSLICCIRTAKAATYQEAAVYLWNEQQPLFWLQALIPLAALIVLCNCLRLLPCCCKTLAFLA

>QOW97278.1:1-798 structural polyprotein, partial [Chikungunya virus]MEYIPTQTFYNRRYQPRPWTPRPTIQVIRPRPRPQRQAGQLAQLISAVNKLTMRAVPQQKPRRNRKNKKQKQKRQAPQNNTNQKKQPPKKKPAQKKKKPGRRERMCMKIENDCIFEVKYEGKVTGYACLVGDKVMKPAHVKGTIDNADLAKLAFKRSSKYDLECAQIPVHMKSDASKFTHEKPEGYYNWHHGAVQYSGGRFTIPTGAGKPGDSGRPIFDNKGRVVAIVLGGANEGARTALSVVTWNKDIVTKITPEGAEEWSLAIPVMCLLANTTFPCSQPPCTPCCYEREPEETLRMLEDNVMRPGYYQLLQASLTCSPHRQRRSTKDNFNVYKATRPYLAHCPDCGEGHSCHSPVALERIRNEATDGTLKIQVSLQIGIKTDDSHDWTKLRYMDNHTPADAERAGLFVRTSAPCTITGTMGHFILTRCPKGETLTVGFTDSRKISHSCTHPFHHDPPVIGREKFHSRPQHGKELPCSTYVQSTAATTEEIEVHMPPDTPDRTLMSQQSGNVKITVNGQTVRYKCNCGGSNEGLITTDKVINNCKVDQCHAAVTNHKKWQYNSPLVPRNAELGDRKGKIHIPFPLANVTCRVPKARNPTVTYGKNQVIMLLYPDHPTLLSYRNMGEEPNYQEEWVTHKKEVVLTVPTEGLEVTWGNNEPYKYWPQLSTNGTAHGHPHEIILYYYELYPTMTVVVVSVASFILLSMVGVAVGMCMCARRRCITPYELTPGATVPFLLSLICCIRTAKAATYQEAAVYLWNEQQPLFWLQALIPLAALIVLCNCLRLLPCCCKTLAFLA

>QEP54722.1:1-798 structural polyprotein [Chikungunya virus]MEFIPTQTFYNRRYQPRPWTPRSTIQIIRPRPRPQRQAGQLAQLISAVNKLTMRAVPQQKPRRNRKNKKQKQKQQAPQNNTNQKKQPPKKKPAQKKKKPGRRERMCMKIENDCIFEVKHEGKVTGYACLVGDKVMKPAHVKGTIDNADLAKLAFKRSSKYDLECAQIPVHMKSDASKFTHEKPEGYYNWHHGAVQYSGGRFTIPTGAGKPGDSGRPIFDNKGRVVAIVLGGANEGARTALSVVTWNKDIVTKITPEGAEEWSLAIPVMCLLANTTFPCSQPPCTPCCYEKEPEETLRMLEDNVMRPGYYQLLQASLTCSPHRQRRSTKDNFNVYKATRPYLAHCPDCGEGHSCHSPVALERIRNEATDGTLKIQVSLQIGIKTDDSHDWTKLRYMDNHMPADAERAGLFVRTSAPCTITGTMGHFILARCPKGETLTVGFTDSRKISHSCTHPFHHDPPVIGREKFHSRPQHGKELPCSTYVQSTAATTEEIEVHMPPDTPDRTLMSQQSGNVKITVNGQTVRYKCNCGGSNEGLTTTDKVINNCKVDQCHAAVTNHKKWQYNSPLVPRNAELGDRKGKIHIPFPLANATCRVPKARNPTVTYGKNQVIMLLYPDHPTLLSYRNMGEEPNYQEEWVMHKKEVVLTVPTEGLEVTWGNNEPYKYWPQLSTNGTAHGHPHEIILYYYELYPTMTVVVVSVATFILLSMVGMAAGMCMCARRRCITPYELTPGATVPFLLSLICCIRTAKAATYQEAAIYLWNEQQPLFWLQALIPLAALIVLCNCLRLLPCCCKTLAFLA

>QCC38214.1:1-798 structural polyprotein [Chikungunya virus]MEFIPTQTFYNRRYQPRPWTPRSTIQIIRPRPRPQRQAGQLAQLISAVNKLTMRAVPQQKPRRNRKNKKQKQKQQAPQNNTNQKKQPPKKKPAQKKKKPGRRERMCMKIENDCIFEVKHEGKVTGYACLVGDKVMKPAHVKGTIDNADLAKLAFKRSSKYDLECAQIPVHMKSDASKFTHEKPEGYYNWHHGAVQYSGGRFTIPTGAGKPGDSGRPIFDNKGRVVAIVLGGANEGARTALSVVTWNKDIVTKITPEGAEEWSLAIPVMCLLANTTFPCSQPPCTPCCYEKEPEETLRMLEDNVMRPGYYQLLQASLTCSPHRQRRSTKDNFNVYKATRPYLAHCPDCGEGHSCHSPVALERIRNEATDGTLKIQVSLQIGIKTDDSHDWTKLRYMDNHMPADAERAGLFVRTSAPCTITGTMGHFILARCPKGETLTVGFTDSRKISHSCTHPFHHDPPVIGREKFHSRPQHGKELPCSTYVQSTAATTEEIEVHMPPDTPDRTLMSQQSGNVKITVNGQTVRYKCNCGGSNEGLTTTDKVINNCKVDQCHAAVTNHKKWQYNSPLVPRNAELGDRKGKIHIPFPLANATCRVPKARNPTVTYGKNQVIMLLYPDHPTLLSYRNMGEEPNYQEEWVMHKKEVVLTVPTEGLEVTWGNNEPYKYWPQLSTNGTAHGHPHEIILYYYELYPTMTVVVVSVATFILLSMVGMAAGMCMCARRRCITPYELTPGATVPFLLSLICCIRTAKAATYQEAAIYLWNEQQPLFWLQALIPLAALIVLCNCLRLLPCCCKTLAFLA

>QOW97290.1:1-798 structural polyprotein, partial [Chikungunya virus]MEYIPTQTFYNRRYQPRPWTPRPTIQVIRPRPRPQRQAGQLAQLISAVNKLTMRAVPQQKPRRNRKNKKQKQKRQAPQNNTNQKKQPPKKKPAQKKKKPGRRERMCMKIENDCIFEVKYEGKVTGYACLVGDKVMKPAHVKGTIDNADLAKLAFKRSSKYDLECAQIPVHMKSDASKFTHEKPEGYYNWHHGAVQYSGGRFTIPTGAGKPGDSGRPIFDNKGRVVAIVLGGANEGARTALSVVTWNKDIVTKITPEGAEEWSLAIPVMCLLANTTFPCSQPPCTPCCYEREPEETLRMLEDNVMRPGYYQLLQASLTCSPHRQRRSTKDNFNVYKATRPYLAHCPDCGEGHSCHSPVALERIRNEATDGTLKIQVSLQIGIKTDDSHDWTKLRYMDNHMPADAERAGLFVRTSAPCTITGTMGHFILTRCPKGETLTVGFTDSRKISHSCTHPFHHDPPVIGREKFHSRPQHGKELPCSTYVQSTAATTEEIEVHMPPDTPDRTLMSQQSGNVKITVNGQTVRYKCNCGGSNEGLITTDKVINNCKVDQCHAAVTNHKKWQYNSPLVPRNAEFGDRKGKIHIPFPLANVTCRVPKARNPTVTYGKNQVIMLLYPDHPTLLSYRNMGEEPNYQEEWVTHKKEVVLTVPTEGLEVTWGNNEPYKYWPQLSTNGTAHGHPHEIILYYYELYPTMTVVVVSVASFILLSMVGVAVGMCMCARRRCITPYELTPGATVPFLLSLICCIRTAKAATYQEAAVYLWNEQQPLFWLQALIPLAALIVLCNCLRLLPCCCKTLAFLA

>QCC38236.1:1-798 structural polyprotein [Chikungunya virus]MEFIPTQTFYNRRYQPRPWTPRSTIQIIRPRPRPQRQAGQLAQLISAVNKLTMRAVPQQKPRRNRKNKKQKQKQQAPQNNTNQKKQPPKKKPAQKKKKPGRRERMCMKIENDCIFEVKHEGKVTGYACLVGDKVMKPAHVKGTIDNADLAKLAFKRSSKYDLECAQIPVHMKSDASKFTHEKPEGYYNWHHGAVQYSGGRFTIPTGAGKPGDSGRPIFDNKGRVVAIVLGGANEGARTALSVVTWNKDIVTKITPEGAEEWSLAIPVMCLLANTTFPCSQPPCTPCCYEKEPEETLRMLEDNVMRPGYYQLLQASLTCSPHRQRRSTKDNFNVYKATRPYLAHCPDCGEGHSCHSPVALERIRNEATDGTLKIQVSLQIGIKTDDSHDWTKLRYMDNHMPADAERAGLFVRTSAPCTITGTMGHFILARCPKGETLTVGFTDSRKISHSCTHPFHHDPPVIGREKFHSRPQHGKELPCSTYVQSTAATTEEIEVHMPPDTPDRTLMSQQSGNVKITVNGQTVRYKCNCGGSNEGLTTTDKVINNCKVDQCHAAVTNHKKWQYNSPLVPRNAELGDRKGKIHIPFPLANATCRVPKARNPTVTYGKNQVIMLLYPDHPTLLSYRNMGEEPNYQEEWVMHKKEVVLTVPTEGLEVTWGNNEPYKYWPQLSTNGTAHGHPHEIILYYYELYPTMTVVVVSVATFILLSMVGMAAGMCMCARRRCITPYELTPGATVPFLLSLICCIRTAKAATYQEAAIYLWNEQQPLFWLQALIPLAALIVLCNCLRLLPCCCKTLAFLA

>ANH22467.1:1-798 structural polyprotein [Chikungunya virus]MEFIPTQTFYNRRYQPRPWTPRSTIQIIRPRPRPQRQAGQLAQLISAVNKLTMRAVPQQKPRRNRKNKKQKQKQQAPQNNTNQKKQPPKKKPAQKKKKPGRRERMCMKIENDCIFEVKHEGKVTGYACLVGDKVMKPAHVKGTIDNADLAKLAFKRSSKYDLECAQIPVHMKSDASKFTHEKPEGYYNWHHGAVQYSGGRFTIPTGAGKPGDSGRPIFDNKGRVVAIVLGGANEGARTALSVVTWNKDIVTKITPEGAEEWSLAIPVMCLLANTTFPCSQPPCTPCCYEKEPEETLRMLEDNVMRPGYYQLLQASLTCSPHRQRRSTKNNFNVYKATRPYLAHCPDCGEGHSCHSPVALERIRNEATDGTLKIQVSLQIGIKTDDSHDWTKLRYMDNHMPADAERAGLFVRTSAPCTITGTMGHFILARCPKGETLTVGFTDSRKISHSCTHPFHHDPPVIGREKFHSRPQHGKELPCSTYVQSTAATTEEIEVHMPPDTPDRTLMSQQSGNVKITVNGQTVRYKCNCGGSNEGLTTTDKVINNCKVDQCHAAVTNHKKWQYNSPLVPRNAELGDRKGKIHIPFPLANVTCRVPKARNPTVTYGKNQVIMLLYPDHPTLLSYRNMGEEPNYQEEWVMHKKEVVLTVPTEGLEVTWGNNEPYKYWPQLSTNGTAHGHPHEIILYYYELYPTMTVVVVSVATFILLSMVGMAAGMCMCARRRCITPYELTPGATVPFLLSLICCIRTAKAATYQEAAIYLWNEQQPLFWLQALIPLAALIVLCNCLRLLPCCCKTLAFLA

>QEP54780.1:1-798 structural polyprotein [Chikungunya virus]MEFIPTQTFYNRRYQPRPWTPRSTIQIIRPRPRPQRQAGQLAQLISAVNKLTMRAVPQQKPRRNRKNKKQKQKQQAPQNNTNQKKQPPKKKPAQKKKKPGRRERMCMKIENDCIFEVKHEGKVTGYACLVGDKVMKPAHVKGTIDNADLAKLAFKRSSKYDLECAQIPVHMKSDASKFTHEKPEGYYNWHHGAVQYSGGRFTIPTGAGKPGDSGRPIFDNKGRVVAIVLGGANEGARTALSVVTWNKDIVTKITPEGAEEWSLAIPVMCLLANTTFPCSQPPCTPCCYEKEPEETLRMLEDNVMRPGYYQLLQASLTCSPHRQRRSTKDNFNVYKATRPYLAHCPDCGEGHSCHSPVALERIRNEATDGTLKIQVSLQIGIKTDDSHDWTKLRYMDNHMPADAERAGLFVRTSAPCTITGTMGHFILARCPKGETLTVGFTDSRKISHSCTHPFHHDPPVIGREKFHSRPQHGKELPCSTYVQSTAATTEEIEVHMPPDTPDRTLMSQQSGNVKITVNGQTVRYKCNCGGSNEGLTTTDKVINNCKVDQCHAAVTNHKKWQYNSPLVPRNAELGDRKGKIHIPFPLANATCRVPKARNPTVTYGKNQVIMLLYPDHPTLLSYRNMGEEPNYQEEWVMHKKEVVLTVPTEGLEVTWGNNEPYKYWPQLSTNGTAHGHPHEIILYYYELYPTMTVVVVSVATFILLSMVGMAAGMCMCARRRCITPYELTPGATVPFLLSLICCIRTAKAATYQEAAIYLWNEQQPLFWLQALIPLAALIVLCNCLRLLPCCCKTLAFLA

>BAP74237.1:1-798 structural polyprotein [Chikungunya virus]MEFIPTQTFYNRRYQPRPWTPRSTIQIIRPRPRPQRQAGQLAQLISAVNKLTMRAVPQQKPRRNRKNKKQKQKQQAPQNNTNQKKQPPKKKPAQKKKKPGRRERMCMKIENDCIFEVKHEGKVTGYACLVGDKVMKPAHVKGTIDNADLAKLAFKRSSKYDLECAQIPVHMKSDASKFTHEKPEGYYNWHHGAVQYSGGRFTIPTGAGKPGDSGRPIFDNKGRVVAIVLGGANEGARTALSVVTWNKDIVTKITPEGAEEWSLAIPVMCLLANTTFPCSQPPCTPCCYEKEPEETLRMLEDNVMRPGYYQLLQASLTCSPHRQRRSTKDNFNVYKATRPYLAHCPDCGEGHSCHSPVALERIRNEATDGTLKIQVSLQIGIKTDDSHDWTKLRYMDNHMPADAERAGLFVRTSAPCTITGTMGHFILARCPKGETLTVGFTDSRKISHSCTHPFHHDPPVIGREKFHSRPQHGKELPCSTYVQSTAATTEEIEVHMPPDTPDRTLMSQQSGNVKITVNGQTVRYKCNCGGSNEGLTTTDKVINNCKVDQCHAAVTNHKKWQYNSPLVPRNAELGDRQGKIHIPFPLANVTCRVPKARNPTVTYGKNQVIMLLYPDHPTLLSYRNMGEEPNYQEEWVMHKKEVVLTVPTEGLEVTWGNNEPYKYWPQLSTNGTAHGHPHEIILYYYELYPTMTVVVVSVATFILLSMVGMAAGMCMCARRRCITPYELTPGATVPFLLSLICCIRTAKAATYQEAAIYLWNEQQPLFWLQALIPLAALIVLCNCLRLLPCCCKTLAFLA

>AGT37250.1:1-798 structural polyprotein [Chikungunya virus]MEFIPTQTFYNRRYQPRPWTPRSTIQIIRPRPRPQRQAGQLAQLISAVNKLTMRAVPQQKPRRNRKNKKQKQKQQAPQNNTNQKKQPPKKKPAQKKKKPGRRERMCMKIENDCIFEVKHEGKVTGYACLVGDKVMKPAHVKGTIDNADLAKLAFKRSSKYDLECAQIPVHMKSDASKFTHEKPEGYYNWHHGAVQYSGGRFTIPTGAGKPGDSGRPIFDNKGRVVAIVLGGANEGARTALSVVTWNKDIVTKITPEGAEEWSLAIPVMCLLANTTFPCSQPPCTPCCYEKEPEETLRMLEDNVMRPGYYQLLQASLTCSPHRQRRSTKDNFNVYKATRPYLAHCPDCGEGHSCHSPVALERIRNEATDGTLKIQVSLQIGIKTDDSHDWTKLRYMDNHMPADAERAGLFVRTSAPCTITGTMGHFILARCPKGETLTVGFTDSRKISHSCTHPFHHDPPVIGREKFHSRPQHGKELPCSTYVQSTAATTEEIEVHMPPDTPDRTLMSQQSGNVKISVNGQTVRYKCNCGGSNEGLTTTDKVINNCKVDQCHAAVTNHKKWQYNSPLVPRNAELGDRQGKIHIPFPLANVTCRVPKARNPTVTYGKNQVIMLLYPDHPTLLSYRNMGEEPNYQEEWVMHKKEVVLTVPTEGLEVTWGNNEPYKYWPQLSTNGTAHGHPHEIILYYYELYPTMTVVVVSVATFILLSMVGMAAGMCMCARRRCITPYELTPGATVPFLLSLICCIRTAKAATYQEAAIYLWNEQQPLFWLQALIPLAALIVLCNCLRLLPCCCKTLAFLA

>ACY66838.1:1-798 structural polyprotein [Chikungunya virus]MEFIPTQTFYNRRYQPRPWTPRSTIQIIRPRPRPQRQAGQLAQLISAVNKLTMRAVPQQKPRRNRKNKKQKQKQQAPQNNTNQKKQPPKKKPAQKKKKPGRRERMCMKIENDCIFEVKHEGKVTGYACLVGDKVMKPAHVKGTIDNADLAKLAFKRSSKYDLECAQIPVHMKSDASKFTHEKPEGYYNWHHGAVQYSGGRFTIPTGAGKPGDSGRPIFDNKGRVVAIVLGGANEGARTALSVVTWNKDIVTKITPEGAEEWSLAIPVMCLLANTTFPCSQPPCTPCCYEKEPEETLRMLEDNVMRPGYYQLLQASLTCSPHRQRRSTKDNFNVYKATRPYLAHCPDCGEGHSCHSPVALERIRNEATDGTLKIQVSLQIGIKTDDSHDWTKLRYMDNHMPADAERAGLFVRTSAPCTITGTMGHFILARCPKGETLTVGFTDSRKISHSCTHPFHHDPPVIGREKFHSRPQHGKELPCSTYVQSTAATTEEIEVHMPPDTPDHTLMSQQSGNVKITVNGQTVRYKCNCGGSNEGLTTTDKVINNCKVDQCHAAVTNHKKWQYNSPLVPRNAELGDRKGKIHIPFPLANVTCRVPKARNPTVTYGKNQVIMLLYPDHPTLLSYRNMGEEPNYQEEWVMHKKEVVLTVPTEGLEVTWGNNEPYKYWPQLSTNGTAHGHPHEIILYYYELYPTMTVVVVSVATFILLSMVGMAAGMCMCARRRCITPYELTPGATVPFLLSLICCIRTAKAATYQEAAIYLWNEQQPLFWLQALIPLAALIVLCNCLRLLPCCCKTLAFLA

>QEP54774.1:1-798 structural polyprotein [Chikungunya virus]MEFIPTQTFYNRRYQPRPWTPRSTIQIIRPRPRPQRQAGQLAQLISAVNKLTMRAVPQQKPRRNRKNKKQKQKQQAPQNNTNQKKQPPKKKPAQKKKKPGRRERMCMKIENDCIFEVKHEGKVTGYACLVGDKVMKPAHVKGTIDNADLAKLAFKRSSKYDLECAQIPVHMKSDASKFTHEKPEGYYNWHHGAVQYSGGRFTIPTGAGKPGDSGRPIFDNKGRVVAIVLGGANEGARTALSVVTWNKDIVTKITPEGAEEWSLAIPVMCLLANTTFPCSQPPCTPCCYEKEPEETLRMLEDNVMRPGYYQLLQASLTCSPHRQRRSTKDNFNVYKATRPYLAHCPDCGEGHSCHSPVALERIRNEATDGTLKIQVSLQIGIKTDDSHDWTKLRYMDNHMPADAERAGLFVRTSAPCTITGTMGHFILARCPKGETLTVGFTDSRKISHSCTHPFHHDPPVIGREKFHSRPQHGKELPCSTYVQSTAATTEEIEVHMPPDTPDRTLMSQQSGNVKITVNGQTVRYKCNCGGSNEGLTTTDKVINNCKVDQCHAAVTNHKKWQYNSPLVPRNAELGDRKGKIHIPFPLANATCRVPKARNPTVTYGKNQVIMLLYPDHPTLLSYRNMGEEPNYQEEWVMHKKEVVLTVPTEGLEVTWGNNEPYKYWPQLSTNGTAHGHPHEIILYYYELYPTMTVVVVSVATFILLSMVGMAAGMCMCARRRCITPYELTPGATVPFLLSLICCIRTAKAATYQEAAIYLWNEQQPLFWLQALIPLAALIVLCNCLRLLPCCCKTLAFLA

>ACY25938.1:1-798 structural polyprotein [Chikungunya virus]MEFIPTQTFYNRRYQPRPWTPRSTIQIIRPRPRPQRQAGQLAQLISAVNKLTMRAVPQQKPRRNRKNKKQKQKQQAPQNNTNQKKQPPKKKPAQKKKKPGRRERMCMKIENDCIFEVKHEGKVTGYACLVGDKVMKPAHVKGTIDNADLAKLAFKRSSKYDLECAQIPVHMKSDASKFTHEKPEGYYNWHHGAVQYSGGRFTIPTGAGKPGDSGRPIFDNKGRVVAIVLGGANEGARTALSVVTWNKDIVTKITPEGAEEWSLAIPVMCLLANTTFPCSQPPCTPCCYEKEPEETLRMLEDNVMRPGYYQLLQASLTCSPHRQRRSTKDNFNVYKATRPYLAHCPDCGEGHSCHSPVALERIRNEATDGTLKIQVSLQIGIKTDDSHDWTKLRYMDNHMPADAERAGLFVRTSAPCTITGTMGHFILARCPKGETLTVGFTDSRKISHSCTHPFHHDPPVIGREKFHSRPQHGKELPCSTYVQSTAATTEEIEVHMPPDTPDRTLMSQQSGNVKITVNGQTVRYKCNCGGSNEGLTTTDKVINNCKVDQCHAAVTNHKKWQYNSPLVPRNAELGDRKGKIHIPFPLANVTCRVPKARNPTVTYGKNQVIMLLYPDHPTLLSYRNMGEEPNYQEEWVMHKKKVVLTVPTEGLEVTWGNNEPYKYWPQLSTNGTAHGHPHEIILYYYELYPTMTVVVVSVATFILLSMVGMAAGMCMCARRRCITPYELTPGATVPFLLSLICCIRTAKAATYQEAAIYLWNEQQPLFWLQALIPLAALIVLCNCLRLLPCCCKTLAFLA

>ACM09911.1:1-798 structural polyprotein [Chikungunya virus]MEFIPTQTFYNRRYQPRPWTPRSTIQIIRPRPRPQRQAGQLAQLISAVNKLTMRAVPQQKPRRNRKNKKQKQKQQAPQNNTNQKKQPPKKKPAQKKKKPGRRERMCMKIENDCIFEVKHEGKVTGYACLVGDKVMKPAHVKGTIDNADLAKLAFKRSSKYDLECAQIPVHMKSDASKFTHEKPEGYYNWHHGAVQYSGGRFTIPTGAGKPGDSGRPIFDNKGRVVAIVLGGANEGARTALSVVTWNKDIVTKITPEGAEEWSLAIPVMCLLANTTFPCSQPPCTPCCYEKEPEETLRMLEDNVMRPGYYQLLQASLTCSPHRQRRSTKDNFNVYKATRPYLAHCPDCGEGHSCHSPVALERIRNEATDGTLKIQVSLQIGIKTDDSHDWTKLRYMDNHMPADAERAGLFVRTSAPCTITGTMGHFILARCPKGETLTVGFTDSRKISHSCTHPFHHDPPVIGREKFYSRPQHGKELPCSTYVQSTAATTEEIEVHMPPDTPDRTLMSQQSGNVKITVNGQTVRYKCNCGGSNEGLTTTDKVINNCKVDQCHAAVTNHKKWQYNSPLVPRNAELGDRKGKIHIPFPLANVTCRVPKARNPTVTYGKNQVIMLLYPDHPTLLSYRNMGEEPNYQEEWVMHKKEVVLTVPTEGLEVTWGNNEPYKYWPQLSTNGTAHGHPHEIILYYYELYPTMTVVVVSVATFILLSMVGMAAGMCMCARRRCITPYELTPGATVPFLLSLICCIRTAKAATYQEAAIYLWNEQQPLFWLQALIPLAALIVLCNCLRLLPCCCKTLAFLA

>AJY53702.1:1-798 structural polyprotein [Chikungunya virus]MEYIPTQTFYNRRYQPRPWTPRPTIQVIRPRPRPQRQAGQLAQLISAVNKLTMRAVPQQKPRRNRKNKKQKQKRQAPQNNTNQKKQPPKKKPAQKKKKPGRRERMCMKIENDCIFEVKYEGKVTGYACLVGDKVMKPAHVKGTIDNADLAKLAFKRSSKYDLECAQIPVHMKSDASKFTHEKPEGYYNWHHGAVQYSGGRFTIPTGAGKPGDSGRPIFDNKGRVVAIVLGGANEGARTALSVVTWNKDIVTKITPEGAEEWSLAIPVMCLLANTTFPCSQPPCTPCCYEREPEETLRMLEDNVMRPGYYQLLQASLTCSPHRQRRSTKDNFNVYKATRPYLAHCPDCGEGHSCHSPVALERIRNEATDGTLKIQVSLQIGIKTDDSHDWTKLRYMDNHTPADAERAGLFVRTSAPCTITGTMGHFILTRCPKGETLTVGFTDSRKISHSCTHPFHHDPPVIGREKFHSRPQHGKELPCSTYVQSTAATTEEIEVHMPPDTPDRTLMSQQSGNVKITVNGQTVRYKCNCGGSNEGLITTDKVINNCKVDQCHAAVTNHKKWQYNSPLVPRNAELGDRKGKIHIPFPLANVTCRVPKARNPTVTYGKNQVIMLLYPDHPTLLSYRNMGEEPNYQEEWVTHKKEVVLTVPTEGLEVTWGNNEPYKYWPQLSTNGTAHGHPHEIILYYYELYPTMTVVVVSVASFVLLSMVGVAVGMCMCARRRCITPYELTPGATVPFLLSLICCIRTAKAATYQEAAVYLWNEQQPLFWLQALIPLAALIVLCNCLRLLPCCCKTLAFLA

>ASX98216.1:1-798 structural polyprotein [Chikungunya virus]MEYIPTQTFYNRRYQPRPWTPRPTIQVIRPRPRPQRQAGQLAQLISAVNKLTMRAVPQQKPRRNRKNKKQKQKRQAPQNNTNQKKQPPKKKPAQKKKKPGRRERMCMKIENDCIFEVKYEGKVTGYACLVGDKVMKPAHVKGTIDNADLAKLAFKRSSKYDLECAQIPVHMKSDASKFTHEKPEGYYNWHHGAVQYSGGRFTIPTGAGKPGDSGRPIFDNKGRVVAIVLGGANEGARTALSVVTWNKDIVTKITPEGAEEWSLAIPVMCLLANTTFPCSQPPCTPCCYEREPEETLRMLEDNVMRPGYYQLLQASLTCSPHRQRRSTKDNFNVYKATRPYLAHCPDCGEGHSCHSPVALERIRNEATDGTLKIQVSLQIGIKTDDSHDWTKLRYMDNHTPADAERAGLFVRTSAPCTITGTMGHFILTRCPKGETLTVGFTDSRKISHSCTHPFHHDPPVIGREKFHSRPQHGKELPCSTYVQSTAATTEEIEVHMPPDTPDRTLMSQQSGNVKITVNGQTVRYKCNCGGSNEGLITTDKVINNCKVDQCHAAVTNHKKWQYNSPLVPRNAELGDRKGKIHIPFPLANVTCRVPKARNPTVTYGKNQVIMLLYPDHPTLLSYRNMGEEPNYQEEWVTHKKEVVLTVPTEGLEVTWGNNEPYKYWPQLSTNGTAHGHPHEIILYYYELYPTMTVVVVSVASFVLLSMVGVAVGMCMCARRRCITPYELTPGATVPFLLSLICCIRTAKAATYQEAAVYLWNEQQPLFWLQALIPLAALIVLCNCLRLLPCCCKTLAFLA

>ACY25940.1:1-798 structural polyprotein [Chikungunya virus]MEFIPTQTFYNRRYQPRPWTPRSTIQIIRPRPRPQRQAGQLAQLISAVNKLTMRAVPQQKPRRNRKNKKQKQKQQAPQNNTNQKKQPPKKKPAQKKKKPGRRERMCMKIENDCIFEVKHEGKVTGYACLVGDKVMKPAHVKGTIDNADLAKLAFKRSSKYDLECAQIPVHMKSDASKFTHEKPEGYYNWHHGAVQYSGDRFTIPTGAGKPGDSGRPIFDNKGRVVAIVLGGANEGARTALSVVTWNKDIVTKITPEGAEEWSLAIPVMCLLANTTFPCSQPPCTPCCYEKEPEETLRMLEDNVMRPGYYQLLQASLTCSPHRQRRSTKDNFNVYKATRPYLAHCPDCGEGHSCHSPVALERIRNEATDGTLKIQVSLQIGIKTDDSHDWTKLRYMDNHMPADAERAGLFVRTSAPCTITGTMGHFILARCPKGETLTVGFTDSRKISHSCTHPFHHDPPVIGREKFHSRPQHGKELPCSTYVQSTAATTEEIEVHMPPDTPDRTLMSQQSGNVKITVNGQTVRYKCNCGGSNEGLTTTDKVINNCKVDQCHAAVTNHKKWQYNSPLVPRNAELGDRKGKIHIPFPLANVTCRVPKARNPTVTYGKNQVIMLLYPDHPTLLSYRNMGEEPNYQEEWVMHKKEVVLTVPTEGLEVTWGNNEPYKYWPQLSTNGTAHGHPHEIILYYYELYPTMTVVVVSVATFILLSMVGMAAGMCMCARRRCITPYELTPGATVPFLLSLICCIRTAKAATYQEAAIYLWNEQQPLFWLQALIPLAALIVLCNCLRLLPCCCKTLAFLA

>QNS30840.1:1-798 structural polyprotein, partial [Chikungunya virus]MEYIPTQTFYNRRYQPRPWTPRPTIQVIRPRPRPQRQAGQLAQLISAVNKLTMRAVPQQKPRRNRKNKKQKQKRQAPQNNTNQKKQPPKKKPAQKKKKPGRRERMCMKIENDCIFEVKYEGKVTGYACLVGDKVMKPAHVKGTIDNADLAKLAFKRSSKYDLECAQIPVHMKSDASKFTHEKPEGYYNWHHGAVQYSGGRFTIPTGAGKPGDSGRPIFDNKGRVVAIVLGGANEGARTALSVVTWNKDIVTKITPEGAEEWSLAIPVMCLLANTTFPCSQPPCIPCCYEREPEETLRMLEDNVMRPGYYQLLQASLTCSPRRQRRSTKDNFNVYKATRPYLAHCPDCGEGHSCHSPVALERIRNEATDGTLKIQVSLQIGIKTDDSHDWTKLRYMDNHMPADAERAGLFVRTSAPCTITGTMGHFILTRCPKGETLTVGFTDSRKISHSCTHPFHHDPPVIGREKFHSRPQHGKELPCSTYVQSTAATTEEIEVHMPPDTPDRTLMSQQSGNVKITVNGQTVRYKCNCGGSNEGLITTDKVINNCKVDQCHAAVTNHKKWQYNSPLVPRNAELGDRKGKIHIPFPLANVTCRVPKARNPTVTYGKNQVIMLLYPDHPTLLSYRNMGEEPNYQEEWVTHKKEVVLTVPTEGLEVTWGNNEPYKYWPQLSTNGTAHGHPHEIILYYYELYPTMTVVVVSVASFVLLSMVGVAVGMCMCARRRCITPYELTPGATVPFLLSLICCIRTAKAATYQEAAVYLWNEQQPLFWLQALIPLAALIVLCNCLRLLPCCCKTLAFLA

>QOW97262.1:1-798 structural polyprotein, partial [Chikungunya virus]MEYIPTQTFYNRRYQPRPWTPRPTIQVIRPRPRPQRQAGQLAQLISAVNKLTMRAVPQQKPRRNRKNKKQKQKRQAPQNNTNQKKQPPKKKPAQKKKKPGRRERMCMKIENDCIFEVKYEGKVTGYACLVGDKVMKPAHVKGTIDNADLAKLAFKRSSKYDLECAQIPVHMKSDASKFTHEKPEGYYNWHHGAVQYSGGRFTIPTGAGKPGDSGRPIFDNKGRVVAIVLGGANEGARTALSVVTWNKDIVTKITPEGAEEWSLAIPVMCLLANTTFPCSQPPCTPCCYEREPEETLRMLEDNVMRPGYYQLLQASLTCSPHRQRRSTKDNFNVYKATRPYLAHCPDCGEGHSCHSPVALERIRNEATDGTLKIQVSLQIGIKTDDSHDWTKLRYMDNHMPADAERAGLFVRTSAPCTITGTMGHFILTRCPKGETLTVGFTDSRKISHSCTHPFHHDPPVIGREKFHSRPQHGKELPCSTYVQSTAATTEEIEVHMPPDTPDRTLMSQQSGNVKITVNGQTVRYKCNCGGSNEGLITTDKVINNCKVDQCHAAVTNHKKWQYNSPLVPRNAEFGDRKGKIHIPFPLANVTCRVPKARNPTVTYGKNQVIMLLYPDHPTLLSYRNMGEEPNYQEEWVTHKKEVVLTVPTEGLEVTWGNNEPYKYWPQLSTNGTAHGHPHEIILYYYELYPTMTVVVVSVASFVLLSMVGVAVGMCMCARRRCITPYELTPGATVPFLLSLICCIRTAKAATYQEAAVYLWNEQQPLFWLQALIPLAALIVLCNCLRLLPCCCKTLAFLA

**All the representative sequences of MAYV from all variants/genotypes**

>NP_579970.1:1-1242 structural polyprotein [Mayaro virus]MDFLPTQVFYGRRWRPRMPPRPWRPRMPTMQRPDQQARQMQQLIAAVSTLALRQNAAAPQRGKKKQPRRKKPKPQPEKPKKQEQKPKQKKAPKRKPGRRERMCMKIEHDCIFEVKHEGKVTGYACLVGDKVMKPAHVPGVIDNADLARLSYKKSSKYDLECAQIPVAMKSDASKYTHEKPEGHYNWHYGAVQYTGGRFTVPTGVGKPGDSGRPIFDNKGPVVAIVLGGANEGTRTALSVVTWNKDMVTKITPEGTVEWAASTVTAMCLLTNISFPCFQPSCAPCCYEKGPEPTLRMLEENVNSEGYYDLLHAAVYCRNSSRSKRSTANHFNAYKLTRPYVAYCADCGMGHSCHSPAMIENIQADATDGTLKIQFASQIGLTKTDTHDHTKIRYAEGHDIAEAARSTLKVHSSSECTVTGTMGHFILAKCPPGERISVSFVDSKNEHRTCRIAYHHEQRLIGRERFTVRPHHGIELPCTTYQLTTAETSEEIDMHMPPDIPDRTILSQQSGNVKITVNGRTVRYSSSCGSQAVGTTTTDKTINSCTVDKCQAYVTSHTKWQFNSPFVPRRMQAERKGKVHIPFPLINTTCRVPLAPEALVRSGKREATLSLHPIHPTLLSYRTFGAERVFDEQWITAQTEVTIPVPVEGVEYQWGNHKPQRFVVALTTEGKAHGWPHEIIEYYYGLHPTTTIVVVIRVSVVVLLSFAASVYMCVVARTKCLTPYALTPGAVVPVTIGVLCCAPKAHAASFAEGMAYLWDNNQSMFWMELTGPLALLILATCCARSLLSCCKGSFLVAMSIGSAVASAYEHTAIIPNQVGFPYKAHVAREGYSPLTLQMQVIETSLEPTLNLEYITCDYKTKVPSPYVKCCGTAECRTQDKPEYKCAVFTGVYPFMWGGAYCFCDSENTQMSEAYVERADVCKHDHAAAYRAHTASLRAKIKVTYGTVNQTVEAYVNGDHAVTIAGTKFIFGPVSTPWTPFDTKILVYKGELYNQDFPRYGAGQPGRFGDIQSRTLDSRDLYANTGLKLARPAAGNIHVPYTQTPSGFKTWQKDRDSPLNAKAPFGCIIQTNPVRAMNCAVGNIPVSMDIADSAFTRLTDAPVISELTCTVSTCTHSSDFGGIAVLSYKVEKSGRCDIHSHSNVAVLQEVSIETEGRSVIHFSTASASPSFVVSVCSSRATCTAKCEPPKDHVVTYPANHNGVTLPDLSSTAMTWAQHLAGGVGLLIALAVLILVIVTCVTLRR

>ALI88665.1:1-1242 structural polyprotein [Mayaro virus]MDFLPTQVFYGRRWRPRMPPRPWRPRMPTMQRPDQQARQMRKLIAAVSTLALRQNAAAPQRGKKKQPRRKKPKPQPEKPKKQEQKPKQKKAPKRKPGRRERMCMKIEHDCIFEVKHEGKVTGYACLVGDKVMKPAHVPGVIDNADLARLSYKKSSKYDLECAQIPVAMKSDASKYTHEKPEGHYNWHYGAVQYTGGRFTVPTGVGKPGDSGRPIFDNKGRVVAIVLGGANEGTRTALSVVTWNKDMVTKITPEGTVEWAASTVTAMCLLTNISFPCFQPSCAPCCYEKGPEPTLRMLEENVNSEGYYDLLHAAVYCRNSSRSKRSTANHFNAYKLTRPYVAYCADCGMGHSCHSPAMIENIQADATDGTLKIQFASQIGLTKTDTHDHTKIRYAEGHDIAEAARSTLKVHSSSECTVTGTMGHFILAKCPPGEAISVSFVDSKNEHRTCRIAYHHEQRLIGRERFTVRPHHGIELPCTTYQLTTAETSEEIDMHMPPDIPDRTILSQQSGNVKITVNGRTVRYSCSCGSKPSGTTTTDKTINSCTVDKCQAYVTSHTKWQFNSPFVPRAEQAERKGKVHIPFPLINTTCRVPLAPEALVRSGKREATLSLHPIHPTLLSYRTLGAEPVFDEQWITAQTEVTIPVPVEGVEYQWGNHKPQRLWSQLTTEGKAHGWPHEIIEYYYGLHPTTTIVVVIAVSVVVLLSFAASVYMCVVARNKCLTPYALTPGAVVPVTIGVLCCAPKAHAASFAEGMAYLWDNNQSMFWMELTGPLALLILATCCARSLLSCCKGSFLVAMSIGSAVASAYEHTAIIPNQVGFPYKAHVAREGYSPLTLQMQVIETSLEPTLNLEYITCDYKTKVPSPYVKCCGTAECRTQDKPEYKCAVFTGVYPFMWGGAYCFCDSENTQMSEAYVERADVCKHDHAAAYRAHTASLRAKIKVTYGTVNQTVEAYVNGDHAVTIAGTKFIFGPVSTAWTPFDTKIVVYKGEVYNQDFPPYGAGQPGRFGDIQSRTLDSRDLYANTGLKLARPAAGNIHVPYTQTPSGFKTWQKDRDSPLNAKAPFGCIIQTNPVRAMNCAVGNIPVSMDIADSAFTRLTDAPVISELTCTVSTCTHSSDFGGIAVLSYKVEKSGRCDIHSHSNVAVLQEVSIETEGRSVIHFSTASASPSFVVSVCSSRATCTAKCEPPKDHVVIYPANHNGVTLPDLSSTAMTWAQHLAGGVGLLIALAVLILVIVTCVTLRR

>QDL88200.1:1-1242 structural polyprotein [Mayaro virus]MDFLPTQVFYGRRWRPRMPPRPWRPRMPTMQRPDQQARQMQQLIAAVSTLALRQNAAAPQRGKKKQPRRKKPKPQPEKPKKQEQKPKQKMAPKRKPGRRERMCMKIEHDCIFEVKHEGKVTGYACLVGDKVMKPAHVPGVIDNADLARLSYKKSSKYDLECAQIPVAMKSDASKYTHEKPEGHYNWHYGAVQYTGGRFTVPTGVGKPGDSGRPIFDNKGRVVAIVLGGANEGTRTALSVVTWNKDMVTKITPEGTVEWAASTVTAMCLLTNISFPCFQPSCAPCCYEKGPEPTLRMLEENVNSEGYYDLLHAAVYCRNSSRSKRSTANHFNAYKLTRPYVAYCADCGMGHSCHSPAMIENIQADATDGTLKIQFASQIGLTKTDTHDHTKIRYAEGHDIAEAARSTLKVHSSSECTVTGTMGHFILAKCPPGEAISVSFVDSKNEHRTCRIAYLHEQRLIGRERFTVRPHHGIELPCTTYQLTTAETSEEIDMHMPPDIPDRTILSQQSGNVKITVNGRTVRYSCSCGSKPSGTTTTDKTINSCTVDKCQAYVTSHTKWQFNSPFVPRAEQAERKGKVHIPFPLINTTCRVPLAPEALVRSGKREATLSLHPIHPTLLSYRTLGAEPVFDEQWITAQTEVTIPVPVEGVEYQWGNHKPQRLWSQLTTEGKAHGWPHEIIEYYYGLHPTTTIVVVIAVSVVVLLSFAASVYMCVVARNKCLTPYALTPGAVVPVTIGVLCCAPKAHAASFAEGMAYLWDNNQSMFWMELTGPLALLILATCCARSLLSCCKGSFLVAMSIGSAVASAYEHTAIIPNQVGFPYKAHVAREGYSPLTLQMQVIETSLEPTLNLEYITCDYKTKVPSPYVKCCGTAECRTQDKPEYKCAVFTGVYPFMWGGAYCFCDSENTQMSEAYVERADVCKHDHAAAYRAHTASLRAKIKVTYGTVNQTVEAYVNGDHAVTIAGTKFIFGPVSTAWTPFDTKIVVYKGEVYNQDFPPYGAGQPGRFGDIQSRTLDSRDLYANTGLKLARPAAGNIHVPYTQTPSGFKTWQKDRDSPLNAKAPFGCIIQTNPVRAMNCAVGNIPVSMDIADSAFTRLTDAPVISELTCTVSTCTHSSDFGGIAVLSYKVEKSGRCDIHSHSNVAVLQEVSIETEGRSVIHFSTASASPSFVVSVCSSRATCTAKCEPPKDHVVTYPANHNGVTLPDLSSTAMTWAQHLAGGVGLLIALAVLILVIVTCVTLRR

>ALI88668.1:1-1242 structural polyprotein [Mayaro virus]MDFLPTQVFYGRRWRPRMPPRPWRPRMPTMQRPDQQARQMQQLIAAVSTLALRQNAAAPQRGKKKQPRRKKPKPQPEKPKKQEQKPKQKKAPKRKPGRRERMCMKIEHDCIFEVKHEGKVTGYACLVGDKVMKPAHVPGVIDNADLARLSYKKSSKYDLECAQIPVAMKSDASKYTHEKPEGHYNWHYGAVQYTGGRFTVPTGVGKPGDSGRPIFDNKGRVVAIVLGGANEGTRTALSVVTWNKDMVTKITPEGTVEWAASTVTAMCLLTNISFPCFQPSCAPCCYEKGPEPTLRMLEENVNSEGYYDLLHAAVYCRNSSRSKRSTANHFNAYKLTRPYVAYCADCGMGHSCHSPAMIENIQADATDGTLKIQFASQIGLTKTDTHDHTKIRYAEGHDIAEAARSTLKVHSSSECTVTGTMGHFILAKCPPGEAISVSFVDSKNEHRTCRIAFHHEQRLIGRERFTVRPHHGIELPCTTYQLTTAETSEEIDMHMPPDIPDRTILSQQSGNVKITVNGRTVRYSCSCGSKPSGTTTTDKTINSCTVDKCQAYVTSHTKWQFNSPFVPRAEQAERKGKVHIPFPLINTTCRVPLAPEALVRSGKREATLSLHPIHPTLLSYRTLGAEPVFDEQWITAQTEVTIPVPVEGVEYQWGNHKPQRLWSQLTTEGKAHGWPHEIIEYYYGLHPTITIVVVIAVSVVVLLSLAASVYMCVVARNKCLTPYALTPGAVVPVTIGVLCCAPKAHAASFAEGMAYLWDNNQSMFWMELTGPLALLILTTCCARSLFSCCKGSFLVAMSIGSAVASAYEHTAIIPNQVGFPYKAHVAREGYSPLTLQMQVVETSLEPTLNLEYITCDYKTKVPSPYVKCCGTAECRTQDKPEYKCAVFTGVYPFMWGGAYCFCDSENTQMSEAYVERADVCKHDHAAAYRAHTASLRAKIKVTYGTVNQTVEAYVNGDHAVTIAGTKFIFGPVSTAWTPFDTKIVVYKGEVYNQDFPPYGAGQPGRFGDIQSRTLDSRDLYANTGLKLARPAAGNIHVPYTQTPSGFKTWQKDRDSPLNAKAPFGCIIQTNPVRAMNCAVGNIPVSMDIADSAFTRLTDAPVISELTCTVSTCTHSSDFGGIAVLSYKVEKSGRCDIHSHSNVAVLQEVSIETEGRSVIHFSTASAAPSFVVSVCSSRATCTAKCEPPKDHVVTYPANHNGVTLPDLSSTAMTWAQHLAGGVGLLIALAVLILVIVTCVTLRR

>ALJ56197.1:1-1242 structural polyprotein [Mayaro virus]MDFLPTQVFYGRRWRPRMPPRPWRPRMPTMQRPDQQARQMQQLIAAVSTLALRQNAAAPQRGKKKQPRRKKPKPQPEKPKKQEQKPKQKKAPKRKPGRRERMCMKIEHDCIFEVKHEGKVTGYACLVGDKVMKPAHVPGVIDNADLARLSYKKSSKYDLECAQIPVAMKSDASKYTHEKPEGHYNWHYGAVQYTGGRFTVPTGVGKPGDSGRPIFDNKGRVVAIVLGGANEGTRTALSVVTWNKDMVTKITPEGTVEWAASTVTAMCLLTNISFPCFQPSCAPCCYEKGPEPTLRMLEENVNSEGYYDLLHAAVYCRNSSRSKRSTANHFNAYKLTRPYVAYCADCGMGHSCHSPAMIENIQADATDGTLKIQFASQIGLTKTDTHDHTKIRYAEGHYIAEAARSTLKVHSSSECTVTGTMGHFILAKCPPGEAISVSFVDSKNEHRTCRIAFHHEQRLIGRERFTVRPHHGIELPCTTYQLTTAETSEEIDMHMPPDIPDRTILSQQSGNVKITVNGRTVRYSCSCGSKPSGTTTTDKTINSCTVDKCQAYVTSHTKWQFNSPFVPRAEQAERKGKVHIPFPLINTTCRVPLAPEALVRSGKREATLSLHPIHPTLLSYRTLGAEPVFDEQWITAQTEVTIPVPVEGVEYQWGNHKPQRLWSQLTTEGKAHGWPHEIIEYYYGLHPTITIVVVIAVSVVVLLSLAASVYMCVVARNKCLTPYALTPGAVVPVTIGVLCCAPKAHAASFAEGMAYLWDNNQSMFWMELTGPLALLILTTCCARSLFSCCKGSFLVAMSIGSAVASAYEHTAIIPNQVGFPYKAHVAREGYSPLTLQMQVVETSLEPTLNLEYITCDYKTKVPSPYVKCCGTAECRTQDKPEYKCAVFTGVYPFMWGGAYCFCDSENTQMSEAYVERADVCKHDHAAAYRAHTASLRAKIKVTYGTVNQTVEAYVNGDHAVTIAGTKFIFGPVSTAWTPFDTKIVVYKGEVYNQDFPPYGAGQPGRFGDIQSRTLDSRDLYANTGLKLARPAAGNIHVPYTQTPSGFKTWQKDRDSPLNAKAPFGCIIQTNPVRAMNCAVGNIPVSMDIADSAFTRLTDAPVISELTCTVSTCTHSSDFGGIAVLSYKVEKSGRCDIHSHSNVAVLQEVSIETEGRSVIHFSTASAAPSFVVSVCSSRATCTAKCEPPKDHVVTYPANHNGVTLPDLSSTAMTWAQHLAGGVGLLIALAVLILVIVTCVTLRR

>AXF50013.1:1-1242 structural polyprotein [Mayaro virus]MDFLPTQVFYGRRWRPRMPPRPWRPRMPTMQRPDQQARQMQQLIAAVSTLALRQNAAAPQRGKKKQPRRKKPKPQPEKPKKQEQKPKQKKAPKRKPGRRERMCMKIEHDCIFEVKHEGKVTGYACLVGDKVMKPAHVPGVIDNADLARLSYKKSSKYDLECAQIPVAMKSDASKYTHEKPEGHYNWHYGAVQYTGGRFTVPTGVGKPGDSGRPIFDNKGRVVAIVLGGANEGTRTALSVVTWNKDMVTKITPEGTVEWAASTVTAMCLLTNISFPCFQPSCAPCCYEKGPEPTLRMLEENVNSEGYYDLLHAAVYCRNSSRSKRSTANHFNAYKLTRPYVAYCADCGMGHSCHSPAMIENIQADATDGTLKIQFASQIGLTKTDTHDHTKIRYAEGHDIAEAARSTLKVHSSSECTVTGTMGHFILAKCPPGEAISVSFVDSKNEHRTCRIAFHHEQRLIGRERFTVRPHHGIELPCTTYQLTTAETSEEIDMHMPPDIPDRTILSQQSGNVKITVNGRTVRYSCSCGSKPSGTTTTDKTINSCTVDKCQAYVTSHTKWQFNSPFVPRAEQAERKGKVHIPFPLINTTCRVPLAPEALVRSGKREATLSLHPIHPTLLSYRTLGAEPVFDEQWITAQTEVTIPVPVEGVEYQWGNHKPQRLWSQLTTEGKAHGWPHEIIEYYYGLHPTITIVVVIAVSVVVLLSLAASVYMCVVARNKCLTPYALTPGAVVPVTIGVLCCAPKAHAASFAEGMAYLWDNNQSMFWMELTGPLALLILTTCCARSLFSCCKGSFLVAMSIGSAVASAYEHTAIIPNQVGFPYKAHVAREGYSPLTLQMQVVETSLEPTLNLEYITCDYKTKVPSPYVKCCGTAECRTQDKPEYKCAVFTGVYPFMWGGAYCFCDSENTQMSEAYVERADVCKHDHAAAYRAHTASLRAKIKVTYGTVNQTVEAYVNGDHAVTIAGTKFIFGPVSTAWTPFDTKIVVYKGEVYNQDFPPYGAGQPGRFGDIQSRTLDSRDLYANTGLKLARPAAGNIHVPYTQTPSGFKTWQKDRDSPLNAKAPFGCIIQTNPVRAMNCAVGNIPVSMDIADSAFTRLTDAPVISELTCTVSTCTHSSDFGGIAVLSYKVEKSGRCDIHSHSNVAVLQEVSIETEGRSVIHFSTASAAPSFVVSVCSSRATCTAKCEPPKDHVVTYPANHNGVTLPDLSSTAMTWAQHLAGGVGLLIALAVLSLAIVTCVTLRR

>ANY58848.1:1-1242 structural polyprotein [Mayaro virus]MDFLPTQVFYGRRWRPRMPPRPWRPRMPTMQRPDQQARQMQQLIAAVSTLALRQNAAAPQRGKKKQPRRKKPKPQPEKPKKQEQKPKQKKAPKRKPGRRERMCMKIEHDCIFEVKHEGKVTGYACLVGDKVMKPAHVPGVIDNADLARLSYKKSSKYDLECAQIPVAMKSDASKYTHEKPEGHYNWHYGAVQYTGGRFTVPTGVGKPGDSGRPIFDNKGRVVAIVLGGANEGTRTALSVVTWNKDMVTKITPEGTVEWAASTVTAMCLLANISFPCFQPSCAPCCYEKGPEPTLRMLEENVNSEGYYDLLHAAVYCRNSSRSKRSTANHFNAYKLTRPYVAYCADCGMGHSCHSPAMIENIQADATDGTLKIQFASQIGLTKTDTHDHTKIRYAEGHDIAEAARSTLKVHSSSECTVTGTMGHFILAKCPPGEAISVSFVDSKNEHRTCRIAFHHEQRLIGRERFTVRPHHGIELPCTTYQLTTAETSEEIDMHMPPDIPDRTILSQQSGNVKITVNGRTVRYSCSCGSKPSGTTTTDKTINSCTVDKCQAYVTSHTKWQFNSPFVPRAEQAERKGKVHIPFPLINTTCRVPLAPEALVRSGKREATLSLHPIHPTLLSYRTLGAEPVFDEQWITAQTEVTIPVPVEGVEYQWGNHKPQRLWSQLTTEGKAHGWPHEIIEYYYGLHPTTTIVVVVAVSVVVLLSFAASVYMCVVARNKCLTPYALTPGAVVPVTIGVLCCAPKAHAASFAEGMAYLWDNNQSMFWMELTGPLALLILTTCCARSLFSCCKGSFLVAMSIGSAVASAYEHTAIIPNQVGFPYKAHVAREGYSPLTLQMQVVETSLEPTLNLEYITCDYKTKVPSPYVKCCGTAECRTQDKPEYKCAVFTGVYPFMWGGAYCFCDSENTQMSEAYVERADVCKHDHAAAYRAHTASLRAKIKVTYGTVNQTVEAYVNGDHAVTIAGTKFIFGPVSTAWTPFDTKIVVYKGEVYNQDFPPYGAGQPGRFGDIQSRTLDSRDLYANTGLKLARPAAGNIHVPYTQTPSGFKTWQKDRDSPLNAKAPFGCTIQTNPVRAMNCAVGNIPVSMDIADSAFTRLTDAPVISELLCTVSTCTHSSDFGGIAVLSYKVEKSGRCDIHSHSNVAVLQEVSIEAEGRSVIHFSTASAAPSFIVSVCSSRATCTAKCEPPKDHVVTYPANHNGITLPDLSSTAMTWAQHLAGGVGLLIALAVLILVIVTCITLRR

>ASY08148.1:1-1242 structural polyprotein [Mayaro virus]MDFLPTQVFYGRRWRPRMPPRPWRPRMPTTQRPDQQARQMQQLIAAVSTLALRQNAAAPQRGKKKQPRRKKPKPQPEKPKKQEQKPKQKKAPKRKPGRRERMCMKIEHDCIFEVKHEGKVTGYACLVGDKVMKPAHVPGVIDNADLARLSYKKSSKYDLECAQIPVAMKSDASKYTHEKPEGHYNWHYGAVQYTGGRFTVPTGVGKPGDSGRPIFDNKGRVVAIVLGGANEGTRTALSVVTWNKDMVTKITPEGTVEWAASTVTAMCLLTNISFPCFQPSCAPCCYEKGPEPTLRMLEENVNSEGYYDLLHAAVYCRNSSRSKRSTANHFNAYKLTRPYVAYCADCGMGHSCHSPAMIENIQADATDGTLKIQFASQIGLTKTDTHDHTKIRYAEGHDIAEAARSTLKVHSSSECTVTGTMGHFILAKCPPGEAISVSFVDSKNEHRTCRIAYHHEQRLIGRERFTVRPHHGIELPCTTYQLTTAETSEEIDMHMPPDIPDRTILSQQSGNVKITVNGRTVRYSCSCGSKPSGTTTTDKTINSCTVDKCQAYVTSHTKWQFNSPFVPRAEQAERKGKVHIPFPLINTTCRVPLAPEALVRSGKREATLSLHPIHPTLLSYRTLGAEPVFDEQWITAQTEVTIPVPVEGVEYQWGNHKPQRLWSQLTTEGKAHGWPHEIIEYYYGLHPTITIVVVIAVSVVVLLSLAASVYMCVVARNKCLTPYALTPGAVVPVTIGVLCCAPKAHAASFAEGMAYLWDNNQSMFWMELTGPLALLILTTCCARSLLSCCKGSFLVAMSIGSAVASAYEHTAIIPNQVGFPYKAHVAREGYSPLTLQMQVVETSLEPTLNLEYITCDYKTKVPSPYVKCCGTAECRTQDKPEYKCAVFTGVYPFMWGGAYCFCDSENTQMSEAYVERADVCKHDHAAAYRAHTASLRAQIKVTYGTVNQTVEAYVNGDHAVTIAGTKFIFGPVSTAWTPFDTKIVVYKGEVYNQDFPPYGAGQPGRFGDIQSRTLDSRDLYANTGLKLARPAAGNIHVPYTQTPSGFKTWQKDRDSPLNAKAPFGCVIQTNPVRAMNCAVGNIPVSMDIADSAFTRLTDAPVISELTCTVSTCTHSSDFGGIAVLSYKVEKPGRCDIHSHSNVAVLQEVSIETEGRSVIHFSTASAAPSFVVSVCSSRATCTAKCEPPKDHVVTYPANHNGVTLPDLSSTAMTWAQHLAGGVGLLIVLAVLILVIVTCVTLRR

>QDL88203.1:1-1242 structural polyprotein [Mayaro virus]MDFLPTQVFYGRRWRPRMPPRPWRPRMPTIQRPDQQARQMQQLIAAVSTLALRQNAAAPQRGKKKQPRRKKPKPQPEKPKKQEQKPKQKKAPKRKPGRRERMCMKIEHDCIFEVKHEGKVTGYACLVGDKVMKPAHVPGVIDNADLARLSYKKSSKYDLECAQIPVAMKSDASKYTHEKPEGHYNWHYGAVQYTGGRFTVPTGVGKPGDSGRPIFDNKGRVVAIVLGGANEGTRTALSVVTWNKDMVTKITPEGTVEWAASTVTAMCLLSNISFPCFQPSCAPCCYEKGPEPTLRMLEENVNSEGYYDLLHAAVYCRNSSRMKRSTANHFNAYKLTRPYVAYCADCGMGHSCHSPAMIENIQADATDGTLKIQFASQIGLTKTDTHDHTKIRYAEGHDIAEAARSTLKVHSSSECTVTGTMGHFILAKCPPGEVISVSFVDSKNEHRTCRIAYHHEQRLIGRERFTVRPHHGIELPCTTYQLTTAETSEEIDMHMPPDVPDRTILSQQSGNVKITVNGRTVRYSCSCGSKPSGTTTTDKTINSCTVDKCQAYVTSHTKWQFNSPFVPRAEQAERKGKVHIPFPLINTTCRVPLAPEALVRSGKREATLSLHPIHPTLLSYRTLGAEPVFDEQWITAQTEVTIPVPVEGVEYQWGNHKPQRLWSQLTTEGKAHGWPHEIIEYYYGLHPTITIIVVIAVSVVVLLSVAASVYMCVVARNKCLTPYALTPGAVVPVTIGVLCCAPKAHAASFAEGMAYLWDNNQSMFWMELTGPLALLILTTCCARSLLSCCKGSFLVAMSIGSAVASAYEHTAIIPNQVGFPYKAHVAREGYSPLTLQMQVVETSLEPTLNLEYITCDYKTKVPSPYVKCCGTAECRTQDKPEYKCAVFTGVYPFMWGGAYCFCDSENTQMSEAYVERADVCKHDHAAAYRAHTASLRAKIKVTYGTVNQTVEAYVNGDHAVTIAGTKFIFGPVSTAWTPFDTKIVVYKGEVYNQDFPPYGAGQPGRFGDIQSRTLDSRDLYANTGLKLARPAAGNIHVPYTQTPSGFKTWQKDRDSPLNAKAPFGCIIQTNPVRAMNCAVGNIPVSMDIADSAFTRLTDAPVISELACTVSTCTHSSDFGGIAVLSYKVEKSGRCDIHSHSNVAVLQEVSIETEGRSVIHFSTASAAPSFVVSVCSSRATCTAKCEPPKDHVVTYPANHNGVTLPDLSSTAMTWAQHLAGGVGLLIALAVLILVIVTCVTLRR

>ALG64706.1:1-1242 structural polyprotein [Mayaro virus]MDFLPTQVFYGRRWRPRMPPRPWRPRMPTMQRPDQQARQMQQLIAAVSTLALRQNAAAPQRGKKKQPRRKKPKPQPEKPKKQEQKPKQKKAPKRKPGRRERMCMKIEHDCIFEVKHEGKVTGYACLVGDKVMKPAHVPGVIDNADLARLSYKKSSKYDLECAQIPVAMKSDASKYTHEKPEGHYNWHYGAVQYTGGRFTVPTGVGKPGDSGRPIFDNKGRVVAIVLGGANEGTRTALSVVTWNKDMVTKITPEGTVEWAASTVTAMCLLANISFPCFQPSCAPCCYEKGPEPTLRMLEENVNSEGYYDLLHAAVYCRNSSRSKRSTANHFNAYKLTRPYVAYCADCGMGHSCHSPAMIENIQADATDGTLKIQFASQIGLTKTDTHDHTKIRYAEGHDIAEAARSTLKVHSSSECTVTGTMGHFILAKCPPGEAISVSFVDSKNEHRTCRIAFHHEQRLIGRERFTVRPHHGIELPCTTYQLTTAETSEEIDMHMPPDIPDRTILSQQSGNVKITVNGRTVRYSCSCGSKPSGTTTTDKTINSCTVDKCQAYVTSHTKWQFNSPFVPRAEQAERKGKVHIPFPLINTTCRVPLAPEALVRSGKREATLSLHPIHPTLLSYRTLGAEPVFDEQWITAQTEVTIPVPVEGVEYQWGNHKPQRLWSQLTTEGKAHGWPHEIIEYYYGLHPTTTIVVVVAVSVVVLLSFAASVYMCVVARNKCLTPYALTPGAVVPVTIGVLCCAPKAHAASFAEGMAYLWDNNQSMFWMELTGPLALLILTTCCARSLFSCCKGSFLVAMSIGSAVASAYEHTAIIPNQVGFPYKAHVAREGYSPLTLQMQVVETSLEPTLNLEYITCDYKTKVPSPYVKCCGTAECRTQDKPEYKCAVFTGVYPFMWGGAYCFCDSENTQMSEAYVERADVCKHDHAAAYRAHTASLRAKIKVTYGTVNQTVEAYVNGDHAVTIAGTKFIFGPVSTAWTPFDTKIVVYKGEVYNQDFPPYGAGQPGRFGDIQSRTLDSRDLYANTGLKLARPAAGNIHVPYTQTPSGFKTWQKDRDSPLNAKAPFGCTIQTNPVRAMNCAVGNIPVSMDIADSAFTRLTDAPVISELLCTVSTCTHSSDFGGIAVLSYKVEKAGRCDIHSHSNVAVLQEVSIEAEGRSVIHFSTASAAPSFIVSVCSSRATCTAKCEPPKDHVVTYPANHNGITLPDLSSTAMTWAQHLAGGVGLLIALAVLILVIVTCITLRR

>ALI88662.1:1-1243 structural polyprotein [Mayaro virus]MDFLPTQVFYGRRWRPRMPPRPWRPRMPTTQRPDQQARQMQQLIAAVSTLALRQNAAAPQRGKKKQPRRKKPKPQPEKPKKQEQKPKQKKAPKRKPGRRERMCMKIEHDCIFEVKHEGKVTGYACLVGDKVMKPAHVPGVIDNADLARLSYKKSSKYDLECAQIPVAMKSDASKYTHEKPEGHYNWHYGAVQYTGGRFTVPTGVGKPGDSGRPIFDNKGRVVAIVLGGANEGTRTALSVVTWNKDMVTKITPEGTVEWAASTVTAMCLLTNISFPCFQPSCAPCCYEKGPEPTLRMLEENVNSEGYYDLLHAAVYCRNSSRSKRSTANHFNAYKLTRPYVAYCADCGMGHSCHSPAMIENIQADATDGTLKIQFASQIGLTKTDTHDHTKIRYAEGHDIAEAARSTLKVHSSSECTVTGTMGHFILAKCPPGEAISVSFVDSKNEHRTCRIAYHHEQRLIGRERFTVRPHHGIELPCTTYQLTTAETSEEIDMHMPPDIPDRTILSQQSGNVKITVNGRTVRYSCSCGSKPSGTTTTDKTINSCTVDKCQAYVTSHTKWQFNSPFVPRAEQAERKGKVHIPFPLINTTCRVPLAPEALVRSGKREATLSLHPIHPTLLSYRTLGAEPVFDEQWITAQTEVTIPVPVEGVEYQWGNHKPQRLWSQLTTEGKAHGWPHEIIEYYYGLHPTITIVVVIAVQSVVVLLSLAASVYMCVVARNKCLTPYALTPGAVVPVTIGVLCCAPKAHAASFAEGMAYLWDNNQSMFWMELTGPLALLILTTCCARSLLSCCKGSFLVAMSIGSAVASAYEHTAIIPNQVGFPYKAHVAREGYSPLTLQMQVVETSLEPTLNLEYITCDYKTKVPSPYVKCCGTAECRTQDKPEYKCAVFTGVYPFMWGGAYCFCDSENTQMSEAYVERADVCKHDHAAAYRAHTASLRAQIKVTYGTVNQTVEAYVNGDHAVTIAGTKFIFGPVSTAWTPFDTKIVVYKGEVYNQDFPPYGAGQPGRFGDIQSRTLDSRDLYANTGLKLARPAAGNIHVPYTQTPSGFKTWQKDRDSPLNAKAPFGCVIQTNPVRAMNCAVGNIPVSMDIADSAFTRLTDAPVISELTCTVSTCTHSSDFGGIAVLSYKVEKPGRCDIHSHSNVAVLQEVSIETEGRSVIHFSTASAAPSFVVSVCSSRATCTAKCEPPKDHVVTYPANHNGVTLPDLSSTAMTWAQHLAGGVGLLIVLAVLILVIVTCVTLRR

>AHM95189.2:1-1242 structural polyprotein [Mayaro virus]MDFLPTQVFYGRRWRPRMPPRPWRPRPPTIQRPDQQARQMQQLIAAVSTLALRQNAAAPQRGRRKQPRRKKPKPQPEKPKKQEQKPKQKKTPKKKPGRRERMCMKIEHDCIFEVKHEGKVTGYACLVGDKVMKPAHVPGVIDNIDLARLSYKKSSKYDLECAQIPVAMKSDASKYTHEKPEGHYNWHYGAVQYTGGRFTVPTGVGKPGDSGRPIFDNKGRVVAIVLGGANEGARTALSVVTWNKDMVTKITPEGTEEWAAPTVTAMCLLANVSFPCFQPSCSPCCYEKGPEPTLRMLEENVNSEGYYELLHAAVYCKNSSRSKRSTANHFNAYKLTRPYVAYCADCGMGHSCHSPAMIENVQADATDGTLKIQFASQIGLTKTDTHDHTKIRYAEGHDIAEAARSTLKVHSSSECAVTGTMGHFILAKCPPGEVISVSFVDSKNEQRTCRIAYHHEQRLIGRERFTVRPHHGIELPCTTYQLTTAETSEEIDMHMPPDIPDRTILSQQSGNVKITVNGRTVKYSCSCGSKPSGTTTTDKTINSCTVDKCQAYVTSHTKWQFNSPFVPRAEQAERKGKVHIPFPLINTTCRVPLAPEALVRSGKREATLSLHPIHPTLLSYRTLGREPVFDEQWITTQTEVTIPVPVEGVEYRWGNHKPQRLWSQLTTEGRAHGWPHEIIEYYYGLHPTTTIVVVVAVTVVVLLSVAASVYMCVVARNKCLTPYALTPGAVVPVTIGVLCCAPKAHAASFAEGMAYLWDNNQSMFWMELTGPLALLILTTCCARSLLSCCKGSFLVAVSVGSAVASAYEHTAVIPNQVGFPYKAHVAREGYSPLTLQMQVVETSLEPTLNLEYITCDYKTKVPSPYVKCCGTAECRTQDKPEYKCAVFTGVYPFMWGGAYCFCDSENTQMSEAYVERADVCKHDYAAAYRAHTASLRAKIKVTYGTVNQTVEAYVNGDHAVTIAGTKFIFGPVSTAWTPFDTKIVVYKGEVYNQDFPPYGAGQPGRFGDIQSRTLDSKDLYANTGLKLARPAAGNIHVPYTQTPSGFKTWQKDRDSPLNAKAPFGCTIQTNPVRAMNCAVGNIPVSMDIADSAFTRLTDAPIISELLCTVSTCTHSSDFGGVAVLSYKVEKAGRCDVHSHSNVAVLQEVSIEAEGRSVIHFSTASAAPSFIVSVCSSRATCTAKCEPPKDHVVTYPANHNGITLPDLSSTAMTWAQHLAGGVGLLIALAVLILVIVTCITLRR

>ALI88644.1:1-1242 structural polyprotein [Mayaro virus]MDFLPTQVFYGRRWRPRMPPRPWRPRPPTIQRPDQQARQMQQLIAAVSTLALRQNAAAPQRGRKKQPRRKKPKPQPEKPKKQEQKPKQKKTPKKKPGKRERMCMKIEHDCIFEVKHEGKVTGYACLVGDKVMKPAHVPGVIDNVDLARLSYKKSSKYDLECAQIPVAMKSDASKYTHEKPEGHYNWHYGAVQYTGGRFTVPTGVGKPGDSGRPIFDNKGRVVAIVLGGANEGTRTALSVVTWNKDMVTKITPEGTEEWAAPTVTAMCLLANVSFPCFQPSCAPCCYEKGPEPTLRMLEENVNSEGYYELLHAAVYCKNSSRSKRSTANHFNAYKLTRPYVAYCADCGMGHSCHSPAMIENIQADATDGTLKIQFASQIGLTKTDTHDHTKIRYAEGHDIAEAARSTLKVHSSSECAVTGTMGHFILAKCPPGEVISVSFVDSKNEQRTCRIAYHHEQRLIGRERFTVRPHHGIELPCTTYQLTTAETSEEIDMHMPPDIPDRTILSQQSGNVKITVNGRTVKYSCSCGSKPSGTTTTDKTINSCTVDKCQAYVTSHTKWQFNSPFVPRAEQAERKGKVHIPFPLVNITCRVPLAPEALVRSGKREATLSLHPIHPTLLSYRTLGREPVFDEQWITTQTEVTIPVPVEGVEYQWGNHKPQRLWSQLTTEGRAHGWPHEIIEYYYGLHPTTTIVVVVAVSVVVLLSVAASVYMCVVARNKCLTPYALTPGAVVPVSIGVLCCAPKAHAASFAEGMAYLWDNNQSMFWMELTGPLALLILTTCCARSLLSCCKGSFLVAVSVGSAVASAYEHTAIIPNQVGFPYKAHVAREGYSPLTLQMQVVETSLEPTLNLEYITCDYKTKVPSPYVKCCGTAECRTQDKPEYKCAVFTGVYPFMWGGAYCFCDSENTQMSEAYVERADVCKHDYAAAYRAHTASLRAKIKVTYGTVNQTVEAYVNGDHAVTIAGTKFIFGPVSTAWTPFDTKIVVYKGEVYNQDFPPYGAGQPGRFGDIQSRTLDSKDLYANTGLKLARPAAGNIHVPYTQTPSGFKTWQRDRDSPLNAKAPFGCTIQTNPVRALNCAVGNIPVSMDIADSAFTRLTDAPIISELLCTVSTCTHSSDFGGVAVLSYKVEKSGRCDVHSHSNVAVLQEVSIEAEGRSVIHFSTASAAPSFIVSVCSSRATCTAKCEPPKDHVVTYPANHNGITLPDLSSTAMTWAQHLAGGVGLLIALAVIILVIVTCITMRR

>QDL88221.1:1-1242 structural polyprotein [Mayaro virus]MDFLPTQVFYGRRWRPRMPPRPWRPRPPTIQRPDQQARQMQQLIAAVSTLALRQNAAAPQRGRKKQPRRKKPKPQPEKPKKQEQKPKQKKTPKKKPGRRERMCMKIEHDCIFEVKHEGKVTGYACLVGDKVMKPAHVPGVIDNIDLARLSYKKSSKYDLECAQIPVAMKSDASKYTHEKPEGHYNWHYGAVQYTGGRFTVPTGVGKPGDSGRPIFDNKGRVVAIVLGGANEGARTALSVVTWNKDMVTKITPEGTEEWAAPTVTAMCLLANVSFPCFQPSCSPCCYEKGPEPTLRMLEENVNSEGYYELLHAAVYCKNSSRSKRSTANHFNAYKLTRPYVAYCADCGMGHSCHSPAMIENVQADATDGTLKIQFASQIGLTKTDTHDHTKIRYAEGHDIAEAARSTLKVHSSSECAVTGTMGHFILAKCPPGEVISVSFVDSKNEQRTCRIAYHHEQRLIGRERFTVRPHHGIELPCTTYQLTTAETSEEIDMHMPPDIPDRTILSQQSGNVKITVNGRTVKYSCSCGSKPSGTTTTDKTINSCTVDKCQAYVTSHTKWQFNSPFVPRAEQAERKGKVHIPFPLINTTCXVPLAPEALVRSGKREATLSLHPIHPTLLSYRTLGREPVFDEQWITTQTEVTIPVPVEGVEYRWGNHKPQRLWSQLTTEGRAHGWPHEIIEYYYGLHPTTTIVVVVAVSVVVLLSVAASVYMCVVARNKCLTPYALTPGAVVPVTIGVLCCAPKAHAASFAEGMAYLWDNNQSMFWMELTGPLALLILTTCCARSLFSCCKGSFLVAVSVGSAVASAYEHTAVIPNQVGFPYKAHVAREGYSPLTLQMQVVETSLEPTLNLEYITCDYKTKVPSPYVKCCGTAECRTQDKPEYKCAVFTGVYPFMWGGAYCFCDSENTQMSEAYVERADVCKHDYAAAYRAHTASLRAKIKVTYGTVNQTVEAYVNGDHAVTIAGTKFIFGPVSTAWTPFDTKIVVYKGEVYNQDFPPYGAGQPGRFGDIQSRTLDSKDLYANTGLKLARPAAGNIHVPYTQTPSGFKTWQKDRDSPLNARAPFGCTIQTNPVRAMNCAVGNIPVSMDIADSAFTRLTDAPIISELLCTVSTCTHSSDFGGVAVLSYKVEKAGRCDVHSHSNVAVLQEVSIEAEGRSVIHFSTASAAPSFIVSVCSSRATCTAKCEPPKDHVVTYPANHNGITLPDLSSTAMTWAQHLAGGVGLLITLAVLILVIVTCITLRR

>ALI88623.1:1-1242 structural polyprotein [Mayaro virus]MDFLPTQVFYGRRWRPRMPPRPWRPRPPTIQRPDQQARQMQQLIAAVSTLALRQNAAAPQRGRKKQPRRKKPKPQPEKPKKQEQKPKQKKTPKKKPGRRERMCMKIEHDCIFEVKHEGKVTGYACLVGDKVMKPAHVPGVIDNIDLARLSYKKSSKYDLECAQIPVAMKSDASKYTHEKPEGHYNWHYGAVQYTGGRFTVPTGVGKPGDSGRPIFDNKGRVVAIVLGGANEGARTALSVVTWNKDMVTKITPEGTEEWAAPTVTAMCLLANVSFPCFQPSCSPCCYEKGPEPTLRMLEENVNSEGYYELLHAAVYCKNSSRSKRSTANHFNAYKLTRPYVAYCADCGMGHSCHSPAMIENVQADATDGTLKIQFASQIGLTKTDTHDHAKIRYAEGHDIAEAARSTLKVHSSSECAVTGTMGHFILAKCPPGEVISVSFVDSKNEQRTCRIAYHHEQRLIGRERFTVRPHHGIELPCTTYQLTTAETSEEIDMHMPPDIPDRTILSQQSGNVKITVNGRTVKYSCSCGSKPSGTTTTDKTINSCTVDKCQAYVTSHTKWQFNSPFVPRAEQAERKGKVHIPFPLINTTCRVPLAPEALVRSGKREATLSLHPIHPTLLSYRTLGREPVFDEQWITTQTEVTIPVPVEGVEYRWGNHKPQRLWSQLTTEGRAHGWPHEIIEYYYGLHPTTTIVVVVAVSVVVLLSVAASVYMCVVARNKCLTPYALTPGAVVPVTIGVLCCAPKAHAASFAEGMAYLWDNNQSMFWMELTGPLALLILTTCCARSLFSCCKGSFLVAVSVGSAVASAYEHTAVIPNQVGFPYKAHVAREGYSPLTLQMQVVETSLEPTLNLEYITCDYKTKVPSPYVKCCGTAECRTQDKPEYKCAVFTGVYPFMWGGAYCFCDSENTQMSEAYVERADVCKHDYAAAYRAHTASLRAKIKVTYGTVNQTVEAYVNGDHAVTIAGTKFIFGPVSTAWTPFDTKIVVYKGEVYNQDFPPYGAGQPGRFGDIQSRTLDSKDLYANTGLKLARPAAGNIHVPYTQTPSGFKTWQKDRDSPLNARAPFGCTIQTNPVRAMNCAVGNIPVSMDIADSAFTRLTDAPIISELLCTVSTCTHSSDFGGVAVLSYKVEKAGRCDVHSHSNVAVLQEVSIEAEGRSVIHFSTASAAPSFIVSVCSSRATCAAKCEPPKDHVVTYPANHNGITLPDLSSTAMTWAQHLAGGVGLLITLAVLILVIVTCITLRR

>AJA37502.1:1-1242 structural polyprotein [Mayaro virus]MDFLPTQVFYGRRWRPRMPPRPWRPRPPTIQRPDQQARQMQQLIAAVSTLALRQNAAAPQRGRKRQPRRKKPKPQPEKPKKQEQKPKQKKTPKKKPGRRERMCMKIEHDCIFEVKHEGKVTGYACLVGDKVMKPAHVPGVIDNIDLARLSYKKSSKYDLECAQIPVAMKSDASKYTHEKPEGHYNWHYGAVQYTGGRFTVPTGVGKPGDSGRPIFDNKGRVVAIVLGGANEGARTALSVVTWNKDMVTKITPEGTEEWAAPTVTAMCLLANVSFPCFQPSCSPCCYEKGPEPTLRMLEENVNSEGYYELLHAAVYCKNSSRSKRSTANHFNAYKLTRPYVAYCADCGMGHSCHSPAMIENVQADATDGTLKIQFASQIGLTKTDTHDHTKIRYAEGHDIAEAARSTLKVHSSSECAVTGTMGHFILAKCPPGEVISVSFVDSKNEQRTCRIAYHHEQRLIGRERFTVRPHHGIELPCTTYQLTTAETSEEIDMHMPPDIPDRTILSQQSGNVKITVNGRTIKYSCSCGSKPSGTTTTDKIINSCTVDKCQAYVTSHTKWQFNSPFVPRAEQAERKGKVHIPFPLINTTCRVPLAPEALVRSGKREATLSLHPIHPTLLSYRTLGREPVFDEQWITTQTEVTIPVPVEGVEYRWGNHKPQRLWSQLTTEGRAHGWPHEIIEYYYGLHPTTTIVVVVAVSVVVLLSVAASVYMCVVARNKCLTPYALTPGAVVPVTIGVLCCAPKAHAASFAEGMAYLWDNNQSMFWMELTGPLALLILTTCCARSLFSCCKGSFLVAVSVGSAVASAYEHTAVIPNQVGFPYKAHVAREGYSPLTLQMQVVETSLEPTLNLEYITCDYKTKVPSPYVKCCGTAECRTQDKPEYKCAVFTGVYPFMWGGAYCFCDSENTQMSEAYVERADVCKHDYAAAYRAHTASLRAKIKVTYGTVNQTVEAYVNGDHAVTIAGTKFIFGPVSTAWTPFDTKIVVYKGEVYNQDFPPYGAGQPGRFGDIQSRTLDSKDLYANTGLKLARPAAGNIHVPYTQTPSGFKTWQKDRDSPLNAKAPFGCTIQTNPVRAMNCAVGNIPVSMDIADSAFTRLTDAPIISELLCTVSTCTHSSDFGGVAVLSYKVEKAGRCDVHSHSNVAVLQEVSIEAEGRSVIHFSTASAAPSFIVSVCSSRATCTAKCEPPKDHVVTYPANHNGITLPDLSSTAMTWAQHLAGGVGLLIALAVLILVIVTCITLRR

>ASY08169.1:1-1242 structural polyprotein [Mayaro virus]MDFLPTQVFYGRRWRPRMPSRPWRPRPPTIQRPDQQARQMQQLIAAVSTLALRQNAAAPQRGRKKQPRRKKPKPQPEKPKKQEQKPKQKKTPKKKPGRRERMCMKIEHDCIFEVKHEGKVTGYACLVGDKVMKPAHVPGVIDNIDLARLSYKKSSKYDLECAQIPVAMKSDASKYTHEKPEGHYNWHYGAVQYTGGRFTVPTGVGKPGDSGRPIFDNKGRVVAIVLGGANEGARTALSVVTWNKDMVTKITPEGTEEWAAPTVTAMCLLANVSFPCFQPSCSPCCYEKGPEPTLRMLEENVNSEGYYELLHAAVYCKNSSRSKRSTANHFNAYKLTRPYVAYCADCGMGHSCHSPAMIENVQADATDGTLKIQFASQIGLTKTDTHDHTKIRYAEGHDIAEAARSTLKVHSSSECAVTGTMGHFILAKCPPGEVISVSFVDSKNEQRTCRIAYHHEQRLIGRERFTVRPHHGIELPCTTYQLTTAETSEEIDMHMPPDIPDRTILSQQSGNVKITVNGRTVKYSCSCGSKPSGTTTTDKTINSCTVDKCQAYVTSHTKWQFNSPFVPRAEQAERKGKVHIPFPLINTTCRVPLAPEALVRSGKREATLSLHPIHPTLLSYRTLGREPVFDDQWITTQTEVTIPVPVEGVEYRWGNHKPQRLWSQLTTEGRAHGWPHEIIEYYYGLHPTTTIVVVVAVSVVVLLSVAASVYMCVVARNKCLTPYALTPGAVVPVTIGVLCCAPKAHAASFAEGMAYLWDNNQSMFWMELTGPLALLILTTCCARSLLSCCKGSFLVAMSVGSAVASAYEHTAVIPNQVGFPYKAHVAREGYSPLTLQMQVVETSLEPTLNLEYITCDYKTKVPSPYVKCCGTAECRTQDKPEYKCAVFTGVYPFMWGGAYCFCDSENTQMSEAYVERADVCKHDYAAAYRAHTASLRAKIKVTYGTVNQTVEAYVNGDHAVTIAGTKFIFGPVSTAWTPFDTKIVVYKGEVYNQDFPPYGAGQPGRFGDIQSRTLDSKDLYANTGLKLARPAAGNIHVPYTQTPSGFKTWQKDRDSPLNAKAPFGCTIQTNPVRAMNCAVGNIPVSMDIADSAFTRLTDAPIISELLCTVSTCTHSSDFGGVAVLSYKVEKAGRCDVHSHSNVAVLQEVSIEAEGRSVIHFSTASAAPSFIVSVCSSRATCTAKCEPPKDHVVTYPANHNGITLPDLSSTAMTWAQHLAGGVGLLIALAVLILVIVTCITLRR

>ASY08151.1:1-1242 structural polyprotein [Mayaro virus]MDFLPTQVFYGRRWRPRMPPRPWRPRPPTIQRPDQQARQMQQLIAAVSTLALRQNAAAPQRGRKKQPRRKKPKPQPEKPKKQEQKPKQKKTPKKKPGRRERMCMKIEHDCIFEVKHEGKVTGYACLVGDKVMKPAHVPGVIDNIDLARLSYKKSSKYDLECAQIPVAMKSDASKYTHEKPEGHYNWHYGAVQYTGGRFTVPTGVGKPGDSGRPIFDNKGRVVAIVLGGANEGARTALSVVTWNKDMVTKITPEGTEEWAAPTVTAMCLLANVSFPCFQPSCSPCCYEKGPEPTLRMLEENVNSEGYYELLHAAVYCKNSSRSKRSTANHFNAYKLTRPYVAYCADCGMGHSCHSPAMIENVQADATDGTLKIQFASQIGLTKTDTHDHTKIRYAEGHDIAEAARSTLKVHSSSECAVTGTMGHFILAKCPPGEVISVSFVDSKNEQRTCRIAYHHEQRLIGRERFTVRPHHGIELPCTTYQLTTAETSEEIDMHMPPDIPDRTILSQQSGNVKITVNGRTVKYSCSCGSKPSGTTTTDKTINSCTVDKCQAYVTSHTKWQFNSPFVPRAEQAERKGKVHIPFPLINTTCRVPLAPEALVRSGKREATLSLHPIHPTLLSYRTLGREPVFDEQWITTQTEVTIPVPVEGVEYRWGNHKPQRLWSQLTTEGRAHGWPHEIIEYYYGLHPTTTIVVVVAVSVVVLLSVAASVYMCVVARNKCLTPYALTPGAVVPVTIGVLCCAPKAHAASFAEGMAYLWDNNQSMFWMELTGPLALLILTTCCARSLLSCCKGSFLVAVSVGSAVASAYEHTAVIPNQVGFPYKAHVAREGYSPLTLQMQVVETSLEPTLNLEYITCDYKTKVPSPYVKCCGTAECRTQDKPEYKCAVFTGVYPFMWGGAYCFCDSENTQMSEAYVERADVCKHDYAAAYRAHTASLRAKIKVTYGTVNQTVEAYVNGDHAVTIAGTKFIFGPVSTAWTPFDTKIVVYKGEVYNQDFPPYGAGQPGRFGDIQSRTLDSKDLYANTGLKLARPAAGNIHVPYTQTPSGFKTWQKDRDSPLNAKAPFGCTIQTNPVRAMNCAVGNIPVSMDIADSAFTRLTDAPIISELLCTVSTCTHSSDFGGVAVLSYKVEKAGRCDVHSHSNVAVLQEVSIEAEGRSVIHFSTASAAPSFVVSVCSSRATCTAKCEPPKDHVVTYPANHNGITLPDLSSTAMTWAQHLAGGVGLLIALAVLILVIVTCITLRR

>ALI88629.1:1-1242 structural polyprotein [Mayaro virus]MDFLPTQVFYGRRWRPRMPPRPWRPRPPTIQRPDQQARQMQQLIAAVSTLALRQNAAAPQRGRKKQPRRKKPKPQPEKPKKQEQKPKQKKTPKKKPGRRERMCMKIEHDCIFEVKHEGKVTGYACLVGDKVMKPAHVPGVIDNIDLARLSYKKSSKYDLECAQIPVAMKSDASKYTHEKPEGHYNWHYGAVQYTGGRFTVPTGVGKPGDSGRPIFDNKGRVVAIVLGGANEGARTALSVVTWNKDMVTKITPEGTEEWAAPTVTAMCLLANVSFPCFQPSCSPCCYEKGPEPTLRMLEENVNSEGYYELLHAAVYCKNSSRSKRSTANHFNAYKLTRPYVAYCADCGMGHSCHSPAMIENVQADATDGTLKIQFASQIGLTKTDTHDHTKIRYAEGHDIAEAARSTLKVHSSSECAVTGTMGHFILAKCPPGEVISVSFVDSKNEQRTCRIAYHHEQRLIGRERFTVRPHHGIELPCTTYQLTTAETSEEIDMHMPPDIPDRTILSQQSGNVKITVNGRTVKYSCSCGSKPSGTTTTDKTINSCTVDKCQAYVTSHTKWQFNSPFVPRAEQAERKGKVHIPFPLINTTCRVPLAPEALVRSGKREATLSLHPIHPTLLSYRTLGREPVFDEQWITTQTEVTIPVPVEGVEYRWGNHKPQRLWSQLTTEGRAHGWPHEIIEYYYGLHPTTTIVVVVAASVVVLLSVAASVYMCVVARNKCLTPYALTPGAVVPVTIGVLCCAPKAHAASFAEGMAYLWDNNQSMFWMELTGPLALLILTTCCARSLLSCCKGSFLVAVSVGSAVASAYEHTAVIPNQVGFPYKAHVAREGYSPLTLQMQVVETSLEPTLNLEYITCDYKTKVPSPYVKCCGTAECRTQDKPEYKCAVFTGVYPFMWGGAYCFCDSENTQMSEAYVERADVCKHDYAAAYRAHTASLRAKIKVTYGTVNQTVEAYVNGDHAVTIAGTKFIFGPVSTAWTPFDTKIVVYKGEVYNQDFPPYGAGQPGRFGDIQSRTLDSKDLYANTGLKLARPAAGNIHVPYTQTPSGFKTWQKDRDSPLNAKAPFGCTIQTNPVRAMNCAVGNIPVSMDIADSAFTRLTDAPIISELLCTVSTCTHSSDFGGVAVLSYKVEKAGRCDVHSHSNVAVLQEVSIEAEGRSVIHFSTASAAPSFIVSVCSSRATCTAKCEPPKDHVVTYPANHNGITLPDLSSTAMTWAQHLAGGVGLLIALAVLILVIVTCITLRR

>ALI88632.1:1-1242 structural polyprotein [Mayaro virus]MDFLPTQVFYGRRWRPRMPPRPWRPRPPTIQRPDQQARQMQQLIAAVSTLALRQNAAAPQRGRKKQPRRKKPKPQPEKPKKQEQKPKQKKTPKKKPGRRERMCMKIEHDCIFEVKHEGKVTGYACLVGDKVMKPAHVPGVIDNIDLARLSYKKSSKYDLECAQIPVAMKSDASKYTHEKPEGHYNWHYGAVQYTGGRFTVPTGVGKPGDSGRPIFDNKGRVVAIVLGGANEGARTALSVVTWNKDMVTKITPEGTEEWAAPTVTAMCLLANVSFPCFQPSCSPCCYEKGPEPTLRMLEENVNSEGYYELLHAAVYCKNSSRSKRSTANHFNAYKLTRPYVAYCADCGMGHSCHSPAMIENVQADATDGTLKIQFASQIGLTKTDTHDHTKIRYAEGHDIAEAARSTLKVHSSSECAVTGTMGHFILAKCPPGEVISVSFVDSKNEQRTCRIAYHHEQRLIGRERFTVRPHHGIELPCTTYQLTTAETSEEIDMHMPPDIPDRTILSQQSGNVKITVNGRTVKYSCSCGSKPSGTTTTDKTINSCTVDKCQAYVTSHTKWQFNSPFVPRAEQAERKGKVHIPFPLINTTCRVPLAPEALVRSGKREATLSLHPIHPTLLSYRTLGREPVFDEQWITTQTEVTIPVPVEGVEYRWGNHKPQRLWSQLTTEGRAHGWPHEIIEYYYGLHPTTTIVVVVAVSVVVLLSVAASVYMCVVARNKCLTPYALTPGAVVPVTIGVLCCAPKAHAASFAEGMAYLWDNNQSMFWMELTGPLALLILTTCCARSLLSCCKGSFLVAVSVGSAVASAYEHTAVIPNQVGFPYKAHVAREGYSPLTLQMQVVETSLEPTLNLEYITCDYKTKVPSPYVKCCGTAECRTQDKPEYKCAVFTGVYPFMWGGAYCFCDSENTQMSEAYVERADVCKHDYAAAYRAHTASLRAKIKVTYGTVNQTVEAYVNGDHAVTIAGTKFIFGPVSTAWTPFDTKIVVYKGEVYNQDFPPYGAGQPGRFGDIQSRTLDSKDLYANTGLKLARPAAGNIHVPYTQTPSGFKTWQKDRDSPLNAKAPFGCTIQTNPVRAMNCAVGNIPVSMDIADSAFTRLTDAPIISELLCTVSTCTHSSDFGGVAVLSYKVEKAGRCDVHSHSNVAVLQEVSIEAEGRSVIHFSTASAAPSFIVSVCSSRATCTAKCEPPKDHVVTYPANHNGITLPDLSSTAMTWAQHLAGGVGLLIALAVLILVIVTCITLRR

>ALI88617.1:1-1242 structural polyprotein [Mayaro virus]MDFLPTQVFYGRRWRPRMPPRPWRPRPPTIQRPDQQARQMQQLIAAVSTLALRQNAAAPQRGRKKQPRRKKPKPQPEKPRKQEQKPKQKKTPKKKPGRRERMCMKIEHDCIFEVKHEGKVTGYACLVGDKVMKPAHVPGVIDNIDLARLSYKKSSKYDLECAQIPVAMKSDASKYTHEKPEGHYNWHYGAVQYTGGRFTVPTGVGKPGDSGRPIFDNKGRVVAIVLGGANEGARTALSVVTWNKDMVTKITPEGTEEWAAPTVTAMCLLANVSFPCFQPSCSPCCYEKGPEPTLRMLEENVNSEGYYELLHAAVYCKNSSRSKRSTANHFNAYKLTRPYVAYCADCGMGHSCHSPAMIENVQADATDGTLKIQFASQIGLTKTDTHDHTKIRYAEGHDIAEAARSTLKVHSSSECAVTGTMGHFILAKCPPGEVISVSFVDSKNEQRTCRIAYHHEQRLIGRERFTVRPHHGIELPCTTYQLTTAETSEEIDMHMPPDIPDRTILSQQSGNVKITVNGRTVKYSCSCGSKPSGTTTTDKTINSCTVDKCQAYVTSHTKWQFNSPFVPRAEQAERKGKVHIPFPLINTTCRVPLAPEALVRSGKREATLSLHPIHPTLLSYRTLGREPVFDEQWITTQTEVTIPVPVEGVEYRWGNHKPQRLWSQLTTEGRAHGWPHEIIEYYYGLHPTTTIVVVVAVSVVVLLSVAASVYMCVVARNKCLTPYALTPGAVVPVTIGVLCCAPKAHAASFAEGMAYLWDNNQSMFWMELTGPLALLILTTCCARSLLSCCKGSFLVAVSVGSAVASAYEHTAVIPNQVGFPYKAHVAREGYSPLTLQMQVVETSLEPTLNLEYITCDYKTKVPSPYVKCCGTAECRTQDKPEYKCAVFTGVYPFMWGGAYCFCDSENTQMSEAYVERADVCKHDYAAAYRAHTASLRAKIKVTYGTVNQTVEAYVNGDHAVTIAGTKFIFGPVSTAWTPFDTKIVVYKGEVYNQDFPPYGAGQPGRFGDIQSRTLDSKDLYANTGLKLARPAAGNIHVPYTQTPSGFKTWQKDRDSPLNAKAPFGCTIQTNPVRAMNCAVGNIPVSMDIADSAFTRLTDAPIISELLCTVSTCTHSSDFGGVAVLSYKVEKAGRCDVHSHSNVAVLQEVSIEAEGRSVIHFSTASAAPSFIVSVCSSRATCTAKCEPPKDHVVTYPANHNGITLPDLSSTAMTWAQHLAGGVGLLIALAVLILVIVTCITLRR

>ARQ30128.1:1-1242 polyprotein [Mayaro virus]MDFLPTQVFYGRRWRPRMPPRPWRPRPPTIQRPDQQARQMQQLIAAVSTLALRQNAAAPQRGRKKQPRRKKPKPQPEKPKKQEQKPKQKKTPKKKPGRRERMCMKIEHDCIFEVKHEGKVTGYACLVGDKVMKPAHVPGVIDNIDLARLSYKKSSKYDLECAQIPVAMKSDASKYTHEKPEGHYNWHYGAVQYTGGRFTVPTGVGKPGDSGRPIFDNKGRVVAIVLGGANEGARTALSVVTWNKDMVTKITPEGTEEWAAPTVTAMCLLANVSFPCFQPSCSPCCYEKGPEPTLRMLEENVNSEGYYELLHAAVYCKNSSRSKRSTANHFNAYKLTRPYVAYCADCGMGHSCHSPAMIENVQADATDGTLKIQFASQIGLTKTDTHDHTKIRYAEGHDIAEAARSTLKVHSSSECAVTGTMGHFILAKCPPGEVISVSFVDSKNEQRTCRIAYHHEQRLIGRERFTVRPHHGIELPCTTYQLTTAETSEEIDMHMPPDIPDRTILSQQSGNVKITVNGRTVKYSCSCGSKPSGTTTTDKTINSCTVDKCQAYVTSHTKWQFNSPFVPRAEQAERKGKVHIPFPLINTTCRVPLAPEALVRSGKREATLSLHPVHPTLLSYRTLGREPVFDEQWITTQTEVTIPVPVEGVEYRWGNHKPQRLWSQLTTEGRAHGWPHEIIEYYYGLHPTTTIVVVVAVSVVVLLSVAASVYMCVVARNKCLTPYALTPGAVVPVTIGVLCCAPKAHAASFAEGMAYLWDNNQSMFWMELTGPLALLILTTCCARSLLSCCKGSFLVAVSVGSAVASAYEHTAVIPNQVGFPYKAHVAREGYSPLTLQMQVVETSLEPTLNLEYITCDYKTKVPSPYVKCCGTAECRTQDKPEYKCAVFTGVYPFMWGGAYCFCDSENTQMSEAYVERADVCKHDYAAAYRAHTASLRAKIKVTYGTVNQTVEAYVNGDHAVTIAGTKFIFGPVSTAWTPFDTKIVVYKGEVYNQDFPPYGAGQPGRFGDIQSRTLDSKDLYANTGLKLARPAAGNIHVPYTQTPSGFKTWQKDRDSPLNAKAPFGCTIQTNPVRAMNCAVGNIPVSMDIADSAFTRLTDAPIISELLCTVSTCTHSSDFGGVAVLSYKVEKAGRCDVHSHSNVAVLQEVSIEAEGRSVIHFSTASAAPSFIVSVCSSRATCTAKCEPPKDHVVTYPANHNGITLPDLSSTAMTWAQHLAGGVGLLIALAVLILVIVTCITLRR

>ASY08178.1:1-1242 structural polyprotein [Mayaro virus]MDFLPTQVFYGRRWRPRMPPRPWRPRPPTIQRPDQQARQMQQLIAAVSTLALRQNAAAPQRGRKKQPRRKKPKPQPEKPKKQEQKPKQKKTPKKKPGRRERMCMKIEHDCIFEVKHEGKVTGYACLVGDKVMKPAHVPGVIDNIDLARLSYKKSSKYDLECAQIPVAMKSDASKYTHEKPEGHYNWHYGAVQYTGGRFTVPTGVGKPGDSGRPIFDNKGRVVAIVLGGANEGARTALSVVTWNKDMVTKITPEGTEEWAAPTVTAMCLLANVSFPCFQPSCSPCCYEKGPEPTLRMLEENVNSEGYYELLHAAVYCKNSSRSKRSTANHFNAYKLTRPYVAYCADCGMGHSCHSPAMIENVQADATDGTLKIQFASQIGLTKTDTHDHTKIRYAEGHDIAEAARSTLKVHSSSECAVTGTMGHFILAKCPPGEVISVSFVDSKNEQRTCRIAYHHEQRLIGRERFTVRPHHGIELPCTTYQLTTAETSEEIDMHMPPDIPDRTILSQQSGNVKITVNGRTVKYSCSCGSKPSGTTTTDKTINSCTVDKCQAYVTSHTKWQFNSPFVPRAEQAERKGKVHIPFPLINTTCRVPLAPEALVRSGKREATLSLHPIHPTLLSYRTLGREPVFDEQWITTQTEVTIPVPVEGVEYRWGNHKPQRLWSQLTTEGRAHGWPHEIIEYYYGLHPTTTIVVVVAVSVVVLLSVAASVYMCVVARNKCLTPYALTPGAVVPVTIGVLCCAPKAHAASFAEGMAYLWDNNQSMFWMELTGPLALLILTTCCARSLLSCCKGSFLVAVSIGSAVASAYEHTAVIPNQVGFPYKAHVAREGYSPLTLQMQVVETSLEPTLNLEYITCDYKTKVPSPYVKCCGTAECRTQDKPEYKCAVFTGVYPFMWGGAYCFCDSENTQMSEAYVERADVCKHDYAAAYRAHTASLRAKIKVTYGTVNQTVEAYVNGDHAVTIAGTKFIFGPVSTAWTPFDTKIVVYKGEVYNQDFPPYGAGQPGRFGDIQSRTLDSKDLYANTGLKLARPAAGNIHVPYTQTPSGFKTWQKDRDSPLNAKAPFGCTIQTNPVRAMNCAVGNIPVSMDIADSAFTRLTDAPIISELLCTVSTCTHSSDFGGVAVLSYKVEKAGRCDVHSHSNVAVLQEVSIEAEGRSVIHFSTASAAPSFIVSVCSSRATCTAKCEPPKDHVVTYPANHNGITLPDLSSTAMTWAQHLAGGVGLLIALAVLILVIVTCITLRR

>ASY08157.1:1-1242 structural polyprotein [Mayaro virus]MDFLPTQVFYGRRWRPRMPPRPWRPRPPTIQRPDQQARQMQQLIAAVSTLALRQNAAAPQRGRKKQPRRKKPKPQPEKPKKQEQKPKQKKTPKKKPGRRERMCMKIEHDCIFEVKHEGKVTGYACLVGDKVMKPAHVPGVIDNIDLARLSYKKSSKYDLECAQIPVAMKSDASKYTHEKPEGHYNWHYGAVQYTGGRFTVPTGVGKPGDSGRPIFDNKGRVVAIVLGGANEGARTALSVVTWNKDMVTKITPEGTEEWAAPTVTAMCLLANVSFPCFQPSCSPCCYEKGPEPTLRMLEENVNSEGYYELLHAAVYCKNSSRSKRSTANHFNAYKLTRPYVAYCADCGMGHSCHSPAMIENVQADATDGTLKIQFASQIGLTKTDTHDHTKIRYAEGHDIAEAARSTLKVHSSSECAVTGTMGHFILAKCPPGEVISVSFVDSKNEQRTCRIAYHHEQRLIGRERFTVRPHHGIELPCTTYQLTTAETSEEIDMHMPPDIPDRTILSQQSGNVKITVNGRTVKYSCSCGSKPSGTTTTDKTINSCTVDKCQAYVTSHTKWQFNSPFVPRAEQAERKGKVHIPFPLINTTCRVPLAPEALVRSGKREATLSLHPIHPTLLSYRTLGREPVFDEQWITTQTEVTIPVPVEGVEYRWGNHKPQRLWSQLTTEGRAHGWPHEIIEYYYGLHPTTTIVVVVAVSVVVLLSVAASVYMCVVARNKCLTPYALTPGAVVPVTIGVLCCAPKAHAASFAEGMAYLWDNNQSMFWMELTGPLALLILTTCCARSLLSCCKGSFLVAVSVGSAVASAYEHTAVIPNQVGFPYKAHVAREGYSPLTLQMQVVETSLEPTLNLEYITCDYKTKVPSPYVKCCGTAECRTQDKPEYKCAVFTGVYPFMWGGAYCFCDSENTQMSEAYVERADVCKHDYAAAYRAHTASLRAKIKVTYGTVNQTVEAYVNGDHAVTIAGTKFIFGPVSTAWTPFDTKIVVYKGEVYNQDFPPYGAGQPGRFGDIQSRTLDSEDLYANTGLKLARPAAGNIHVPYTQTPSGFKTWQKDRDSPLNAKAPFGCTIQTNPVRAMNCAVGNIPVSMDIADSAFTRLTDAPIISELLCTVSTCTHSSDFGGVAVLSYKVEKAGRCDVHSHSNVAVLQEVSIEAEGRSVIHFSTASAAPSFIVSVCSSRATCTAKCEPPKDHVVTYPANHNGITLPDLSSTAMTWAQHLAGGVGLLIALAVLILVIVTCITLRR

>ALI88590.1:1-1242 structural polyprotein [Mayaro virus]MDFLPTQVFYGRRWRPRMPPRPWRPRPPTIQRPDQQARQMQQLIAAVSTLALRQNAAAPQRGRKKQPRRKKPKPQPEKPKKQEQKPKQKKTPKKKPGRRERMCMKIEHDCIFEVKHEGKVTGYACLVGDKVMKPAHVPGVIDNIDLARLSYKKSSKYDLECAQIPVAMKSDASKYTHEKPEGHYNWHYGAVQYTGGRFTVPTGVGKPGDSGRPIFDNKGRVVAIVLGGANEGARTALSVVTWNKDMVTKITPEGTEEWAAPTVTAMCLLANVSFPCFQPSCSPCCYEKGPEPTLRMLEENVNSEGYYELLHAAVYCKNSSRSKRSTANHFNAYKLTRPYVAYCADCGMGHSCHSPAMIENVQADATDGTLKIQFASQIGLTKTDTHDHTKIRYAEGHDIAEAARSTLKVHSSSECAVTGTMGHFILAKCPPGEVISVSFVDSKNEQRTCRIAYHHEQRLIGRERFTVRPHHGIELPCTTYQLTTAETSEEIDMHMPPDIPDRTILSQQSGNVKITVNGRTVKYSCSCGSKPSGTTTTDKTINSCTVDKCQAYVTSHTKWQFNSPFVPRAEQAERKGKVHIPFPLINTTCRVPLAPEALVRSGKREATLSLHPIHPTLLSYRTLGREPVFDEQWITTQTEVTIPVPVEGVEYRWGNHKPQRLWSQLTTEGRAHGWPHEIIEYYYGLHPTTTIVVVVAVSVVVLLSVAASVYMCVVARNKCLTPYALTPGAVVPVTIGVLCCAPKAHAASFAEGMAYLWDNNQSMFWMELTGPLALLILTTCCARSLLSCCKGSFLVAVSVGSAVASAYEHTAVIPNQVGFPYKAHVAREGYSPLTLQMQVVETSLEPTLNLEYITCDYKTKVPSPYVKCCGTAECRTQDKPEYKCAVFTGVYPFMWGGAYCFCDSENTQMSEAYVERADVCKHDYAAAYRAHTASLRAKIKVTYGTVNQTVEAYVNGDHAVTLAGTKFIFGPVSTAWTPFDTKIVVYKGEVYNQDFPPYGAGQPGRFGDIQSRTLDSKDLYANTGLKLARPAAGNIHVPYTQTPSGFKTWQKDRDSPLNAKAPFGCTIQTNPVRAMNCAVGNIPVSMDIADSAFTRLTDAPIISELLCTVSTCTHSSDFGGVAVLSYKVEKAGRCDVHSHSNVAVLQEVSIEAEGRSVIHFSTASAAPSFIVSVCSSRATCTAKCEPPKDHVVTYPANHNGITLPDLSSTAMTWAQHLAGGVGLLIALAVLILVIVTCITLRR

>QDL88209.1:1-1242 structural polyprotein [Mayaro virus]MDFLPTQVFYGRRWRPRMPPRPWRPRPPTIQRPDQQARQMQQLIAAVSTLALRQNAAAPQRGRKKQPRRKKPKPQPEKPKKQEQKPKQKKTPKKKPGRRERMCMKIEHDCIFEVKHEGKVTGYACLVGDKVMKPAHVPGVIDNIDLARLSYKKSSKYDLECAQIPVAMKSDASKYTHEKPEGHYNWHYGAVQYTGGRFTVPTGVGKPGDSGRPIFDNKGRVVAIVLGGANEGARTALSVVTWNKDMVTKITPEGTEEWAAPTVTAMCLLANVSFPCFQPSCSPCCYEKGPEPTLRMLEENVNSEGYYELLHAAVYCKNSSRSKRSTANHFNAYKLTRPYVAYCADCGMGHSCHSPAMIENVQADATDGTLKIQFASQIGLTKTDTHDHTKIRYAEGHDIAEAARSTLKVHSSSECAVTGTMGHFILAKCPPGEVISVSFVDSKNEQRTCRIAYHHEQRLIGREKFTVRPHHGIELPCTTYQLTTAETSEEIDMHMPPDIPDRTILSQQSGNVKITVNGRTVKYSCSCGSKPSGTTTTDKTINSCTVDKCQAYVTSHTKWQFNSPFVPRAEQAERKGKVHIPFPLINTTCRVPLAPEALVRSGKREATLSLHPIHPTLLSYRTLGREPVFDEQWITTQTEVTIPVPVEGVEYRWGNHKPQRLWSQLTTEGRAHGWPHEIIEYYYGLHPTTTIVVVVAVSVVVLLSVAASVYMCVVARNKCLTPYALTPGAVVPVTIGVLCCAPKAHAASFAEGMAYLWDNNQSMFWMELTGPLALLILTTCCARSLLSCCKGSFLVAVSVGSAVASAYEHTAVIPNQVGFPYKAHVAREGYSPLTLQMQVVETSLEPTLNLEYITCDYKTKVPSPYVKCCGTAECRTQDKPEYKCAVFTGVYPFMWGGAYCFCDSENTQMSEAYVERADVCKHDYAAAYRAHTASLRVKIKVTYGTVNQTVEAYVNGDHAVTIAGTKFIFGPVSTAWTPFDTKIVVYKGEVYNQDFPPYGAGQPGRFGDIQSRTLDSKDLYANTGLKLARPAAGNIHVPYTQTPSGFKSWQKDRDSPLNAKAPFGCTIQTNPVRAMNCAVGNIPVSMDIADSAFTRLTDAPIISELLCTVSTCTHSSDFGGVAVLSYKVEKAGRCDVHSHSNVAVLQEVSIEAEGRSVIHFSTASAAPSFIVSVCSSRATCTAKCEPPKDHVVTYPANHNGITLPDLSSTAMTWAQHLAGGVGLLIALAVLILVTVTCITLRR

>ALI88614.1:1-1242 structural polyprotein [Mayaro virus]MDFLPTQVFYGRRWRPRMPPRPWRPRPPTIQRPDQQARQMQQLIAAVSTLALRQNAAAPQRGRKKQPRRKKPKPQPEKPRKQEQKPKQKKTPKKKPGRRERMCMKIEHDCIFEVKHEGKVTGYACLVGDKVMKPAHVPGVIDNIDLARLSYKKSSKYDLECAQIPVAMKSDASKYTHEKPEGHYNWHYGAVQYTGGRFTVPTGVGKPGDSGRPIFDNKGRVVAIVLGGANEGARTALSVVTWNKDMVTKITPEGTEEWAAPTVTAMCLLANVSFPCFQPSCSPCCYEKGPEPTLRMLEENVNSEGYYELLHAAVYCKNSSRSKRSTANHFNAYKLTRPYVAYCADCGMGHSCHSPAMIENVQADATDGTLKIQFASQIGLTKTDTHDHTKIRYAEGHDIAEAARSTLKVHSSSECAVTGTMGHFILAKCPPGEVISVSFVDSKNEQRTCRIAYHHEQRLIGRERFTVRPHHGIELPCTTYQLTTAETSEEIDMHMPPDIPDRTILSQQSGNVKITVNGRTVKYSCSCGSKPSGTTTTDKTINSCTVDKCQAYVTSHTKWQFNSPFVPRAEQAERKGKVHIPFPLINTTCRVPLAPEALVRSGKREATLSLHPIHPTLLSYRTLGREPVFDEQWITTQTEVTIPVPVEGVEYRWGNHKPQRLWSQLTTEGRAHGWPHEIIEYYYGLHPTTTIVVVVAVSVVVLLSVAASVYMCVVARNKCLTPYALTPGAVVPVTIGVLCCAPKAHAASFAEGMAYLWDNNQSMFWMELTGPLALLILTTCCARSLLSCCKGSFLVAVSVGSAVASAYEHTAVIPNQVGFPYKAHVAREGYSPLTLQMQVVETSLEPTLNLEYITCDYKTKVPSPYVKCCGTAECRTQDKPEYKCAVFTGVYPFMWGGAYCFCDSENTQMSEAYVERADVCKHDYAAAYRAHTASLRAKIKVTYGTVNQTVEAYVNGDHAVTIAGTKFIFGPVSTAWTPFDTKIVVYKGEVYNQDFPPYGAGQPGRFGDIQSRTLDSKDLYANTGLKLARPAAGNIHVPYTQTPSGFKTWQKDRDSPLNAKAPFGCTIQTNPVRAMNCAVGNIPVSMDIADSAFTRLTDAPIISELLCTVSTCTHSSDFGGVAVLSYKVEKAGRCDVHSHSNVAVLQEVSIEAEGRSVIHFSTASAAPSFIVSVCSSRATCTAKCEPPKDHAVTYPANHNGITLPDLSSTAMTWAQHLAGGVGLLIALAVLILVIVTCITLRR

>AAY45742.1:1-1242 structural polyprotein [Mayaro virus]MDFLPTQVFYGRRWRPRMPPRPWRPRPPTIQRPDQQARQMQQLIAAVSTLALRQNAAAPQRGRKKQPRRKKPKPQPEKPKKQEQKPKQKKTPKKKPGRRERMCMKIEHDCIFEVKHEGKVTGYACLVGDKVMKPAHVPGVIDNIDLARLSYKKSSKYDLECAQIPVAMKSDASKYTHEKPEGHYNWHYGAVQYTGGRFTVPTGVGKPGDSGRPIFDNKGRVVAIVLGGANEGARTALSVVTWNKDMVTKITPEGTEEWAAPTVTAMCLLANVSFPCFQPSCSPCCYEKGPEPTLRMLEENVNSEGYYELLHAAVYCKNSSRSKRSTANHFNAYKLTRPYVAYCADCGMGHSCHSPAMIENVQADATDGTLKIQFASQIGLTKADTHDHTKIRYAEGHDIAEAARSTLKVHSSSECAVTGTMGHFILAKCPPGEVISVSFVDSKNEQRTCRIAYHHEQRLIGRERFTVRPHHGIELPCTTYQLTTAETSEEIDMHMPPDIPDRTILSQQSGNVKITVNGRTVKYSCSCGSKPSGTTTTDKTINSCTVDKCQAYVTSHTKWQFNSPFVPRAEQAERKGKVHIPFPLINTTCRVPLAPEALVRSGKREATLSLHPIHPTLLSYRTLGREPVFDEQWITTQTEVTIPVPVEGVEYRWGNHKPQRLWSQLTTDGRAHGWPHEIIEYYYGLHPTTTIVVVVAVSVVVLLSVAASVYMCVVARNKCLTPYALTPGAVVPVTIGVLCCAPKAHAASFAEGMAYLWDNNQSMFWMELTGPLALLILTTCCARSLLSCCKGSFLVAVSVGSAVASAYEHTAVIPNQVGFPYKAHVAREGYSPLTLQMQVVETSLEPTLNLEYITCDYKTKVPSPYVKCCGTAECRTQDKPEYKCAVFTGVYPFMWGGAYCFCDSENTQMSEAYVERADVCKHDYAAAYRAHTASLRAKIKVTYGTVNQTVEAYVNGDHAVTIAGTKFIFGPVSTAWTPFDTKIVVYKGEVYNQDFPPYGAGQPGRFGDIQSRTLDSKDLYANTGLKLARPAAGNIHVPYTQTPSGFKTWQKDRDSPLNAKAPFGCTIQTNPVRAMNCAVGNIPVSMDIADSAFTRLTDAPIISELLCTVSTCTHSSDFGGVAVLSYKVEKAGRCDVHSHSNVAVLQEVSIEAEGRSVIHFSTASAAPSFIVSVCSSRATCTAKCEPPKDHVVTYPANHNGITLPDLSSTAMTWAQHLAGGVGLLIALAVLILVIVTCITLRR

>QDL88215.1:1-1242 structural polyprotein [Mayaro virus]MDFLPTQVFYGRRWRPRMPPRPWRPRPPTIQRPDQQARQMQQLIAAVSTLALRQNAAAPQRGRKKQPRRKKPKPQPEKPKKQEQKPKQKKTPKKKPGRRERMCMKIEHDCIFEVKHEGKVTGYACLVGDKVMKPAHVPGVIDNIDLARLSYKKSSKYDLECAQIPVAMKSDASKYTHEKPEGHYNWHYGAVQYTGGRFTVPTGVGKPGDSGRPIFDNKGRVVAIVLGGANEGARTALSVVTWNKDMVTKITPEGTEEWAAPTVTAMCLLANVSFPCFQPSCSPCCYEKGPEPTLRMLEENVNSEGYYELLHAAVYCKNSSRSKRSTANHFNAYKLTRPYVAYCADCGMGHSCHSPAMIENVQADATDGTLKIQFASQIGLTKTDTHDHTKIRYAEGHDIAEAARSTLKVHSSSECAVTGTMGHFILAKCPPGEVISVSFVDSKNEQRTCRIAYHHEQRLIGRERFTVRPHHGIELPCTTYQLTTVETSEEIDMHMPPDIPDRTILSQQSGNVKITVNGRTVKYSCSCGSKPSGTTTTDKTINSCTVDKCQAYVTSHTKWQFNSPFVPRAEQAERKGKVHIPFPLINTTCRVPLAPEALVRSGKREATLSLHPIHPTLLSYRTLGREPVFDEQWITTQTEVTIPVPVEGVEYRWGNHKPQRLWSQLTTEGRAHGWPHEIIEYYYGLHPTTTIVVVVAVSVVVLLSVAASVYMCVVARNKCLTPYALTPGAVVPVTIGVLCCAPKAHAASFAEGMAYLWDNNQSMFWMELTGPLALLILTTCCARSLLSCCKGSFLVAVSVGSAVASAYEHTAVIPNQVGFPYKAHVAREGYSPLTLQMQVVETSLEPTLNLEYITCDYKTKVPSPYVKCCGTAECRTQDKPEYKCAVFTGVYPFMWGGAYCFCDSENTQMSEAYVERADVCKHDYAAAYRAHTASLRAKIKVTYGTVNQTVEAYVNGDHAVTIAGTKFIFGPVSTAWTPFDTKIVVYKGEVYNQDFPPYGAGQPGRFGDIQSRTLDSKDLYANTGLKLARPAAGNIHVPYTQTPSGFKTWQKDRDSPLNAKAPFGCTIQTNPVRAMNCAVGNIPVSMDIADSAFTRLTDAPIISELLCTVSTCTHSSDFGGVAVLSYKVEKAGKCDVHSHSNVAVLQEVSIEAEGRSVIHFSTASAAPSFIVSVCSSRATCTAKCEPPKDHVVTYPANHNGITLPDLSSTAMTWAQHLAGGVGLLIALAVLILVIVTCITLRR

>ALI88635.1:1-1242 structural polyprotein [Mayaro virus]MDFLPTQVFYGRRWRPRMPPRPWRPRPPTIQRPDQQARQMQQLIAAVSTLALRQNAAAPQRGRKKQPRRKKPKPQPEKPKKQEQKPKQKKTPKKKPGRRERMCMKIEHDCIFEVKHEGKVTGYACLVGDKVMKPAHVPGVIDNIDLARLSYKKSSKYDLECAQIPVAMKSDASKYTHEKPEGHYNWHYGAVQYTGGRFTVPTGVGKPGDSGRPIFDNKGRVVAIVLGGANEGTRTALSVVTWNKDMVTKITPEGTEEWAAPTVTAMCLLANVSFPCFQPSCSPCCYEKGPEPTLRMLEENVNSEGYYELLHAAVYCKNSSRSKRSTANHFNAYKLTRPYVAYCADCGMGHSCHSPAMIENVQADATDGTLKIQFASQIGLTKTDTHDHTKIRYAEGHDIAEAARSTLKVHSSSECAVTGTMGHFILAKCPPGEVISVSFVDSKNEQRTCRIAYHHEQRLIGRERFTVRPHHGIELPCTTYQLTTAETSEEIDMHMPPDIPDRTILSQQSGNVKITVNGRTVKYSCSCGSKPSGTTTTDKTINSCTVDKCQAYVTSHTKWQFNSPFVPRAEQAERKGKVHIPFPLINTTCRVPLAPEALVRSGKREATLSLHPIHPTLLSYRTLGREPVFDEQWITTQTEVTIPVPVEGVEYRWGNHKPQRLWSQLTTEGRAHGWPHEIIEYYYGLHPTTTIVVVVAVSVVVLLSVAASVYMWVVARNKCLTPYALTPGAVVPVTIGVLCCAPKAHAASFAEGMAYLWDNNQSMFWMELTGPLALLILTTCCARSLLSCCKGSFLVAVSVGSAVASAYEHTAVIPNQVGFPYKAHVAREGYSPLTLQMQVVETSLEPTLNLEYITCDYKTKVPSPYVKCCGTAECRTQDKPEYKCAVFTGVYPFMWGGAYCFCDSENTQMSEAYVERADVCKHDYAAAYRAHTASLRAKIKVTYGTVNQTVEAYVNGDHAVTIAGTKFIFGPVSTAWTPFDTKIVVYKGEVYNQDFPPYGAGQPGRFGDIQSRTLDSKDLYANTGLKLARPAAGNIHVPYTQTPSGFRTWQKDRDSPLNAKAPFGCTIQTNPVRAMNCAVGNIPVSMDIADSAFTRLTDAPVISELLCTVSTCTHSSDFGGVAVLSYKVEKAGRCDVHSHSNVAVLQEVSIEAEGRSVIHFSTASAAPSFIVSVCSSRATCTAKCEPPKDHVVTYPANHNGITLPDLSSTAMTWAQHLAGGVGLLIALAVLILVIVTCITLRR

>ALI88647.1:1-1242 structural polyprotein [Mayaro virus]MDFLPTQVFYGRRWRPRMPPRPWRPRPPTIQRPDQQARQMQQLIAAVSTLALRQNAAAPQRGRKKQPRRKKPKPQPEKPKKQEQKPKQKKTPKKKPGRRERMCMKIEHDCIFEVKHEGKVTGYACLVGDKVMKPAHVPGVIDNIDLARLSYKKSSKYDLECAQIPVAMKSDASKYTHEKPEGHYNWHYGAVQYTGGRFTVPTGVGKPGDSGRPIFDNKGRVVAIVLGGANEGARTALSVVTWNKDMVTKITPEGTEEWAAPTVTAMCLLANVPFPCFQPSCSPCCYEKGPEPTLRMLEENVNSEGYYELLHAAVYCKNSSRSKRSTANHFNAYKLTRPYVAYCADCGMGHSCHSPAMIENVQADATDGTLKIQFASQIGLTKTDTHDHTKIRYAEGHDIAEAARSTLKVHSSSECAVTGTMGHFILAKCPPGEVISVSFVDSKNEQRTCRIAYHHEQRLIGRERFTVRPHHGIELPCTTYQLTTAETSEEIDMHMPPDIPDRTILSQQSGNVKITVNGRTVKYSCSCGSKPSGTTTTDKTINSCTVDKCQAYVTSHTKWQFNSPFVPRAEQAERKGKVHIPFPLINTTCRVPLAPEALVRSGKREATLSLHPIHPTLLSYRTLGREPVFDEQWITTQTEVTIPVPVEGVEYRWGNHKPQRLWSQLTTEGRAHGWPHEIIEYYYGLHPTTTIVVVVAVSVVVLLSVAASAYMCVVARNKCLTPYALTPGAVVPVTIGVLCCAPKAHAASFAEGMAYLWDNNQSMFWMELTGPLALLILTTCCARSLLSCCKGSFLVAVSVGSAVASAYEHTAVIPNQVGFPYKAHVAREGYSPLTLQMQVVETSLEPTLNLEYITCDYKTKVPSPYVKCCGTAECRTQDKPEYKCAVFTGVYPFMWGGAYCFCDSENTQMSEAYVERADVCKHDYAAAYRAHTASLRAKIKVTYGTVNQTVEAYVNGDHAVTIAGTKFIFGPVSTAWTPFDTKIVVYKGEVYNQDFPPYGAGQPGRFGDIQSRTLDSKDLYANTGLKLARPAAGNIHVPYTQTPSGFKTWQKDRDSPLNAKAPFGCTIQTNPVRAMNCAVGNIPVSMDIADSAFTRLTDAPIISELLCTVSTCTHSSDFGGVAVLSYKVEKAGRCDVHSHSNVAVLQEVSIEAEGRSVIHFSTASAAPSFIVSVCSSRATCTAKCEPPKDHVVTYPANHNGITLPDLSSTAMTWAQHLAGGVGLLIALAVLILVIVTCITLRR

>ALI88641.1:1-1242 structural polyprotein [Mayaro virus]MDFLPTQVFYGRRWRPRMPPRPWRPRPPTIQRPDQQARQMQQLIAAVSTLALRQNAAAPQRGRKKQPRRKKPKPQPEKPKKQEQKPKQKKTPKKKPGRRERMCMKIEHDCIFEVKHEGKVTGYACLVGDKVMKPAHVPGVIDNIDLARLSYKKSSKYDLECAQIPVAMKSDASKYTHEKPEGHYNWHYGAVQYTGGRFTVPTGVGKPGDSGRPIFDNKGRVVAIVLGGANEGARTALSVVTWNKDMVTKITPEGTEEWAAPTVTAMCLLANVSFPCFQPSCSPCCYEKGPEPTLRMLEENVNSEGYYELLHAAVYCKNSSRSKRSTANHFNAYKLTRPYVAYCADCGMGHSCHSPAMIENVQADATDGTLKIQFASQIGLTKTDTHDHTKIRYAEGHDIAEAARSTLKVHSSSECAVTGTMGHFILAKCPPGEVISVSFVDSKNEQRTCRIAYHHEQRLIGRERFTVRPHHGIELPCTTYQLTTAETSEEIDMHMPPDIPDRTILSQQSGNVKITVNGRTVKYSCSCGSKPSGTTTTDKTINSCTVDKCQAYVTSHTKWQFNSPFVPRAEQAERKGKVHIPFPLINTTCRVPLAPEALVRSGKREAMLSLHPIHPTLLSYRTLGREPVFDEQWITTQTEVTIPVPVEGVEYRWGNHKPQRLWSQLTTEGRAHGWPHEIIEYYYGLHPTTTIVVVVAVSVVVLLSVAASVYMCVVARNKCLTPYALTPGAVVPVTIGVLCCAPKAHAASFAEGMAYLWDNNQSMFWMELTGPLALLILTTCCARSLFSCCKGSFLVAVSVGSAVASAYEHTAVIPNQVGFPYKAHVAREGYSPLTLQMQVVETSLEPTLNLEYITCDYKTKVPSPYVKCCGTAECRTQDKPEYKCAVFTGVYPFMWGGAYCFCDSENTQMSEAYVERADVCKHDYAAAYRAHTASLRAKIKVTYGTVNQTVEAYVNGDHAVTIAGTKFIFGPVSTAWTPFDTKIVVYKGEVYNQDFPPYGAGQPGRFGDIQSRTLDSKDLYANTGLKLARPAAGNIHVPYTQTPSGFKTWQKDRDSPLNAKAPFGCTIQTNPVRAMNCAVGNIPVSMDIADSAFTRLTDAPIISELLCTVSTCTHSSDFGGVAVLSYKVEKAGRCDVHSHSNVAVLQEVSIEAEGRSVIHFSTASAAPSFIVSVCSSRATCTAKCEPPKDHVVTYPANHNGITLPDLSSTAMTWAQHLAGGVGLLIALAVLILVIVTCITLRR

>ALI88626.1:1-1242 structural polyprotein [Mayaro virus]MDFLPTQVFYGRRWRPRMPPRPWRPRPPIIQRPDQQARQMQQLIAAVSTLALRQNAAAPQRGRKKQPRRKKPKPQPEKPKKQEQKPKQKKTPKKKPGRRERMCMKIEHDCIFEVKHEGKVTGYACLVGDKVMKPAHVPGVIDNIDLARLSYKKSSKYDLECAQIPVAMKSDASKYTHEKPEGHYNWHYGAVQYTGGRFTVPTGVGKPGDSGRPIFDNKGRVVAIVLGGANEGARTALSVVTWNKDMVTKITPEGTEEWAAPTVTAMCLLANVSFPCFQPSCSPCCYEKGPEPTLRMLEENVNSEGYYELLHAAVYCKNSSRSKRSTANHFNAYKLTRPYVAYCADCGMGHSCHSPAMIENVQADATDGTLKIQFASQIGLTKTDTHDHTKIRYAEGHDIAEAARSTLKVHSSSECAVTGTMGHFILAKCPPGEVISVSFVDSKNEQRTCRIAYHHEQRLIGRERFTVRPHHGIELPCTTYQLTTAETSEEIDMHMPPDIPDRTILSQQSGNVKITVNGRTVKYSCSCGSKPSGTTTTDNTINSCTVDKCQAYVTSHTKWQFNSPFVPRAEQAERKGKVHIPFPLINTTCRVPLAPEALVRSGKREATLSLHPIHPTLLSYRTLGREPVFDEQWITTQTEVTIPVPVEGVEYRWGNHKPQRLWSQLTTEGRAHGWPHEIIEYYYGLHPTTTIVVVVAVSVVVLLSVAASVYMCVVARNKCLTPYALTPGAVVPVTIGVLCCAPKAHAASFAEGMAYLWDNNQSMFWMELTGPLALLILTTCCARSLFSCCKGSFLVAVSVGSAVASAYEHTAVIPNQVGFPYKAHVAREGYSPLTLQMQVVETSLEPTLNLEYITCDYKTKVPSPYVKCCGTAECRTQDKPEYKCAVFTGVYPFMWGGAYCFCDSENTQMSEAYVERADVCKHDYAAAYRAHTASLRAKIKVTYGTVNQTVEAYVNGDHAVTIAGTKFIFGPVSTAWTPFDTKIVVYKGEVYNQDFPPYGAGQPGRFGDIQSRTLDSKDLYANTGLKLARPAAGNIHVPYTQTPSGFKTWQKDRDSPLNAKAPFGCTIQTNPVRAMNCAVGNIPVSMDIADSAFTRLTDAPIISELLCTVSTCTHSSDFGGVAVLSYKVEKAGRCDVHSHSNVAVLQEVSIEAEGRSVIHFSTASAAPSFIVSVCSSRATCTAKCEPPKDHVVTYPANHNGITLPDLSSTAMTWAQHLAGGVGLLIALAVLILVIVTCITLRR

>ALI88611.1:1-1242 structural polyprotein [Mayaro virus]MDFLPTQVFYGRRWRPRMPPRPWRPRPPTIQRPDQQARQMQQLIAAVSTLALRQNAAAPQRGRKKQPRRKKPKPQPEKPKKQEQKPKQKKTPKKKPGRRERMCMKIEHDCIFEVKHEGKVTGYACLVGDKVMKPAHVPGVIDNIDLARLSYKKSSKYDLECAQIPVAMKSDASKYTHEKPEGHYNWHYGAVQYTGGRFTVPTGVGKPGDSGRPIFDNKGRVVAIVLGGANEGARTALSVVTWNKDMVTKITPEGTEEWAAPTVTAMCLLANVSFPCFQPSCTPCCYEKGPEPTLRMLEENVNSEGYYELLHAAVYCKNSSRSKRSTADHFNAYKLTRPYVAYCADCGMGHSCHSPAMIENVQADATDGTLKIQFASQIGLTKTDTHDHTKIRYAEGHDIAEAARSTLKVHSSSECAVTGTMGHFILAKCPPGEVISVSFVDSKNEQRTCRIAYHHEQRLIGRERFTVRPHHGIELPCTTYQLTTAETSEEIDMHMPPDIPDRTILSQQSGNVKITVNGRTVKYSCSCGSKPSGTTTTDKTINSCTVDKCQAYVTSHTKWQFNSPFVPRAEQAERKGKVHIPFPLINTTCRVPLAPEALVRSGKREATLSLHPIHPTLLSYRTLGREPVFDEQWITTQTEVTIPVPVEGVEYRWGNHKPQRLWSQLTTEGRAHGWPHEIIEYYYGLHPTTTIVVVVAVSVVVLLSVAASVYMCVVARNKCLTPYALTPGAVVPVTIGVLCCAPKAHAASFAEGMAYLWDNNQSMFWMELTGPLALLILTTCCARSLLSCCKGSFLVAVSVGSAVASAYEHTAVIPNQVGFPYKAHVAREGYSPLTLQMQVVETSLEPTLNLEYITCDYKTKVPSPYVKCCGTAECRTQDKPEYKCTVFTGVYPFMWGGAYCFCDSENTQMSEAYVERADVCKHDYAAAYRAHTASLRAKIKVTYGTVNQTVEAYVNGDHAVTIAGTKFIFGPVSTAWTPFDTKIVVYKGEVYNQDFPPYGAGQPGRFGDIQSRTLDSKDLYANTGLKLARPAAGNIHVPYTQTPSGFKTWQKDRDSPLNAKAPFGCTIQTNPVRAMNCAVGNIPVSMDIADSAFTRLTDAPIISELLCTVSTCTHSSDFGGVAVLSYKVEKTGRCDVHSHSNVAVLQEVSIEAEGRSVIHFSTASAAPSFIVSVCSSRATCTAKCEPPKDHVVTYPANHNGITLPDLSSTAMTWAQHLAGGVGLLIALAVLILVIVTCITLRR

>ARQ30119.1:1-1242 polyprotein [Mayaro virus]MDFLPTQVFYGRRWRPRMPPRPWRPRPPTIQRPDQQARQMQQLIAAVSTLALRQNAAAPQRGRKKQPRRKKPKPQPEKPKKQEQKPKQKKTPKKKPGRRERMCMKIEHDCIFEVKHEGKVTGYACLVGDKVMKPAHVPGVIDNIDLARLSYKKSSKYDLECAQIPVAMKSDASKYTHEKPEGHYNWHYGAVQYTGGRFTVPTGVGKPGDSGRPIFDNKGRVVAIVLGGANEGARTALSVVTWNKDMVTKITPEGTEEWAAPTVTAMCLLANVSFPCFQPSCSPCCYEKGPEPTLRMLEENVNSEGYYELLHAAVYCKNSSRSKRSTANHFNAYKLTRPYVAYCADCGMGHSCHSPAMIENVQADATDGTLKIQFASQIGLTKTDTHDHTKIRYAEGHDIAEAARSTLKVHSSSECAVTGTMGHFILAKCPPGEVISVSFVDSKNEQRTCRIAYHHEQRLIGRERFTVRPHHGIELPCTTYQLTTAETSEEIDMHMPPDIPDRTILSQQSGNVKITVNGRTVKYSCSCGSKPSGTTTTDKTINSCTVDKCQAYVTSHTKWQFNSPFVPRAEQAERKGKVHIPFPLINTTCRVPLAPEALVRSGKREATLSLHPIHPTLLSYRTLGREPVFDEQWITTQTEVTIPVPVEGVEYRWGNHKPQRLWSQLTTEGRAHGWPHEIIEYYYGLHPTTTIVVVVAVSVVVLLSVAASVYMCVVARNKCLTPYALTPGAVVPVTIGVLCCAPKAHAASFAEGMAYLWDNNQSMFWMELTGPLALLILTTCCARSLLSCCKGSFLVAVSVGSAVASAYEHTAVIPNQVGFPYKAHVAREGYSPLTLQMQVVETSLEPTLNLEYITCDYKTKVPSPYVKCCGTAECRTQDKPEYRCAVFTGVYPFMWGGAYCFCDSENTQMSEAYVERADVCKHDYAAAYRAHTASLRAKIKVTYGTVNQTVEAYVNGDHAVTIAGTKFIFGPVSTAWTPFDTKIVVYKGEVYNQDFPPYGAGQPGRFGDIQSRTLDSKDLYANTGLKLARPAAGNIHVPYTQTPSGFKTWQKDRGSPLNANAPFGCTIQTNPVRAMNCAVGNIPVSMDIADSAFTRLTDAPIISELLCTVSTCTHSSDFGGVAVLSYKVEKAGRCDVHSHSNVAVLQEVSIEAEGRSVIHFSTASAAPSFIVSVCSSRATCTAKCEPPKDHVVTYPANHNGITLPDLSSTAMTWAQHLAGGVGLLIALAVLILVIVTCITLRR

>ARQ30116.1:1-1242 polyprotein [Mayaro virus]MDFLPTQVFYGRRWRPRMPPRPWRPRPPTIQRPDQQARQMQQLIAAVSTLALRQNAAAPQRGRKKQPRRKKPKPQPEKPKKQEQKPKQKKTPKKKPGRRERMCMKIEHDCIFEVKHEGKVTGYACLVGDKVMKPAHVPGVIDNIDLARLSYKKSSKYDLECAQIPVAMKSDASKYTHEKPEGHYNWHYGAVQYTGGRFTVPTGVGKPGDSGRPIFDNKGRVVAIVLGGANEGARTALSVVTWNKDMVTKITPEGTEEWAAPTVTAMCLLANVSFPCFQPSCSPCCYEKGAEPTLRMLEENVNSEGYYELLHAAVYCKNSSRSKRSTANHFNAYKLTRPYVAYCADCGMGHSCHSPAMIENVQADATDGTLKIQFASQIGLTKTDTHDHTKIRYAEGHDIAEAARSTLKVHSSSECAVTGTMGHFILAKCPPGEVISVSFVDSKNEQRTCRIAYHHEQRLIGRERFTVRPHHGIELPCTTYQLTTAETSEEIDMHMPPDIPDRTILSQQSGNVKITVNGRTVKYSCSCGSKPSGTTTTDKTINSCTVDKCQAYVTSHTKWQFNSPFVPRAEQAERKGKVHIPFPLINTTCRVPLAPEALVRSGKREATLSLHPIHPTLLSYRTLGREPVFDEQWITTQTEVTIPVPVEGVEYRWGNHKPQRLWSQLTTEGRAHGWPHEIIEYYYGLHPTATIVVVVAVSVVVLLSVAASVYMCVVARNKCLTPYALTPGAVVPVTIGVLCCAPKAHAASFAEGMAYLWDNNQSMFWMELTGPLALLILTTCCARSLLSCCKGSFLVAVSVGSAVASAYEHTAVIPNQVGFPYKAHVAREGYSPLTLQMQVVETSLEPTLNLEYITCDYKTKVPSPYVKCCGTAECRTQDKPEYKCAVFTGVYPFMWGGAYCFCDSENTQMSEAYVERADVCKHDYAAAYRAHTASLRAKIKVTYGTVNQTVEAYVNGDHAVTIAGTKFIFGPVSTAWTPFDTKIVVYKGEVYNQDFPPYGAGQPGRFGDIQSRTLDSKDLYANTGLKLARPAAGNIHVPYTQTPSGFKTWQKDRDSPLNAKAPFGCTIQSNPVRAMNCAVGNIPVSMDIADSAFTRLTDAPIISELLCTVSTCTHSSDFGGVAVLSYKVEKAGRCDVHSHSNVAVLQEVSIEAEGRSVIHFSTASAAPSFIVSVCSSRATCTAKCEPPKDHVVTYPANHNGITLPDLSSTAMTWAQHLAGGVGLLIALAVLILVIVTCITLRR

>ALI88659.1:1-1242 structural polyprotein [Mayaro virus]MDFLPTQVFYGRRWRPRMPPRPWRPRPPIIQRPDQQARQMQQLIAAVSTLALRQNAAAPQRGRKKQPRRKKPKPQPEKPKKQEQKPKQKKTPKKKPGRRERMCMKIEHDCIFEVKHEGKVTGYACLVGDKVMKPAHVPGVIDNIDLARLSYKKSSKYDLECAQIPVAMKSDASKYTHEKPEGHYNWHYGAVQYTGGRFTVPTGVGKPGDSGRPIFDNKGRVVAIVLGGANEGARTALSVVTWNKDMVTKITPEGTEEWAAPTVTAMCLLANVSFPCFQPSCSPCCYEKGPEPTLRMLEENVNSEGYYELLHAAVYCKNSSRSKRSTANHFNAYKLTRPYVAYCADCGMGHSCHSPAMIENVQADATDGTLKIQFASQIGLTKTDTHDHTKIRYAEGHDIAEAARSTLKVHSSSECAVTGTMGHFILAKCPPGEVISVSFVDSKNEQRTCRIAYHHEQRLIGRERFTVRPHHGIELPCTTYQLTTAETSEEIDMHMPPDIPDRTILSQQSGNVKITVNGRTVKYSCSCGSKPSGTTTTDKTINSCTVDKCQAYVTSHTKWQFNSPFVPRAEQAERKGKVHIPFPLVNTTCRVPLAPEALVRSGKREATLSLHPIHPTLLSYRTLGREPVFDEQWITTQTEVTIPVPVEGVEYRWGNHKPQRLWSQLTTEGRAHGWPHEIIEYYYGLHPTTTIVVVVAVSVVVLLSVAASVYMCVVARNKCLTPYALTPGAVVPVTIGVLCCAPKAHAASFAEGMAYLWDNNQSMFWMELTGPLALLILTTCCARSLFSCCKGSFLVAVSVGSAIASAYEHTAVIPNQVGFPYKAHVAREGYSPLTLQMQVVETSLEPTLNLEYITCDYKTKVPSPYVKCCGTAECRTQDKPEYKCAVFTGVYPFMWGGAYCFCDSENTQMSEAYVERADVCKHDYAAAYRAHTASLRAKIKVTYGTVNQTVEAYVNGDHAVTIAGTKFIFGPVSTAWTPFDTKIVVYKGEVYNQDFPPYGAGQPGRFGDIQSRTLDSKDLYANTGLKLARPAAGNIHVPYTQTPSGFKTWQKDRDSPLNAKAPFGCTIQTNPVRAMNCAVGNIPVSMDIADSAFTRLTDAPIISELLCTVSTCTHSSDFGGVAVLSYKVEKAGRCDVHSHSNVAVLQEVSIEAEGRSVIHFSTASAAPSFIVSVCSSRATCTAKCEPPKDHVVTYPANHNGITLPDLSSTAMTWAQHLAGGVGLLIALAVLILVIVTCITLRR

>ALI88650.1:1-1242 structural polyprotein [Mayaro virus]MDFLPTQVFYGRRWRPRMPPRPWRPRPPTIQRPDQQARQMQQLIAAVSTLALRQNAAAPQRGRKKQPRRKKPKPQPEKPKKQEQKPKQKKTPKKKPGRRERMCMKIEHDCIFEVKHEGKVTGYACLVGDKVMKPAHVPGVIDNIDLARLSYKKSSKYDLECAQIPVAMKSDASKYTHEKPEGHYNWHYGAVQYTGGRFTVPTGVGKPGDSGRPIFDNKGRVVAIVLGGANEGARTALSVVTWNKDMVTKITPEGTEEWAAPTVTAMCLLANVSFPCFQPSCSPCCYEKGPEPTLRMLEENVNSEGYYELLHAAVYCKNSSRSKRSTANHFNAYKLTRPYVAYCADCGMGHSCHSPAMIEKVQADATDGTLKIQFASQIGLTKTDTHDHTKIRYAEGHDIAEAARSTLKVHSSSECAVTGTMGHFILAKCPPGEVISVSFVDSKNEQRTCRIAYHHEQRLIGRERFTVRPHHGIELPCTTYQLTTAETSEEIDMHMPPDIPDRTILSQQSGNVKITVNGRTVKYSCSCGSKPSGTTTTDKTINSCTVDKCQAYVTSHTKWQFNSPFVPRAEQAERKGKVHIPFPLINTTCRVPLAPEALVRSGKREATLSLHPIHPTLLSYRTLGREPVFDEQWITTQTEVTIPVPVEGVEYRWGNHKPQRLWSQLTTEGRAHGWPHEIIEYYYGLHPTTTIVVVVAVSVVVLLSVAASVYMCVVARNKCLTPYALTPGAVVPVTIGVLCCAPKAHAASFAEGMAYLWDNNQSMFWMELTGPLALLILTTCCARSLFSCCKGSFLVAVSVGSAVASAYEHTAVIPNQVGFPYKAHVAREGYSPLTLQMQVVETSLEPTLNLEYITCDYKTKVPSPYVKCCGTAECRTQDKPEYKCAVFTGVYPFMWGGAYCFCDSENTQMSEAYVERADVCKHDYAAAYRAHTASLRAKIKVTYGTVNQTVEAYVNGDHAVTIAGTKFIFGPVSTAWTPFDTKIVVYKGEVYNQDFPPYGAGQPGRFGDIQSRTLDSKDLYANTGLKLARPAAGNIHVPYTQTPSGFKTWQKDRDSPLNARAPFGCTIQTNPVRAMNCAVGNIPVSMDIADSAFTRLTDAPIISELLCTVSTCTHSSDFGGVAVLSYKVEKAGRCDVHSHSNVAVLQEVSIEAEGRSVIHFSTASAAPSFIVSVCSSRATCTAKCEPPKDHVVTYPANHNGITLPDLSSTAMTWAQHLAGGVGLLIALAVLILVIVTCITLRR

>ALI88608.1:1-1242 structural polyprotein [Mayaro virus]MDFLPTQVFYGRRWRPRMPPRPWRPRPPTIQRPDQQARQMQQLIAAVSTLALRQNAAAPQRGRKKQPRRKKPKPQPEKPKKQEQKPKQKKTPKKKPGRRERMCMKIEHDCIFEVKHEGKVTGYACLVGDKVMKPAHVPGVIDNIDLARLSYKKSSKYDLECAQIPVAMKSDASKYTHEKPEGHYNWHYGAVQYTGGRFTVPTGVGKPGDSGRPIFDNKGRVVAIVLGGANEGARTALSVVTWNKDMVTKITPEGTEEWAAPTVTAMCLLANVSFPCFQPSCSPCCYEKGPEPTLRMLEENVNSEGYYELLHAAVYCKNSSRSKRSTANHFNAYKLTRPYVAYCADCGMGHSCHSPAMIENIQADATDGTLKIQFASQIGLTKTDTHDHTKIRYAEGHDIAEAARSTLKVHSSSECAVTGTMGHFILAKCPPGEVISVSFVDSKNEQRTCRIAYHHEQRLIGRERFTVRPHHGIEIPCTTYQLTTAETSEEIDMHMPPDIPDRTILSQQSGNVRITVNGRTVKYSCSCGSKPSGTTTTDKTINSCTVDKCQAYVTSHTKWQFNSPFVPRAEQAERKGKVHIPFPLINTTCRVPLAPEALVRSGKREATLSLHPIHPRLLSYRTLGREPVFDEQWITTQTEVTIPVPVEGVEYRWGNHKPQRLWSQLTTEGRAHGWPHEIIEYYYGLHPTTTIVVVVAVSVVVLLSVAASVYMCVVARNKCLTPYALTPGAVVPVTIGVLCCAPKAHAASFAEGMAYLWDNNQSMFWMELTGPLALLILTTCCARSLLSCCKGSFLVAVSVGSAVASAYEHTAVIPNQVGFPYKAHVAREGYSPLTLQMQVVETSLEPTLNLEYITCDYKTKVPSPYVKCCGTAECRTQDKPEYKCAVFTGVYPFMWGGAYCFCDSENTQMSEAYVERADVCKHDYAAAYRAHTASLRAKIKVTYGTVNQTVEAYVNGDHAVTIAGTKFIFGPVSTAWTPFDTKIVVYKGEVYNQDFPPYGAGQPGRFGDIQSRTLDSKDLYANTGLKLARPAAGNIHVPYTQTPSGFKTWQKDRDSPLNAKAPFGCTIQTNPVRAMNCAVGNIPISMDIADSAFTRLTDAPIISELLCTVSTCTHSSDFGGVAVLSYKVEKAGRCDVHSHSNVAVLQEVSIEAEGRSVIHFSTASAAPSFIVSVCSSRATCTAKCEPPKDHVVTYPANHNGITLPDLSSTAMTWAQHLAGGVGLLIALAVLILVIVTCITLRR

>QDL88212.1:1-1242 structural polyprotein [Mayaro virus]MDFLPTQVFYGRRWRPRMPPRPWRPRPPTIQRPDQQARQMQQLIAAVSTLALRQNAAAPQRGRKKQPRRKKPKPQPEKPKKQEQKPKQKKTPKKKPGRRERMCMKIEHDCIFEVKHEGKVTGYACLVGDKVMKPAHVPGVIDNIDLARLSYKKSSKYDLECAQIPVAMKSDASKYTHEKPEGHYNWHYGAVQYTGGRFTVPTGVGKPGDSGRPIFDNKGRVVAIVLGGANEGARTALSVVTWNKDMVTKITPEGTEEWAAPTVTAMCLLANVSFPCFQPSCSPCCYEKGPEPTLRMLEENVNSEGYYELLHAAVYCKNSSRSKRSTANHFNAYKLTRPYVAYCADCGMGHSCHSPAMIENIQADATDGTLKIQFASQIGLTKTDTHDHTKIRYAEGHDIAEAARSTLKVHSSSECAVTGTMGHFILAKCPPGEVISVSFVDSKNEQRTCRIAYHHEQRLIGRERFTVRPHHGIEIPCTTYQLTTAETSEEIDMHMPPDIPDRTILSQQSGNVRITVNGRTVKYSCSCGSKPSGTTTTDKTINSCTVDKCQAYVTSHTKWQFNSPFVPRAEQAERKGKVHIPFPLINTTXXVPLAPEALVRSGKREATLSLHPIHPTLLSYRTLGREPVFDEQWITTQTEVTIPVPVEGVEYRWGNHKPQRLWSQLTTEGRAHGWPHEIIEYYYGLHPTTTIVVVVAVSVVVLLSVAASVYMCVVARNKCLTPYALTPGAVVPVTIGVLCCAPKAHAASFAEGMAYLWDNNQSMFWMELTGPLALLILTTCCARSLLSCCKGSFLVAVSVGSAVASAYEHTAVIPNQVGFPYKAHVAREGYSPLTLQMQVVETSLEPTLNLEYITCDYKTKVPSPYVKCCGTAECRTQDKPEYKCAVFTGVYPFMWGGAYCFCDSENTQMSEAYVERADVCKHDYAAAYRAHTASLRAKIKVTYGTVNQTVEAYVNGDHAVTIAGTKFIFGPVSTAWTPFDTKIVVYKGEVYNQDFPPYGAGQPGRFGDIQSRTLDSKDLYANTGLKLARPAAGNIHVPYTQTPSGFKTWQKDRDSPLNAKAPFGCTIQTNPVRAMNCAVGNIPISMDIADSAFTRLTDAPIISELLCTVSTCTHSSDFGGVAVLSYKVEKAGRCDVHSHSNVAVLQEVSIEAEGRSVIHFSTASAAPSFIVSVCSSRATCTAKCEPPKDHVVTYPANHNGITLPDLSSTAMTWAQHLAGGVGLLIALAVLILVIVTCITLRR

>QDL88224.1:1-1242 structural polyprotein [Mayaro virus]MDFLPTQVFYGRRWRPRMPPRPWRPRPPTIQRPDQQARQMQQLIAAVSTLALRQNAAAPQRGRKKQPRRKKPKPQPEKPKKQEQKPKQKKTPKKKPGRRERMCMKIEHDCIFEVKHEGKVTGYACLVGDKVMKPAHVPGVIDNIDLARLSYKKSSKYDLECAQIPVAMKSDASKYTHEKPEGHYNWHYGAVQYTGGRFTVPTGVGKPGDSGRPIFDNKGRVVAIVLGGANEGARTALSVVTWNKDMVTKITPEGTEEWAAPTVTAMCLLANVSFPCFQPSCSPCCYEKGPEPTLRMLEENVNSEGYYELLHAAVYCKNSSRSKRSTANHFNAYKLTRPYVAYCADCGMGHSCHSPAMIENVQADATDGTLKIQFASQIGLTKTXTHDHTKIRYAEGHDIAEAARSTLKVHSSSECAVTGTMGHFILAKCPPGEVISVSFVDSKNEQRTCRIAYHHEQRLIGRERFTVRPHHGIELPCTTYQLTTAETSEEIDMHMPPDIPDRTILSQQSGNVKITVNGRTVKYSCSCGFKPSGTTTTDKTINSCTVDKCQAYVTSHTKWQFNSPFVPRAEQAERKGKVHIPFPLINTTXXXPLAPEALVRSGKREATLSLHPIHPTLLSYRTLGREPVFDEQWITTQTEVTIPVPVEGVEYRWGNHKPQRLWSQLTTEGRAHGWPHEIIEYYYGLHPTTTIVVVVAVSVVVLLSVAASVYMCVVARNKCLTPYALTPGAVVPVTIGVLCCAPKAHAASFAEGMAYLWDNNQSMFWMELTGPLALLILTTCCARSLLSCCKGSFLVAVSVGSAVASAYEHTAVIPNQVGFPYKAHVAREGYSPLTLQMQVVETSLEPTLNLEYITCDYKTKVPSPYVKCCGTAECRTQDKPEYKCAVFTGVYPFMWGGAYCFCDSENTQMSEAYVERADVCKHDYAAAYRAHTASLRAKIKVTYGTVNQTVEAYVNGDHAVTIAGTKFIFGPVSTAWTPFDTKIVVYKGEVYNQDFPPYGAGQPGRFGDIQSRTLDSKDLYANTGLKLARPAAGNIHVPYTQTPSGFKTWQKDRDSPLNAKAPFGCTIQTNPVRAMNCAVGNIPVSMDIADSAFTRLTDAPIISELLCTVSTCTHSSDFGGVAVLSYKVEKAGRCDVHSHSNVAVLQEVSIEAEGRSVIHFSTASAAPSFIVSVCSSRATCTAKCEPPKDHVVTYPANHNGITLPDLSSTAMTWAQHLAGGVGLLIALAVLILVIVTCITLRR

>AZM66146.1:1-1242 structural polyprotein [Mayaro virus]MDFLPTQVFYGRRWRPRMPPRPWRPRPPTIQRPDQQARQMQQLIAAVSTLALRQNAAAPQRGRKKQPRRKKPKPQPEKPKKQEQKPKQKKTPKKKPGRRERMCMKIEHDCIFEVKHEGKVTGYACLVGDKVMKPAHVPGVIDNIDLARLSYKKSSKYDLECAQIPVAMKSDASKYTHEKPEGHYNWHYGAVQYTGGRFTVPTGVGKPGDSGRPIFDNKGRVVAIVLGGANEGARTALSVVTWNKDMVTKITPEGTEEWAAPTVTAMCLLANVSFPCFQPSCSPCCYEKGPEPTLRMLEENVNSEGYYELLHAAVYCKNSSRSKRSTANHFNAYKLTRPYVAYCADCGMGHSCHSPAMIENVQADATDGTLKIQFASQIGLTKKDTHDHTKIRYAEGHDIAEAARSTLKVHSSSECAVTGTMGHFILAKCPPGEVISVSFVDSKNEQRTCRIAYRHEQRLIGRERFTVRPHHGIELPCTTYQLTTAETSEEIDMHMPPDVPDRTILSQQSGNVKITVNGRIVKYSCSCGSKPSGTTTTDKTINSCTVDKCQAYVTSHTKWQFNSPFVPRAEQAERKGKVHIPFPLINTTCRVPLAPEALVRSGKREATLSLHPIHPTLLSYRTLGREPVFDEQWITTQTEVTIPVPVEGVEYRWGNHKPQRLWSQLTTEGRAHGWPHEIIEYYYGLHPTTTIVVVVAVSVVVLLSVAASVYMCVVARNKCLTPYALTPGAVVPVTIGVLCCAPKAHAASFAEGMAYLWDNNQSMFWMELTGPLALLILTTCCARSLLSCCKGSFLVAVSVGSAVASAYEHTAVIPNQVGFPYKAHVAREGYSPLTLQMQVIETSLEPTLNLEYITCEYKTKVPSPYVKCCGTAECRTQDKPEYKCAVFTGVYPFMWGGAYCFCDSENTQMSEAYVERADVCKHDYAAAYRAHTASLRAKIKVAYGTVNQTVEAYVNGDHAVTIAGTKFIFGPVSTAWTPFDTKIVVYKGEVYNQDFPPYGAGQPGRFGDIQSRTLDSKDLYANTGLKLARPAAGNIHVPYTQTPSGFKTWQKDRDSPLNAKAPFGCTIQTNPVRAMNCAVGNIPVSMDIADSAFTRLTDAPIISELLCTVSTCTHSSDFGGVAVLSYKVEKAGRCDVHSHSNVAVLQEVFIEAEGRSVIHFSTASAAPSFIVSVCSSRATCTAKCEPPKDHVVTYPANHNGIILPDLSSTAMTWAQHLAGGVGLLIALAVLILVIVTCITLRR

>AAO33335.1:1-1242 structural polyprotein precursor [Mayaro virus]MDFLPTQVFYGRRWRPRMPPRPWRPRPPTIQRPDQQARQMQQLIAAVSTLALRQNAAAPQRGRKKQPRRKKPKPQPEKPKKQEQKPKQKKTPKKKPGRRERMCMKIEHDCIFEVKHEGKVTGYACLVGDKVMKPAHVPGVIDNIDLARLSYKKSSKYDLECAQIPVAMKSDASKYTHEKPEVHYNWHYGAVQYTGGRFTVPTGVGKPGDSGRPIFDNKGRVVAIVLGGANEGARTALSVVTWNKDMVTKITPEGTEEWAAPTVTAMCLLANVSFPCFQPSCSPCCYEKGPEPTLRMLEENVNSEGYYELLHAAVYCKNSSRSKRSTANHFNAYKLTRPYVAYCADCGMGHSCHSPAMIENVQADATDGTLKIQFASQIGLTKTDTHDHTKIRYAEGHDIAEAARSTLKVHSSSECAVTGTMGHFILAKCPPGEVISVSFVDSKNEQRTCRIAYRHEQRLIGRERFTVRPHHGIELPCTTYQLTTAKTSEEIDMHMPPDVPDRTILSQQSGNVKITVNGRTVKYSCSCGSKPSGTTTTDKTINSCTVDKCQAYVTSHTKWQFNSPFVPRAEQAERKGKVHIPFPLINTTCRVPLAPEALVRSGKREATLSLHPIHPTLLSYRTLGREPVFDERWITTQTEVTIPVPVEGVEYRWGNHKPQRLWSQLTTEGRAHGWPHEIIEYYYGLHPTTTIVVVVAVSVVVLLSVAASVYMCVVARNKCLTPYALTPGAVVPVTIGVLCCAPKAHAASFAEGMAYLWDNNQSMFWMELTGPLALLILTTCCARSLLSCCKGSFLVAVSVGSAVASAYEHTAVIPNQVGFPYKAHVAREGYSPLTLQMQVIETSLEPTLNLEYITCEYKTKVPSPYVKCCGTAECRTQDKPEYKCAVFTGVYPFMWGGAYCFCDSENTQMSEAYVERADVCKHDYAAAYRAHTASLRAKIKVAYGTVNQTVEAYVNGDHAVTIAGTKFIFGPVSTAWTPFDTKIVVYKGEVYNQDFPPYGAGQPGRFGDIQSRTLDSKDLYANTGLKLARPAAGNIHVPYTQTPSGFKTWQKDRDSPLNAKAPFGCTIQTNPVRAMNCAVGNIPVSMDIADSAFTRLTDAPIISELLCTVSTCTHSSDFGGVAVLSYKVEKAGRCDVHSHSNVAVLQEVFIEAEGRSVIHFSTASAAPSFIVSVCSSRATCTAKCEPPKDHVVTYPANHNGIILPDLSSTAMTWAQHLAGGVGLLIALAVLILVIVTCITLRR

>QNS29916.1:1-1239 Structural polyprotein [Mayaro virus]MDFLPTQVFYGRRWRPRMPPRPWRPRPPTIQRPDQQARQMQQLIAAVSTLALRQNAAAPQRGRKKQPRRKKPKPQPEKPKKQEQKPKQKKTPKKKPGRRERMCMKIEHDCIFEVKHEGKVTGYACLVGDKVMKPAHVPGVIDNIDLARLSYKKSSKYDLECAQIPVAMKSDASKYTHEKPEGHYNWHYGAVQYTGGRFTVPTGVGKPGDSGRPIFDNKGRVVAIVLGGANEGARTALSVVTWNKDMVTKITPEGTEEWAAPTVTAMCLLANVSFPCFQPSCSPCCYEKGPEPTLRMLEENVNSEGYYELLHAAVYCKNSSRSKRSTANHFNAYKLTRPYVAYCADCGMGHSCHSPAMIENVQADATDGTLKIQFASQIGLTKKDTHDHTKIRYAEGHDIAEAARSTLKVHSSSECAVTGTMGHFILAKCPPGEVISVSFVDSKNEQRTCRIAYRHEQRLIGRERFTVRPHHGIELPCTTYQLTTAETSEEIDMHMPPDVPDRTILSQQSGNVKITVNGRTVKYSCSCGSKPSGTTTTDKTINSCTVDKCQAYVTSHTKWQFNSPFVPRAERAERKGKVHIPFPLINTTCRVPLAPEALVRSGKREATLSLHPIHPTLLSYRTLGREPVFDEQWITTQTEVTIPVPVEGVEYRWGNHKPQRLWSQLTTEGRAHGWPHEIIEYYYGLHPTTTIVVVVAVSVVVLLSVAASVYMCVVARNKCLTPYALTPGAVVPVTIGVLCCAPKAHAASFAEGMAYLWDNNQSMFWMELTGPLALLILTTCCARSLLSCCKGSFLVAVSVGSAVASAYEHTAVIPNQVGFPYKAHVAREGYSPLTLQMQVIETSLEPTLNLEYITCEYKTKVPSPYVKCCGAAECRTQDKPEYKCAVFTGVYPFMWGGAYCFCDSENTQMSEAYVERADVCKHDYAAAYRAHTASLRAKIKVAYGTVNQTVEAYVNGDHAVTIAGTKFIFGPVSTAWTPFDTKIVVYKGEVYNQDFPPYGAGQPGRFGDIQSRTLDSKDLYANTGLKLARPAAGNIHVPYTQTPSGFKTWQKDRDSPLNAKAPFGCTIQTNPVRAMNCAVGNIPVSMDIADSAFTRLTDAPIISELLCTVSTCTHSSDFGGVAVLSYKVEKAGRCDVHSHSNVAVLQEVFIEAEGRSVIHFSTASAAPSFIVSVCSSRATCTAKCEPPKDHVVTYPANHNGIILPDLSSTAMTWAQHLAGGVGLLIALAVLILVIVTCIT

>ALI88638.1:1-1239 structural polyprotein [Mayaro virus]MDFLPTQVFYGRRWRPRMPPRPWRPRPPTIQRPDQQARQMQQLIAAVSTLALRQNAAAPQRGRKKQPRRKKPKPQPEKPKKQEQKPKQKKTPKKKPGRRERMCMKIEHDCIFEVKHEGKVTGYACLVGDKVMKPAHVPGVIDNIDLARLSYKKSSKYDLECAQIPVAMKSDASKYTHEKPEGHYNWHYGAVQYTGGRFTVPTGVGKPGDSGRPIFDNKGRVVAIVLGGANEGARTALSVVTWNKDMVTKITPEGTEEWAAPTVTAMCLLANVSFPCFQPSCSPCCYEKGPEPTLRMLEENVNSEGYYELLHAAVYCKNSSRSKRSTANHFNAYKLTRPYVAYCADCGMGHSCHSPAMIENVQADATDGTLKIQFASQIGLTKKDTHDHTKIRYAEGHDIAEAARSTLKVHSSSECAVTGTMGHFILAKCPPGEVISVSFVDSKNEQRTCRIAYRHEQRLIGRERFTVRPHHGIELPCTTYQLTTAETSEEIDMHMPPDVPDRTILSQQSGNVKITVNGRTVKYSCSCGSKPSGITTTDKTINSCTVDKCQAYVTSHTKWQFNSPFVPRAERAERKGKVHIPFPLINTTCRVPLAPEALVRSGKREATLSLHPIHPTLLSYRTLGREPVFDEQWITTQTEVTIPVPVEGVEYRWGNHKPQRLWSQLTTEGRAHGWPHEIIEYYYGLHPTTTIVVVVAVSVVVLLSVAASVYMCVVARNKCLTPYALTPGAVVPVTIGVLCCAPKAHAASFAEGMAYLWDNNQSMFWMELTGPLALLILTTCCARSLLSCCKGSFLVAVSVGSAVASAYEHTAVIPNQVGFPYKAHVAREGYSPLTLQMQVIETSLEPTLNLEYITCEYKTKVPSPYVKCCGAAECRTQDKPEYKCAVFTGVYPFMWGGAYCFCDSENTQMSEAYVERADVCKHDYAAAYRAHTASLRAKIKVAYGTVNQTVEAYVNGDHAVTIAGTKFIFGPVSTAWTPFDTKIVVYKGEVYNQDFPPYGAGQPGRFGDIQSRTLDSKDLYANTGLKLARPAAGNIHVPYTQTPSGFKTWQKDRDSPLNAKAPFGCTIQTNPVRAMNCAVGNIPVSMDIADSAFTRLTDAPIISELLCTVSTCTHSSDFGGVAVLSYKVEKAGRCDVHSHSNVAVLQEVFIEAEGRSVIHFSTASAAPSFIVSVCSSRATCTAKCEPPKDHVVTYPANHNGIILPDLSSTAMTWAQHLAGGVGLLIALAVLILVIVTCIT

>QDL88206.1:1-1239 structural polyprotein [Mayaro virus]MDFLPTQVFYGRRWRPRMPPRPWRPRPPTIQRPDQQARQMQQLIAAVSTLALRQNAAAPQRGRKKQPRRKKPKPQPEKPKKQEQKPKQKKTPKKKPGRRERMCMKIEHDCIFEVKHEGKVTGYACLVGDKVMKPAHVPGVIDNIDLARLSYKKSSKYDLECAQIPVAMKSDASKYTHEKPEGHYNWHYGAVQYTGGRFTVPTGVGKPGDSGRPIFDNKGRVVAIVLGGANEGARTALSVVTWNKDMVTKITPEGTEEWAAPTVTAMCLLANVSFPCFQPSCSPCCYEKGPEPTLRMLEENVNSEGYYELLHAAVYCKNSSRSKRSTANHFNAYKLTRPYVAYCADCGMGHSCHSPAMIENVQADATDGTLKIQFASQIGLTKKDTHDHTKIRYAEGHDIAEAARSTLKVHSSSECAVTGTMGHFILAKCPPGEVISVSFVDSKNEQRTCRIAYRHEQRLIGRERFTVRPHHGIELPCTTYQLTTAETSEEIDMHMPPDVPDRTILSQQSGNVKITVNGRTVKYSCSCGSKPSGITTTDKTINSCTVDKCQAYVTSHTKWQFNSPFVPRAERAERKGKVHIPFPLINTTCRVPLAPEALVRSGKREATLSLHPIHPTLLSYRTLGREPVFDEQWITTQTEVTIPVPVEGVEYRWGNHKPQRLWSQLTTEGRAHGWPHEIIEYYYGLHPTTTIVVVVAVSVVVLLSVAASVYMCVVARNKCLTPYALTPGAVVPVTIGVLCCAPKAHAASFAEGMAYLWDNNQSMFWMELTGPLALLILTTCCARSLLSCCKGSFLVAVSVGSAVASAYEHTAVIPNQVGFPYKAHVAREGYSPLTLQMQVIETSLEPTLNLEYITCEYKTKVPSPYVKCCGAAECRTQDKPEYKCAVFTGVYPFMWGGAYCFCDSENTQMSEAYVERADVCKHDYAAAYRAHTASLRAKIKVAYGTVNQTVEAYVNGDHAVTIAGTKFIFGPVSTAWTPFDTKIVVYKGEVYNQDFPPYGAGQPGRFGDIQSRTLDSKDLYANTGLKLARPAAGNIHVPYTQTPSGFKTWQKDRDSPXNAKAPFGCTIQTNPVRAMNCAVGNIPVSMDIADSAFTRLTDAPIISELLCTVSTCTHSSDFGGVAVLSYKVEKAGRCDVHSHSNVAVLQEVFIEAEGRSVIHFSTASAAPSFIVSVCSSRATCTAKCEPPKDHVVTYPANHNGIILPDLSSTAMTWAQHLAGGVGLLIALAVLILVIVTCIT

>YP_006491249.1:1-794 truncated polyprotein [Mayaro virus]MDFLPTQVFYGRRWRPRMPPRPWRPRMPTMQRPDQQARQMQQLIAAVSTLALRQNAAAPQRGKKKQPRRKKPKPQPEKPKKQEQKPKQKKAPKRKPGRRERMCMKIEHDCIFEVKHEGKVTGYACLVGDKVMKPAHVPGVIDNADLARLSYKKSSKYDLECAQIPVAMKSDASKYTHEKPEGHYNWHYGAVQYTGGRFTVPTGVGKPGDSGRPIFDNKGPVVAIVLGGANEGTRTALSVVTWNKDMVTKITPEGTVEWAASTVTAMCLLTNISFPCFQPSCAPCCYEKGPEPTLRMLEENVNSEGYYDLLHAAVYCRNSSRSKRSTANHFNAYKLTRPYVAYCADCGMGHSCHSPAMIENIQADATDGTLKIQFASQIGLTKTDTHDHTKIRYAEGHDIAEAARSTLKVHSSSECTVTGTMGHFILAKCPPGERISVSFVDSKNEHRTCRIAYHHEQRLIGRERFTVRPHHGIELPCTTYQLTTAETSEEIDMHMPPDIPDRTILSQQSGNVKITVNGRTVRYSSSCGSQAVGTTTTDKTINSCTVDKCQAYVTSHTKWQFNSPFVPRRMQAERKGKVHIPFPLINTTCRVPLAPEALVRSGKREATLSLHPIHPTLLSYRTFGAERVFDEQWITAQTEVTIPVPVEGVEYQWGNHKPQRFVVALTTEGKAHGWPHEIIEYYYGLHPTTTIVVVIRVSVVVLLSFAASVYMCVVARTKCLTPYALTPGAVVPVTIGVLCCAPKAHAASFAEGMAYLWDNNQSMFWMELTGPLALLILATCCARSLLSCCKGSFL

>AJA30088.1:1-575 structural polyprotein, partial [Mayaro virus]EGRAHGWPHEIIEYYYGLHPTTTIVVVVAVSVVVLLSVAASVYMCVVARNKCLTPYALTPGAVVPVTIGVLCCAPKAHAASFAEGMAYLWDNNQSMFWMELTGPLALLILTTCCARSLLSCCKGSFLVAVSVGSAVASAYEHTAVIPNQVGFPYKAHVAREGYSPLTLQMQVVETSLEPTLNLEYITCDYKTKVPSPYVKCCGTAECRTQDKPEYKCAVFTGVYPFMWGGAYCFCDSENTQMSEAYVERADVCKHDYAAAYRAHTASLRAKIKVTYGTVNQTVEAYVNGDHAVTIAGTKFIFGPVSTAWTPFDTKIVVYKGEVYNQDFPPYGAGQPGRFGDIQSRTLDSKDLYANTGLKLARPAAGNIHVPYTQTPSGFKTWQKDRDSPLNAKAPFGCTIQTNPVRAMNCAVGNIPVSMDIADSAFTRLTDAPIISELLCTVSTCTHSSDFGGVAVLSYKVEKAGRCDVHSHSNVAVLQEVSIEAEGRSVIHFSTASAAPSFIVSVCSSRATCTAKCEPPKDHVVTYPANHNGITLPDLSSTAMTWAQHLAGGVGLLIALAVLILVIVTCITLRR

>AJA30080.1:1-575 structural polyprotein, partial [Mayaro virus]EGRAHGWPHEIIEYYYGLHPTTTIVVVVAVSVVVLLSVAASVYMCVVARNKCLTPYALTPGAVVPVTIGVLCCAPKAHAASFAEGMAYLWDNNQSMFWMELTGPLALLILTTCCARSLLSCCKGSFLVAVSVGSAVASAYEHTAVIPNQVGFPYKAHVAREGYSPLTLQMQVVETSLEPTLNLEYITCDYKTKVPSPYVKCCGTAECRTQDKPEYKCAVFTGVYPFMWGGAYCFCDSENTQMSEAYVERADVCKHDYAAAYRAHTASLRAKIKVTYGTVNQTVEAYVNGDHAVTLAGTKFIFGPVSTAWTPFDTKIVVYKGEVYNQDFPPYGAGQPGRFGDIQSRTLDSKDLYANTGLKLARPAAGNIHVPYTQTPSGFKTWQKDRDSPLNAKAPFGCTIQTNPVRAMNCAVGNIPVSMDIADSAFTRLTDAPIISELLCTVSTCTHSSDFGGVAVLSYKVEKAGRCDVHSHSNVAVLQEVSIEAEGRSVIHFSTASAAPSFIVSVCSSRATCTAKCEPPKDHVVTYPANHNGITLPDLSSTAMTWAQHLAGGVGLLIALAVLILVIVTCITLRR

>AJA30086.1:1-575 structural polyprotein, partial [Mayaro virus]EGRAHGWPHEIIEYYYGLHPTTTIVVVVAVSVVVLLSVAASVYMCVVARNKCLTPYALTPGAVVPVTIGVLCCAPKAHAASFAEGMAYLWDNNQSMFWMELTGPLALLILTTCCARSLFSCCKGSFLVAVSVGSAVASAYEHTAVIPNQVGFPYKAHVAREGYSPLTLQMQVVETSLEPTLNLEYITCDYKTKVPSPYVKCCGTAECRTQDKPEYKCAVFTGVYPFMWGGAYCFCDSENTQMSEAYVERADVCKHDYAAAYRAHTASLRAKIKVTYGTVNQTVEAYVNGDHAVTIAGTKFIFGPVSTAWTPFDTKIVVYKGEVYNQDFPPYGAGQPGRFGDIQSRTLDSKDLYANTGLKLARPAAGNIHVPYTQTPSGFKTWQKDRDSPLNAKAPFGCTIQTNPVRAMNCAVGNIPVSMDIADSAFTRLTDAPIISELLCTVSTCTHSSDFGGVAVLSYKVEKAGRCDVHSHSNVAVLQEVSIEAEGRSVIHFSTASAAPSFIVSVCSSRATCTAKCEPPKDHVVTYPANHNGITLPDLSSTAMTWAQHLAGGVGLLIALAVLILVIVTCITLRR

>AJA30087.1:1-575 structural polyprotein, partial [Mayaro virus]EGRAHGWPHEIIEYYYGLHPTTTIVVVVAVSVVVLLSVAASVYMCVVARNKCLTPYALTPGAVVPVTIGVLCCAPKAHAASFAEGMAYLWDNNQSMFWMELTGPLALLILTTCCARSLLSCCKGSFLVAVSVGSAVASAYEHTAVIPNQVGFPYKAHVAREGYSPLTLQMQVVETSLEPTLNLEYITCDYKTKVPSPYVKCCGTAECRTQDKPEYKCTVFTGVYPFMWGGAYCFCDSENTQMSEAYVERADVCKHDYAAAYRAHTASLRAKIKVTYGTVNQTVEAYVNGDHAVTIAGTKFIFGPVSTAWTPFDTKIVVYKGEVYNQDFPPYGAGQPGRFGDIQSRTLDSKDLYANTGLKLARPAAGNIHVPYTQTPSGFKTWQKDRDSPLNAKAPFGCTIQTNPVRAMNCAVGNIPVSMDIADSAFTRLTDAPIISELLCTVSTCTHSSDFGGVAVLSYKVEKTGRCDVHSHSNVAVLQEVSIEAEGRSVIHFSTASAAPSFIVSVCSSRATCTAKCEPPKDHVVTYPANHNGITLPDLSSTAMTWAQHLAGGVGLLIALAVLILVIVTCITLRR

>NP_740694.1:1-436 envelope glycoprotein E1 [Mayaro virus]YEHTAIIPNQVGFPYKAHVAREGYSPLTLQMQVIETSLEPTLNLEYITCDYKTKVPSPYVKCCGTAECRTQDKPEYKCAVFTGVYPFMWGGAYCFCDSENTQMSEAYVERADVCKHDHAAAYRAHTASLRAKIKVTYGTVNQTVEAYVNGDHAVTIAGTKFIFGPVSTPWTPFDTKILVYKGELYNQDFPRYGAGQPGRFGDIQSRTLDSRDLYANTGLKLARPAAGNIHVPYTQTPSGFKTWQKDRDSPLNAKAPFGCIIQTNPVRAMNCAVGNIPVSMDIADSAFTRLTDAPVISELTCTVSTCTHSSDFGGIAVLSYKVEKSGRCDIHSHSNVAVLQEVSIETEGRSVIHFSTASASPSFVVSVCSSRATCTAKCEPPKDHVVTYPANHNGVTLPDLSSTAMTWAQHLAGGVGLLIALAVLILVIVTCVTLRR

>NP_740693.1:1-422 envelope glycoprotein E2 [Mayaro virus]STANHFNAYKLTRPYVAYCADCGMGHSCHSPAMIENIQADATDGTLKIQFASQIGLTKTDTHDHTKIRYAEGHDIAEAARSTLKVHSSSECTVTGTMGHFILAKCPPGERISVSFVDSKNEHRTCRIAYHHEQRLIGRERFTVRPHHGIELPCTTYQLTTAETSEEIDMHMPPDIPDRTILSQQSGNVKITVNGRTVRYSSSCGSQAVGTTTTDKTINSCTVDKCQAYVTSHTKWQFNSPFVPRRMQAERKGKVHIPFPLINTTCRVPLAPEALVRSGKREATLSLHPIHPTLLSYRTFGAERVFDEQWITAQTEVTIPVPVEGVEYQWGNHKPQRFVVALTTEGKAHGWPHEIIEYYYGLHPTTTIVVVIRVSVVVLLSFAASVYMCVVARTKCLTPYALTPGAVVPVTIGVLCCAPKAHA

>6W1C_A:1-380 Human mAbs broadly protect against infection of arthritiogenic alphaviruses by recognizing conserved elements of the MXR8 receptor binding domain [Mayaro virus (strain Brazil)]YEHTAIIPNQVGFPYKAHVAREGYSPLTLQMQVIETSLEPTLNLEYITCDYKTKVPSPYVKCCGTAECRTQDKPEYKCAVFTGVYPFMWGGAYCFCDSENTQMSEAYVERADVCKHDHAAAYRAHTASLRAKIKVTYGTVNQTVEAYVNGDHAVTIAGTKFIFGPVSTPWTPFDTKILVYKGELYNQDFPRYGAGQPGRFGDIQSRTLDSRDLYANTGLKLARPAAGNIHVPYTQTPSGFKTWQKDRDSPLNAKAPFGCIIQTNPVRAMNCAVGNIPVSMDIADSAFTRLTDAPVISELTCTVSTCTHSSDFGGIAVLSYKVEKSGRCDIHSHSNVAVLQEVSIETEGRSVIHFSTASASPSFVVSVCSSRATCTAKCEP>6W1C_E:1-340 Human mAbs broadly protect against infection of arthritiogenic alphaviruses by recognizing conserved elements of the MXR8 receptor binding domain [Mayaro virus (strain Brazil)]STANHFNAYKLTRPYVAYCADCGMGHSCHSPAMIENIQADATDGTLKIQFASQIGLTKTDTHDHTKIRYAEGHDIAEAARSTLKVHSSSECTVTGTMGHFILAKCPPGERISVSFVDSKNEHRTCRIAYHHEQRLIGRERFTVRPHHGIELPCTTYQLTTAETSEEIDMHMPPDIPDRTILSQQSGNVKITVNGRTVRYSSSCGSQAVGTTTTDKTINSCTVDKCQAYVTSHTKWQFNSPFVPRRMQAERKGKVHIPFPLINTTCRVPLAPEALVRSGKREATLSLHPIHPTLLSYRTFGAERVFDEQWITAQTEVTIPVPVEGVEYQWGNHKPQRFVVA

>AAL35780.1:1-333 glycoprotein, partial [Mayaro virus]GAYCFCDSENTQMSEAYVERADVCKHDYAAAYRAHTASLRAKIKVAYGTVNQTVEAYVNGDHAVTIAGTKFIFGPVSTARTPFDTKIVVYKGEVYNQDFPPYGAGQPGRFGDIQSRTLDSKDLYANTGLKLARPAAGNIHVPYTQTPSGFKTWQKDRDSPLNAKAPFGCTIQTNPVRAMNCAVGNIPVSMDIADSAFTRLTDAPIISELLCTVSTCTHSSDFGGVAVLSYKVEKAGRCDVHSHSNVAVLQEVFIEAEGRSVIHFSTASAAPSFIVSVCSSRATCTAKCEPPKDHVVTYPANHNGIILPDLSSTAMTWAQHLAGGVGLLIALAV

>NP_740691.1:1-258 capsid protein [Mayaro virus]MDFLPTQVFYGRRWRPRMPPRPWRPRMPTMQRPDQQARQMQQLIAAVSTLALRQNAAAPQRGKKKQPRRKKPKPQPEKPKKQEQKPKQKKAPKRKPGRRERMCMKIEHDCIFEVKHEGKVTGYACLVGDKVMKPAHVPGVIDNADLARLSYKKSSKYDLECAQIPVAMKSDASKYTHEKPEGHYNWHYGAVQYTGGRFTVPTGVGKPGDSGRPIFDNKGPVVAIVLGGANEGTRTALSVVTWNKDMVTKITPEGTVEW

>AIF75100.1:1-215 envelope glycoprotein E1, partial [Mayaro virus]LLSCCKGSFLVAVSVGSAVASAYEHTAVIPNQVGFPYKAHVAREGYSPLTLQMQVVETSLEPTLNLEYITCDYKTKVPSPYVKCCGTAECRTQDKPEYKCAVFTGVYPFMWGGAYCFCDSENTQMSEAYVERADVCKHDYAAAYRAHTASLRAKIKVTYGTVNQTVEAYVNGDHAVTIAGTKFIFGPVSTAWTPFDTKIVVYKGEVYNQDFPPYG

>AAD41271.1:1-123 polyprotein, partial [Mayaro virus]SEGYYDLLHAAVYCRNSSRSKRSTANHFNAYKLTRPYVAYCADCGMGHSCHSPAMIENIQADATDGTLKIQFASQIGLTKTDTHDHTKIRYAEGHDIAEAARSTLKVHSSSECTVTGTMGHFI

>AAD41272.1:1-123 polyprotein, partial [Mayaro virus]SEGYYELLHAAVYCKNSSRSKRSTANHFNAYKLTRPYVAYCADCGMGHSCHSPAMIENVQADATDGTLKIQFASQIGLTKADTHDHTKIRYAEGHDIAEAARSTLKVHSSSECAVTGTMGHFI

>NP_740692.1:1-66 envelope glycoprotein E3 [Mayaro virus]AASTVTAMCLLTNISFPCFQPSCAPCCYEKGPEPTLRMLEENVNSEGYYDLLHAAVYCRNSSRSKR

>AEG77352.1:1-67 structural polyprotein precursor, partial [Mayaro virus]PFMWGGAYCFCDSENTQMSEAYVERADVCKHDYAAAYRAHTASLRAKIKVAYGTVNQTVEAYVNGDH

>NP_740695.1:1-60 6k protein [Mayaro virus]ASFAEGMAYLWDNNQSMFWMELTGPLALLILATCCARSLLSCCKGSFLVAMSIGSAVASA>YP_006491250.1:1-48 transframe fusion protein [Mayaro virus]ASFAEGMAYLWDNNQSMFWMELTGPLALLILATCCARSLLSCCKGSFL
