# Supplementary material for: Implementation of in silico methods to predict common epitopes for vaccine development against Chikungunya and Mayaro viruses
Source: Heliyon. 2021 Mar 8;7(3):e06396. doi: 10.1016/j.heliyon.2021.e06396 (PMC7944042; doi:10.1016/j.heliyon.2021.e06396)
Supplement: Supplementary Table 1 [file mmc2.docx]

**Supplementary Table 1:** Potential CD8+ T cell epitopes predicted from CHIKV polyprotein

| **Supertype** | **Epitope** | **Score** |
| --- | --- | --- |
| A1 | FTHEKPEGY | 2.2459 |
|  | STKDNFNVY | 2.1502 |
|  | ATYQEAAVY | 1.6065 |
|  | TVNSQTVRY | 1.5822 |
|  | FTDSRKISH | 1.4179 |
|  | VTWGNNEPY | 1.2063 |
|  | IILYYYELY | 1.037 |
|  | SHDWTKLRY | 1.0321 |
|  | NADLAKLAF | 1.0117 |
|  | LAFKRSSKY | 0.9088 |
|  | KNQVIMLLY | 0.8541 |
|  | KSDASKFTH | 0.833 |
|  | RSMGEEPNY | 0.8313 |
|  | TQTFYNRRY | 0.8222 |
|  | TTDKVINNC | 0.8219 |
|  | TIDNADLAK | 0.7657 |
|  | HPHEIILYY | 0.7537 |
| A2 | YLWNEQQPL | 1.4477 |
|  | FLLSLICCI | 1.3985 |
|  | LLYPDHPTL | 1.3117 |
|  | TMTVVVVSV | 1.1081 |
|  | QLAQLISAV | 1.0762 |
|  | ALIPLAALI | 1.0231 |
|  | ATVPFLLSL | 1.0083 |
|  | QLISAVNKL | 0.9544 |
|  | WLQALIPLA | 0.9372 |
|  | SLAIPVMCL | 0.9213 |
|  | LLSMVGMAV | 0.8911 |
|  | FILLSMVGM | 0.8817 |
|  | KVTGYACLV | 0.869 |
|  | ALIVLCNCL | 0.8527 |
|  | KVDQCHAAV | 0.8238 |
|  | VLTVPTEGL | 0.8189 |
|  | ELYPTMTVV | 0.7916 |
|  | VMRPGYYQL | 0.7753 |
|  | GTLKIQVSL | 0.7745 |
|  | IVLCNCLRL | 0.7545 |
| A3 | KLAFKRSSK | 1.7353 |
|  | KVMKPAHVK | 1.5347 |
|  | TMRAVPQQK | 1.412 |
|  | TVNSQTVRY | 1.3554 |
|  | ALSVVTWNK | 1.2483 |
|  | NVYKATRPY | 1.0171 |
|  | TMGHFILAR | 1.0104 |
|  | LICCIRTAK | 1.0005 |
|  | ATYQEAAVY | 0.9761 |
|  | LAFKRSSKY | 0.9759 |
|  | KARNPTVTY | 0.9495 |
|  | TVGFTDSRK | 0.8994 |
|  | FTIPTGAGK | 0.8993 |
|  | AQLISAVNK | 0.8869 |
|  | HAAVTNHKK | 0.8395 |
|  | ITVNSQTVR | 0.812 |
|  | HPHEIILYY | 0.7833 |
|  | RSMGEEPNY | 0.7767 |
| A24 | LYPDHPTLL | 1.7496 |
|  | YYQLLQASL | 1.5416 |
|  | YYYELYPTM | 1.5201 |
|  | KWQYNSPLV | 1.4042 |
|  | QYSGGRFTI | 1.3444 |
|  | LWNEQQPLF | 1.333 |
|  | LYPTMTVVV | 1.3186 |
|  | TYQEAAVYL | 1.2683 |
|  | YYNWHHGAV | 1.2078 |
|  | VYKATRPYL | 1.1919 |
|  | RYQPRPWTP | 1.1443 |
|  | KYDLECAQI | 0.9517 |
|  | TYGKNQVIM | 0.9325 |
|  | TVPFLLSLI | 0.8981 |
|  | RYMDNHIPA | 0.8642 |
|  | ISHSCTHPF | 0.8403 |
|  | FWLQALIPL | 0.8144 |
|  | VVVVSVASF | 0.8107 |
|  | VYLWNEQQP | 0.7636 |
| A26 | STKDNFNVY | 2.3559 |
|  | EFIPTQTFY | 2.157 |
|  | TITGTMGHF | 1.5139 |
|  | VTWGNNEPY | 1.5023 |
|  | FTHEKPEGY | 1.4754 |
|  | NVYKATRPY | 1.4658 |
|  | EIILYYYEL | 1.2953 |
|  | ATYQEAAVY | 1.2606 |
|  | FILLSMVGM | 1.1827 |
|  | TVNSQTVRY | 1.0946 |
|  | FTIPTGAGK | 0.9111 |
|  | ATVPFLLSL | 0.8315 |
|  | TVPFLLSLI | 0.829 |
|  | CTITGTMGH | 0.8027 |
|  | ELYPTMTVV | 0.7948 |
|  | LTPGATVPF | 0.7839 |
|  | SMVGMAVGM | 0.769 |
| B7 | RPWTPRPTI | 1.65 |
|  | KPGDSGRPI | 1.6136 |
|  | KPAHVKGTI | 1.6117 |
|  | RPQRQAGQL | 1.4675 |
|  | APCTITGTM | 1.4605 |
|  | TPRPTIQVI | 1.3254 |
|  | YPTMTVVVV | 1.3197 |
|  | RPRPQRQAG | 1.3002 |
|  | IPLAALIVL | 1.3001 |
|  | RPRPRPQRQ | 1.2637 |
|  | RPYLAHCPD | 1.18 |
|  | SPHRQRRST | 1.1588 |
|  | VPKARNPTV | 1.1264 |
|  | EPEETLRML | 1.0726 |
|  | FPLANVTCM | 1.0694 |
|  | QPLFWLQAL | 1.0638 |
|  | IPADAGRAG | 0.9914 |
|  | KPRKNRKNK | 0.9882 |
|  | IPVHMKSDA | 0.941 |
|  | PPVIGREKF | 0.9386 |
|  | AVQYSGGRF | 0.9254 |
|  | KARNPTVTY | 0.8805 |
|  | CPKGETLTV | 0.8705 |
|  | VASFILLSM | 0.8666 |
|  | EPYKYWPQL | 0.8554 |
|  | HPFHHDPPV | 0.8307 |
|  | SPLVPRNAE | 0.8189 |
|  | KPGRRERMC | 0.8161 |
|  | APQNNTNQK | 0.8114 |
|  | KGRVVAIVL | 0.7895 |
|  | VVVVSVASF | 0.7763 |
|  | GARTALSVV | 0.7649 |
| B8 | YYQLLQASL | 1.4425 |
|  | VTHKKEVVL | 1.3697 |
|  | VPKARNPTV | 1.2971 |
|  | YYNWHHGAV | 1.2429 |
|  | KGRVVAIVL | 1.2139 |
|  | QPLFWLQAL | 1.1728 |
|  | VMRPGYYQL | 1.0712 |
|  | RPQRQAGQL | 1.0555 |
|  | YGKNQVIML | 1.004 |
|  | FILLSMVGM | 0.9886 |
|  | VNKLTMRAV | 0.9458 |
|  | EIILYYYEL | 0.9302 |
|  | RPRPQRQAG | 0.912 |
|  | ELYPTMTVV | 0.8813 |
|  | TPRPTIQVI | 0.8772 |
|  | LERIRNEAT | 0.85 |
|  | YEKEPEETL | 0.8353 |
|  | HGHPHEIIL | 0.8344 |
|  | YPTMTVVVV | 0.8334 |
|  | DNKGRVVAI | 0.8033 |
|  | MCARRRCIT | 0.7931 |
|  | QALIPLAAL | 0.7922 |
|  | PLVPRNAEL | 0.7861 |
|  | YYYELYPTM | 0.78 |
|  | LLSMVGMAV | 0.7678 |
| B27 | RRCITPYEL | 1.5067 |
|  | RQAGQLAQL | 1.4699 |
|  | IRTAKAATY | 1.3962 |
|  | ARRRCITPY | 1.3437 |
|  | RRERMCMKI | 1.2916 |
|  | KKWQYNSPL | 1.2507 |
|  | GRFTIPTGA | 1.2 |
|  | RRYQPRPWT | 1.178 |
|  | RKGKIHIPF | 1.1681 |
|  | YQEEWVTHK | 1.0706 |
|  | GRAGLFVRT | 1.0374 |
|  | MRPGYYQLL | 1.0195 |
|  | RRRCITPYE | 0.9935 |
|  | GRRERMCMK | 0.9834 |
|  | RKNKKQKQK | 0.951 |
|  | QRRSTKDNF | 0.9323 |
|  | QRQAGQLAQ | 0.8035 |
|  | SRNEHRCPH | 0.7621 |
| B39 | ANEGARTAL | 1.4326 |
|  | YYYELYPTM | 1.4206 |
|  | YEKEPEETL | 1.2318 |
|  | HSCHSPVAL | 1.2242 |
|  | LLYPDHPTL | 1.0858 |
|  | YYQLLQASL | 1.049 |
|  | TGTMGHFIL | 0.9569 |
|  | RRCITPYEL | 0.9529 |
|  | TYQEAAVYL | 0.9339 |
|  | YQEAAVYLW | 0.8682 |
|  | MRPGYYQLL | 0.8584 |
|  | SLAIPVMCL | 0.8327 |
|  | VVSVASFIL | 0.8021 |
|  | YGKNQVIML | 0.7991 |
|  | RQAGQLAQL | 0.7879 |
|  | SHDWTKLRY | 0.7606 |
|  | EPYKYWPQL | 0.7546 |
|  | AHGHPHEII | 0.753 |
| B44 | MEFIPTQTF | 1.5984 |
|  | NEQQPLFWL | 1.5265 |
|  | YELYPTMTV | 1.3666 |
|  | YEKEPEETL | 1.3611 |
|  | PEGAEEWSL | 1.3502 |
|  | KELPCSTYV | 1.1625 |
|  | IENDCIFEV | 1.152 |
|  | FEVKHEGKV | 1.0928 |
|  | EEWSLAIPV | 1.0909 |
|  | YELTPGATV | 0.9963 |
|  | KEPEETLRM | 0.9779 |
|  | AEEWSLAIP | 0.8702 |
|  | RQAGQLAQL | 0.8569 |
|  | GKIHIPFPL | 0.8015 |
|  | EEIEVHMPP | 0.7744 |
| B58 | RTALSVVTW | 2.0043 |
|  | RSMGEEPNY | 1.7764 |
|  | ISAVNKLTM | 1.5762 |
|  | ITPEGAEEW | 1.4723 |
|  | ISHSCTHPF | 1.2737 |
|  | AAVTNHKKW | 1.2546 |
|  | IGTDDSHDW | 1.2186 |
|  | LAIPVMCLL | 1.1907 |
|  | VTNHKKWQY | 1.1338 |
|  | ATYQEAAVY | 1.0453 |
|  | VVSVASFIL | 0.979 |
|  | KSDASKFTH | 0.9368 |
|  | VASFILLSM | 0.9318 |
|  | KARNPTVTY | 0.8886 |
|  | LAFKRSSKY | 0.8562 |
|  | VVVVSVASF | 0.8529 |
|  | RSSKYDLEC | 0.8421 |
|  | LLYPDHPTL | 0.827 |
|  | VTWGNNEPY | 0.7522 |
| B62 | KARNPTVTY | 1.3726 |
|  | ATYQEAAVY | 1.3594 |
|  | ISHSCTHPF | 1.3418 |
|  | VTWGNNEPY | 1.3173 |
|  | STKDNFNVY | 1.3159 |
|  | NVYKATRPY | 1.2905 |
|  | VVVVSVASF | 1.2904 |
|  | HMKSDASKF | 1.2332 |
|  | RSMGEEPNY | 1.19 |
|  | LTPGATVPF | 1.1522 |
|  | TQTFYNRRY | 1.1402 |
|  | LAFKRSSKY | 1.1284 |
|  | MEFIPTQTF | 1.0936 |
|  | RQAGQLAQL | 1.0906 |
|  | YLWNEQQPL | 1.0456 |
|  | FTHEKPEGY | 1.0374 |
|  | VMRPGYYQL | 1.0361 |
|  | AVQYSGGRF | 1.0219 |
|  | SMVGMAVGM | 0.9674 |
|  | QLSANGTAH | 0.9427 |
|  | TVNSQTVRY | 0.9053 |
|  | LLYPDHPTL | 0.898 |
|  | YQEAAVYLW | 0.8781 |
|  | ARRRCITPY | 0.7883 |
|  | ALIVLCNCL | 0.7849 |
|  | SLAIPVMCL | 0.7795 |
|  | EFIPTQTFY | 0.7698 |
