# Supplementary material for: Implementation of in silico methods to predict common epitopes for vaccine development against Chikungunya and Mayaro viruses
Source: Heliyon. 2021 Mar 8;7(3):e06396. doi: 10.1016/j.heliyon.2021.e06396 (PMC7944042; doi:10.1016/j.heliyon.2021.e06396)
Supplement: Supplementary Table 2 [file mmc3.docx]

**Supplementary Table 2:** Potential CD4+ T cell epitopes predicted from CHIKV Frameshifted Structural Polyprotein.

| **Peptide sequence** | **Start** | **End** | **Length** | **MHC-II Allele** | **IC50** |
| --- | --- | --- | --- | --- | --- |
|  |  |  |  |  |  |
| WTPRPTIQVIRPRPR | 19 | 33 | 15 | HLA-DRB5*01:01 | 12 |
| RPTIQVIRPRPRPQR | 22 | 36 | 15 | HLA-DRB5*01:01 | 12 |
| TPRPTIQVIRPRPRP | 20 | 34 | 15 | HLA-DRB5*01:01 | 13 |
| PRPTIQVIRPRPRPQ | 21 | 35 | 15 | HLA-DRB5*01:01 | 13 |
| PTIQVIRPRPRPQRQ | 23 | 37 | 15 | HLA-DRB5*01:01 | 13 |
| TVVVVSVASFILLSM | 692 | 706 | 15 | HLA-DRB1*07:01 | 13 |
| TMTVVVVSVASFILL | 690 | 704 | 15 | HLA-DRB1*07:01 | 14 |
| MTVVVVSVASFILLS | 691 | 705 | 15 | HLA-DRB1*07:01 | 14 |
| PTMTVVVVSVASFIL | 689 | 703 | 15 | HLA-DRB1*07:01 | 15 |
| VVVVSVASFILLSMV | 693 | 707 | 15 | HLA-DRB1*07:01 | 17 |
| YPTMTVVVVSVASFI | 688 | 702 | 15 | HLA-DRB1*07:01 | 32 |
| FWLQALIPLAALIVL | 766 | 780 | 15 | HLA-DRB1*07:01 | 34 |
| WLQALIPLAALIVLC | 767 | 781 | 15 | HLA-DRB1*07:01 | 34 |
| TIQVIRPRPRPQRQA | 24 | 38 | 15 | HLA-DRB5*01:01 | 35 |
| LQALIPLAALIVLCN | 768 | 782 | 15 | HLA-DRB1*07:01 | 35 |
| IQVIRPRPRPQRQAG | 25 | 39 | 15 | HLA-DRB5*01:01 | 36 |
| LFWLQALIPLAALIV | 765 | 779 | 15 | HLA-DRB1*07:01 | 37 |
| TDGTLKIQVSLQIGI | 367 | 381 | 15 | HLA-DRB1*07:01 | 38 |
| QALIPLAALIVLCNC | 769 | 783 | 15 | HLA-DRB1*07:01 | 38 |
| DGTLKIQVSLQIGIG | 368 | 382 | 15 | HLA-DRB1*07:01 | 40 |
| GTLKIQVSLQIGIGT | 369 | 383 | 15 | HLA-DRB1*07:01 | 40 |
| EATDGTLKIQVSLQI | 365 | 379 | 15 | HLA-DRB1*07:01 | 41 |
| VVVSVASFILLSMVG | 694 | 708 | 15 | HLA-DRB1*07:01 | 42 |
| ATDGTLKIQVSLQIG | 366 | 380 | 15 | HLA-DRB1*07:01 | 43 |
| ITPYELTPGATVPFL | 722 | 736 | 15 | HLA-DRB1*07:01 | 47 |
| TPYELTPGATVPFLL | 723 | 737 | 15 | HLA-DRB1*07:01 | 48 |
| CITPYELTPGATVPF | 721 | 735 | 15 | HLA-DRB1*07:01 | 51 |
| VVSVASFILLSMVGM | 695 | 709 | 15 | HLA-DRB1*07:01 | 52 |
| RRCITPYELTPGATV | 719 | 733 | 15 | HLA-DRB1*07:01 | 52 |
| RCITPYELTPGATVP | 720 | 734 | 15 | HLA-DRB1*07:01 | 53 |
| GTLKIQVSLQIGIGT | 369 | 383 | 15 | HLA-DRB4*01:01 | 57 |
| TDGTLKIQVSLQIGI | 367 | 381 | 15 | HLA-DRB4*01:01 | 58 |
| DGTLKIQVSLQIGIG | 368 | 382 | 15 | HLA-DRB4*01:01 | 58 |
| EATDGTLKIQVSLQI | 365 | 379 | 15 | HLA-DRB4*01:01 | 60 |
| ATDGTLKIQVSLQIG | 366 | 380 | 15 | HLA-DRB4*01:01 | 60 |
| DLAKLAFKRSSKYDL | 148 | 162 | 15 | HLA-DRB1*07:01 | 60 |
| LAKLAFKRSSKYDLE | 149 | 163 | 15 | HLA-DRB1*07:01 | 61 |
| AKLAFKRSSKYDLEC | 150 | 164 | 15 | HLA-DRB1*07:01 | 61 |
| TVVVVSVASFILLSM | 692 | 706 | 15 | HLA-DRB1*15:01 | 64 |
| KLAFKRSSKYDLECA | 151 | 165 | 15 | HLA-DRB1*07:01 | 65 |
| VVVVSVASFILLSMV | 693 | 707 | 15 | HLA-DRB1*15:01 | 67 |
| LAFKRSSKYDLECAQ | 152 | 166 | 15 | HLA-DRB1*07:01 | 67 |
| TMTVVVVSVASFILL | 690 | 704 | 15 | HLA-DRB1*15:01 | 68 |
| MTVVVVSVASFILLS | 691 | 705 | 15 | HLA-DRB1*15:01 | 68 |
| ETLTVGFTDSRKISH | 434 | 448 | 15 | HLA-DRB1*07:01 | 70 |
| TLTVGFTDSRKISHS | 435 | 449 | 15 | HLA-DRB1*07:01 | 70 |
| FWLQALIPLAALIVL | 766 | 780 | 15 | HLA-DRB1*15:01 | 71 |
| LTVGFTDSRKISHSC | 436 | 450 | 15 | HLA-DRB1*07:01 | 71 |
| KDNFNVYKATRPYLA | 328 | 342 | 15 | HLA-DRB1*07:01 | 72 |
| DNFNVYKATRPYLAH | 329 | 343 | 15 | HLA-DRB1*07:01 | 72 |
| KGETLTVGFTDSRKI | 432 | 446 | 15 | HLA-DRB1*07:01 | 73 |
| PTMTVVVVSVASFIL | 689 | 703 | 15 | HLA-DRB1*15:01 | 74 |
| LSLICCIRTAKAATY | 737 | 751 | 15 | HLA-DRB5*01:01 | 74 |
| ICCIRTAKAATYQEA | 740 | 754 | 15 | HLA-DRB5*01:01 | 74 |
| GETLTVGFTDSRKIS | 433 | 447 | 15 | HLA-DRB1*07:01 | 74 |
| SLICCIRTAKAATYQ | 738 | 752 | 15 | HLA-DRB5*01:01 | 75 |
| LICCIRTAKAATYQE | 739 | 753 | 15 | HLA-DRB5*01:01 | 75 |
| LFWLQALIPLAALIV | 765 | 779 | 15 | HLA-DRB1*15:01 | 76 |
| WLQALIPLAALIVLC | 767 | 781 | 15 | HLA-DRB1*15:01 | 76 |
| CCIRTAKAATYQEAA | 741 | 755 | 15 | HLA-DRB5*01:01 | 77 |
| NFNVYKATRPYLAHC | 330 | 344 | 15 | HLA-DRB1*07:01 | 77 |
| LQALIPLAALIVLCN | 768 | 782 | 15 | HLA-DRB1*15:01 | 78 |
| GQLAQLISAVNKLTM | 39 | 53 | 15 | HLA-DRB5*01:01 | 79 |
| VMRPGYYQLLQASLT | 303 | 317 | 15 | HLA-DRB5*01:01 | 80 |
| FNVYKATRPYLAHCP | 331 | 345 | 15 | HLA-DRB1*07:01 | 80 |
| FTDSRKISHSCTHPF | 440 | 454 | 15 | HLA-DRB1*07:01 | 80 |
| AGQLAQLISAVNKLT | 38 | 52 | 15 | HLA-DRB5*01:01 | 81 |
| TDSRKISHSCTHPFH | 441 | 455 | 15 | HLA-DRB1*07:01 | 81 |
| DSRKISHSCTHPFHH | 442 | 456 | 15 | HLA-DRB1*07:01 | 81 |
| MRPGYYQLLQASLTC | 304 | 318 | 15 | HLA-DRB5*01:01 | 82 |
| RPGYYQLLQASLTCS | 305 | 319 | 15 | HLA-DRB5*01:01 | 82 |
| QYSGGRFTIPTGAGK | 195 | 209 | 15 | HLA-DRB5*01:01 | 83 |
| RAGLFVRTSAPCTIT | 405 | 419 | 15 | HLA-DRB1*07:01 | 83 |
| SRKISHSCTHPFHHD | 443 | 457 | 15 | HLA-DRB1*07:01 | 83 |
| QALIPLAALIVLCNC | 769 | 783 | 15 | HLA-DRB1*15:01 | 84 |
| GRAGLFVRTSAPCTI | 404 | 418 | 15 | HLA-DRB1*07:01 | 84 |
| PGYYQLLQASLTCSP | 306 | 320 | 15 | HLA-DRB5*01:01 | 85 |
| NVYKATRPYLAHCPD | 332 | 346 | 15 | HLA-DRB1*07:01 | 85 |
| RKISHSCTHPFHHDP | 444 | 458 | 15 | HLA-DRB1*07:01 | 85 |
| QLAQLISAVNKLTMR | 40 | 54 | 15 | HLA-DRB5*01:01 | 86 |
| LAQLISAVNKLTMRA | 41 | 55 | 15 | HLA-DRB5*01:01 | 87 |
| YSGGRFTIPTGAGKP | 196 | 210 | 15 | HLA-DRB5*01:01 | 87 |
| DLAKLAFKRSSKYDL | 148 | 162 | 15 | HLA-DRB5*01:01 | 88 |
| LAKLAFKRSSKYDLE | 149 | 163 | 15 | HLA-DRB5*01:01 | 88 |
| AGLFVRTSAPCTITG | 406 | 420 | 15 | HLA-DRB1*07:01 | 88 |
| SGGRFTIPTGAGKPG | 197 | 211 | 15 | HLA-DRB5*01:01 | 90 |
| TLKIQVSLQIGIGTD | 370 | 384 | 15 | HLA-DRB1*07:01 | 90 |
| GLFVRTSAPCTITGT | 407 | 421 | 15 | HLA-DRB1*07:01 | 90 |
| AQLISAVNKLTMRAV | 42 | 56 | 15 | HLA-DRB5*01:01 | 91 |
| AKLAFKRSSKYDLEC | 150 | 164 | 15 | HLA-DRB5*01:01 | 91 |
| GGRFTIPTGAGKPGD | 198 | 212 | 15 | HLA-DRB5*01:01 | 91 |
| LKIQVSLQIGIGTDD | 371 | 385 | 15 | HLA-DRB1*07:01 | 91 |
| GYYQLLQASLTCSPH | 307 | 321 | 15 | HLA-DRB5*01:01 | 92 |
| TDGTLKIQVSLQIGI | 367 | 381 | 15 | HLA-DRB5*01:01 | 92 |
| EATDGTLKIQVSLQI | 365 | 379 | 15 | HLA-DRB5*01:01 | 94 |
| GTLKIQVSLQIGIGT | 369 | 383 | 15 | HLA-DRB5*01:01 | 94 |
| DGTLKIQVSLQIGIG | 368 | 382 | 15 | HLA-DRB5*01:01 | 95 |
| SVASFILLSMVGMAV | 697 | 711 | 15 | HLA-DRB1*07:01 | 95 |
| GRFTIPTGAGKPGDS | 199 | 213 | 15 | HLA-DRB5*01:01 | 96 |
| ATDGTLKIQVSLQIG | 366 | 380 | 15 | HLA-DRB5*01:01 | 97 |
| FILLSMVGMAVGMCM | 701 | 715 | 15 | HLA-DRB1*07:01 | 97 |
| LYYYELYPTMTVVVV | 682 | 696 | 15 | HLA-DRB1*07:01 | 99 |
| ASFILLSMVGMAVGM | 699 | 713 | 15 | HLA-DRB1*07:01 | 99 |
| QPLFWLQALIPLAAL | 763 | 777 | 15 | HLA-DRB4*01:01 | 100 |
| AGRAGLFVRTSAPCT | 403 | 417 | 15 | HLA-DRB1*07:01 | 102 |
| ILYYYELYPTMTVVV | 681 | 695 | 15 | HLA-DRB1*07:01 | 102 |
| SFILLSMVGMAVGMC | 700 | 714 | 15 | HLA-DRB1*07:01 | 102 |
| VASFILLSMVGMAVG | 698 | 712 | 15 | HLA-DRB1*07:01 | 103 |
| PLFWLQALIPLAALI | 764 | 778 | 15 | HLA-DRB4*01:01 | 105 |
| PHEIILYYYELYPTM | 677 | 691 | 15 | HLA-DRB1*15:01 | 105 |
| HEIILYYYELYPTMT | 678 | 692 | 15 | HLA-DRB1*15:01 | 105 |
| QQPLFWLQALIPLAA | 762 | 776 | 15 | HLA-DRB4*01:01 | 107 |
| HPHEIILYYYELYPT | 676 | 690 | 15 | HLA-DRB1*15:01 | 109 |
| MRPGYYQLLQASLTC | 304 | 318 | 15 | HLA-DRB1*07:01 | 111 |
| PQNNTNQKKQPPKKK | 77 | 91 | 15 | HLA-DRB5*01:01 | 112 |
| VMRPGYYQLLQASLT | 303 | 317 | 15 | HLA-DRB1*07:01 | 112 |
| RPGYYQLLQASLTCS | 305 | 319 | 15 | HLA-DRB1*07:01 | 112 |
| ALIPLAALIVLCNCL | 770 | 784 | 15 | HLA-DRB1*07:01 | 112 |
| KDNFNVYKATRPYLA | 328 | 342 | 15 | HLA-DRB5*01:01 | 113 |
| NTNQKKQPPKKKPAQ | 80 | 94 | 15 | HLA-DRB5*01:01 | 114 |
| LIPLAALIVLCNCLR | 771 | 785 | 15 | HLA-DRB1*07:01 | 114 |
| NNTNQKKQPPKKKPA | 79 | 93 | 15 | HLA-DRB5*01:01 | 115 |
| DNFNVYKATRPYLAH | 329 | 343 | 15 | HLA-DRB5*01:01 | 115 |
| TDGTLKIQVSLQIGI | 367 | 381 | 15 | HLA-DRB1*15:01 | 116 |
| PGYYQLLQASLTCSP | 306 | 320 | 15 | HLA-DRB1*07:01 | 116 |
| QNNTNQKKQPPKKKP | 78 | 92 | 15 | HLA-DRB5*01:01 | 117 |
| TNQKKQPPKKKPAQK | 81 | 95 | 15 | HLA-DRB5*01:01 | 117 |
| EIILYYYELYPTMTV | 679 | 693 | 15 | HLA-DRB1*15:01 | 118 |
| FPLANVTCMVPKARN | 584 | 598 | 15 | HLA-DRB5*01:01 | 118 |
| DGTLKIQVSLQIGIG | 368 | 382 | 15 | HLA-DRB1*15:01 | 119 |
| GTLKIQVSLQIGIGT | 369 | 383 | 15 | HLA-DRB1*15:01 | 119 |
| VVSVASFILLSMVGM | 695 | 709 | 15 | HLA-DRB1*15:01 | 119 |
| PFPLANVTCMVPKAR | 583 | 597 | 15 | HLA-DRB5*01:01 | 119 |
| YYYELYPTMTVVVVS | 683 | 697 | 15 | HLA-DRB1*07:01 | 120 |
| SVASFILLSMVGMAV | 697 | 711 | 15 | HLA-DRB1*15:01 | 121 |
| GYYQLLQASLTCSPH | 307 | 321 | 15 | HLA-DRB1*07:01 | 121 |
| EATDGTLKIQVSLQI | 365 | 379 | 15 | HLA-DRB1*15:01 | 122 |
| LANVTCMVPKARNPT | 586 | 600 | 15 | HLA-DRB5*01:01 | 123 |
| TLKIQVSLQIGIGTD | 370 | 384 | 15 | HLA-DRB4*01:01 | 124 |
| PLANVTCMVPKARNP | 585 | 599 | 15 | HLA-DRB5*01:01 | 124 |
| ATDGTLKIQVSLQIG | 366 | 380 | 15 | HLA-DRB1*15:01 | 125 |
| VASFILLSMVGMAVG | 698 | 712 | 15 | HLA-DRB1*15:01 | 125 |
| YELTPGATVPFLLSL | 725 | 739 | 15 | HLA-DRB1*07:01 | 127 |
| ANVTCMVPKARNPTV | 587 | 601 | 15 | HLA-DRB5*01:01 | 129 |
| PYELTPGATVPFLLS | 724 | 738 | 15 | HLA-DRB1*07:01 | 130 |
| EQQPLFWLQALIPLA | 761 | 775 | 15 | HLA-DRB4*01:01 | 132 |
| IILYYYELYPTMTVV | 680 | 694 | 15 | HLA-DRB1*07:01 | 132 |
| FWLQALIPLAALIVL | 766 | 780 | 15 | HLA-DRB4*01:01 | 135 |
| NEQQPLFWLQALIPL | 760 | 774 | 15 | HLA-DRB4*01:01 | 136 |
| LKIQVSLQIGIGTDD | 371 | 385 | 15 | HLA-DRB4*01:01 | 137 |
| KLAFKRSSKYDLECA | 151 | 165 | 15 | HLA-DRB5*01:01 | 137 |
| ASFILLSMVGMAVGM | 699 | 713 | 15 | HLA-DRB1*15:01 | 139 |
| GREKFHSRPQHGKEL | 462 | 476 | 15 | HLA-DRB5*01:01 | 140 |
| EIILYYYELYPTMTV | 679 | 693 | 15 | HLA-DRB1*07:01 | 140 |
| LAFKRSSKYDLECAQ | 152 | 166 | 15 | HLA-DRB5*01:01 | 142 |
| LFWLQALIPLAALIV | 765 | 779 | 15 | HLA-DRB4*01:01 | 143 |
| VVSVASFILLSMVGM | 695 | 709 | 15 | HLA-DRB4*01:01 | 144 |
| REKFHSRPQHGKELP | 463 | 477 | 15 | HLA-DRB5*01:01 | 147 |
| KPAQKKKKPGRRERM | 91 | 105 | 15 | HLA-DRB5*01:01 | 148 |
| CTITGTMGHFILARC | 416 | 430 | 15 | HLA-DRB1*07:01 | 148 |
| PKKKPAQKKKKPGRR | 88 | 102 | 15 | HLA-DRB5*01:01 | 150 |
| VIGREKFHSRPQHGK | 460 | 474 | 15 | HLA-DRB5*01:01 | 150 |
| IGREKFHSRPQHGKE | 461 | 475 | 15 | HLA-DRB5*01:01 | 150 |
| SAPCTITGTMGHFIL | 413 | 427 | 15 | HLA-DRB1*07:01 | 150 |
| APCTITGTMGHFILA | 414 | 428 | 15 | HLA-DRB1*07:01 | 150 |
| VSVASFILLSMVGMA | 696 | 710 | 15 | HLA-DRB4*01:01 | 151 |
| GHPHEIILYYYELYP | 675 | 689 | 15 | HLA-DRB1*15:01 | 151 |
| KKPAQKKKKPGRRER | 90 | 104 | 15 | HLA-DRB5*01:01 | 151 |
| STYVQSNAATAEEIE | 479 | 493 | 15 | HLA-DRB5*01:01 | 151 |
| PCTITGTMGHFILAR | 415 | 429 | 15 | HLA-DRB1*07:01 | 151 |
| SVASFILLSMVGMAV | 697 | 711 | 15 | HLA-DRB4*01:01 | 152 |
| IILYYYELYPTMTVV | 680 | 694 | 15 | HLA-DRB1*15:01 | 152 |
| PAQKKKKPGRRERMC | 92 | 106 | 15 | HLA-DRB5*01:01 | 153 |
| EKFHSRPQHGKELPC | 464 | 478 | 15 | HLA-DRB5*01:01 | 155 |
| CSTYVQSNAATAEEI | 478 | 492 | 15 | HLA-DRB5*01:01 | 155 |
| VVVSVASFILLSMVG | 694 | 708 | 15 | HLA-DRB1*15:01 | 156 |
| TKDNFNVYKATRPYL | 327 | 341 | 15 | HLA-DRB5*01:01 | 157 |
| YYELYPTMTVVVVSV | 684 | 698 | 15 | HLA-DRB1*07:01 | 158 |
| KKKPAQKKKKPGRRE | 89 | 103 | 15 | HLA-DRB5*01:01 | 159 |
| NPTVTYGKNQVIMLL | 598 | 612 | 15 | HLA-DRB1*07:01 | 161 |
| ALSVVTWNKDIVTKI | 239 | 253 | 15 | HLA-DRB3*01:01 | 162 |
| LSVVTWNKDIVTKIT | 240 | 254 | 15 | HLA-DRB3*01:01 | 162 |
| PCSTYVQSNAATAEE | 477 | 491 | 15 | HLA-DRB5*01:01 | 163 |
| PTVTYGKNQVIMLLY | 599 | 613 | 15 | HLA-DRB1*07:01 | 163 |
| ELPCSTYVQSNAATA | 475 | 489 | 15 | HLA-DRB5*01:01 | 164 |
| LPCSTYVQSNAATAE | 476 | 490 | 15 | HLA-DRB5*01:01 | 164 |
| TSAPCTITGTMGHFI | 412 | 426 | 15 | HLA-DRB1*07:01 | 164 |
| VVTWNKDIVTKITPE | 242 | 256 | 15 | HLA-DRB3*01:01 | 165 |
| NADLAKLAFKRSSKY | 146 | 160 | 15 | HLA-DRB5*01:01 | 165 |
| SVVTWNKDIVTKITP | 241 | 255 | 15 | HLA-DRB3*01:01 | 166 |
| AQLISAVNKLTMRAV | 42 | 56 | 15 | HLA-DRB1*07:01 | 166 |
| TVVVVSVASFILLSM | 692 | 706 | 15 | HLA-DRB5*01:01 | 167 |
| YELYPTMTVVVVSVA | 685 | 699 | 15 | HLA-DRB1*07:01 | 167 |
| VMRPGYYQLLQASLT | 303 | 317 | 15 | HLA-DRB4*01:01 | 169 |
| MTVVVVSVASFILLS | 691 | 705 | 15 | HLA-DRB5*01:01 | 169 |
| VTWNKDIVTKITPEG | 243 | 257 | 15 | HLA-DRB3*01:01 | 170 |
| TMTVVVVSVASFILL | 690 | 704 | 15 | HLA-DRB5*01:01 | 170 |
| VASFILLSMVGMAVG | 698 | 712 | 15 | HLA-DRB4*01:01 | 172 |
| ASFILLSMVGMAVGM | 699 | 713 | 15 | HLA-DRB4*01:01 | 172 |
| LFVRTSAPCTITGTM | 408 | 422 | 15 | HLA-DRB1*07:01 | 173 |
| RPGYYQLLQASLTCS | 305 | 319 | 15 | HLA-DRB4*01:01 | 174 |
| GQLAQLISAVNKLTM | 39 | 53 | 15 | HLA-DRB1*07:01 | 174 |
| STKDNFNVYKATRPY | 326 | 340 | 15 | HLA-DRB5*01:01 | 175 |
| MRPGYYQLLQASLTC | 304 | 318 | 15 | HLA-DRB4*01:01 | 176 |
| SFILLSMVGMAVGMC | 700 | 714 | 15 | HLA-DRB1*15:01 | 176 |
| QLAQLISAVNKLTMR | 40 | 54 | 15 | HLA-DRB1*07:01 | 176 |
| RNPTVTYGKNQVIML | 597 | 611 | 15 | HLA-DRB1*07:01 | 176 |
| LAQLISAVNKLTMRA | 41 | 55 | 15 | HLA-DRB1*07:01 | 177 |
| VSVASFILLSMVGMA | 696 | 710 | 15 | HLA-DRB1*15:01 | 178 |
| PLAALIVLCNCLRLL | 773 | 787 | 15 | HLA-DRB5*01:01 | 178 |
| SGNVKITVNSQTVRY | 510 | 524 | 15 | HLA-DRB1*07:01 | 181 |
| ERMCMKIENDCIFEV | 103 | 117 | 15 | HLA-DRB3*01:01 | 183 |
| FILLSMVGMAVGMCM | 701 | 715 | 15 | HLA-DRB1*15:01 | 184 |
| PTMTVVVVSVASFIL | 689 | 703 | 15 | HLA-DRB5*01:01 | 184 |
| GNVKITVNSQTVRYK | 511 | 525 | 15 | HLA-DRB1*07:01 | 184 |
| RMCMKIENDCIFEVK | 104 | 118 | 15 | HLA-DRB3*01:01 | 185 |
| QSGNVKITVNSQTVR | 509 | 523 | 15 | HLA-DRB1*07:01 | 185 |
| LAALIVLCNCLRLLP | 774 | 788 | 15 | HLA-DRB5*01:01 | 186 |
| QQSGNVKITVNSQTV | 508 | 522 | 15 | HLA-DRB1*07:01 | 186 |
| MCMKIENDCIFEVKH | 105 | 119 | 15 | HLA-DRB3*01:01 | 187 |
| SDASKFTHEKPEGYY | 173 | 187 | 15 | HLA-DRB5*01:01 | 188 |
| DASKFTHEKPEGYYN | 174 | 188 | 15 | HLA-DRB5*01:01 | 188 |
| RSTKDNFNVYKATRP | 325 | 339 | 15 | HLA-DRB5*01:01 | 188 |
| NFNVYKATRPYLAHC | 330 | 344 | 15 | HLA-DRB5*01:01 | 190 |
| PGYYQLLQASLTCSP | 306 | 320 | 15 | HLA-DRB4*01:01 | 191 |
| TVGFTDSRKISHSCT | 437 | 451 | 15 | HLA-DRB1*07:01 | 191 |
| VGFTDSRKISHSCTH | 438 | 452 | 15 | HLA-DRB1*07:01 | 191 |
| KSDASKFTHEKPEGY | 172 | 186 | 15 | HLA-DRB5*01:01 | 192 |
| IPLAALIVLCNCLRL | 772 | 786 | 15 | HLA-DRB5*01:01 | 193 |
| ASKFTHEKPEGYYNW | 175 | 189 | 15 | HLA-DRB5*01:01 | 195 |
| LIPLAALIVLCNCLR | 771 | 785 | 15 | HLA-DRB1*15:01 | 196 |
| AALIVLCNCLRLLPC | 775 | 789 | 15 | HLA-DRB5*01:01 | 196 |
| LQALIPLAALIVLCN | 768 | 782 | 15 | HLA-DRB4*01:01 | 197 |
| WLQALIPLAALIVLC | 767 | 781 | 15 | HLA-DRB4*01:01 | 198 |
| SLAIPVMCLLANTTF | 262 | 276 | 15 | HLA-DRB1*07:01 | 199 |
| ATVPFLLSLICCIRT | 731 | 745 | 15 | HLA-DRB1*07:01 | 199 |
| ALIPLAALIVLCNCL | 770 | 784 | 15 | HLA-DRB1*15:01 | 200 |
| AGQLAQLISAVNKLT | 38 | 52 | 15 | HLA-DRB1*07:01 | 200 |
| SKFTHEKPEGYYNWH | 176 | 190 | 15 | HLA-DRB5*01:01 | 201 |
| TVPFLLSLICCIRTA | 732 | 746 | 15 | HLA-DRB1*07:01 | 202 |
| FNVYKATRPYLAHCP | 331 | 345 | 15 | HLA-DRB5*01:01 | 203 |
| VPFLLSLICCIRTAK | 733 | 747 | 15 | HLA-DRB1*07:01 | 203 |
| AIPVMCLLANTTFPC | 264 | 278 | 15 | HLA-DRB1*07:01 | 204 |
| IPVMCLLANTTFPCS | 265 | 279 | 15 | HLA-DRB1*07:01 | 205 |
| RTLLSQQSGNVKITV | 503 | 517 | 15 | HLA-DRB1*07:01 | 205 |
| LAIPVMCLLANTTFP | 263 | 277 | 15 | HLA-DRB1*07:01 | 206 |
| RTLLSQQSGNVKITV | 503 | 517 | 15 | HLA-DRB1*15:01 | 208 |
| FWLQALIPLAALIVL | 766 | 780 | 15 | HLA-DRB5*01:01 | 208 |
| DRTLLSQQSGNVKIT | 502 | 516 | 15 | HLA-DRB1*15:01 | 209 |
| RTLLSQQSGNVKITV | 503 | 517 | 15 | HLA-DRB5*01:01 | 210 |
| GNVKITVNSQTVRYK | 511 | 525 | 15 | HLA-DRB5*01:01 | 210 |
| PDRTLLSQQSGNVKI | 501 | 515 | 15 | HLA-DRB1*15:01 | 211 |
| MAVGMCMCARRRCIT | 709 | 723 | 15 | HLA-DRB5*01:01 | 212 |
| DRTLLSQQSGNVKIT | 502 | 516 | 15 | HLA-DRB1*07:01 | 212 |
| ARNPTVTYGKNQVIM | 596 | 610 | 15 | HLA-DRB1*07:01 | 213 |
| GMAVGMCMCARRRCI | 708 | 722 | 15 | HLA-DRB5*01:01 | 214 |
| PDRTLLSQQSGNVKI | 501 | 515 | 15 | HLA-DRB1*07:01 | 214 |
| WTPRPTIQVIRPRPR | 19 | 33 | 15 | HLA-DRB4*01:01 | 215 |
| VVVVSVASFILLSMV | 693 | 707 | 15 | HLA-DRB5*01:01 | 215 |
| SVASFILLSMVGMAV | 697 | 711 | 15 | HLA-DRB5*01:01 | 215 |
| AFKRSSKYDLECAQI | 153 | 167 | 15 | HLA-DRB1*07:01 | 215 |
| LFWLQALIPLAALIV | 765 | 779 | 15 | HLA-DRB5*01:01 | 216 |
| IRTAKAATYQEAAVY | 743 | 757 | 15 | HLA-DRB5*01:01 | 217 |
| RPTIQVIRPRPRPQR | 22 | 36 | 15 | HLA-DRB4*01:01 | 218 |
| GYYQLLQASLTCSPH | 307 | 321 | 15 | HLA-DRB4*01:01 | 218 |
| QALIPLAALIVLCNC | 769 | 783 | 15 | HLA-DRB4*01:01 | 218 |
| DRTLLSQQSGNVKIT | 502 | 516 | 15 | HLA-DRB5*01:01 | 218 |
| SQQSGNVKITVNSQT | 507 | 521 | 15 | HLA-DRB1*07:01 | 218 |
| TPRPTIQVIRPRPRP | 20 | 34 | 15 | HLA-DRB4*01:01 | 219 |
| FKRSSKYDLECAQIP | 154 | 168 | 15 | HLA-DRB1*07:01 | 219 |
| TPDRTLLSQQSGNVK | 500 | 514 | 15 | HLA-DRB1*15:01 | 220 |
| PDRTLLSQQSGNVKI | 501 | 515 | 15 | HLA-DRB5*01:01 | 220 |
| MVGMAVGMCMCARRR | 706 | 720 | 15 | HLA-DRB5*01:01 | 220 |
| PVMCLLANTTFPCSQ | 266 | 280 | 15 | HLA-DRB1*07:01 | 220 |
| PRPTIQVIRPRPRPQ | 21 | 35 | 15 | HLA-DRB4*01:01 | 221 |
| NVKITVNSQTVRYKC | 512 | 526 | 15 | HLA-DRB5*01:01 | 222 |
| VASFILLSMVGMAVG | 698 | 712 | 15 | HLA-DRB5*01:01 | 222 |
| AVGMCMCARRRCITP | 710 | 724 | 15 | HLA-DRB5*01:01 | 222 |
| CIRTAKAATYQEAAV | 742 | 756 | 15 | HLA-DRB5*01:01 | 222 |
| ADLAKLAFKRSSKYD | 147 | 161 | 15 | HLA-DRB5*01:01 | 223 |
| ASFILLSMVGMAVGM | 699 | 713 | 15 | HLA-DRB5*01:01 | 224 |
| KARNPTVTYGKNQVI | 595 | 609 | 15 | HLA-DRB1*07:01 | 224 |
| VGMAVGMCMCARRRC | 707 | 721 | 15 | HLA-DRB5*01:01 | 225 |
| LIPLAALIVLCNCLR | 771 | 785 | 15 | HLA-DRB5*01:01 | 225 |
| PTIQVIRPRPRPQRQ | 23 | 37 | 15 | HLA-DRB4*01:01 | 227 |
| VKITVNSQTVRYKCN | 513 | 527 | 15 | HLA-DRB5*01:01 | 229 |
| ILLSMVGMAVGMCMC | 702 | 716 | 15 | HLA-DRB1*07:01 | 229 |
| TPDRTLLSQQSGNVK | 500 | 514 | 15 | HLA-DRB5*01:01 | 232 |
| LQALIPLAALIVLCN | 768 | 782 | 15 | HLA-DRB5*01:01 | 236 |
| NQVIMLLYPDHPTLL | 606 | 620 | 15 | HLA-DRB1*15:01 | 238 |
| WLQALIPLAALIVLC | 767 | 781 | 15 | HLA-DRB5*01:01 | 238 |
| TLLSQQSGNVKITVN | 504 | 518 | 15 | HLA-DRB5*01:01 | 240 |
| LLSMVGMAVGMCMCA | 703 | 717 | 15 | HLA-DRB1*07:01 | 241 |
| AQLISAVNKLTMRAV | 42 | 56 | 15 | HLA-DRB4*01:01 | 242 |
| DTPDRTLLSQQSGNV | 499 | 513 | 15 | HLA-DRB1*15:01 | 242 |
| QAGQLAQLISAVNKL | 37 | 51 | 15 | HLA-DRB5*01:01 | 243 |
| KITVNSQTVRYKCNC | 514 | 528 | 15 | HLA-DRB5*01:01 | 243 |
| VIMLLYPDHPTLLSY | 608 | 622 | 15 | HLA-DRB1*07:01 | 243 |
| IMLLYPDHPTLLSYR | 609 | 623 | 15 | HLA-DRB1*07:01 | 243 |
| QPLFWLQALIPLAAL | 763 | 777 | 15 | HLA-DRB1*07:01 | 244 |
| QKKQPPKKKPAQKKK | 83 | 97 | 15 | HLA-DRB5*01:01 | 245 |
| PLFWLQALIPLAALI | 764 | 778 | 15 | HLA-DRB1*07:01 | 246 |
| NQKKQPPKKKPAQKK | 82 | 96 | 15 | HLA-DRB5*01:01 | 248 |
| PGATVPFLLSLICCI | 729 | 743 | 15 | HLA-DRB1*07:01 | 248 |
| QVIMLLYPDHPTLLS | 607 | 621 | 15 | HLA-DRB1*07:01 | 249 |
| NQVIMLLYPDHPTLL | 606 | 620 | 15 | HLA-DRB1*07:01 | 250 |
