# Supplementary material for: Implementation of in silico methods to predict common epitopes for vaccine development against Chikungunya and Mayaro viruses
Source: Heliyon. 2021 Mar 8;7(3):e06396. doi: 10.1016/j.heliyon.2021.e06396 (PMC7944042; doi:10.1016/j.heliyon.2021.e06396)
Supplement: Supplementary Table 3 [file mmc4.docx]

**Supplementary Table 3:** Potential B cell epitopes predicted from CHIKV Frameshifted Structural Polyprotein

| **B Cell Epitope** | **Score** |
| --- | --- |
|  |  |
| KQPPKKKPAQKKKKPGRRER | 1 |
| GGRFTIPTGAGKPGDSGRPI | 1 |
| KPRKNRKNKKQKQKQQAPQN | 1 |
| TPRPTIQVIRPRPRPQRQAG | 1 |
| AEEIEVHMPPDTPDRTLLSQ | 0.998 |
| PPVIGREKFHSRPQHGKELP | 0.986 |
| FPCSQPPCIPCCYEKEPEET | 0.984 |
| VVTWNKDIVTKITPEGAEEW | 0.98 |
| MGEEPNYQEEWVTHKKEVVL | 0.974 |
| KCNCGGSNEGLITTDKVINN | 0.971 |
| KFTHEKPEGYYNWHHGAVQY | 0.968 |
| PLVPRNAELGDRKGKIHIPF | 0.966 |
| VTWGNNEPYKYWPQLSANGT | 0.946 |
| CPHCERVRTRNSDPEHGGST | 0.935 |
| NVTCMVPKARNPTVTYGKNQ | 0.902 |
| ERIRNEATDGTLKIQVSLQI | 0.812 |
| SLICCIRTAKAATYQEAAVY | 0.787 |
| MCARRRCITPYELTPGATVP | 0.757 |
| LVGDKVMKPAHVKGTIDNAD | 0.752 |
| VRTSAPCTITGTMGHFILAR | 0.744 |
| DWTKLRYMDNHIPADAGRAG | 0.743 |
| YYYELYPTMTVVVVSVASFI | 0.733 |
