# Supplementary material for: Implementation of in silico methods to predict common epitopes for vaccine development against Chikungunya and Mayaro viruses
Source: Heliyon. 2021 Mar 8;7(3):e06396. doi: 10.1016/j.heliyon.2021.e06396 (PMC7944042; doi:10.1016/j.heliyon.2021.e06396)
Supplement: Supplementary Table 4 [file mmc5.docx]

**Supplementary Table 4:** Cross reaction possibility analysis of the conserved epitopes of both MAYV and CHIK with other arboviruses (ZIKV and DENV). All of the epitopes found 100% unique in the conservancy analysis.

| **CD8+ T cell Epitope (CTL)** | | |
| --- | --- | --- |
| **Epitope** | **Conservancy_Hit**  **(ZIKV*)** | **Conservancy_Hit**  **(DENV* -1,2,3,4)** |
| KVTGYACLV | 0.00% (1/1) | 0.00% (1/1) |
| ALSVVTWNK | 0.00% (1/1) | 0.00% (1/1) |
| KYDLECAQI | 0.00% (1/1) | 0.00% (1/1) |
| KPGDSGRPI | 0.00% (1/1) | 0.00% (1/1) |
| KPGRRERMC | 0.00% (1/1) | 0.00% (1/1) |
| RRERMCMKI | 0.00% (1/1) | 0.00% (1/1) |
| GRRERMCMK | 0.00% (1/1) | 0.00% (1/1) |
| TGTMGHFIL | 0.00% (1/1) | 0.00% (1/1) |
| FEVKHEGKV | 0.00% (1/1) | 0.00% (1/1) |
|  | | |
| **CD4+ T cell Epitope (HTL)** | | |
| **Epitope** | **Conservancy_Hit**  **(ZIKV)** | **Conservancy_Hit**  **(DENV -1,2,3,4)** |
| ALSVVTWNKDIVTKI | 0.00% (1/1) | 0.00% (1/1) |
| LSVVTWNKDIVTKIT | 0.00% (1/1) | 0.00% (1/1) |
| VVTWNKDIVTKITPE | 0.00% (1/1) | 0.00% (1/1) |
| SVVTWNKDIVTKITP | 0.00% (1/1) | 0.00% (1/1) |
| VTWNKDIVTKITPEG | 0.00% (1/1) | 0.00% (1/1) |
| MCMKIENDCIFEVKH | 0.00% (1/1) | 0.00% (1/1) |
| RTLLSQQSGNVKITV | 0.00% (1/1) | 0.00% (1/1) |
| DRTLLSQQSGNVKIT | 0.00% (1/1) | 0.00% (1/1) |
| PDRTLLSQQSGNVKI | 0.00% (1/1) | 0.00% (1/1) |
| TLLSQQSGNVKITVN | 0.00% (1/1) | 0.00% (1/1) |
| **B cell Epitope (BCL)** | | |
|  | | |
| **Epitope** | **Conservancy_Hit**  **(ZIKV)** | **Conservancy_Hit**  **(DENV -1,2,3,4)** |
| GGRFTIPTGAGKPGDSGRPI | 0.00% (1/1) | 0.00% (1/1) |
| LVGDKVMKPAHVKGTIDNAD | 0.00% (1/1) | 0.00% (1/1) |

*ZIKV: Zikavirus; *DENV: Dengue Virus
