# Supplementary material for: Implementation of in silico methods to predict common epitopes for vaccine development against Chikungunya and Mayaro viruses
Source: Heliyon. 2021 Mar 8;7(3):e06396. doi: 10.1016/j.heliyon.2021.e06396 (PMC7944042; doi:10.1016/j.heliyon.2021.e06396)
Supplement: Supplementary Table 5 [file mmc6.docx]

**Supplementary Table 5:** Conservancy analysis of epitopes in all the Variants of CHIKV and MAYV structural polyprotein

| **CD8+ T cell Epitope (CTL)** | | | |
| --- | --- | --- | --- |
| **Epitope** | **Conservancy_Hit**  **CHIKV** | **Conservancy_Hit**  **MAYV** | **Combined Score** |
| KVTGYACLV | 99.00% (99/100) | 75.00% (48/64) | 0.869 |
| ALSVVTWNK | 100.00% (100/100) | 75.00% (48/64) | 1.2483 |
| KYDLECAQI | 100.00% (100/100) | 75.00% (48/64) | 0.9517 |
| KPGDSGRPI | 100.00% (100/100) | 75.00% (48/64) | 1.6136 |
| KPGRRERMC | 100.00% (100/100) | 75.00% (48/64) | 0.8161 |
| RRERMCMKI | 100.00% (100/100) | 75.00% (48/64) | 1.2916 |
| GRRERMCMK | 100.00% (100/100) | 75.00% (48/64) | 0.9834 |
| TGTMGHFIL | 99.00% (99/100) | 76.56% (49/64) | 0.9569 |
| FEVKHEGKV | 86.00% (86/100) | 75.00% (48/64) | 1.0928 |
|  | | | |
| **CD4+ T cell Epitope (HTL)** | | | |
| **Epitope** | **Conservancy_Hit**  **CHIKV** | **Conservancy_Hit**  **MAYV** | **SMM IC50 Value** |
| ALSVVTWNKDIVTKI | 100.00% (100/100) | 75.00% (48/64) | 162 |
| LSVVTWNKDIVTKIT | 100.00% (100/100) | 75.00% (48/64) | 162 |
| VVTWNKDIVTKITPE | 100.00% (100/100) | 75.00% (48/64) | 165 |
| SVVTWNKDIVTKITP | 100.00% (100/100) | 75.00% (48/64) | 166 |
| VTWNKDIVTKITPEG | 100.00% (100/100) | 75.00% (48/64) | 170 |
| MCMKIENDCIFEVKH | 100.00% (100/100) | 75.00% (48/64) | 187 |
| RTLLSQQSGNVKITV | 99.00% (99/100) | 73.44% (47/64) | 205 |
| DRTLLSQQSGNVKIT | 100.00% (100/100) | 73.44% (47/64) | 209 |
| PDRTLLSQQSGNVKI | 99.00% (99/100) | 73.44% (47/64) | 211 |
| TLLSQQSGNVKITVN | 99.00% (99/100) | 73.44% (47/64) | 240 |
|  |  |  |  |
| **B cell Epitope (BCL)** | | | |
| **Epitope** | **Conservancy_Hit**  **CHIKV** | **Conservancy_Hit**  **MAYV** | **Score** |
| GGRFTIPTGAGKPGDSGRPI | 99.00% (99/100) | 75.00% (48/64) | 1 |
| LVGDKVMKPAHVKGTIDNAD | 100.00% (100/100) | 75.00% (48/64) | 0.752 |
